# Supplementary material for: A broadly applicable protein-polymer adjuvant system for antiviral vaccines
Source: EMBO Mol Med. 2024 May 15;16(6):1451–83. doi: 10.1038/s44321-024-00076-4 (PMC11178928; doi:10.1038/s44321-024-00076-4)
Supplement: Supplementary file 1 — Appendix [file 44321_2024_76_MOESM1_ESM.pdf]

Appendix Data

Table of contents:

APPENDIX FIGURES	PAGE
Appendix Figure S1	2
Appendix Figure S2	3
Appendix Figure S3	4
Appendix Figure S4	5
Appendix Figure S5	6
Appendix Figure S6	7
Appendix Figure S7	8
Appendix Figure S8	9
Appendix Figure S9	10
Appendix Figure S10	11
Appendix Figure S11	12
Appendix Figure S12	13
Appendix Figure S13	14
Appendix Figure S14	15
Appendix Figure S15	16
Appendix Figure S16	17
Appendix Figure S17	18
Appendix Figure S18	19
Appendix Figure S19	20
Appendix Table S1	21

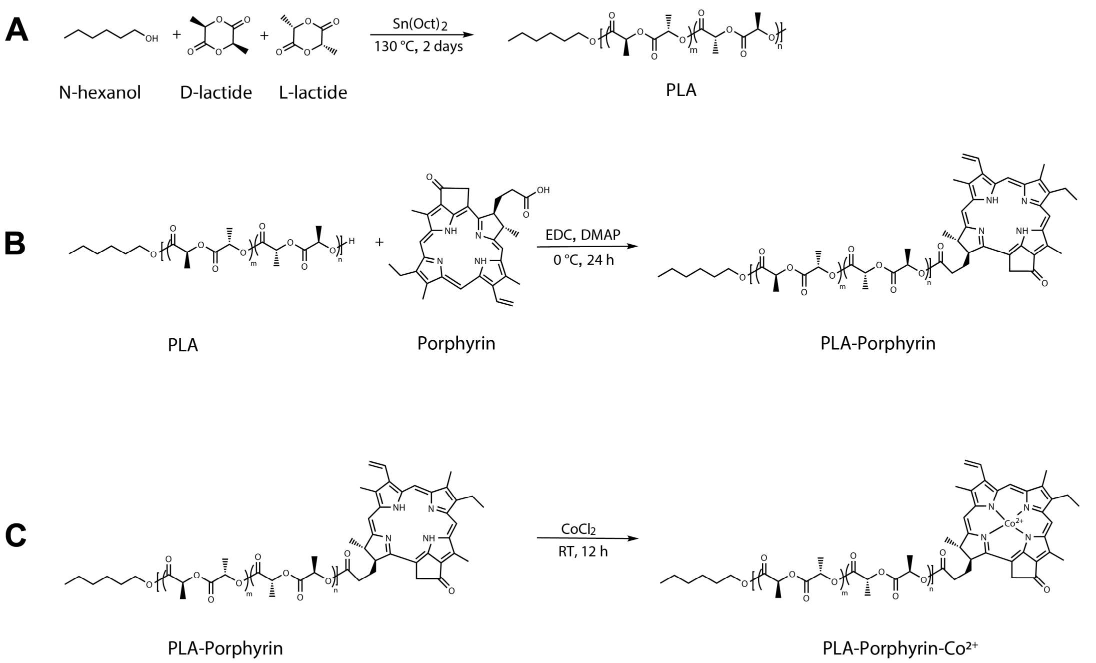

Appendix Figure S1. Synthetic route of PLA-Porphyrin-Co^{2+}.

(**A-C**) Synthetic route of poly(lactic acid) (PLA, **A**), PLA-Porphyrin(**B**), and PLA-Porphyrin-Co^{2+}(**C**).

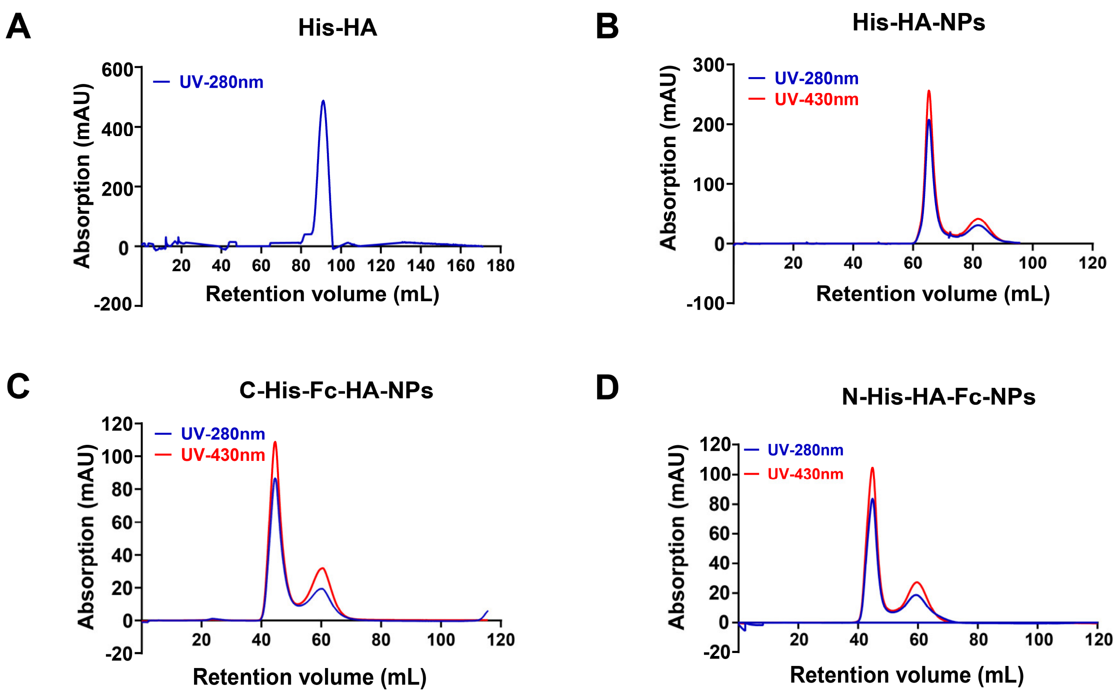

Appendix Figure S2. Purification of HA@PPCDQ nanovaccine.

(**A-D**) Size exclusion chromatography (SEC) of His-HA (**A**), His-HA-NPs (**B**), C-His-HA-Fc-NPs (**C**), and N-His-HA-Fc-NPs (**D**). The ultraviolet absorptions of protein and PPCDQ nanoparticles showed at 280 nm or 430 nm, respectively. The retention volume represented peaks of each nanoparticle.

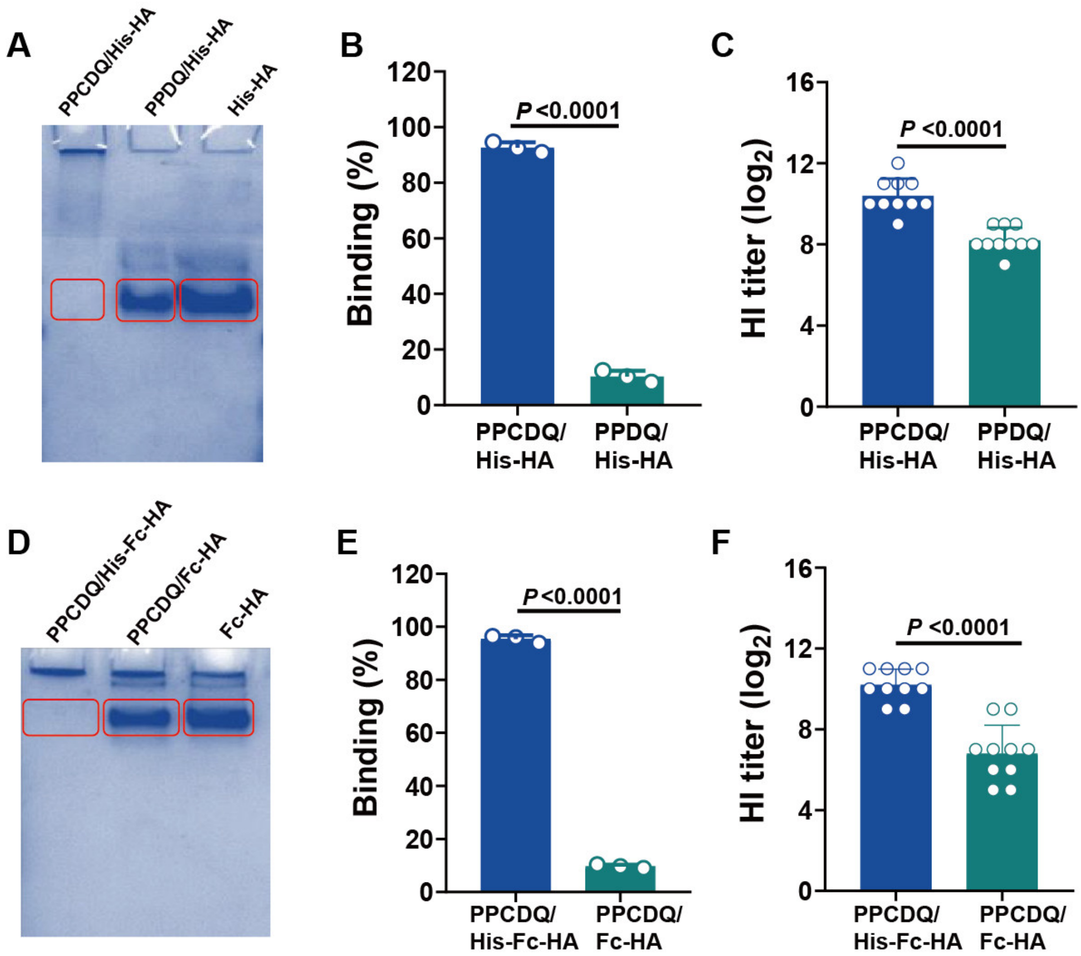

Appendix Figure S3. The conjugation between PPCDQ micelles and antigens is mediated by cobalt and His-tag.

(A-B) Native PAGE gel (A) and statistical graph of binding efficiency (B) of PPCDQ or PPDQ micelles to His-HA proteins (n = 3 biological replicates). **(C)** C57/BL6 mice were immunized with His-HA@PPCDQ or His-HA@PPDQ (without cobalt), respectively. And HI titers were detected at 3 weeks post vaccination (n = 10 animals per group). **(D-E)** Native PAGE gel (D) and statistical graph of binding efficiency (E) of PPCDQ micelles to C-His-Fc-HA or Fc-HA protein (n = 3 biological replicates). (**F**) HI titers of His-Fc-HA@PPCDQ and Fc-HA@ PPCDQ (without His-tag) at 3 weeks post vaccination (n = 10 animals per group). Data are presented as mean ± SD, Statistical significance was calculated by unpaired two-tailed Student' s t test.

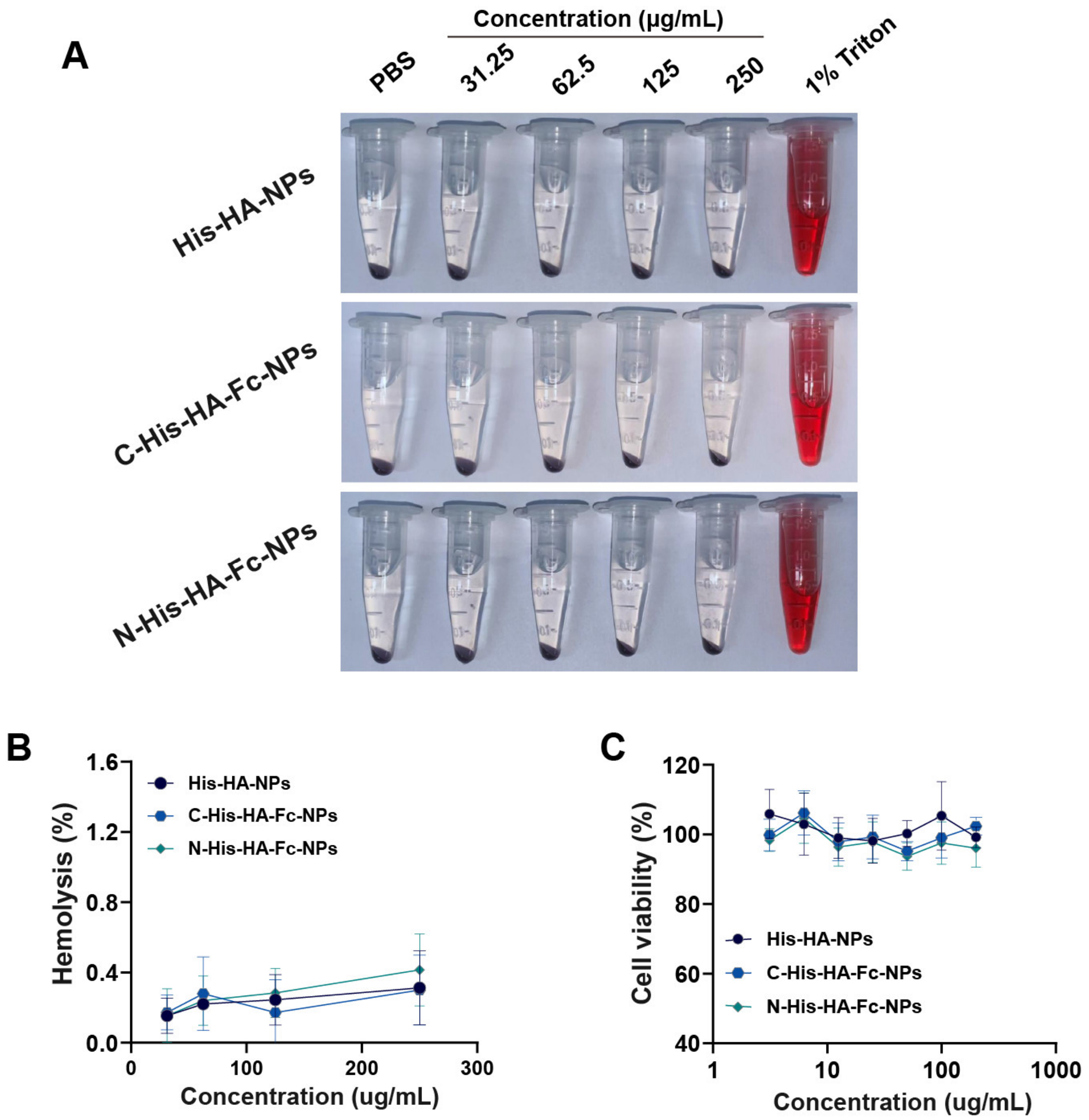

Appendix Figure S4. HA@PPCDQ cytotoxicity assays *in vitro*.

(A) *In vitro* hemolysis of His-HA-NPs, C-His-HA-NPs, and N-His-HA-NPs at different concentrations (quantified by the content of QS-21 in NPs). **(B)** Statistical graphs of hemolysis rates of different nanoparticles. Data are presented as the means ± SD, n = 3 biological replicates. (**C**) Relative cell viability of DC2.4 cells exposed to different concentrations (quantified by the content of PPCD micelles) of His-HA-NPs, C-His-HA-NPs, or N-His-HA-NPs. Data are presented as mean ± SD, n = 3 biological replicates.

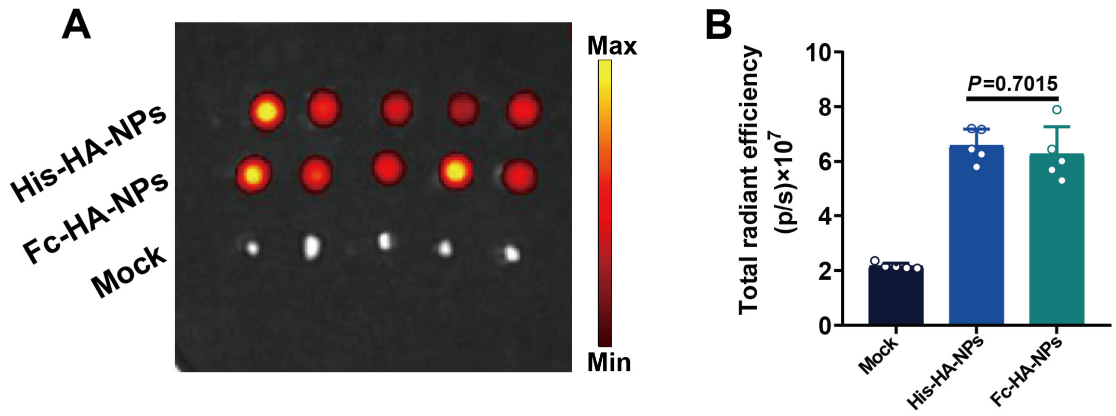

Appendix Figure S5. His-HA-NPs rapidly targeted the inguinal lymph nodes and drained to other lymph node.

(**A-B**) C57BL/6 mice were intramuscularly injected with equal mass of Cy7-tagged His-HA or His-HA-NPs. (**A**) ILNs were harvested at 4 h post injection for living image by IVIS Spectrum system (n = 5 animals per group). (**B**) Statistical graphs of the total radiant efficiency analyzed by Living Image Vision 4.4. Data are presented as mean ± SD. *P* values were calculated by one-way ANOVA with Tukey's multiple comparisons test.

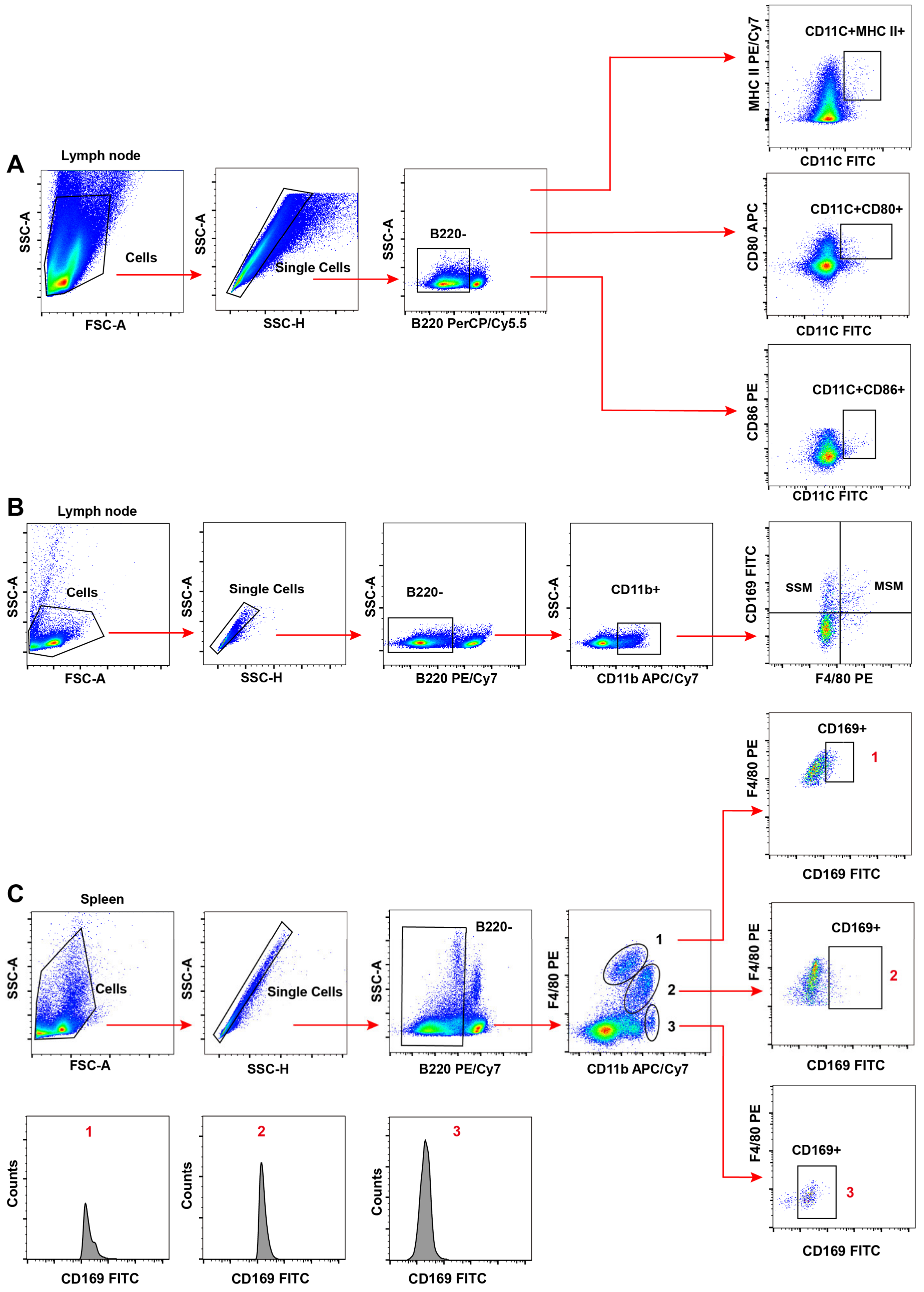

Appendix Figure S6. FCM gating strategy to identify DCs and macrophages.

In all analyses, cells were first gated to exclude debris. (**A**) Gating strategy to identify activated DCs in the inguinal LNs following intramuscular injection. (**B**) Gating strategy to identify SSMs and MSMs in the inguinal LNs. SSMs are $B220^{-}CD11b^{+}F4/80^{-}CD169^{+}$ and MSMs are $B220^{-}CD11b^{+}F4/80^{+}CD169^{+}$. (**C**) Gating strategy to identify tissue-resident macrophages ($B220^{-}F4/80^{hi}CD11b^{int}$), monocyte-derived macrophages ($B220^{-}F4/80^{int}CD11b^{hi}$) and neutrophils ($B220^{-}F4/80^{-}CD11b^{hi}$) in spleen. And the surface expression of CD169 by these three populations was further identified.

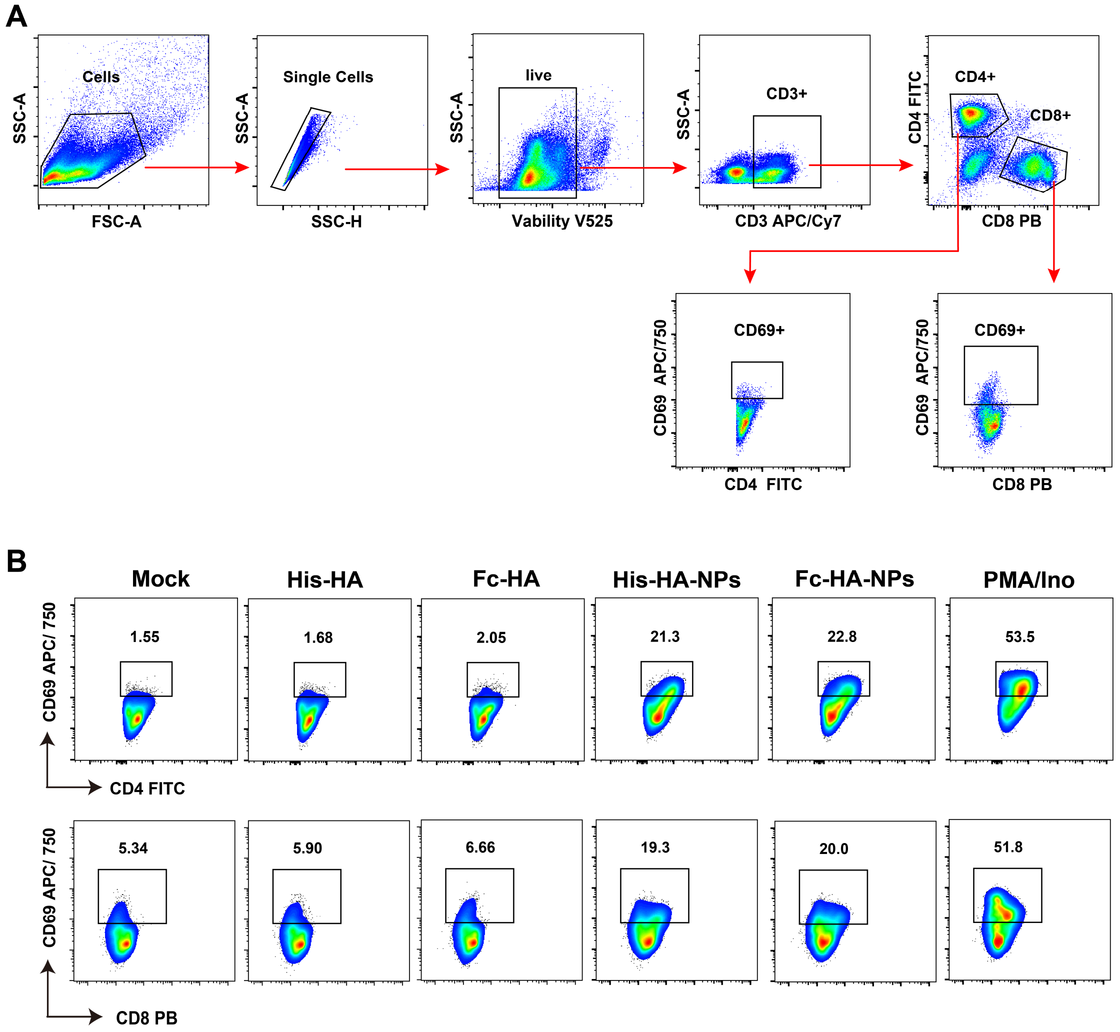

Appendix Figure S7. HA@PPCDQ promotes CD4^{+} T cells and CD8^{+} T cells activation.

(**A**) FCM gating strategy to identify CD69 positive T cells. (**B**) Representative flow cytometric plots of CD69 positive cells in CD4^{+} and CD8^{+} T cells populations.

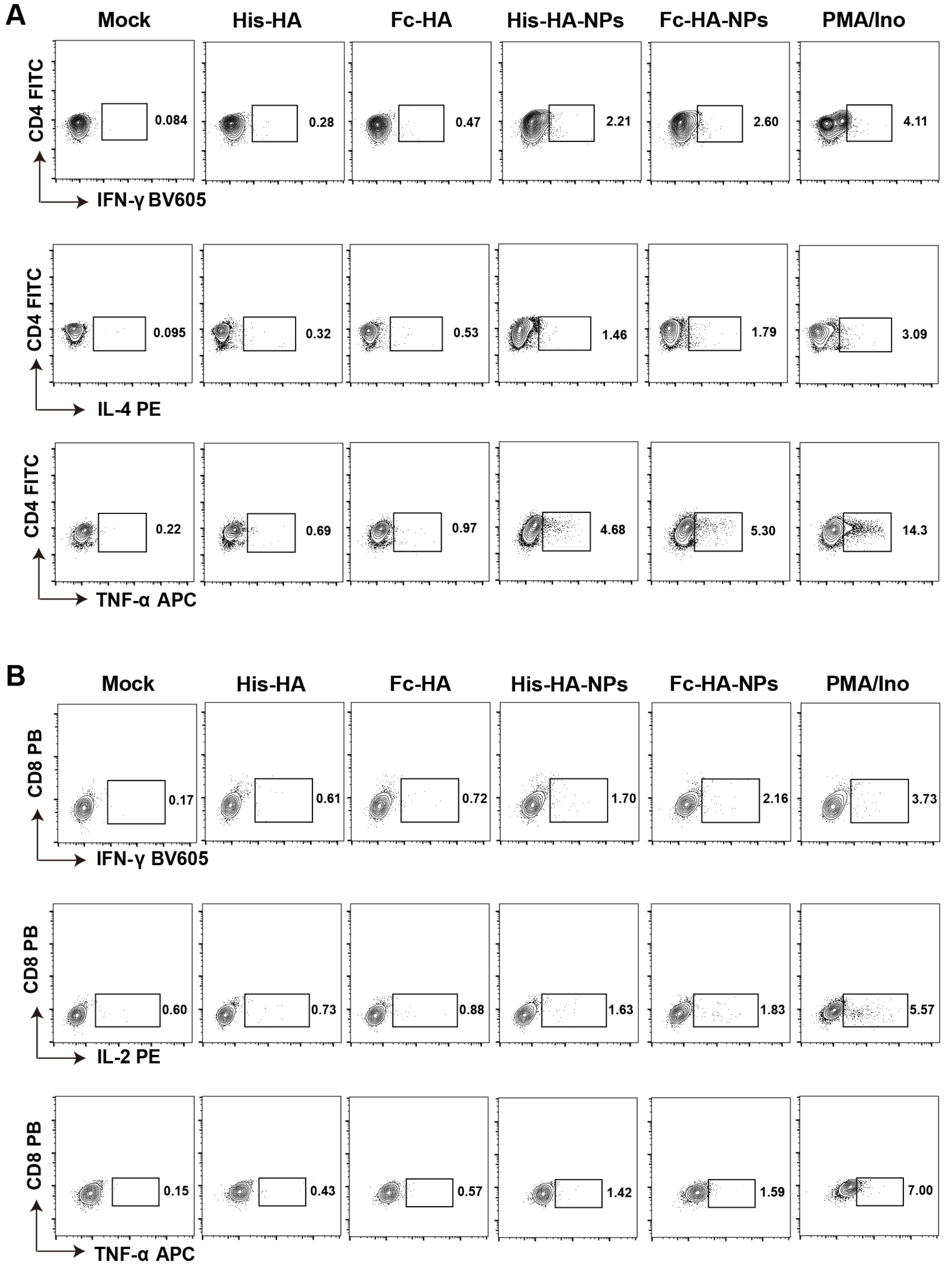

Appendix Figure S8. Representative flow cytometric plots of antigen-specific $CD4^+$ T cells and $CD8^+$ T cells.

(**A and B**) Splenocytes were stimulated with homologous His-tagged HA for 18 h, Golgi Stop and Golgi Plug were added 6 h before the end of the stimulation. Representative flow cytometric plots of HA-specific IFN-γ^+, IL-4^+ and TNF-α^+ in $CD4^+$ T cells (**A**), and IFN-γ^+, IL-2^+ and TNF-α^+ in $CD8^+$ T cells (**B**).

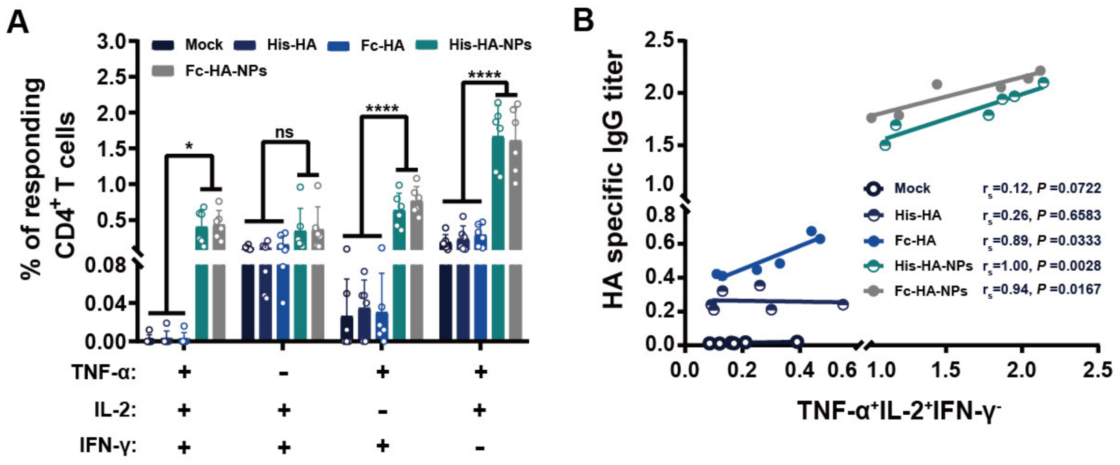

Appendix Figure S9. PPCDQ elicits robust CD4$^+$ T cell responses.

(**A**) Statistical results of TNFα$^+$IL-2$^+$ IFNγ$^+$, TNFα$^-$IL-2$^+$ IFNγ$^+$, TNFα$^+$IL-2$^-$ IFNγ$^+$ and TNFα$^+$IL-2$^+$ IFNγ$^-$ cells in CD4$^+$ T cells (n = 6 animals per group). Data are presented as mean ± SD, Statistical significance was calculated by one-way ANOVA with Tukey's multiple comparisons test. **P* < 0.05, *****P* < 0.0001. (**B**) Spearman's rank correlation technique was applied to evaluate the relationship between the frequency of TNFα$^+$IL-2$^+$ IFNγ$^-$ CD4$^+$ T cells in the spleen and HA specific IgG titer (n = 6 animals per group).

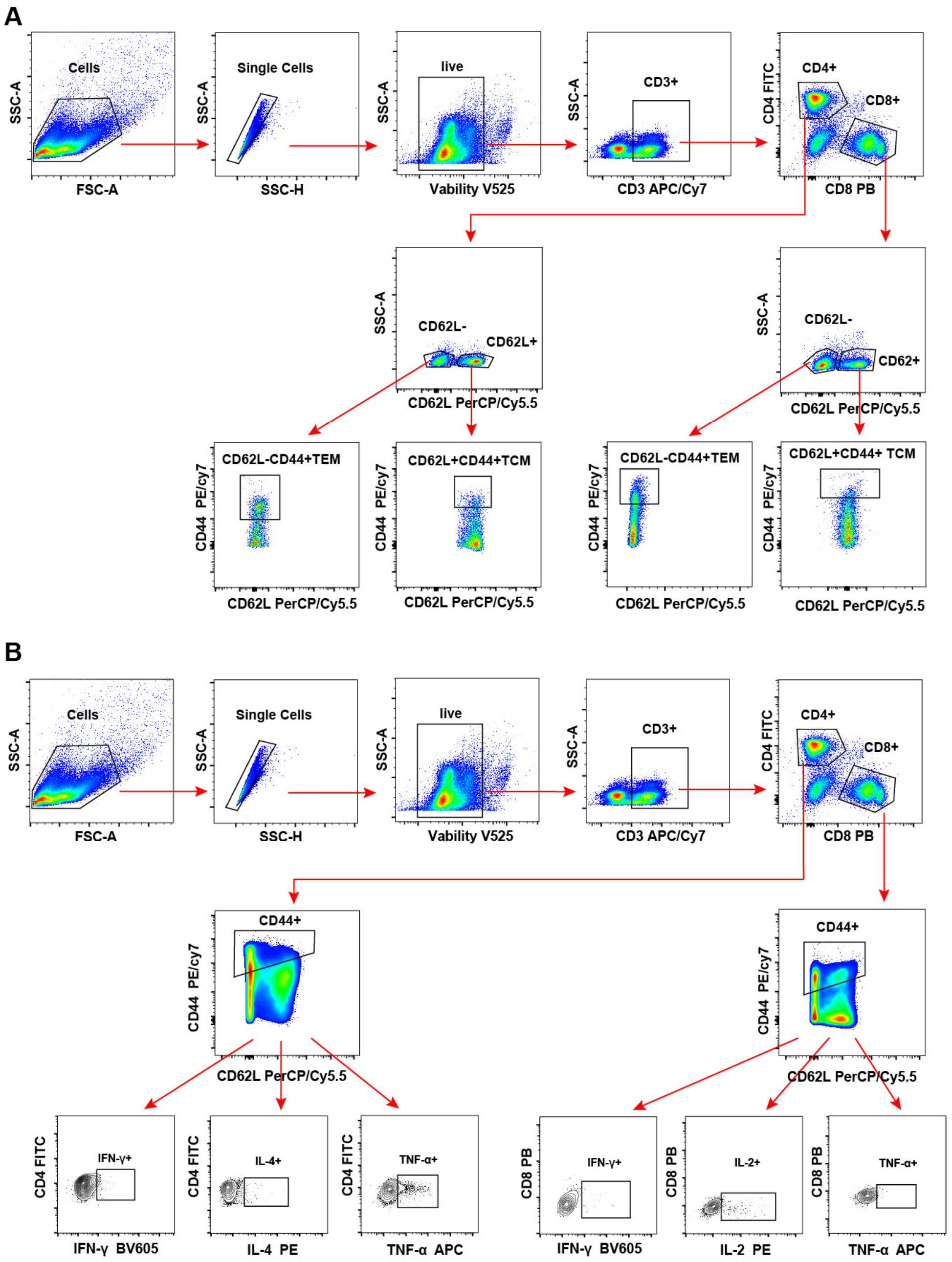

Appendix Figure S10. FCM gating strategy to identify TEMs, TCMs and antigen-specific CD4$^+$ T cells and CD8$^+$ T cells.

Cells were first gated to exclude debris and dead cells. (**A**) Gating strategy to identify TEMs (CD62L$^-$CD44$^+$) and TCMs (CD62L$^+$CD44$^+$) in CD4$^+$ T cells or CD8$^+$ T cells. (**B**) Antigen experienced CD4$^+$ T cells or CD8$^+$ T cells were distinguished based on the expression of CD44. HA-specific CD4$^+$ T cells were identified based on the expression of IFN-γ, IL-4 and TNF-α. HA-specific CD8$^+$ T cells were identified based on the expression of IFN-γ, IL-2 and TNF-α. The gating strategy for CD4$^+$ or CD8$^+$ T cells is consistent with Appendix Figure S7.

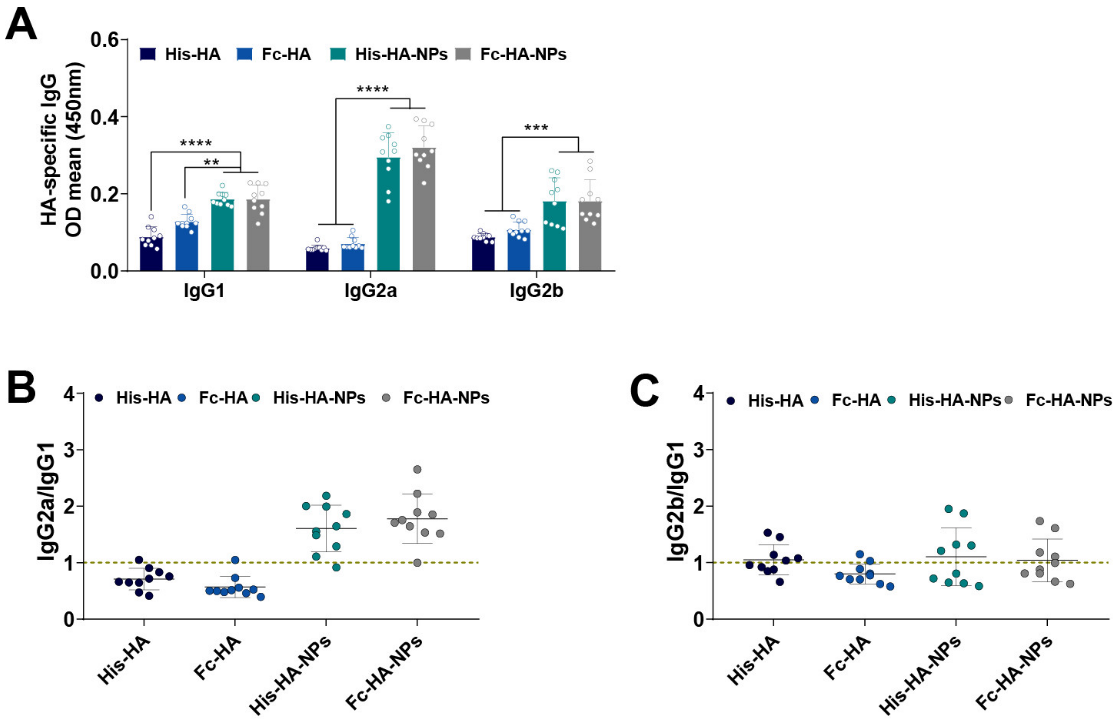

Appendix Figure S11. HA-specific IgG subtypes of HA@PPCDQ.

(**A-C**) Mice from each group were prime/boost-vaccinated (I.M.) with different vaccines at week 0 and week 2. Sera were collected at 3 weeks after immunization (n = 10 animals per group). (**A**) HA-specific IgG1, IgG2a and IgG2b were detected by ELISA with the same dilution. (**B**) The ratio of IgG2a and IgG1 in different vaccinated groups. (**C**) The ratio of IgG2b and IgG1 in different vaccinated groups. Data are shown as mean ± SD. Statistical significance in (**A**) was determined by one-way ANOVA with Tukey's multiple comparisons test. $^{**}P < 0.01$, $^{***}P < 0.001$, $^{****}P < 0.0001$.

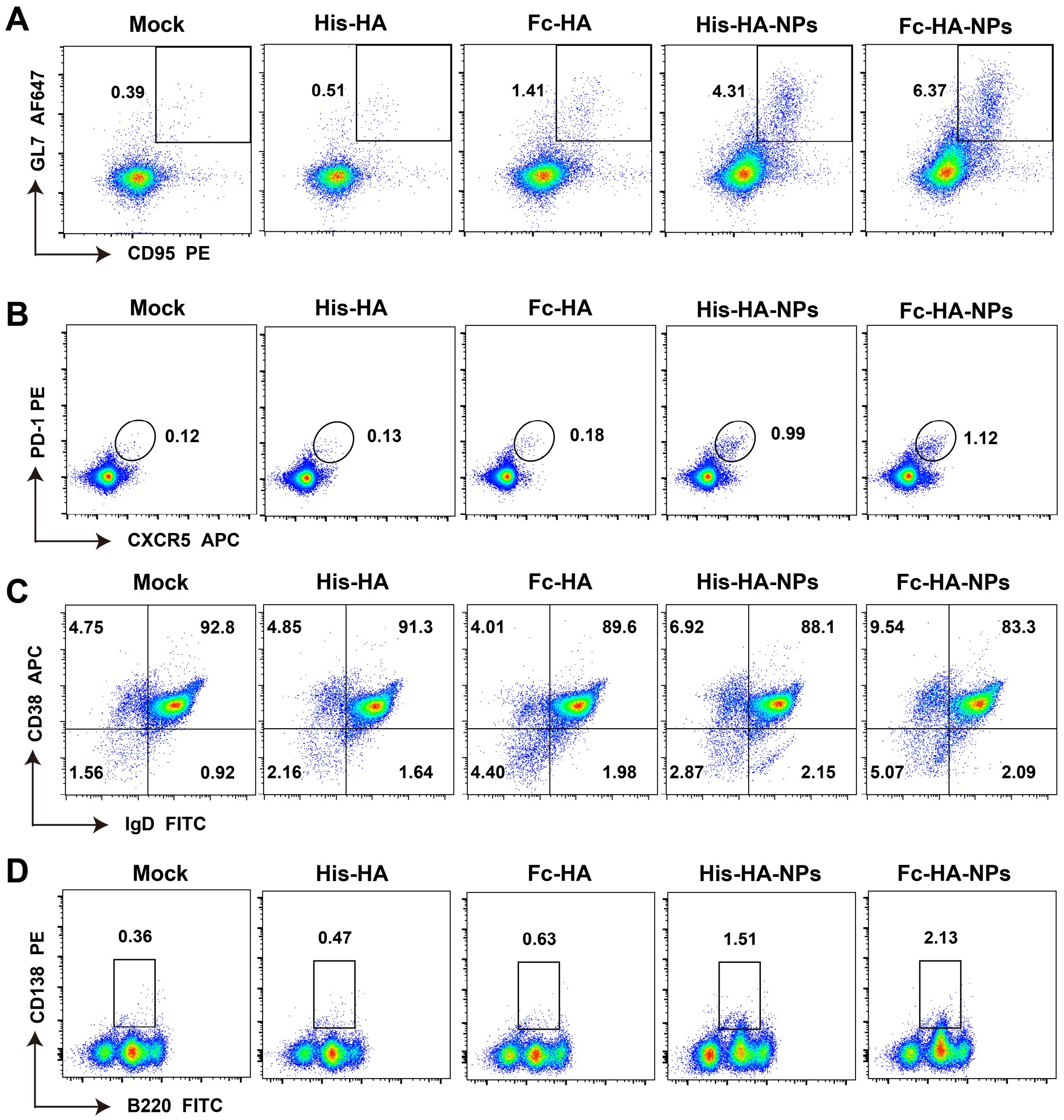

Appendix Figure S12. HA@PPCDQ induces potent germinal center response.

(**A-B**) Inguinal LNs at one weeks after boost vaccination were harvested for FCM analysis (n = 6 animals per group). (**A**) Representative flow cytometric plots of GC B cells ($B220^{+}CD95^{+}GL7^{+}$). (**B**) Representative flow cytometric plots of GC Tfh cells ($CXCR5^{+}PD1^{+}$). (**C**) Representative flow cytometric plots of switched MBCs ($B220^{+}IgD^{-}CD38^{+}$) at 2 weeks after boost vaccination. (**D**) Bone marrow was collected at 2 weeks after boost vaccination to evaluated long-live plasma cells (LLPCs, $B220^{lo}CD138^{+}$) (n = 6 animals per group).

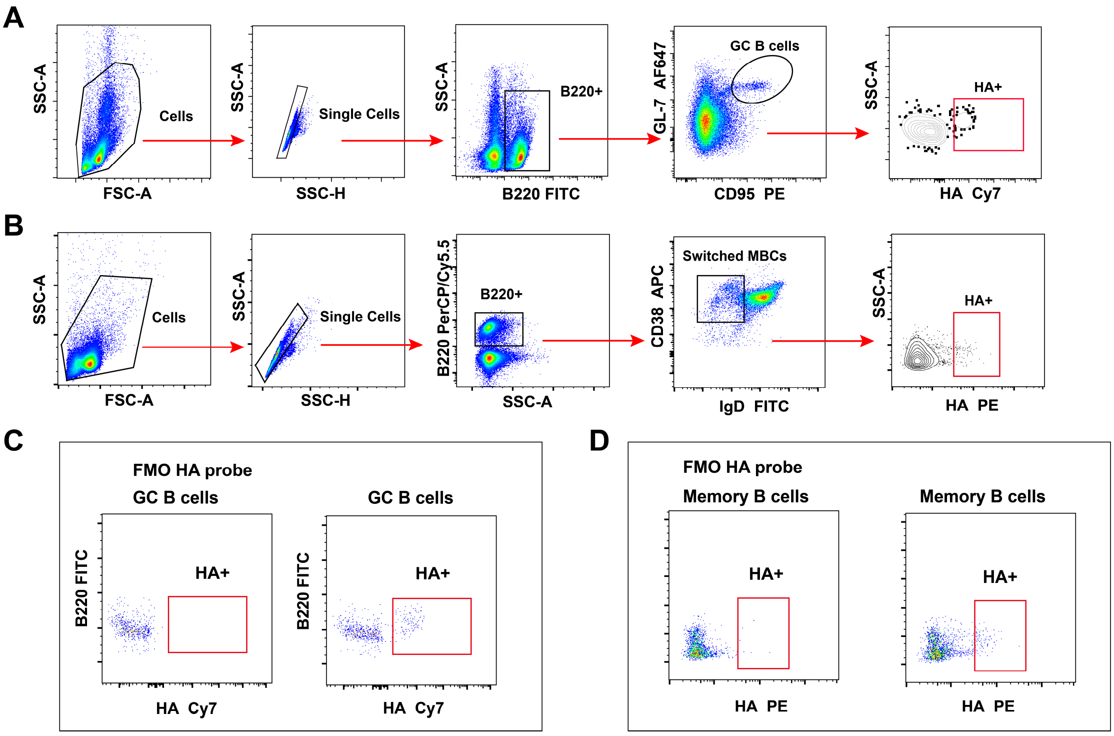

Appendix Figure S13. FCM gating strategy to identify HA-specific germinal center B cells and memory B cells.

(**A**) Gating strategy to identify HA-specific GC B cells ($B220^{+}CD95^{+}GL\text{-}7^{+}Cy7^{+}$). (**B)** Gating strategy to identify HA-specific MBCs ($B220^{+}IgD^{-}CD38^{+}PE^{+}$). (**C)** Fluorescence-Minus-One control (FMO) of HA-specific GC B cells. Prior to FCM analysis, cells were incubated with 10 μg/mL Cy7 tagged His-HA for 30 min as a probe to identify HA-specific B cells. B cells that bound to the probe were identified as HA specific GC B cells (**C, right panel**). **(D)** FMO of HA-specific MBCs. single-cell suspensions were incubated with 5μg/mL His-HA-Biotin for 30 min. After washing 3 times, cells were incubated with PE-Streptavidin for another 30 min as a probe to identify HA-specific switched MBCs. B cells that bound to the probe were identified as HA specific MBCs (**D, right panel**). HA^{+} cells were negligible when the His-HA-Cy7 or His-HA-Biotin probe was not added prior to staining (**left panel of C and D**).

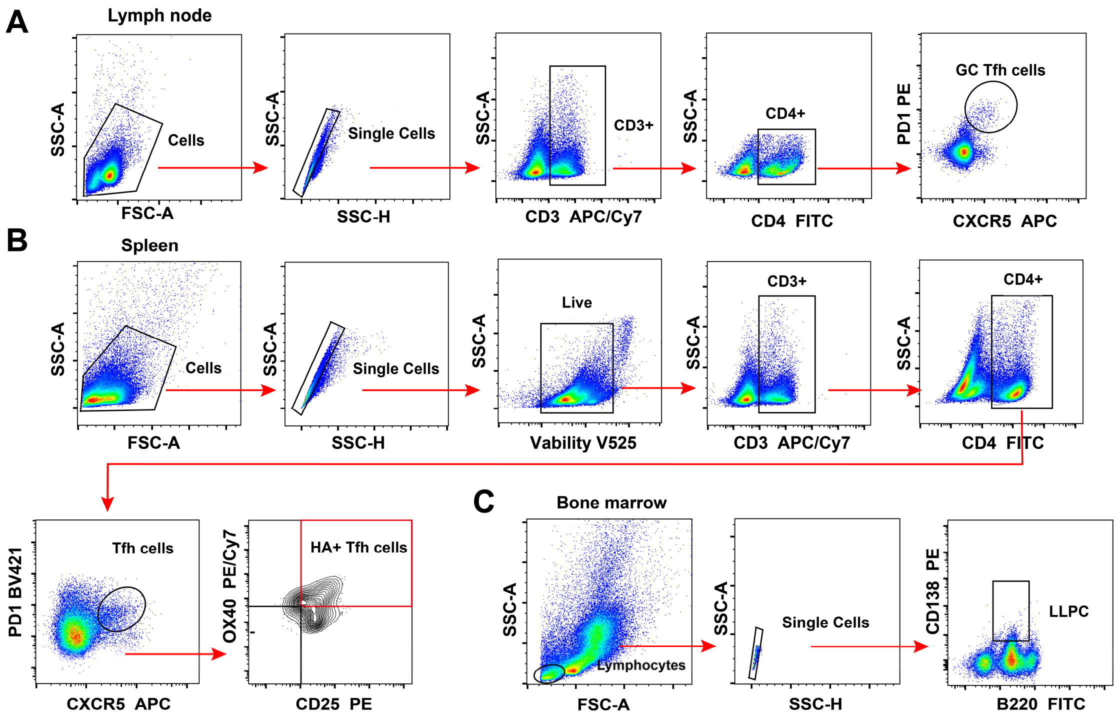

Appendix Figure S14 FCM gating strategy to identify GC Tfh cells and LLPC. Cells were first gated to exclude debris and dead cells. (**A**) Gating strategy to identify GC Tfh cells ($CD4^{+}CXCR5^{+}PD1^{+}$). (**B**) HA specific GC Tfh cells was identified by the expression of surface markers CD25 and OX40 in spleen. (**C**) Gating strategy to identify LLPCs ($B220^{lo}CD138^{+}$) in bone marrow.

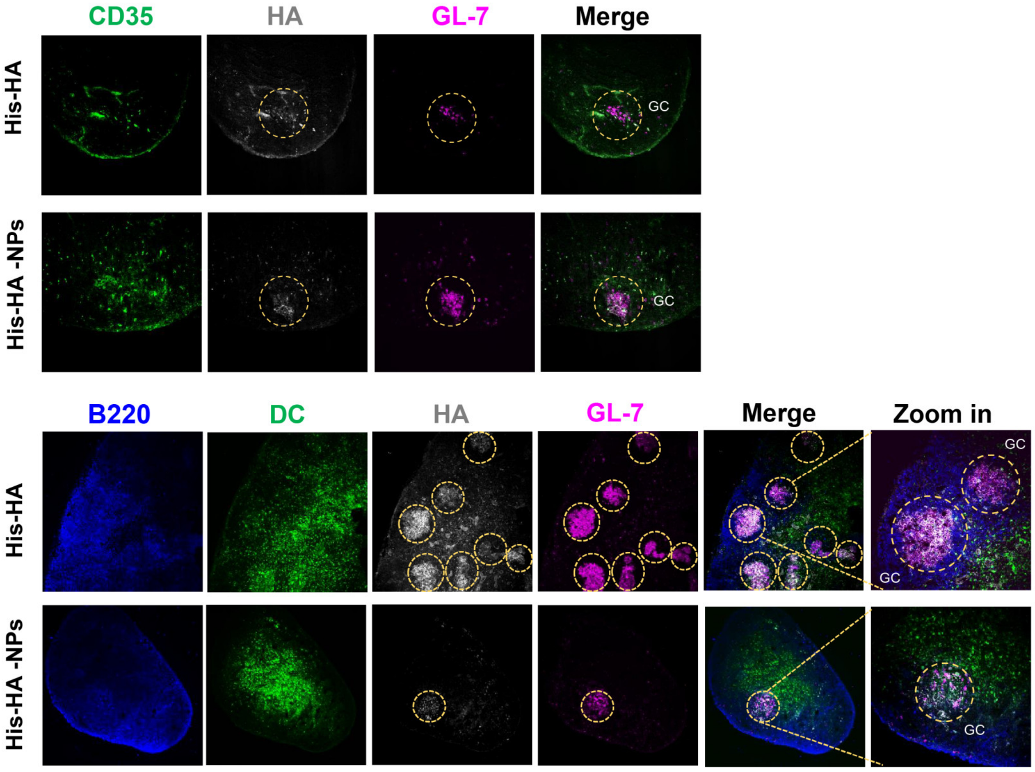

Appendix Figure S15. HA@PPCDQ facilitates cross-linking between antigen and GC B cells. (**A**) C57BL/6 mice were immunized with equal mass of iFlour^{TM} 594-tagged His-HA or His-HA-NPs. ILNs were collected at 4 h after boost immunization. Cryosections of iLNs were incubated with BV510 anti-mouse CD35 and Alexa Flour 647 anti-mouse GL-7. Scalar bar: 100 μm. (**B**)Transgenic mice CD11c-EYFP were immunized with equal mass of iFlour^{TM} 594-tagged His-HA or His-HA-NPs. Cryosections of iLNs were collected at 4 h after second vaccination and incubated with Brilliant Violet 421™ anti-mouse/human CD45R/B220 and Alexa Flour 647 anti-mouse GL-7. Scalar bar: 100 μm (left), 50 μm (right).

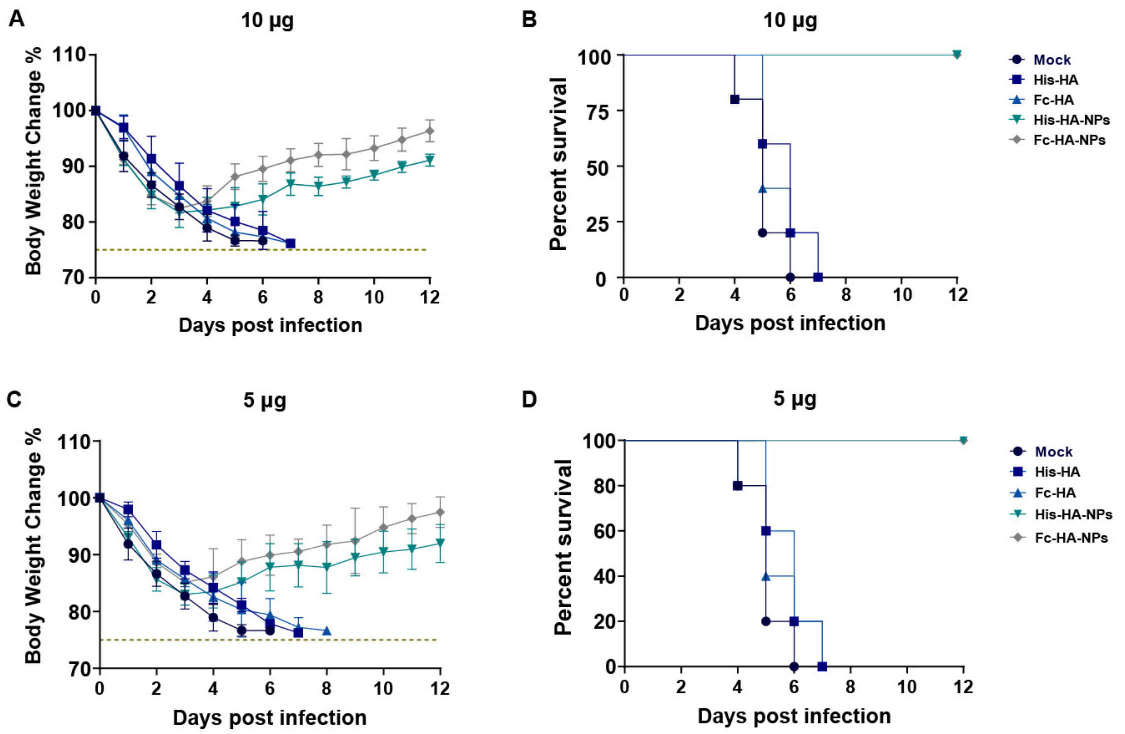

Appendix Figure S16. HA@PPCDQ protects mice from lethal IAV virus challenge. (**A-D**) Mice within each group were prime/boost-vaccinated with different vaccines (5 µg and 10 µg dose) at week 0 and week 2. Four weeks after booster immunization, mice were challenged with authentic IAV. Mean weight loss (**A and C**) and survival rates (**B and D**) were monitored for 12 dpi (n = 5 animals per group). Mice weighing <75% of their pre-challenge weight were euthanized.

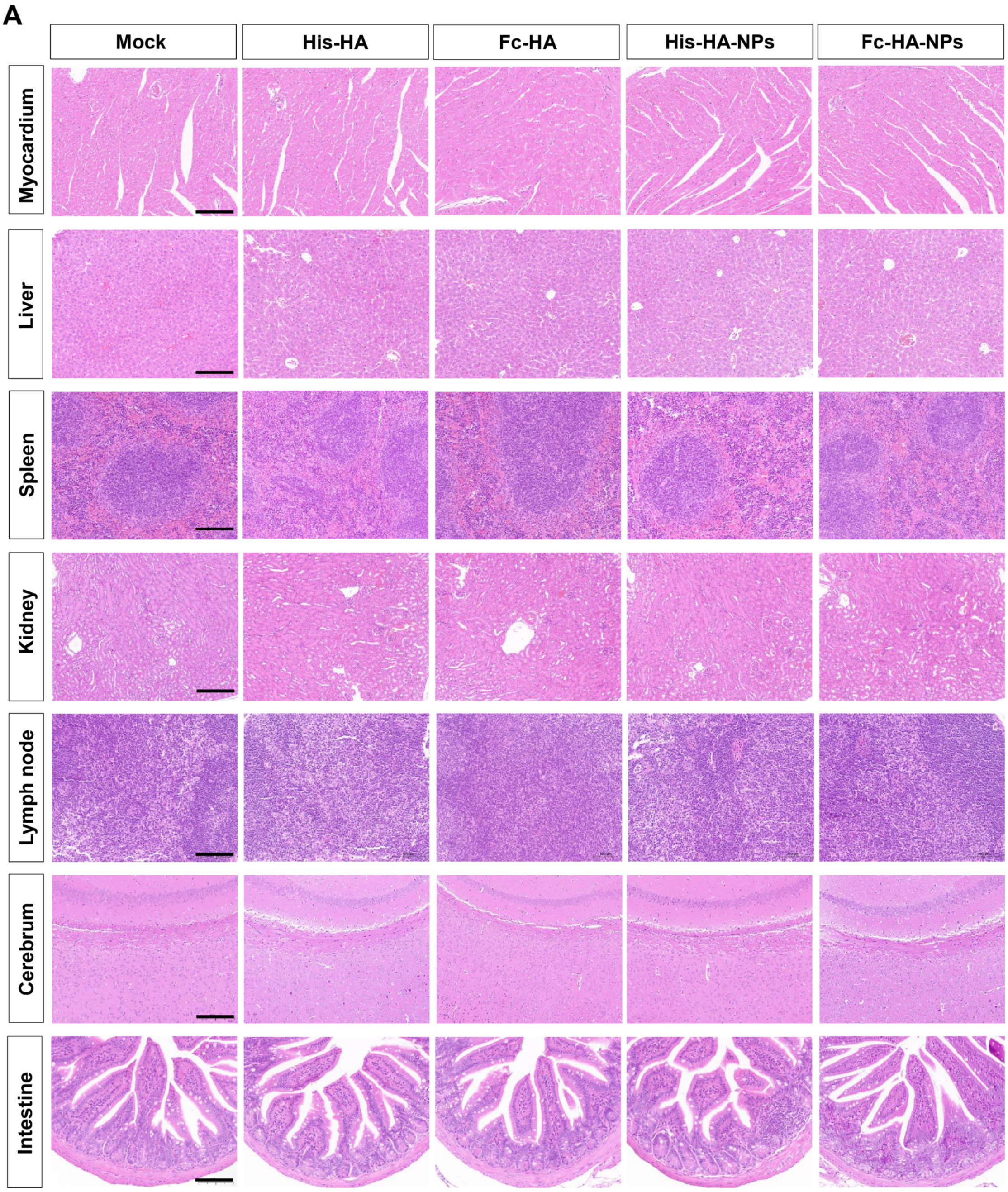

Appendix Figure S17. HA@PPCDQ is bio-safe in mice.

(**A**) Histopathological analysis of different tissues of vaccinated mice. Tissues contained myocardium, liver, spleen, kidney, cerebrum, intestine, and lymph node. Scale bars, 100 μm.

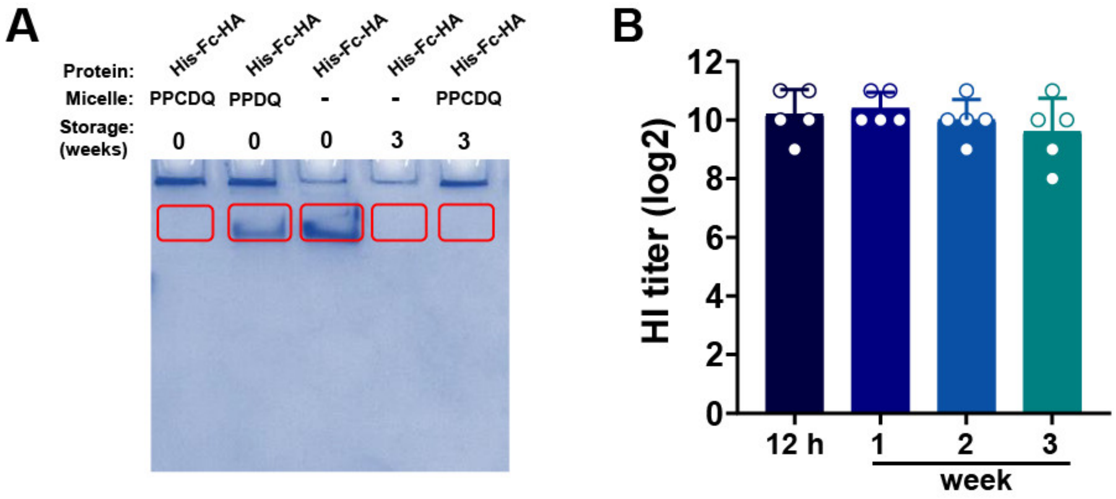

Appendix Figure S18. Stability of PPCDQ micelle with pre-bound antigen.

(**A**) Native PAGE was used to check the stability of pre-bound samples after 3-weeks of storage at 4 °C. (**B**) Mice were immunized with Fc-HA-NPs after different storage periods. Data are presented as mean ± SD, n = 5 animals per group.

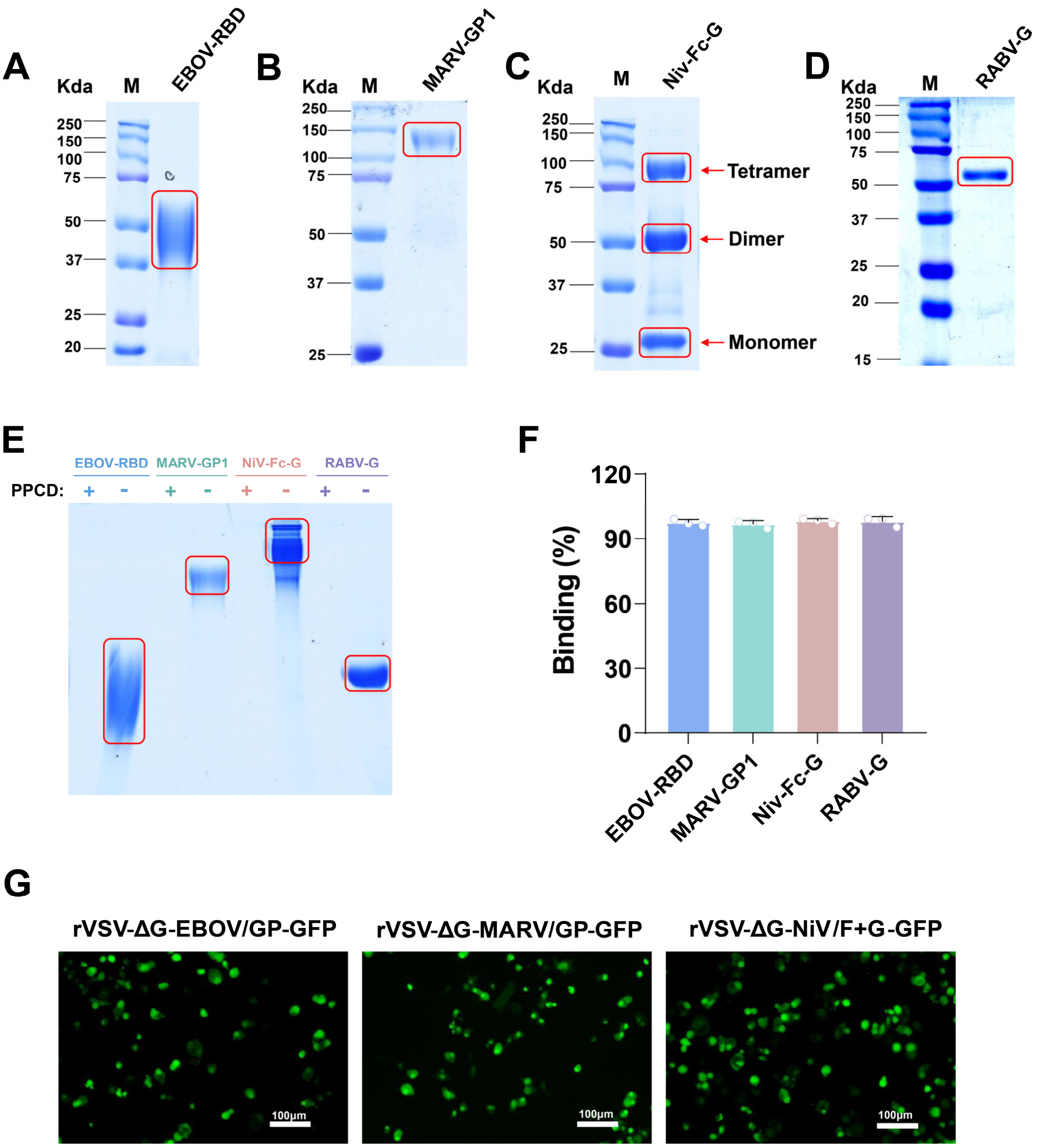

Appendix Figure S19. SDS–PAGE gels of various proteins and fluorescence images of pesodoviruses.

(**A-D**) SDS–PAGE gel of EBOV-RBD (**A**), MARV-GP1 (**B**), NiV-Fc-G (**C**), and RABV-G (**D**). (**E-F**) Native PAGE gel (**E**) and statistical graph of binding efficiency (**F**) of PPCD micelles to EBOV-RBD, MARV-GP1, NiV-Fc-G, and RABV-G. Data are presented as mean ± SD, n = 3 biological replicates. (**G**) Fluorescence images of different recombinant vesicular stomatitis viruses (rVSVs), scalar bar:100 μm.

Appendix Table S1. Primers used for plasmid construction.

Primer	Sequences (5'-3')
**C-His-HA-F**	GGCGTGCAGAGTGACACCATCTGCATCGGGTACC
**C-His-HA-R**	CGCCGCCTCCCAGCTTCACTCCGTCCACCTTCTC
**C-Fc-HA-F**	TCTCATCATTTTGGCAAAGAATTCGCCACCATGGACTGGACCT
**C-Fc-HA-R**	GTACATATGCAAGGCTTACAACCCTCGAGACTTCCTCCTCCTC
**C-His-Fc-HA-F**	ATCATTTTGGCAAAGAATTCGCCACCATGGACTGGA
**C-His-Fc-HA-R**	ACTTCCTCCTCCTCCGCTGCCGCCGCCTCCTTTACCAGGAGAGTGGGAG
**N-His-Fc-HA-F**	ATCACCATCACCATCACCATCACGGAAGCGGAAGCGACACCATCTGCATC
**N-His-Fc-HA-R**	CCGCTGCCGCCGCCTCCCAGCTTCACTCCGTCCAC
**NiV-Fc-G-F**	CAGCGGAGGAGGAGGAAGTCAAAACTACACAAGATCAAC
**NiV-Fc-G-R**	AAAAGATCTGCTAGCTCGAGTTATGTACATTGCTCTGGTATCTT
**EBOV-RBD-F**	GTGGCCGCCGCCACAGGCGTGCAGAGTATCCCACTTGGAGTCATCCAC
**EBOV-RBD-R**	GATGACTTCCTCCTCCTCCGCTGCCGCCGCCTCCGAAAGACAACTCTTCA
**MARV-GP1-F**	TCTCATCATTTTGGCAAAGAATTCGCCACCATGAAGACAACCTGTCTGTT
**MARV-GP1-R**	TCCTCCTCCGCTGCCGCCGCCTCCTCTTTTCTTTCTGAAGTAGAC
