# Supplementary figures and images for: A broadly applicable protein-polymer adjuvant system for antiviral vaccines (part 1 of 2)
Source: EMBO Mol Med. 2024 May 15;16(6):1451–83. doi: 10.1038/s44321-024-00076-4 (PMC11178928; doi:10.1038/s44321-024-00076-4)

## Slide 1
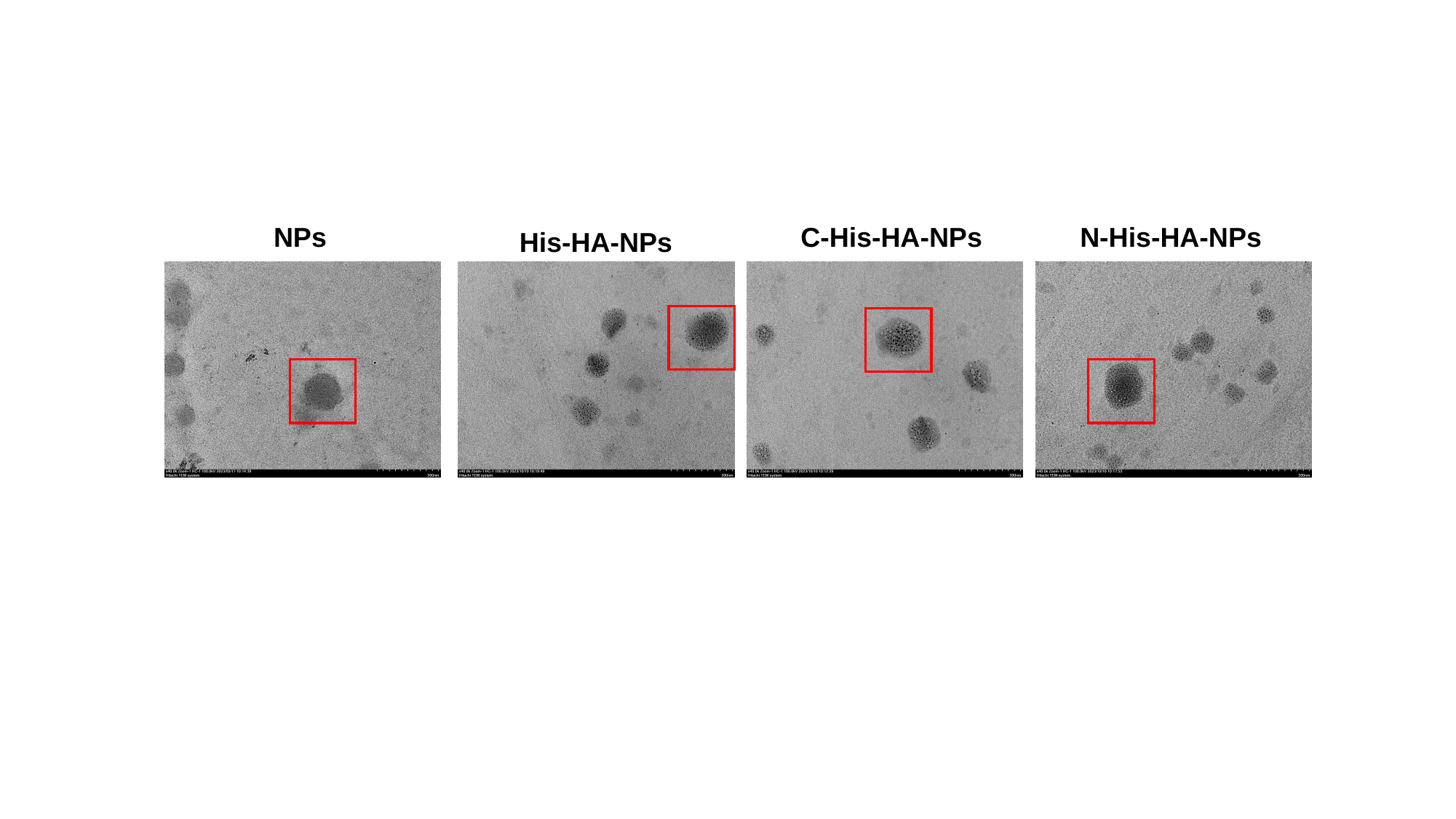

NPs
C-His-HA-NPs
N-His-HA-NPs
His-HA-NPs

Supplement: Supplementary file 4 — Source data Fig. 1 [file 44321_2024_76_MOESM4_ESM.zip › Figure 1C/Figure 1C.pptx]

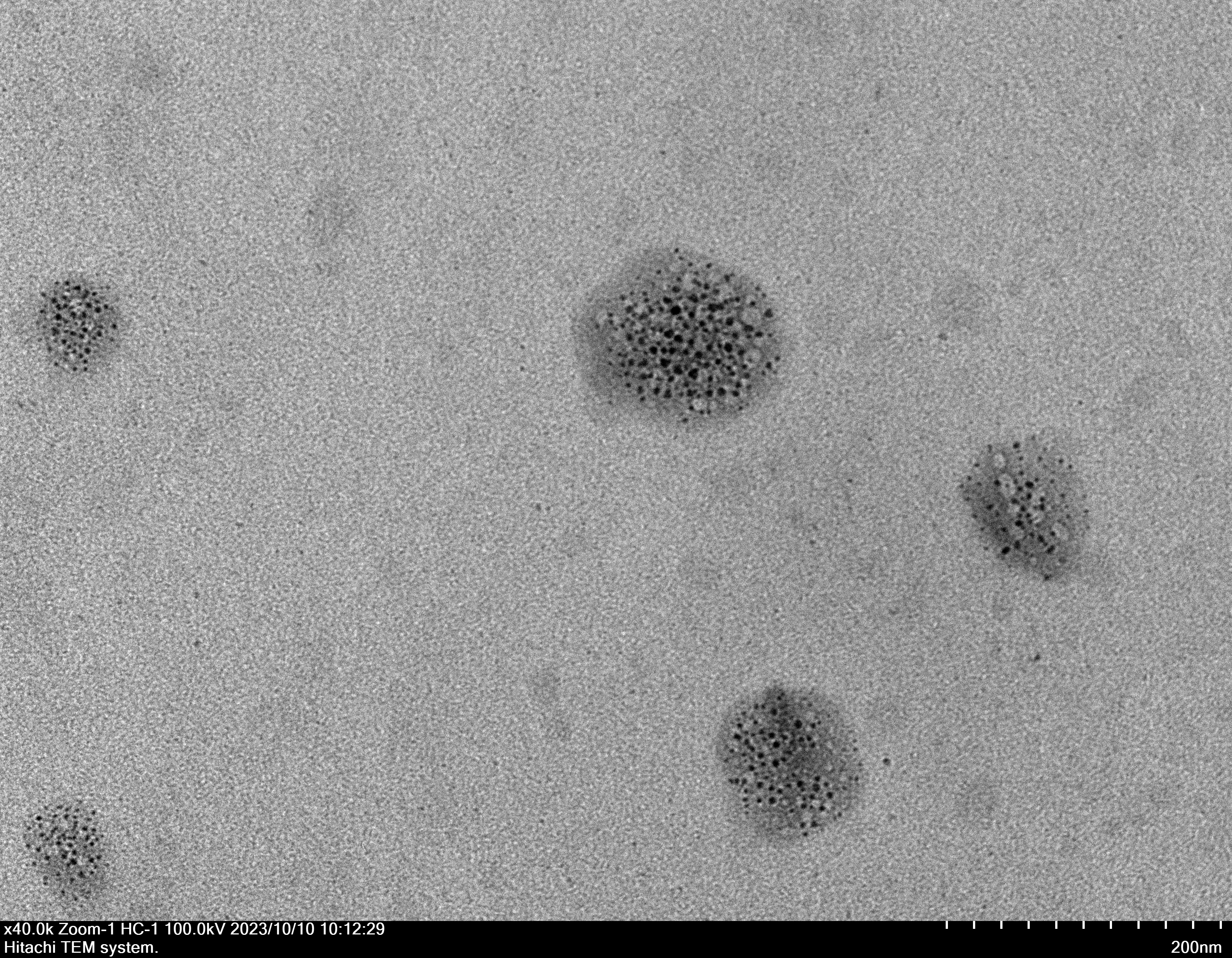

Supplement: Supplementary file 4 — Source data Fig. 1 [file 44321_2024_76_MOESM4_ESM.zip › Figure 1C/TEM/C-Fc-HA-NPs.tif]

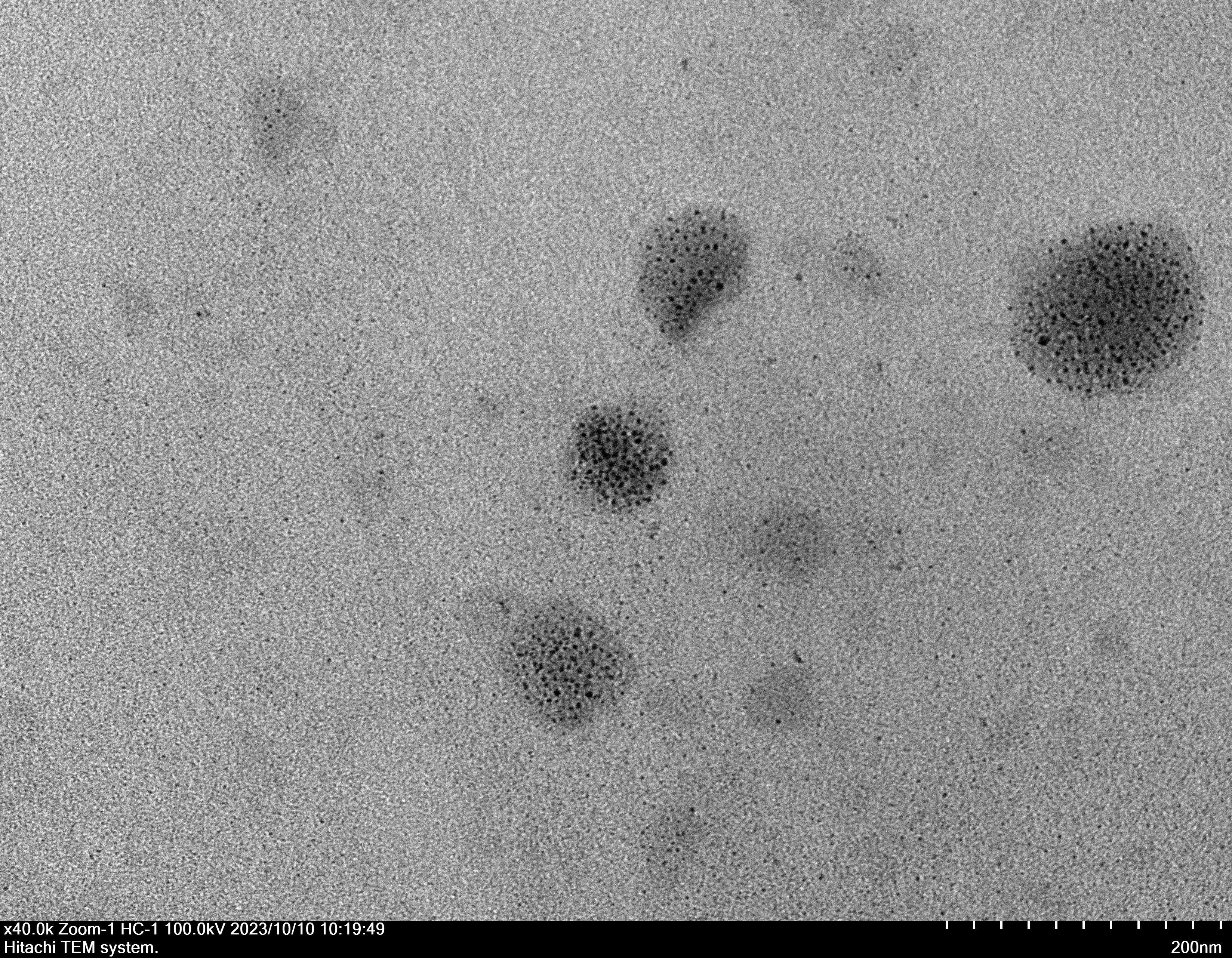

Supplement: Supplementary file 4 — Source data Fig. 1 [file 44321_2024_76_MOESM4_ESM.zip › Figure 1C/TEM/His-HA-NPs.tif]

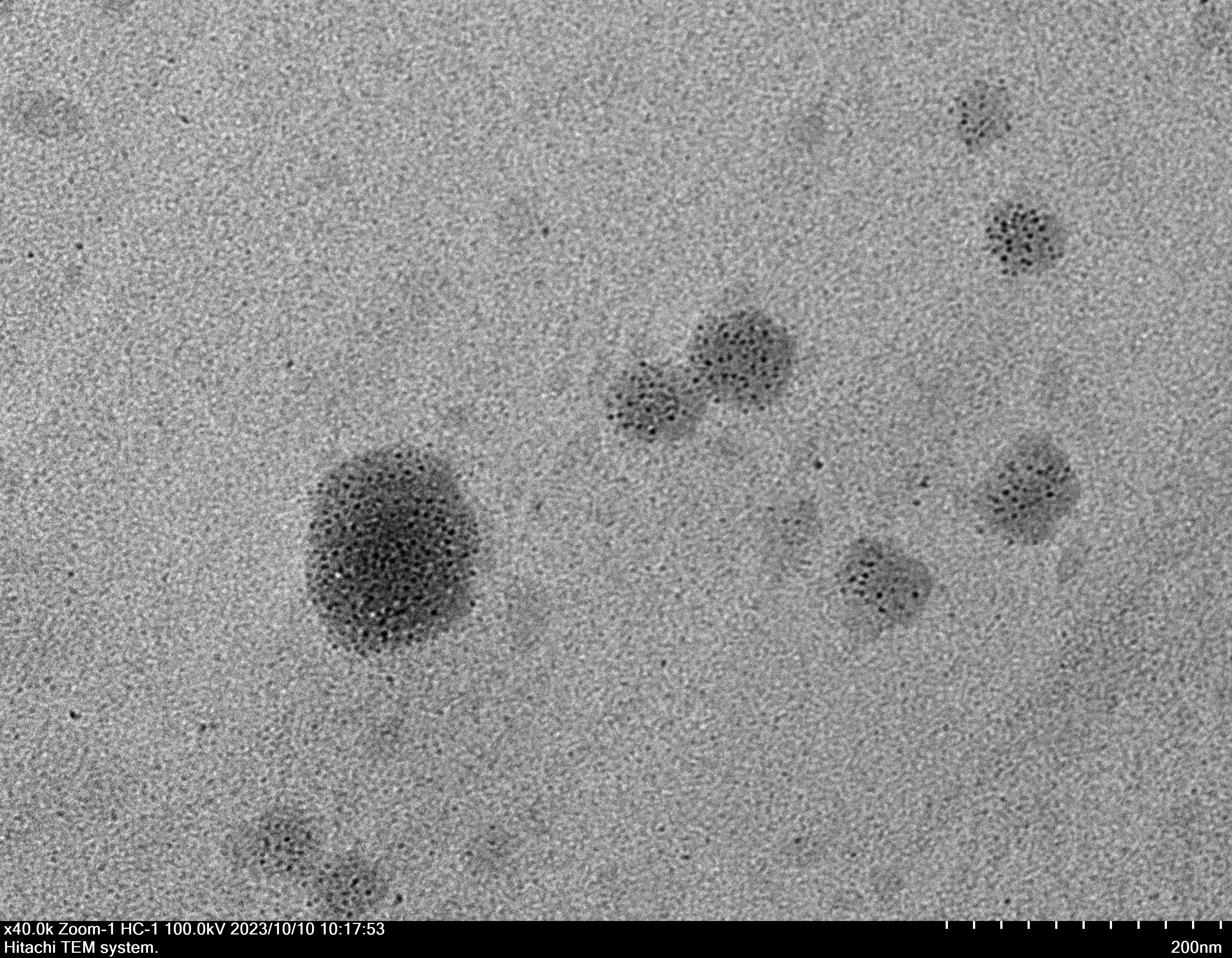

Supplement: Supplementary file 4 — Source data Fig. 1 [file 44321_2024_76_MOESM4_ESM.zip › Figure 1C/TEM/N-Fc-HA-NPs.tif]

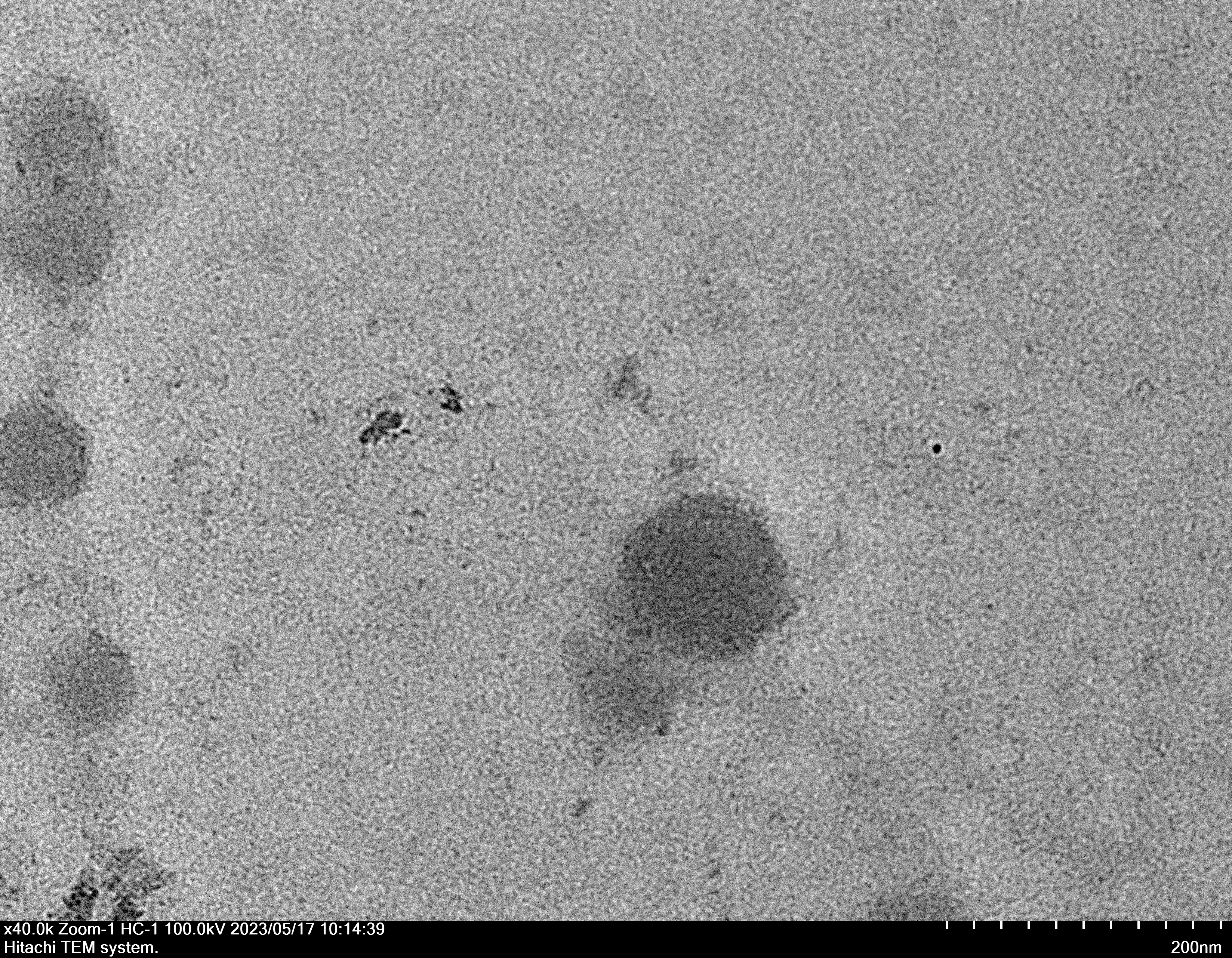

Supplement: Supplementary file 4 — Source data Fig. 1 [file 44321_2024_76_MOESM4_ESM.zip › Figure 1C/TEM/NPs.tif]

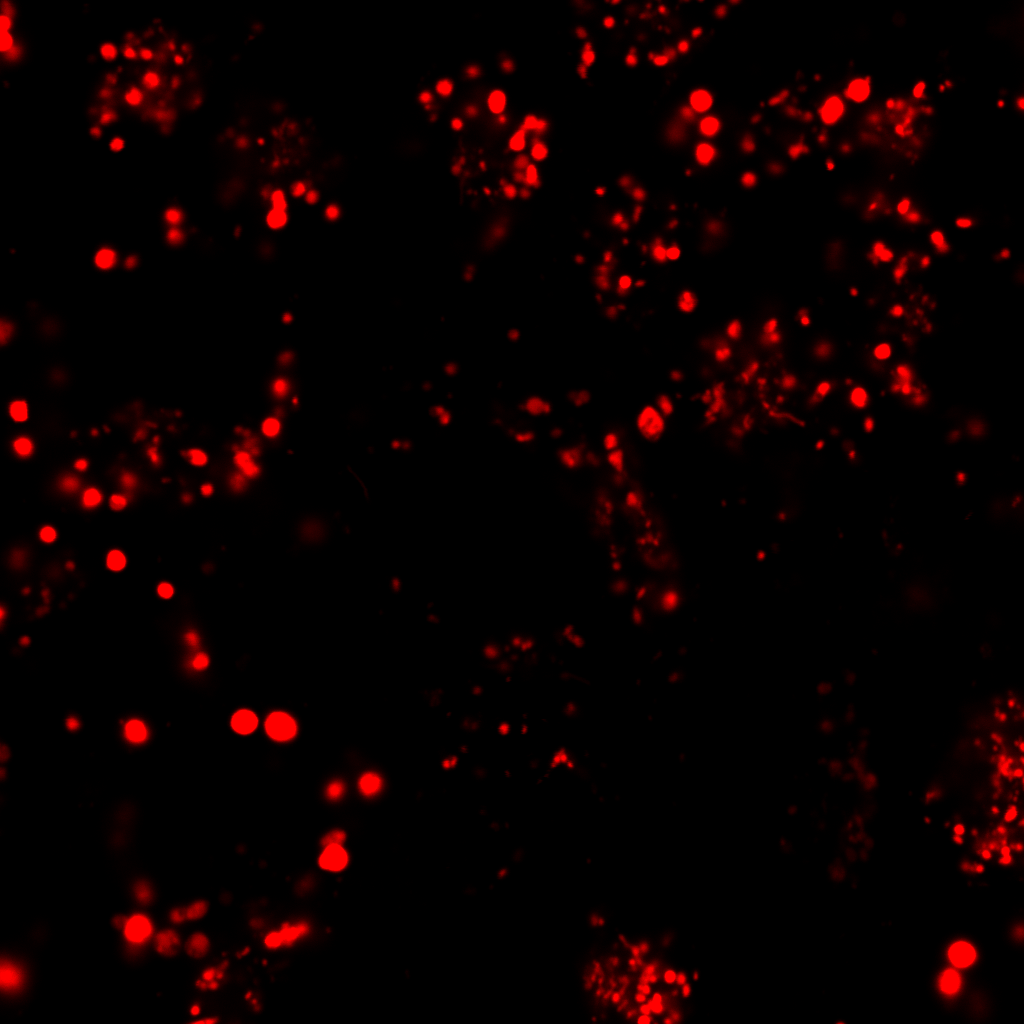

Supplement: Supplementary file 5 — Source data Fig. 2 [file 44321_2024_76_MOESM5_ESM.zip › Figure 2B/DC2.4/His-HA-NPs/Hemagglutinin.tif]

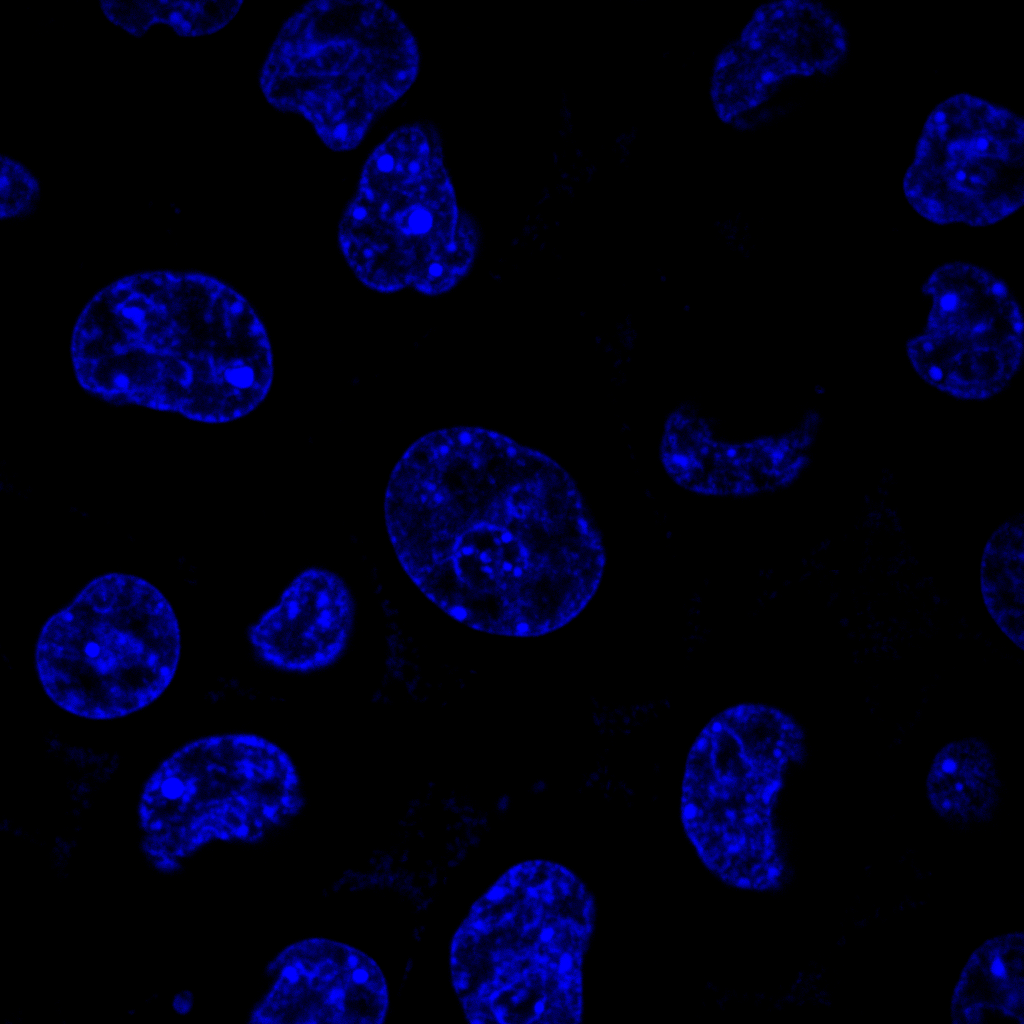

Supplement: Supplementary file 5 — Source data Fig. 2 [file 44321_2024_76_MOESM5_ESM.zip › Figure 2B/DC2.4/His-HA-NPs/Hoechst.tif]

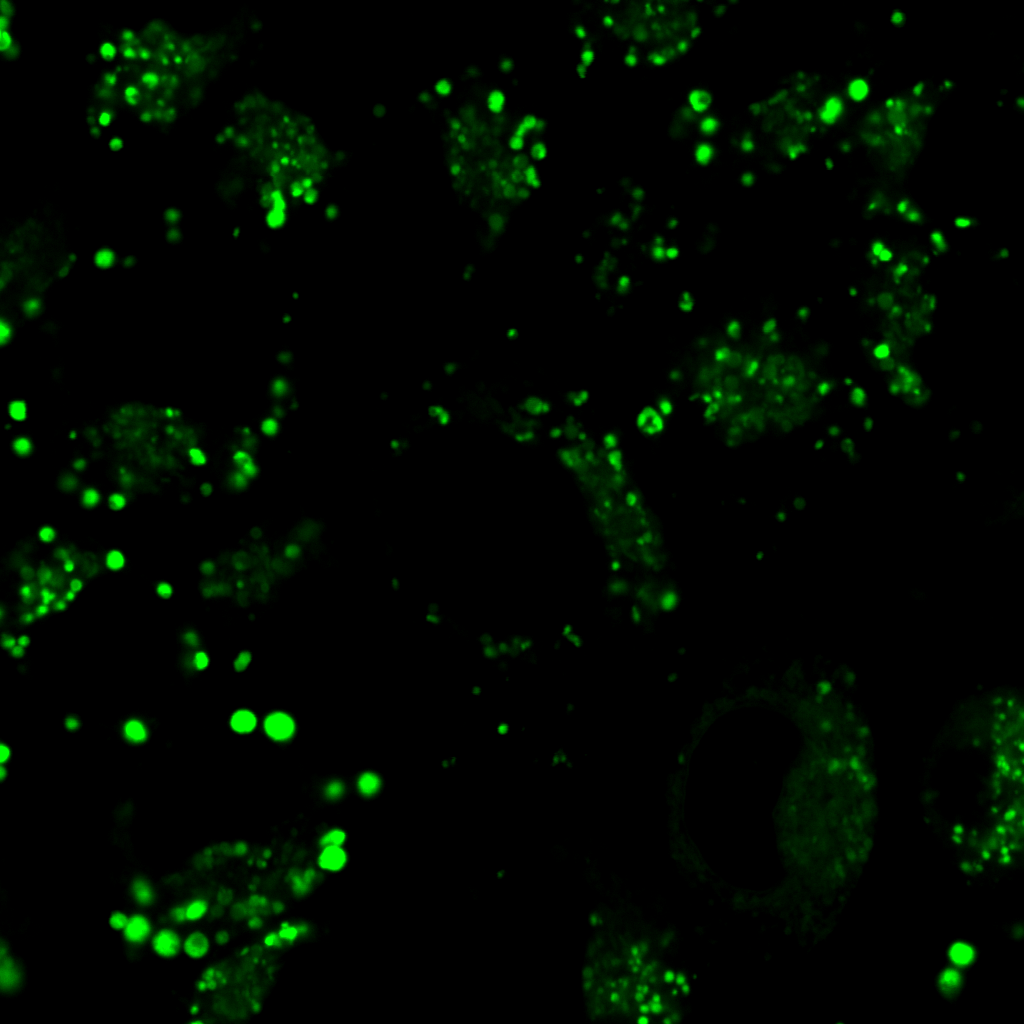

Supplement: Supplementary file 5 — Source data Fig. 2 [file 44321_2024_76_MOESM5_ESM.zip › Figure 2B/DC2.4/His-HA-NPs/Lysotracker.tif]

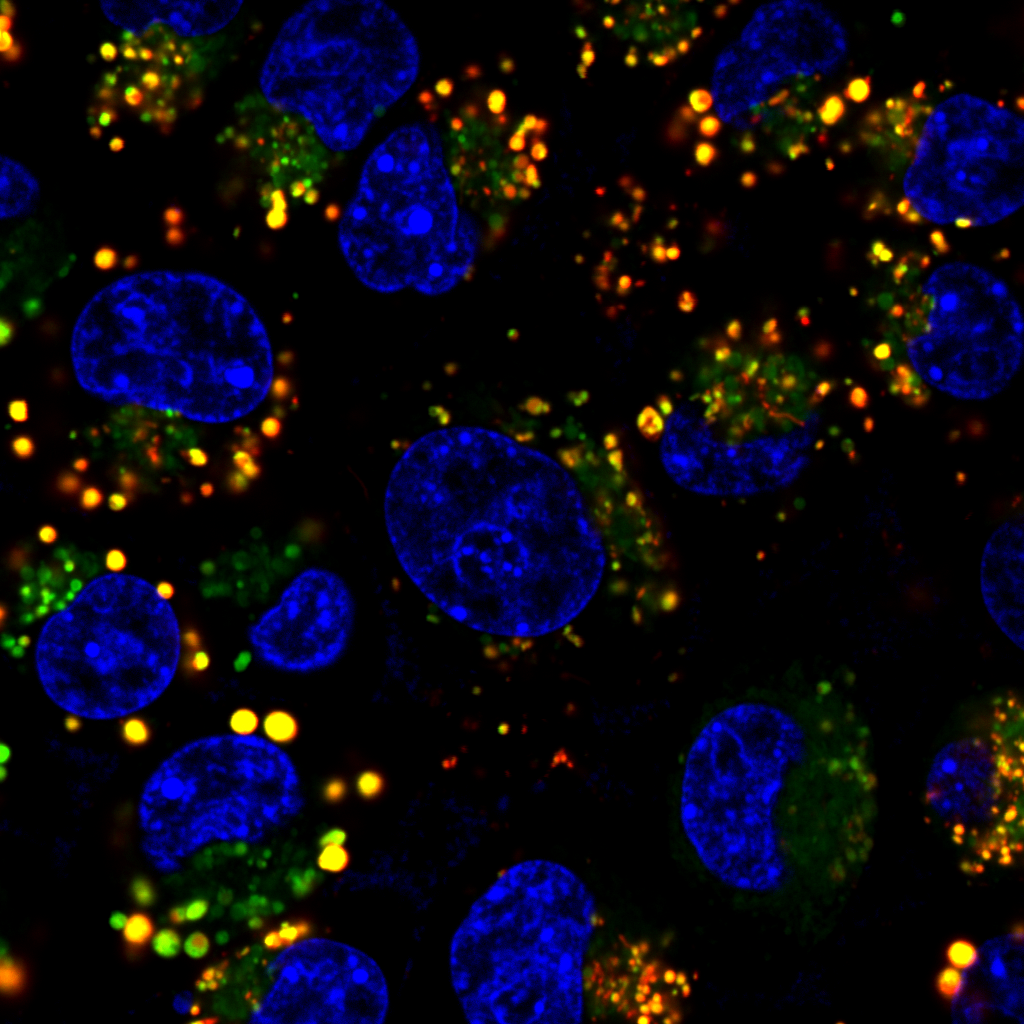

Supplement: Supplementary file 5 — Source data Fig. 2 [file 44321_2024_76_MOESM5_ESM.zip › Figure 2B/DC2.4/His-HA-NPs/Merge.tif]

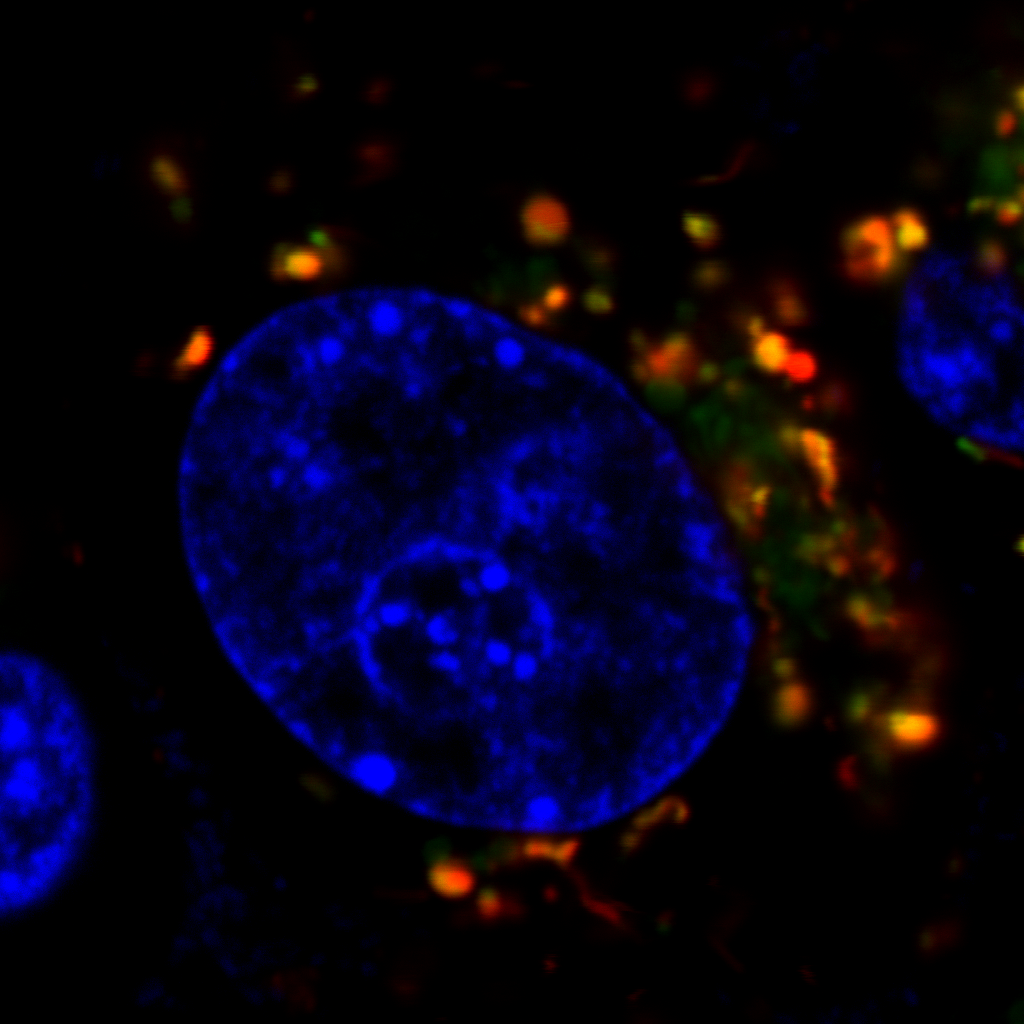

Supplement: Supplementary file 5 — Source data Fig. 2 [file 44321_2024_76_MOESM5_ESM.zip › Figure 2B/DC2.4/His-HA-NPs/Zoom.tif]

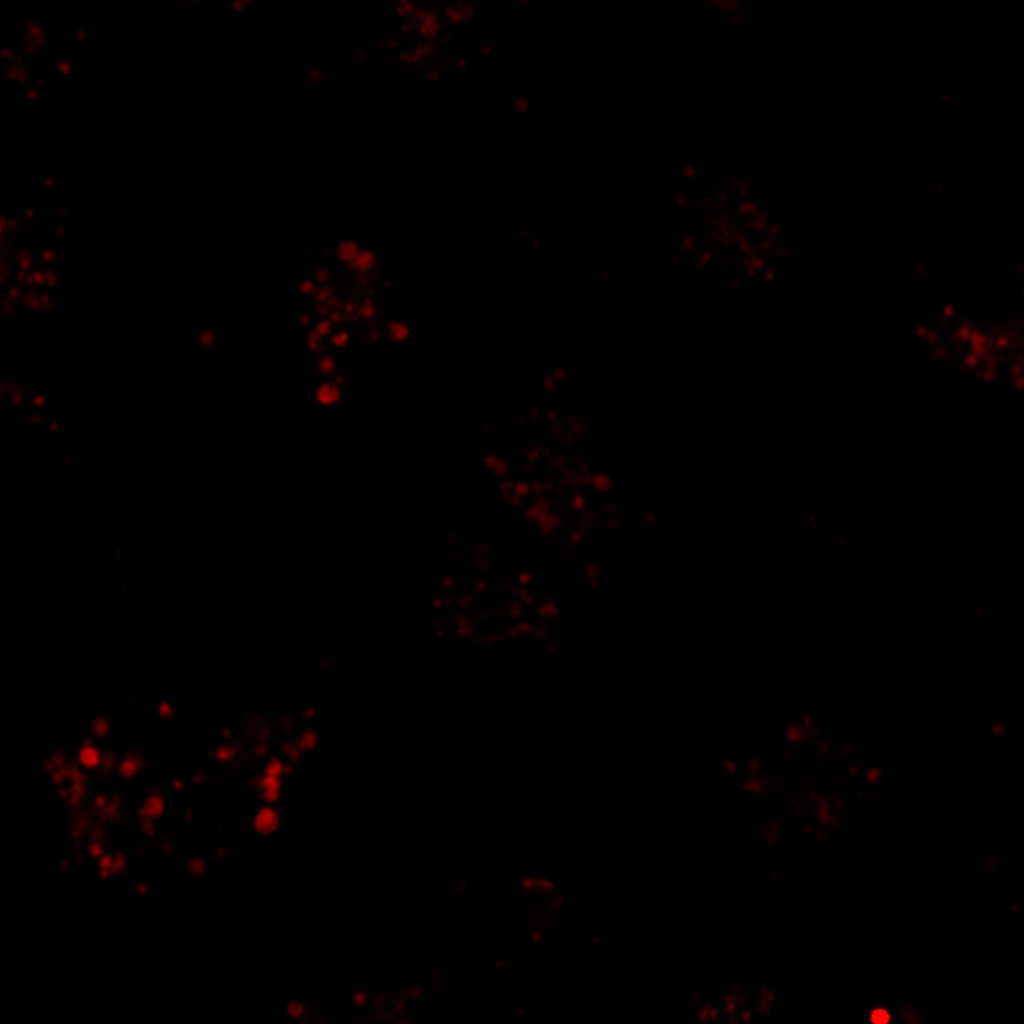

Supplement: Supplementary file 5 — Source data Fig. 2 [file 44321_2024_76_MOESM5_ESM.zip › Figure 2B/DC2.4/His-HA/Hemagglutinin.tif]

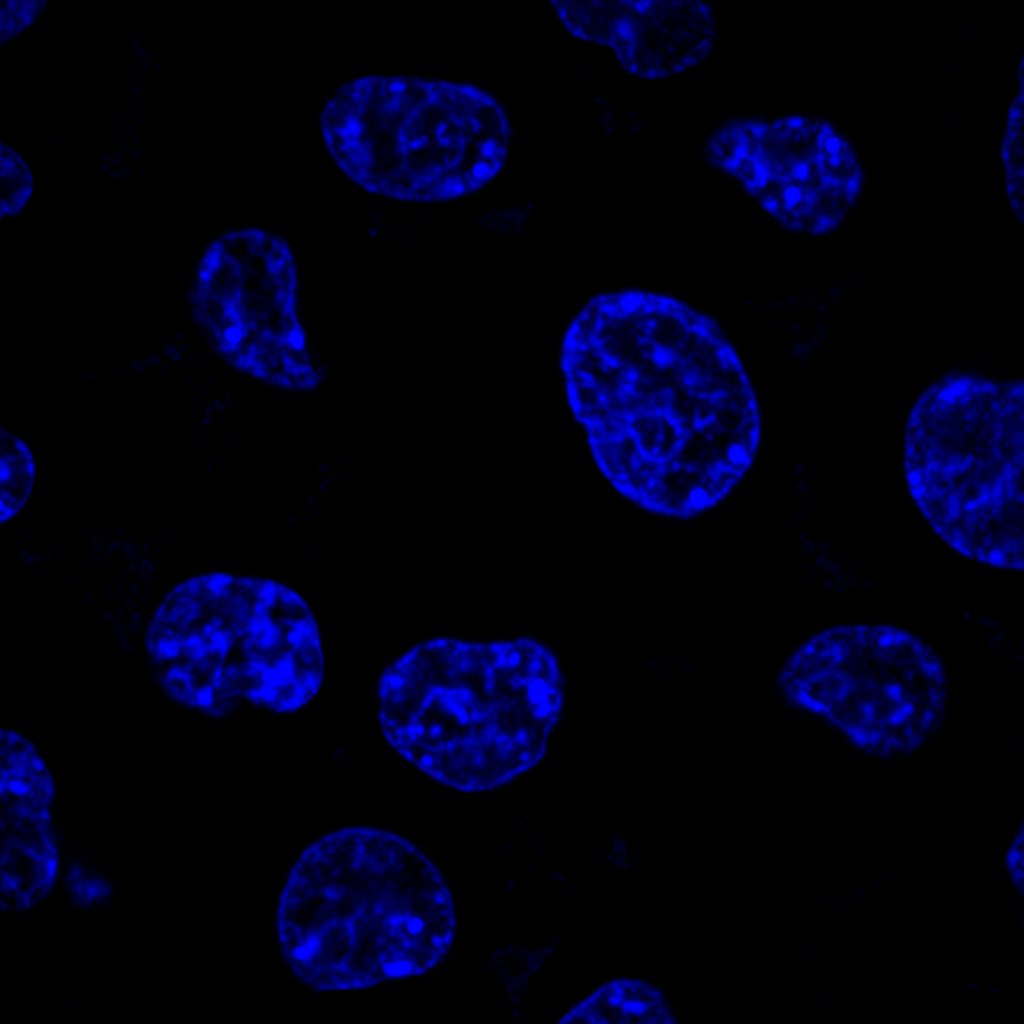

Supplement: Supplementary file 5 — Source data Fig. 2 [file 44321_2024_76_MOESM5_ESM.zip › Figure 2B/DC2.4/His-HA/Hoechst.tif]

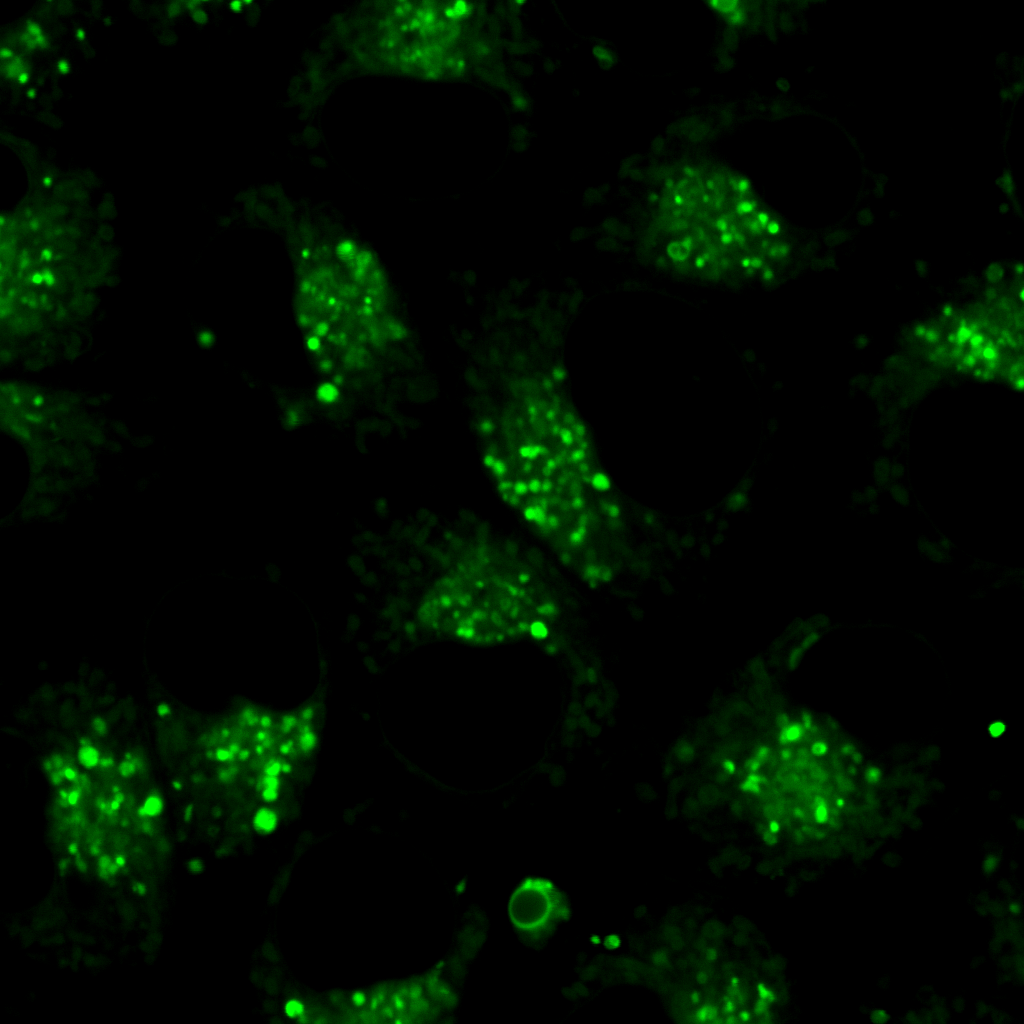

Supplement: Supplementary file 5 — Source data Fig. 2 [file 44321_2024_76_MOESM5_ESM.zip › Figure 2B/DC2.4/His-HA/Lysotracker.tif]

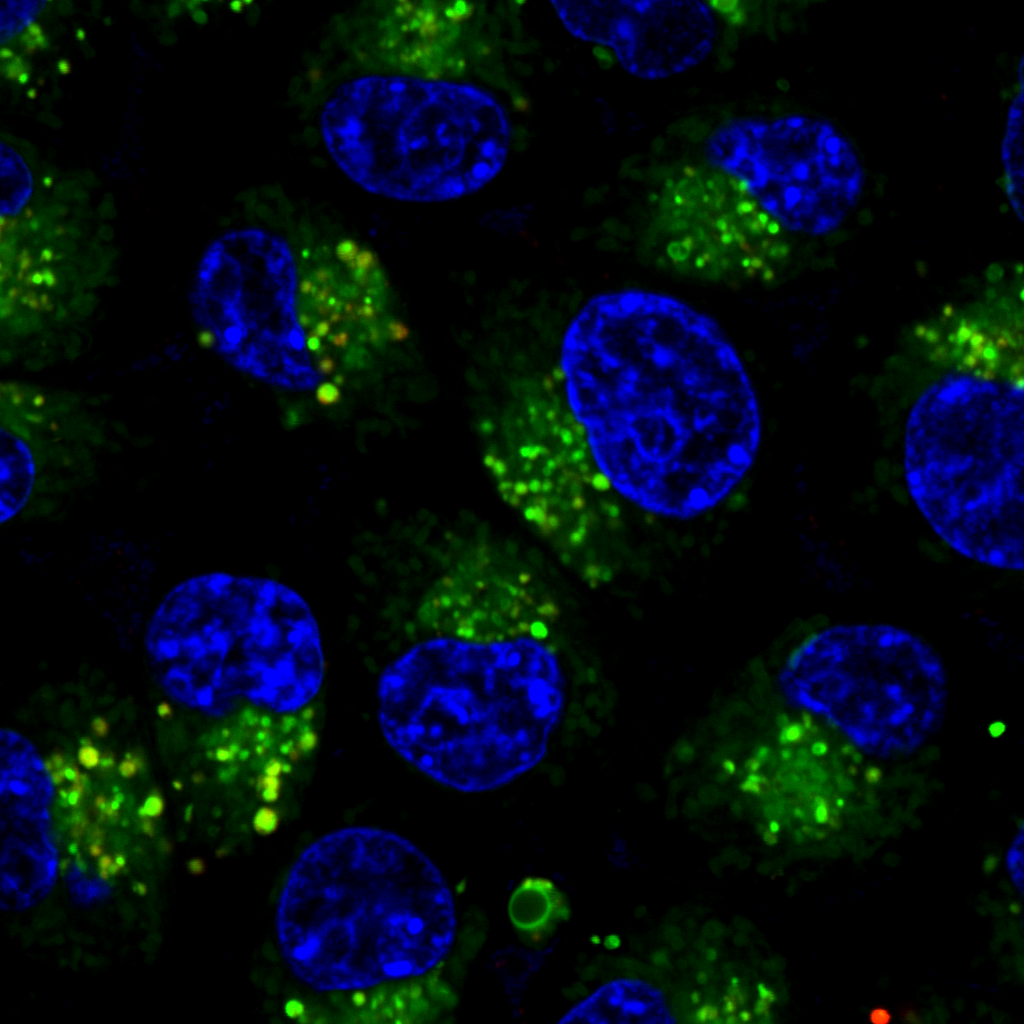

Supplement: Supplementary file 5 — Source data Fig. 2 [file 44321_2024_76_MOESM5_ESM.zip › Figure 2B/DC2.4/His-HA/Merge.tif]

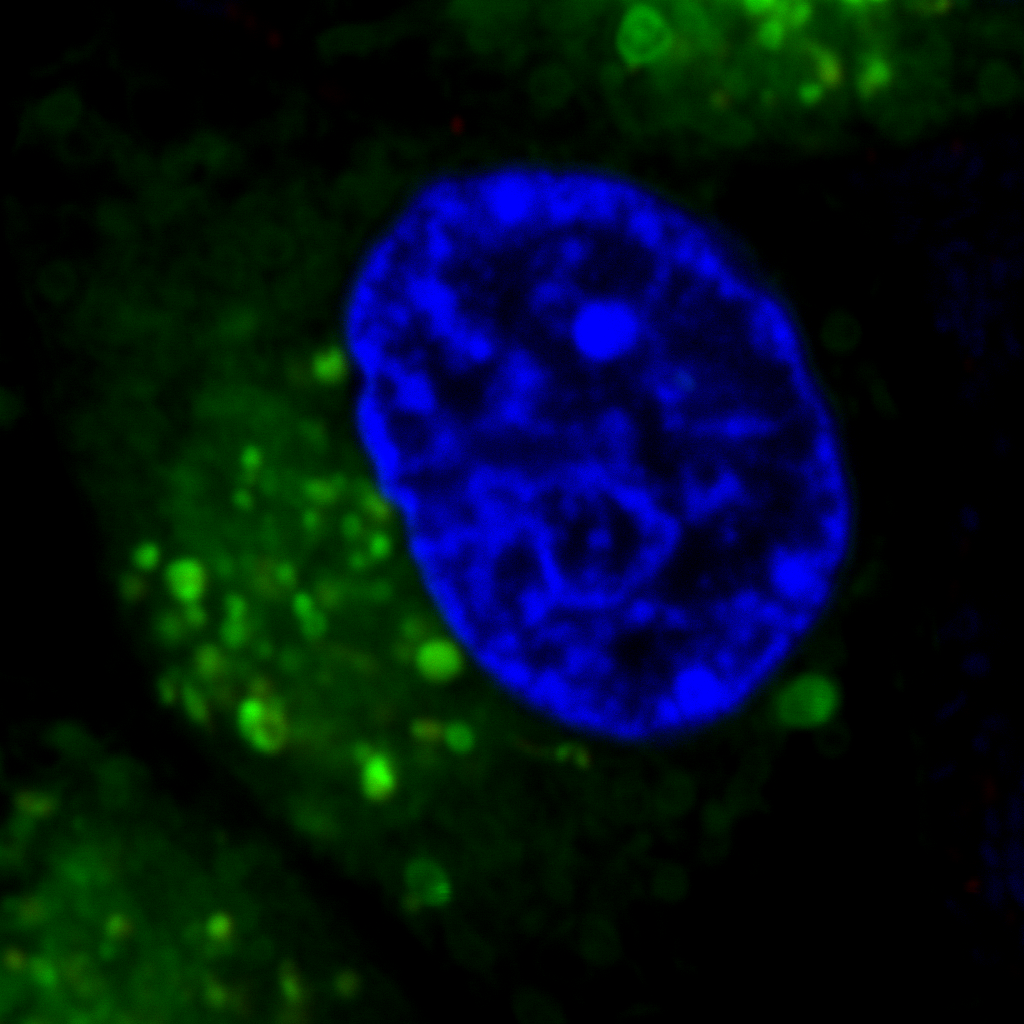

Supplement: Supplementary file 5 — Source data Fig. 2 [file 44321_2024_76_MOESM5_ESM.zip › Figure 2B/DC2.4/His-HA/Zoom.tif]

## Slide 1
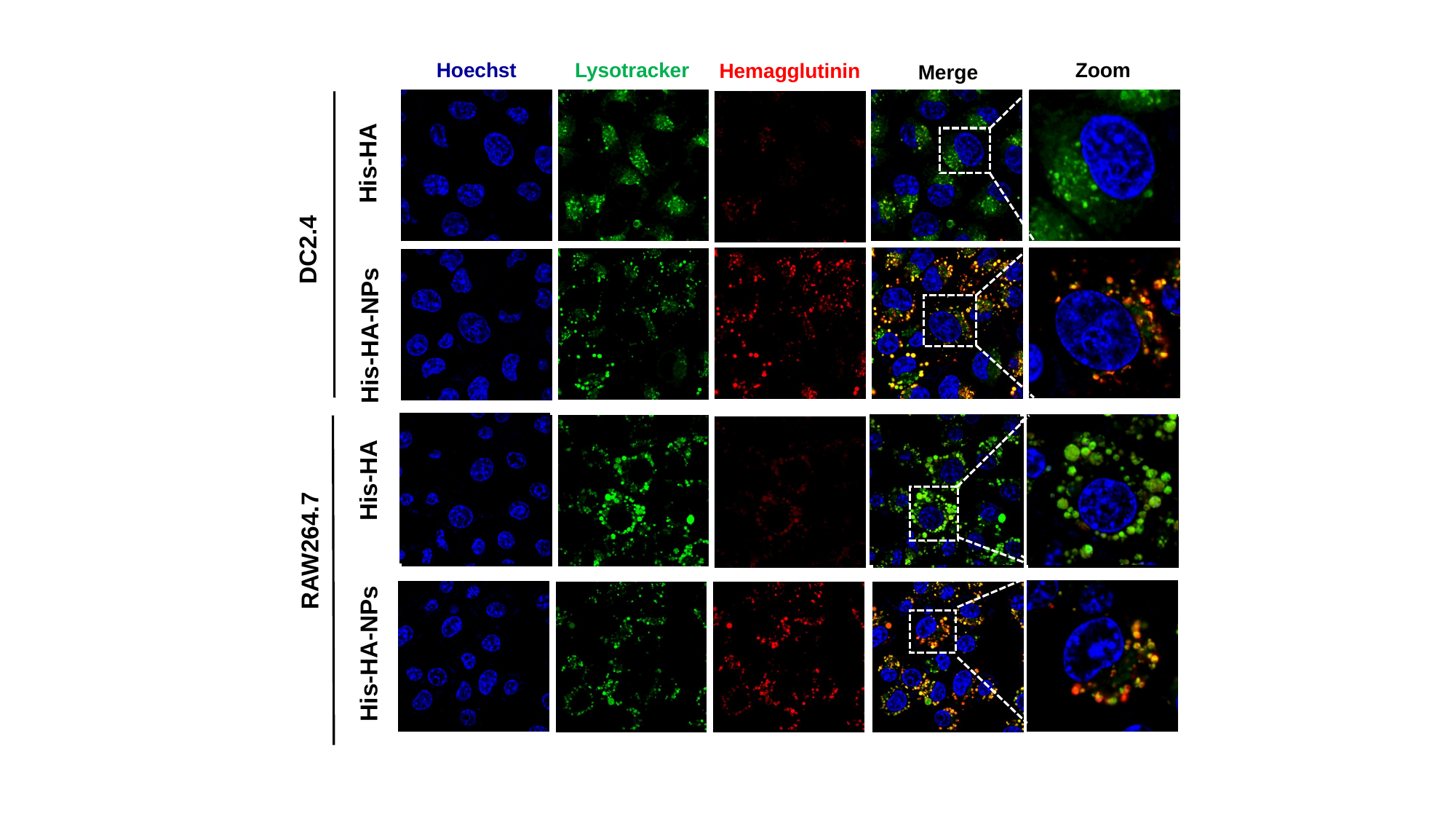

Lysotracker
Hoechst
Zoom
Hemagglutinin
Merge
His-HA
DC2.4
His-HA-NPs
His-HA
RAW264.7
His-HA-NPs

Supplement: Supplementary file 5 — Source data Fig. 2 [file 44321_2024_76_MOESM5_ESM.zip › Figure 2B/Figure 2B.pptx]

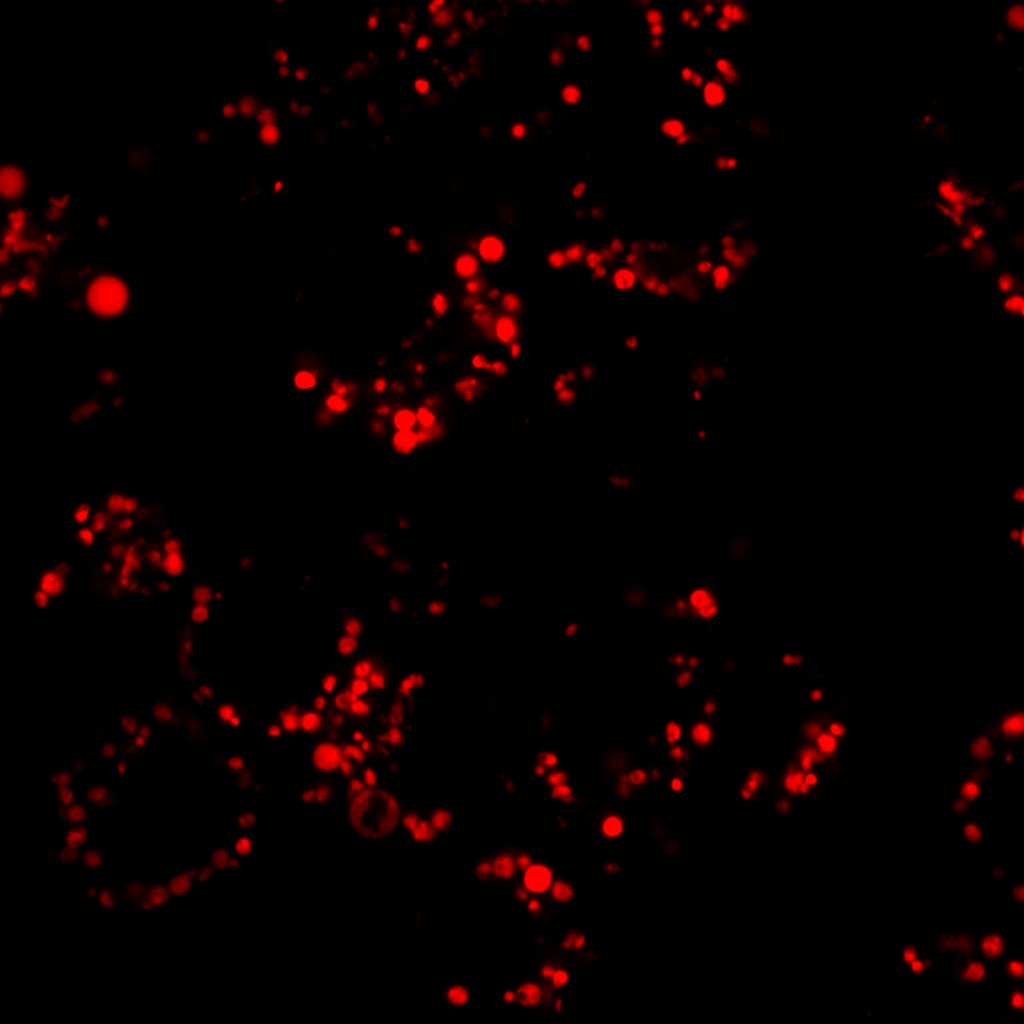

Supplement: Supplementary file 5 — Source data Fig. 2 [file 44321_2024_76_MOESM5_ESM.zip › Figure 2B/RAW264.7/His-HA-NPs/Hemagglutinin.tif]

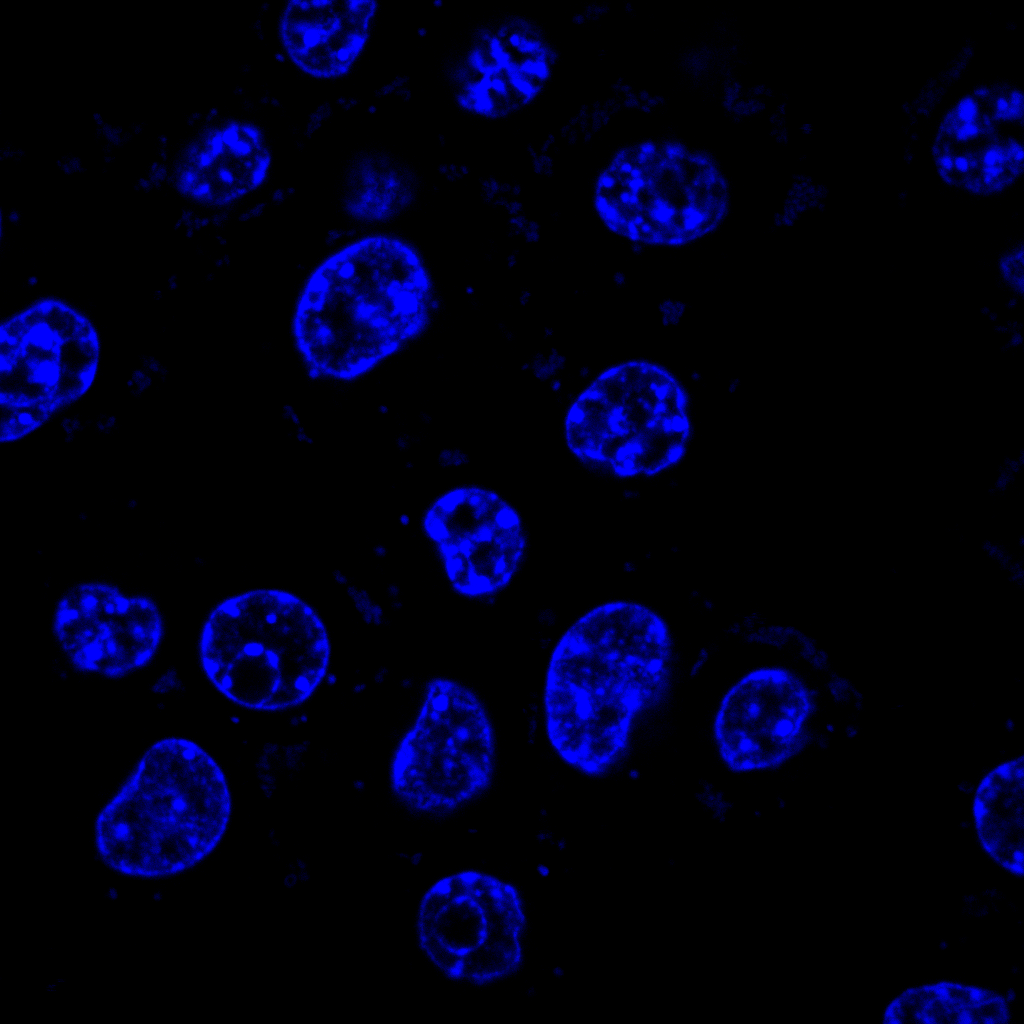

Supplement: Supplementary file 5 — Source data Fig. 2 [file 44321_2024_76_MOESM5_ESM.zip › Figure 2B/RAW264.7/His-HA-NPs/Hoechst.tif]

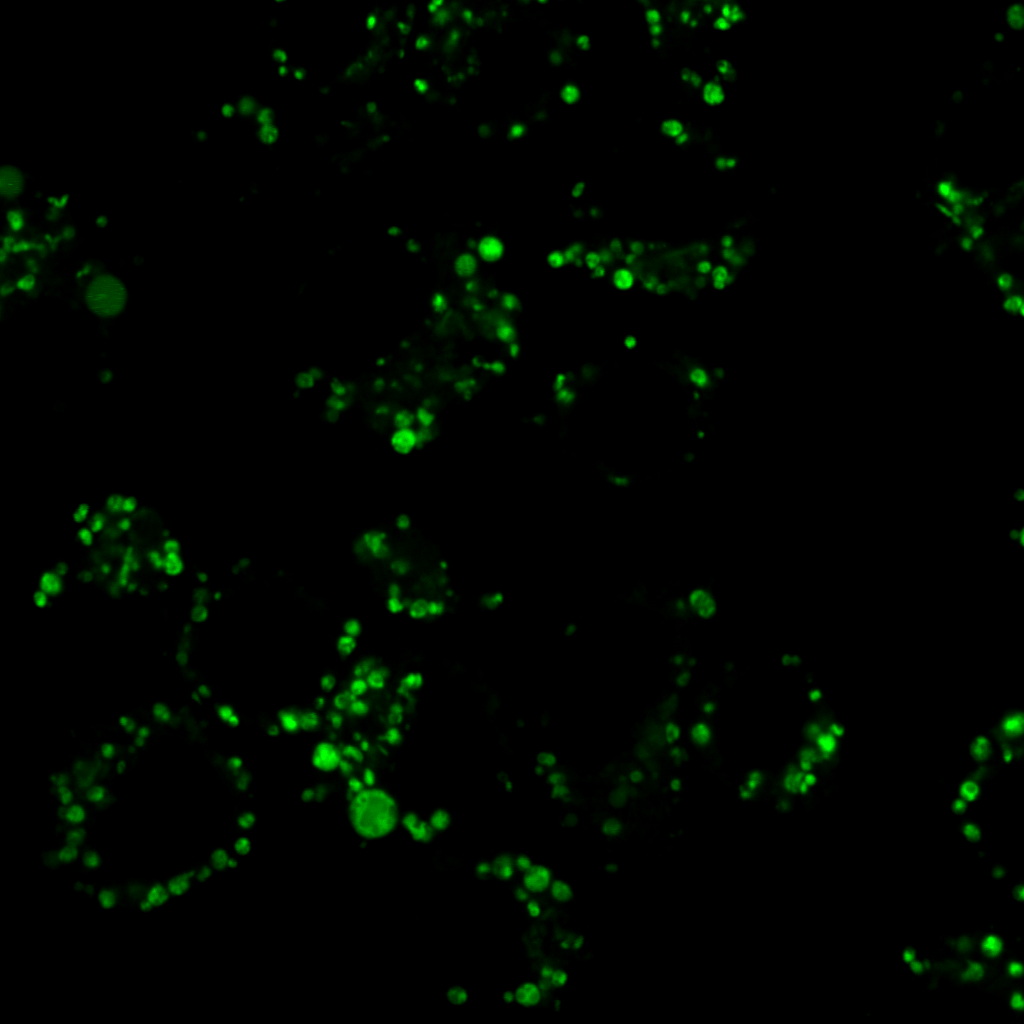

Supplement: Supplementary file 5 — Source data Fig. 2 [file 44321_2024_76_MOESM5_ESM.zip › Figure 2B/RAW264.7/His-HA-NPs/Lysotracker.tif]

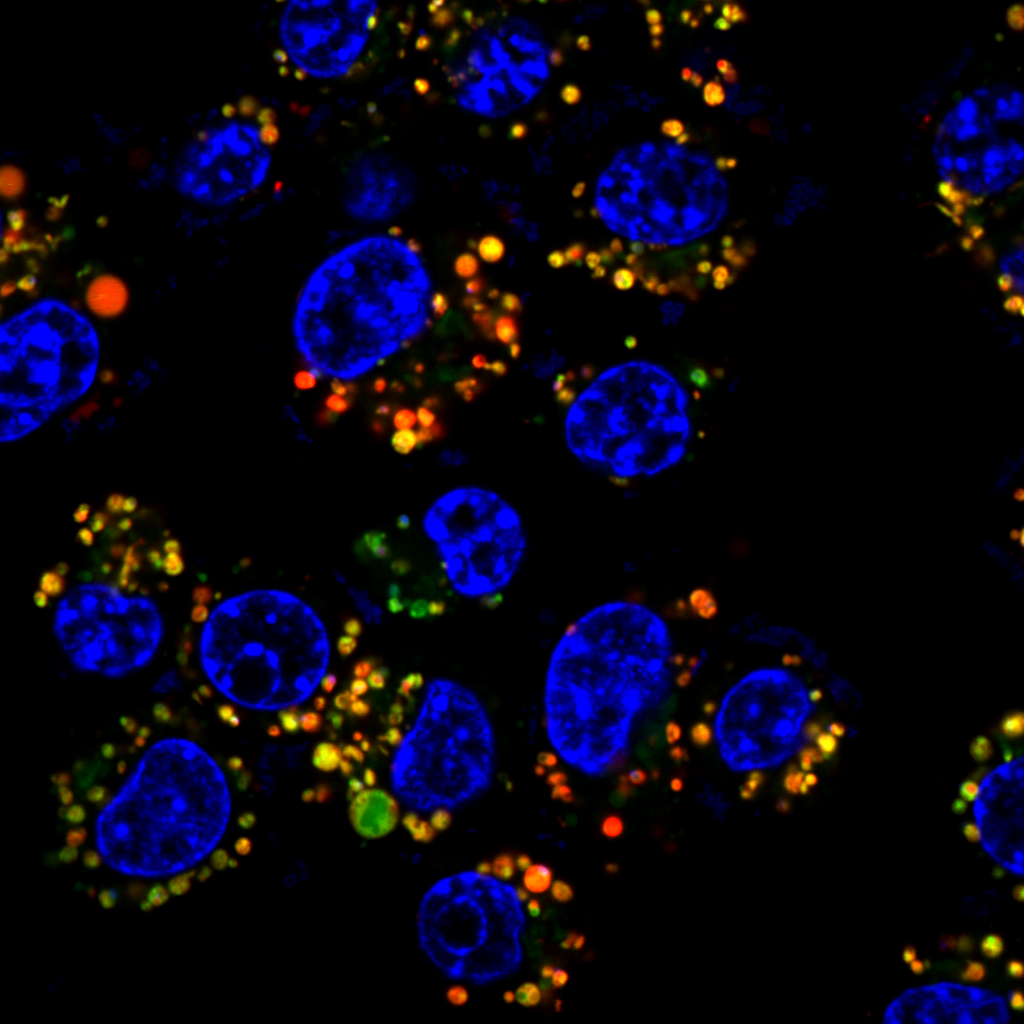

Supplement: Supplementary file 5 — Source data Fig. 2 [file 44321_2024_76_MOESM5_ESM.zip › Figure 2B/RAW264.7/His-HA-NPs/Merge.tif]

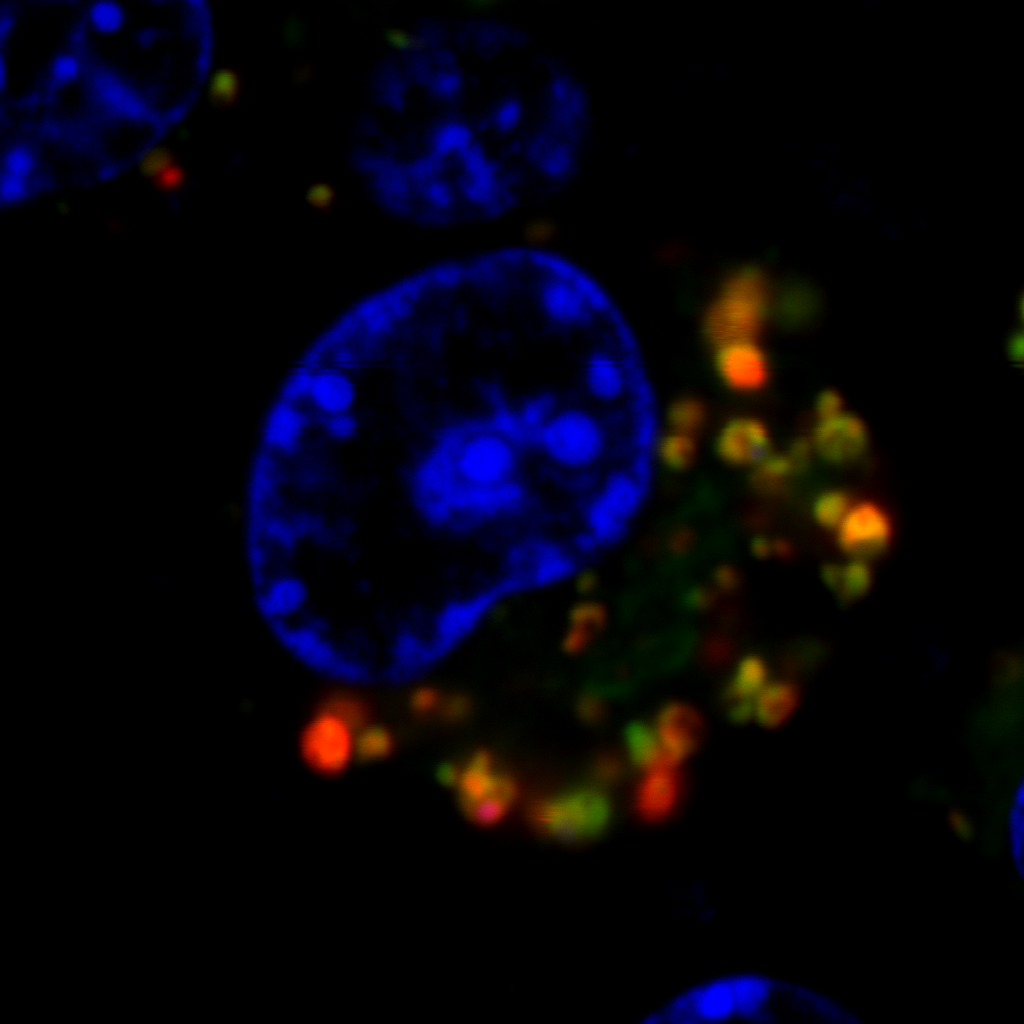

Supplement: Supplementary file 5 — Source data Fig. 2 [file 44321_2024_76_MOESM5_ESM.zip › Figure 2B/RAW264.7/His-HA-NPs/Zoom.tif]

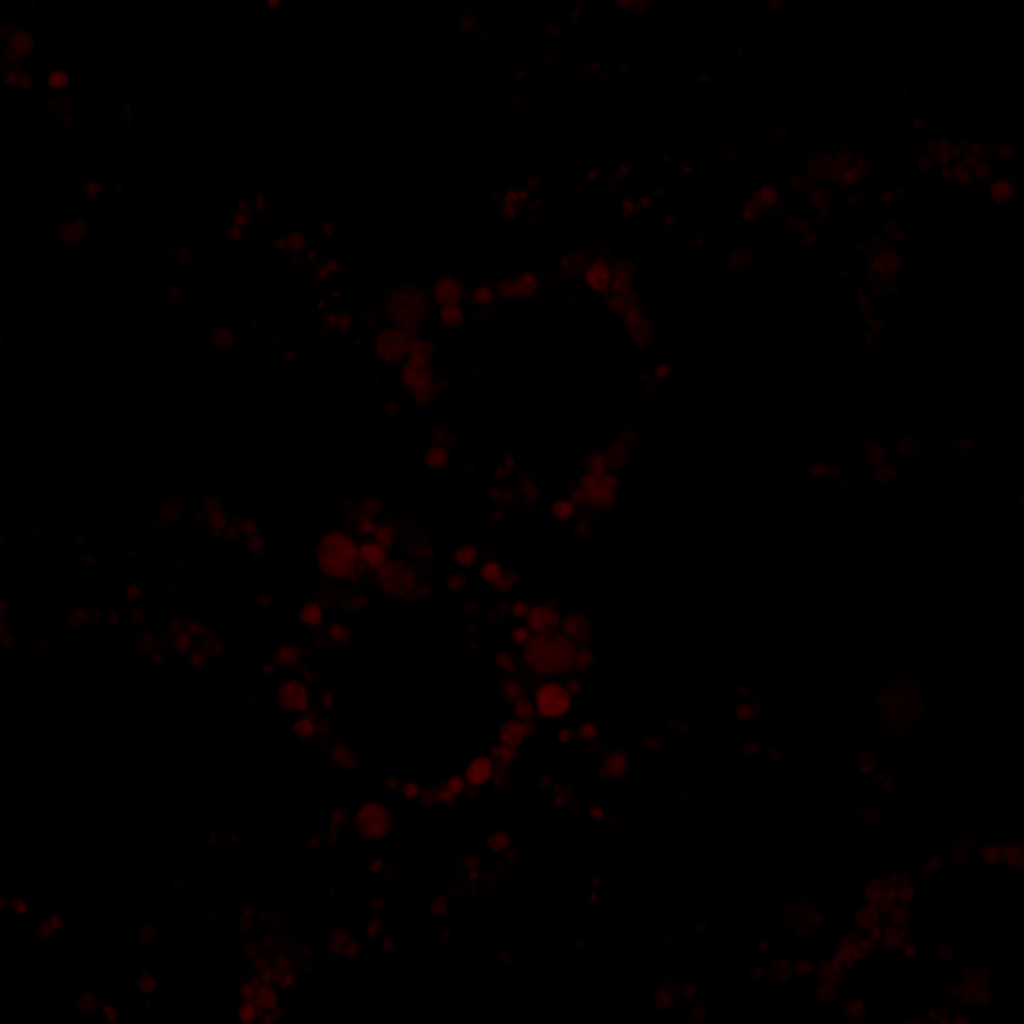

Supplement: Supplementary file 5 — Source data Fig. 2 [file 44321_2024_76_MOESM5_ESM.zip › Figure 2B/RAW264.7/His-HA/Hemagglutinin.tif]

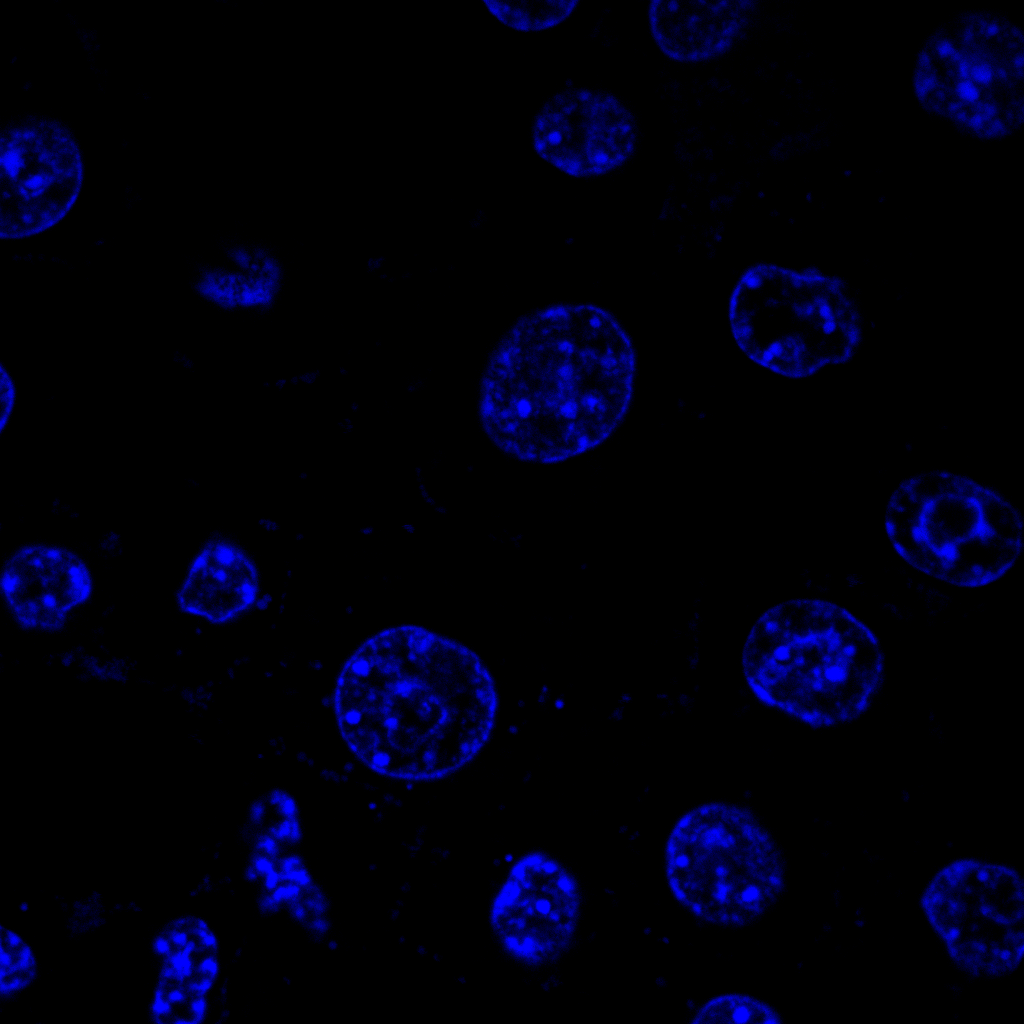

Supplement: Supplementary file 5 — Source data Fig. 2 [file 44321_2024_76_MOESM5_ESM.zip › Figure 2B/RAW264.7/His-HA/Hoechst.tif]

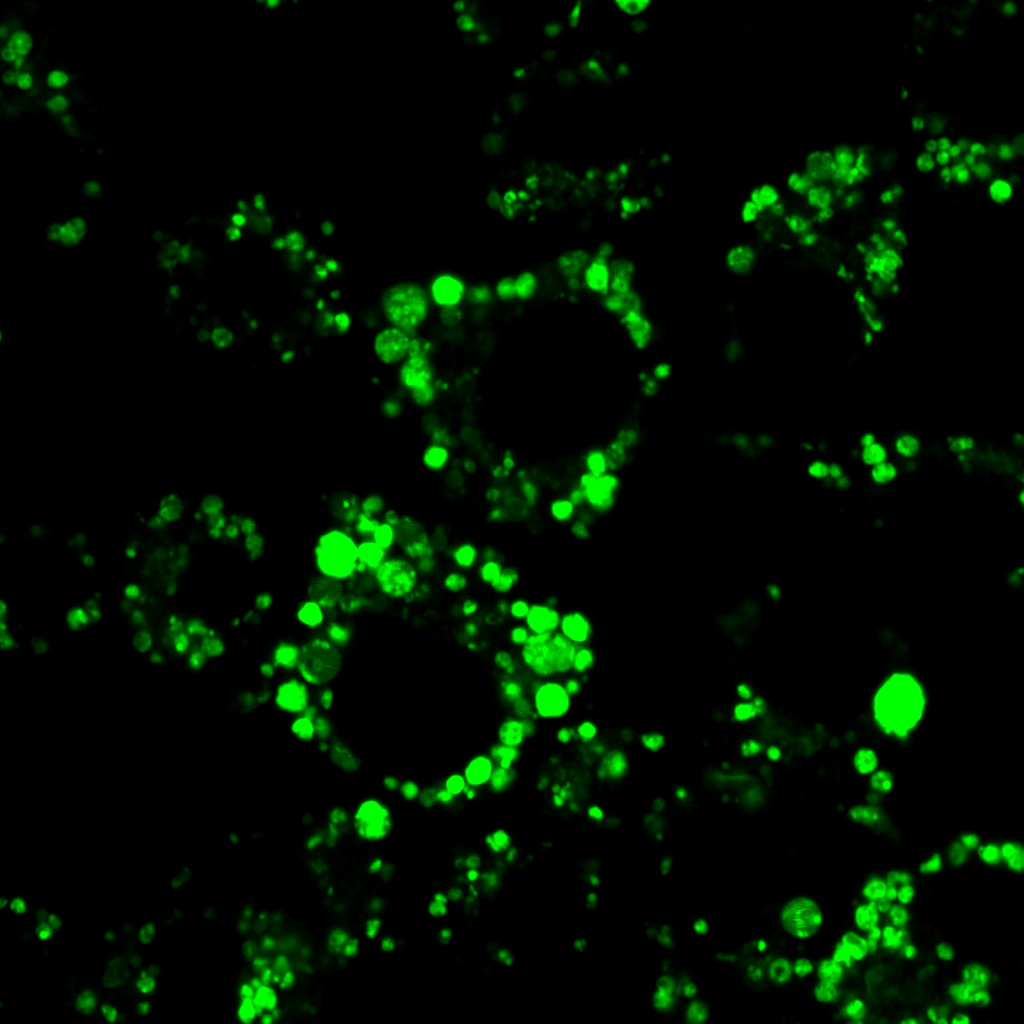

Supplement: Supplementary file 5 — Source data Fig. 2 [file 44321_2024_76_MOESM5_ESM.zip › Figure 2B/RAW264.7/His-HA/Lysotracker.tif]

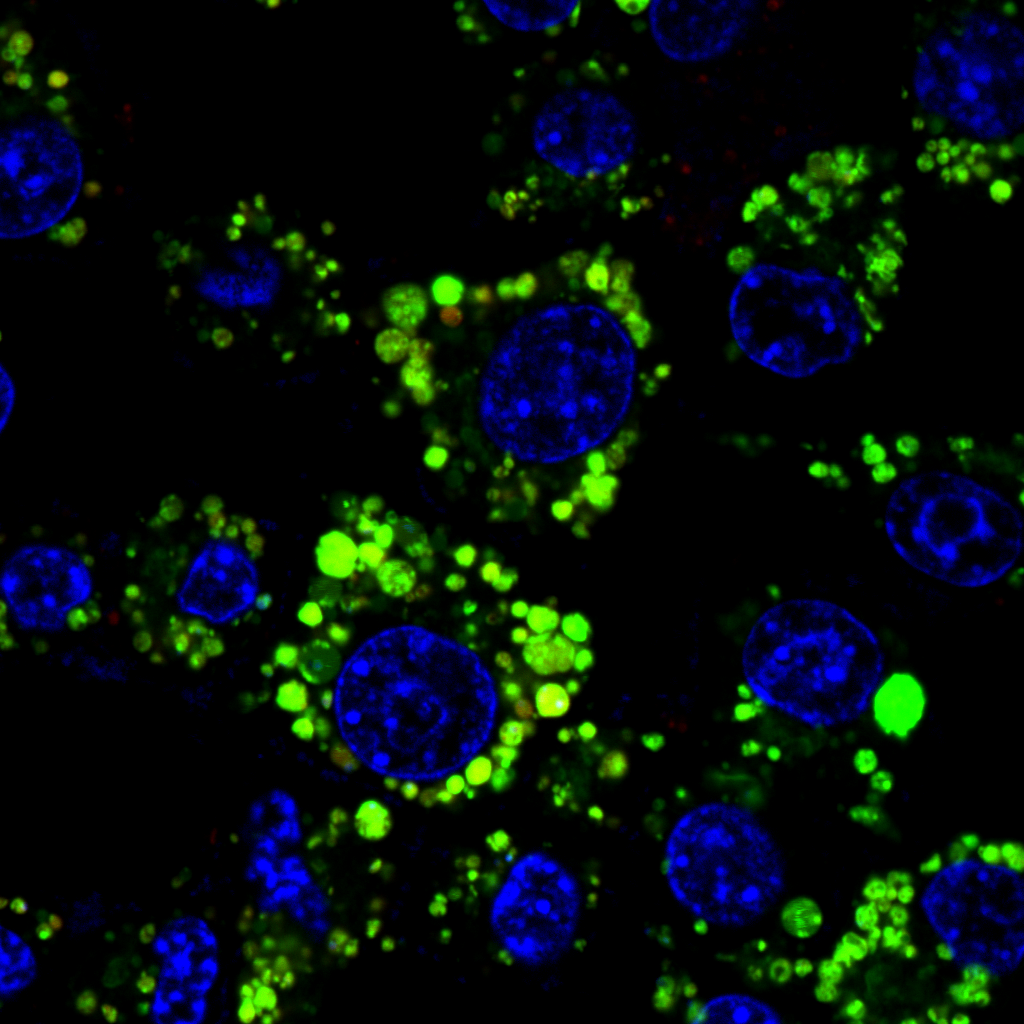

Supplement: Supplementary file 5 — Source data Fig. 2 [file 44321_2024_76_MOESM5_ESM.zip › Figure 2B/RAW264.7/His-HA/Merge.tif]

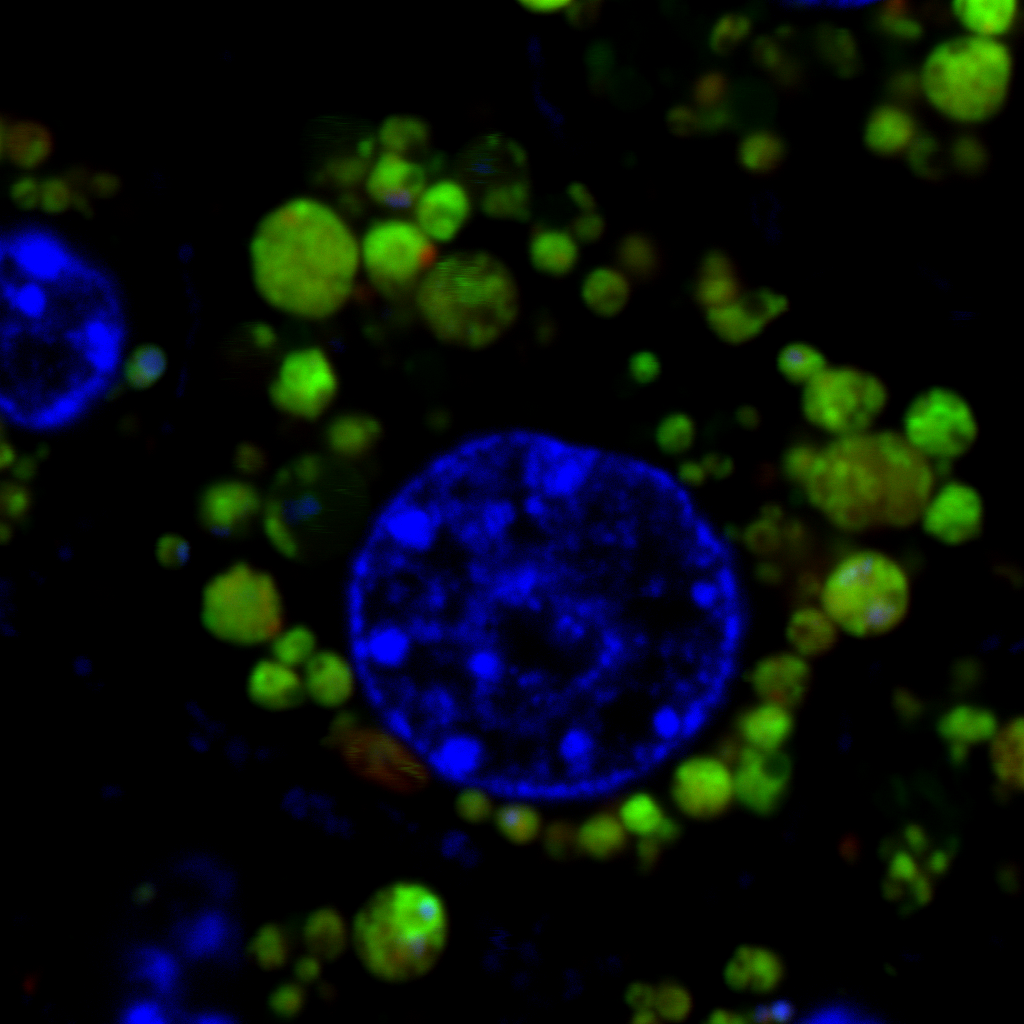

Supplement: Supplementary file 5 — Source data Fig. 2 [file 44321_2024_76_MOESM5_ESM.zip › Figure 2B/RAW264.7/His-HA/Zoom.tif]

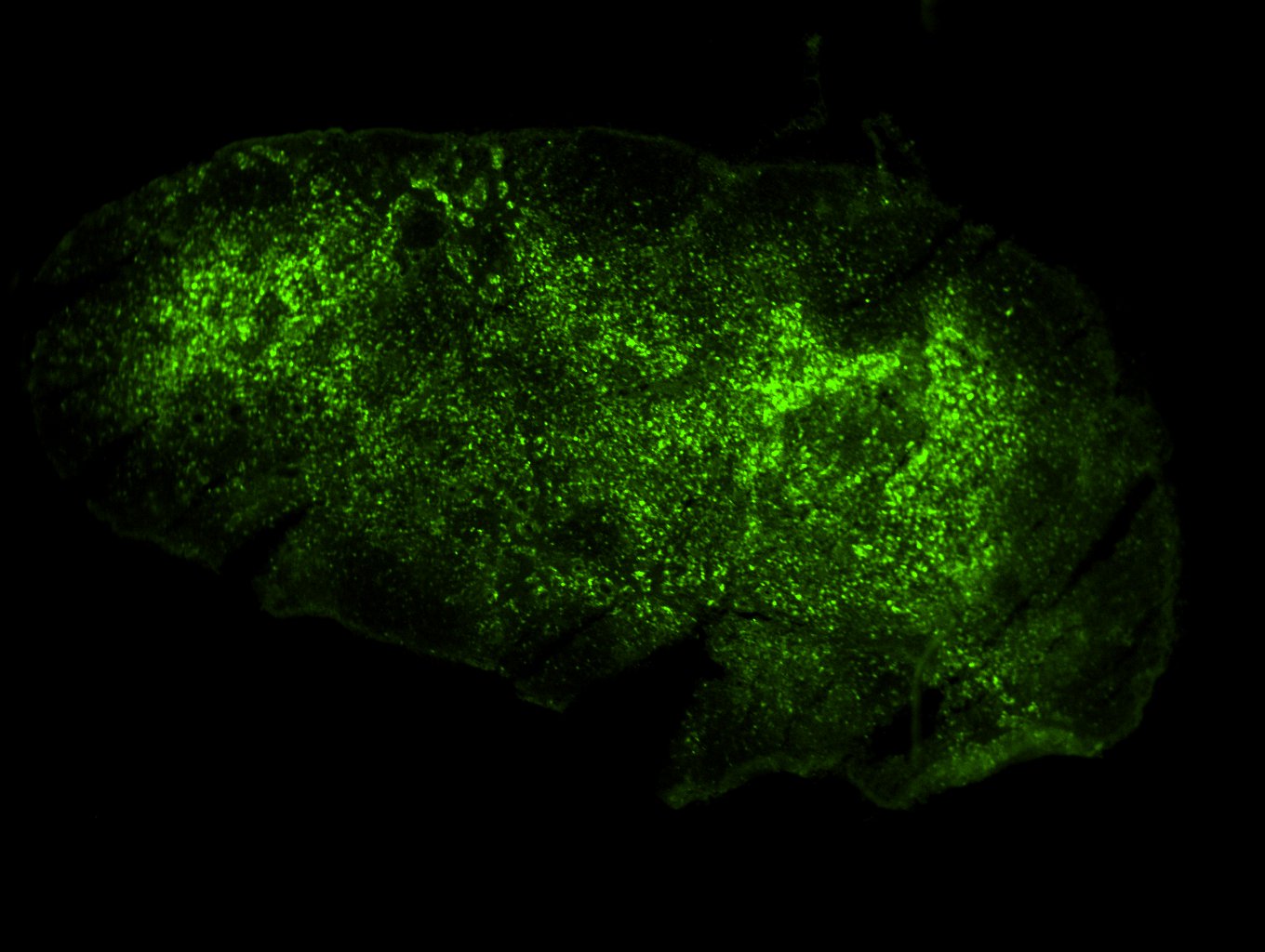

Supplement: Supplementary file 5 — Source data Fig. 2 [file 44321_2024_76_MOESM5_ESM.zip › Figure 2F/Day 0/His-HA-NPs/DC.jpg]

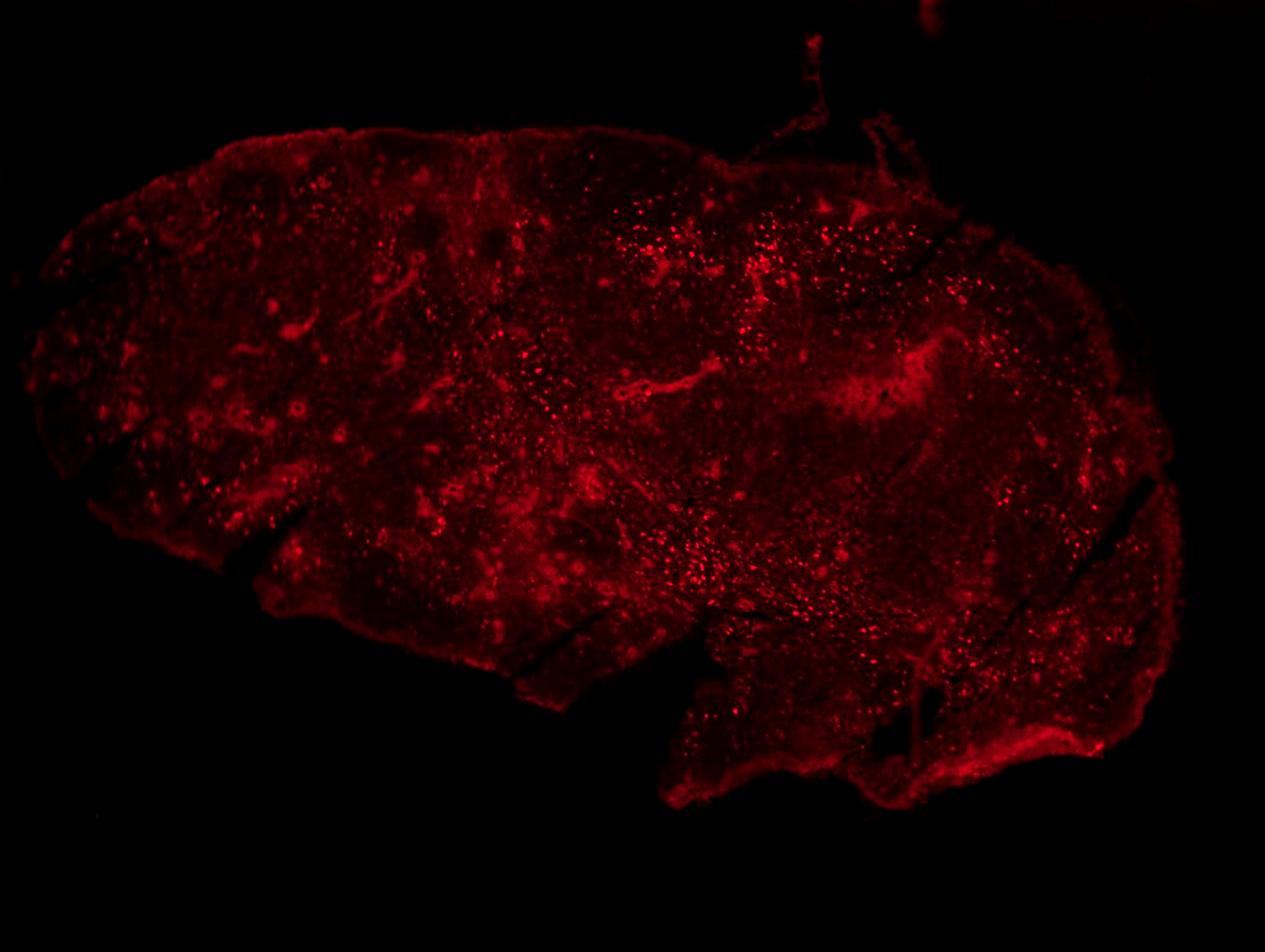

Supplement: Supplementary file 5 — Source data Fig. 2 [file 44321_2024_76_MOESM5_ESM.zip › Figure 2F/Day 0/His-HA-NPs/HA.jpg]

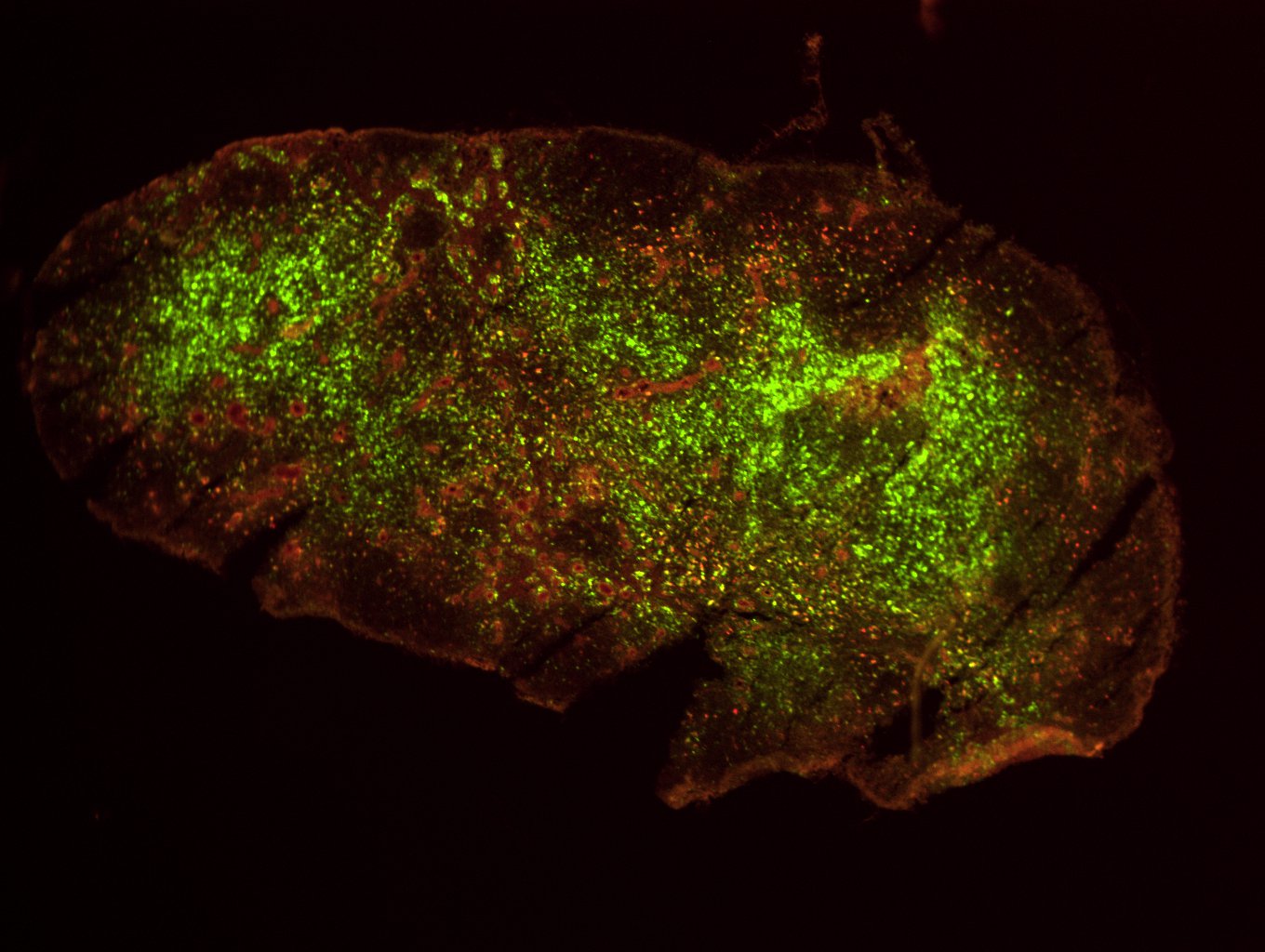

Supplement: Supplementary file 5 — Source data Fig. 2 [file 44321_2024_76_MOESM5_ESM.zip › Figure 2F/Day 0/His-HA-NPs/Merge.jpg]

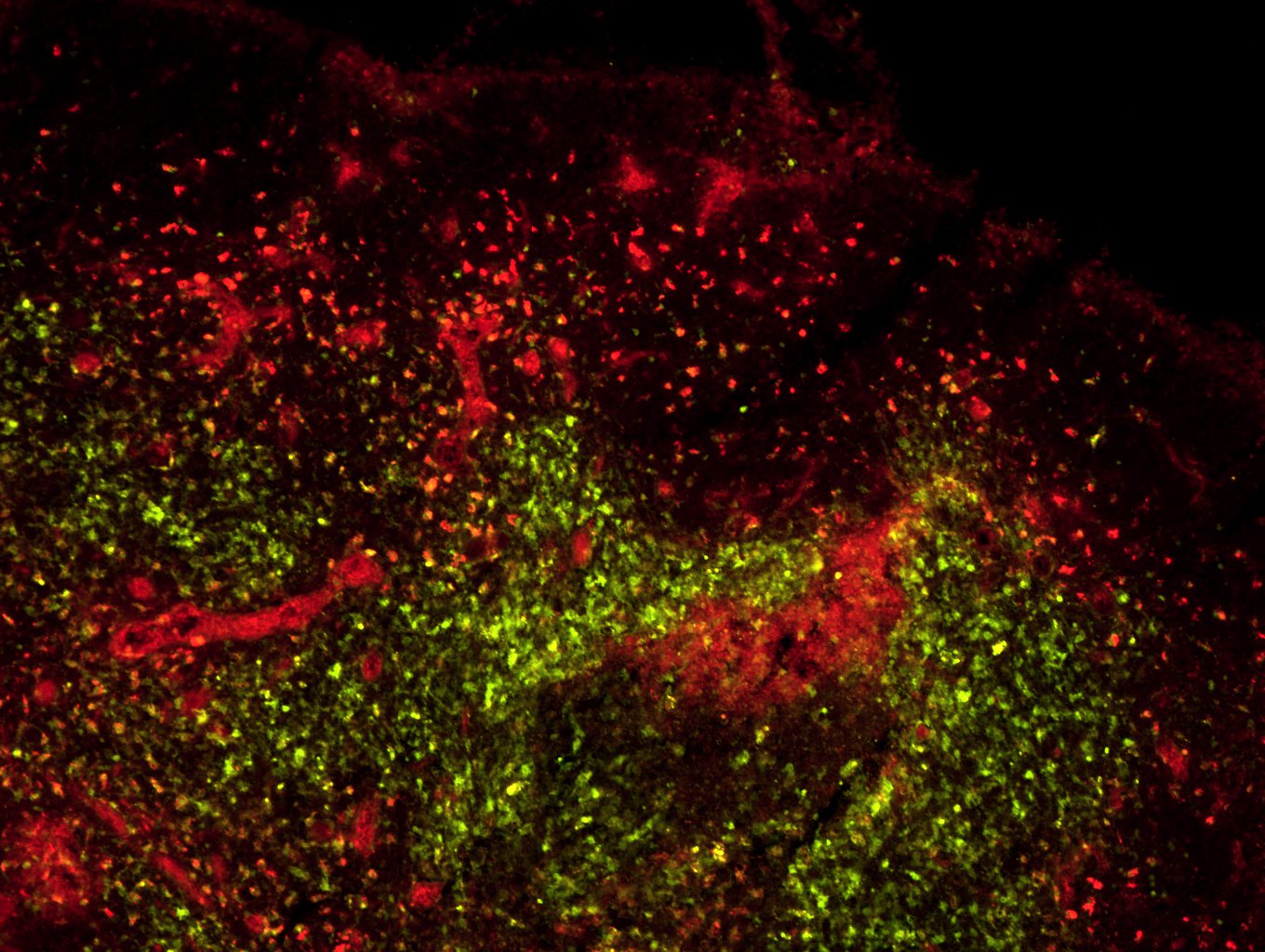

Supplement: Supplementary file 5 — Source data Fig. 2 [file 44321_2024_76_MOESM5_ESM.zip › Figure 2F/Day 0/His-HA-NPs/Zoom.jpg]

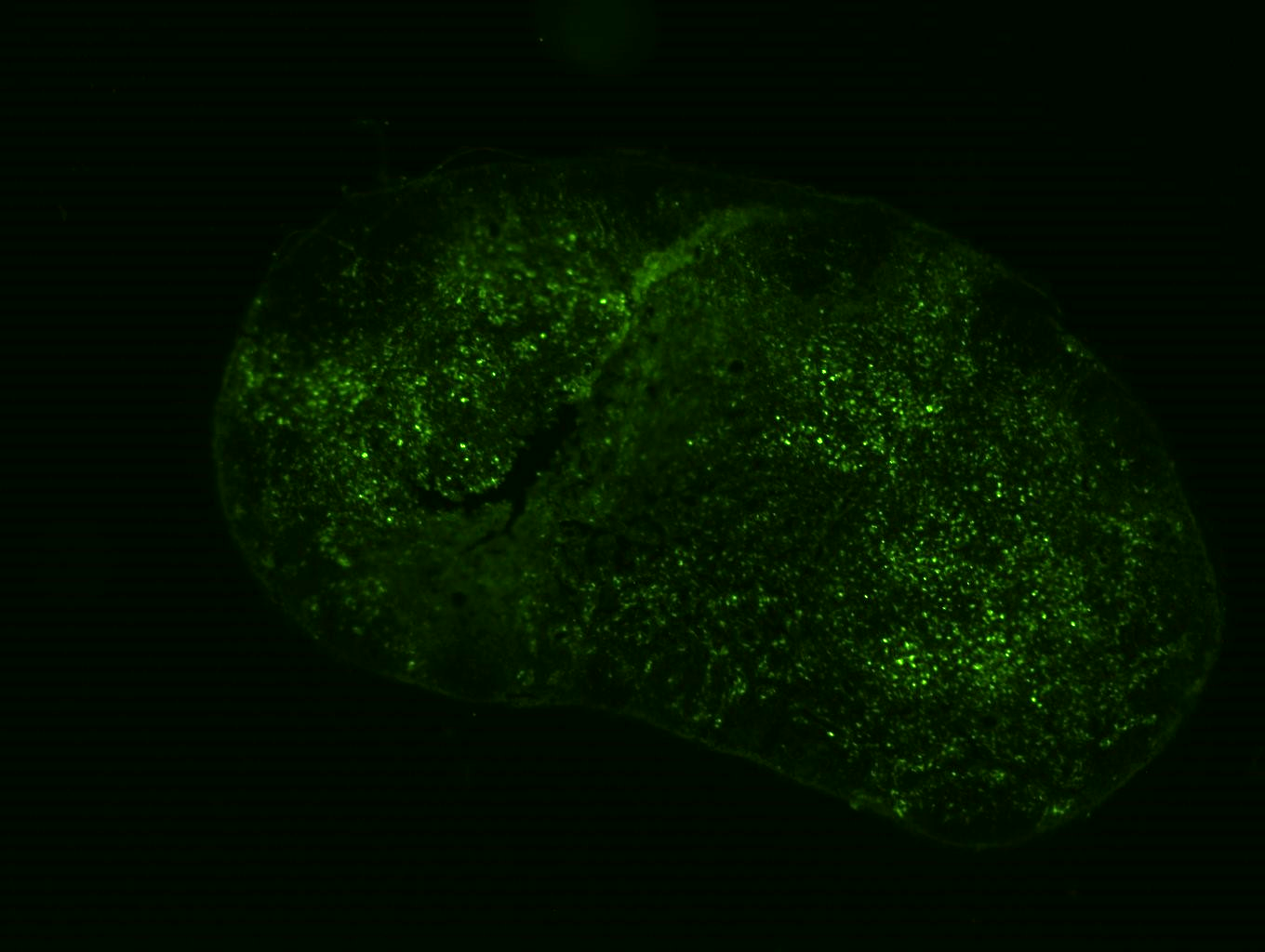

Supplement: Supplementary file 5 — Source data Fig. 2 [file 44321_2024_76_MOESM5_ESM.zip › Figure 2F/Day 0/His-HA/DC.jpg]

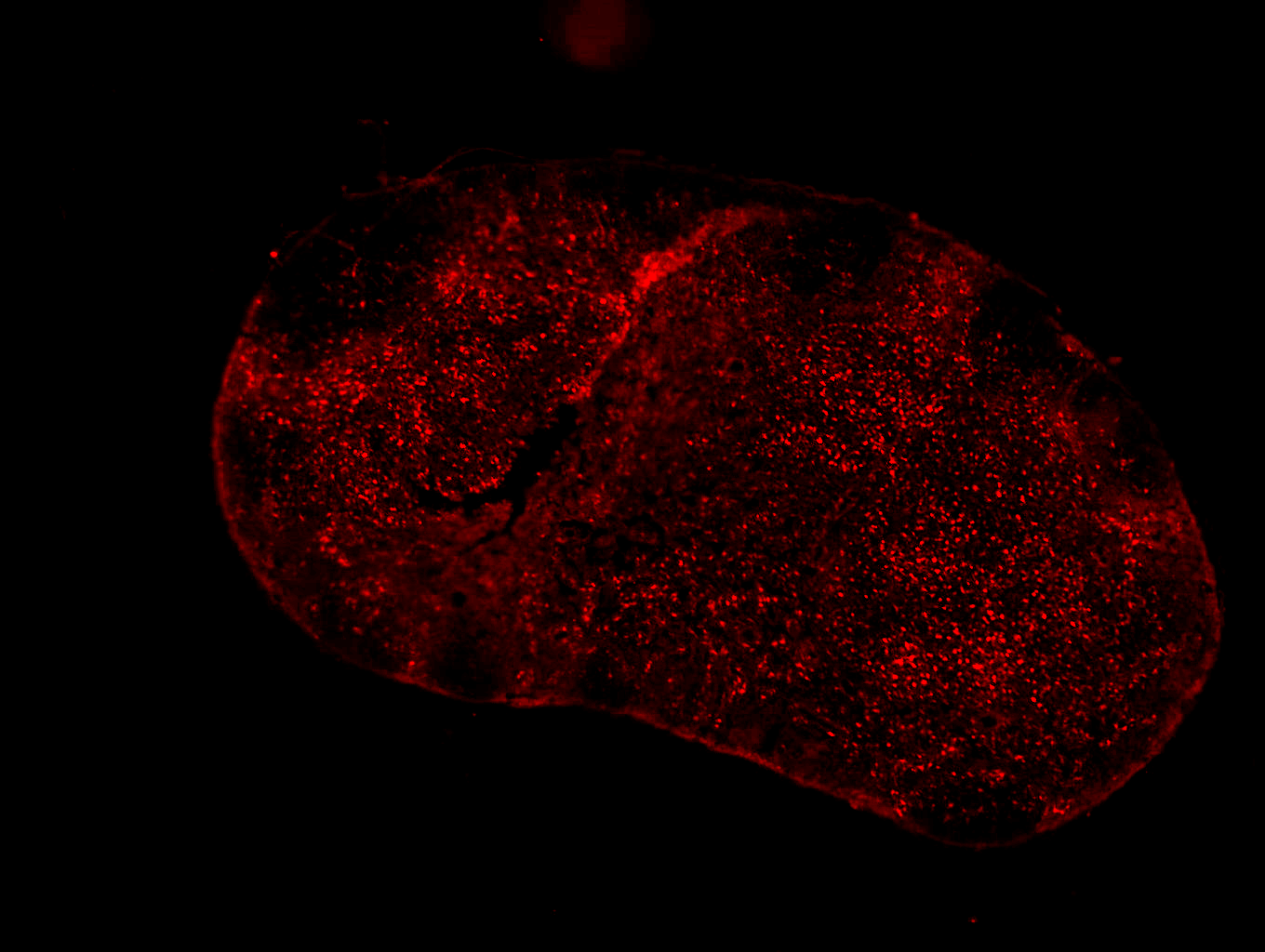

Supplement: Supplementary file 5 — Source data Fig. 2 [file 44321_2024_76_MOESM5_ESM.zip › Figure 2F/Day 0/His-HA/HA.tif]

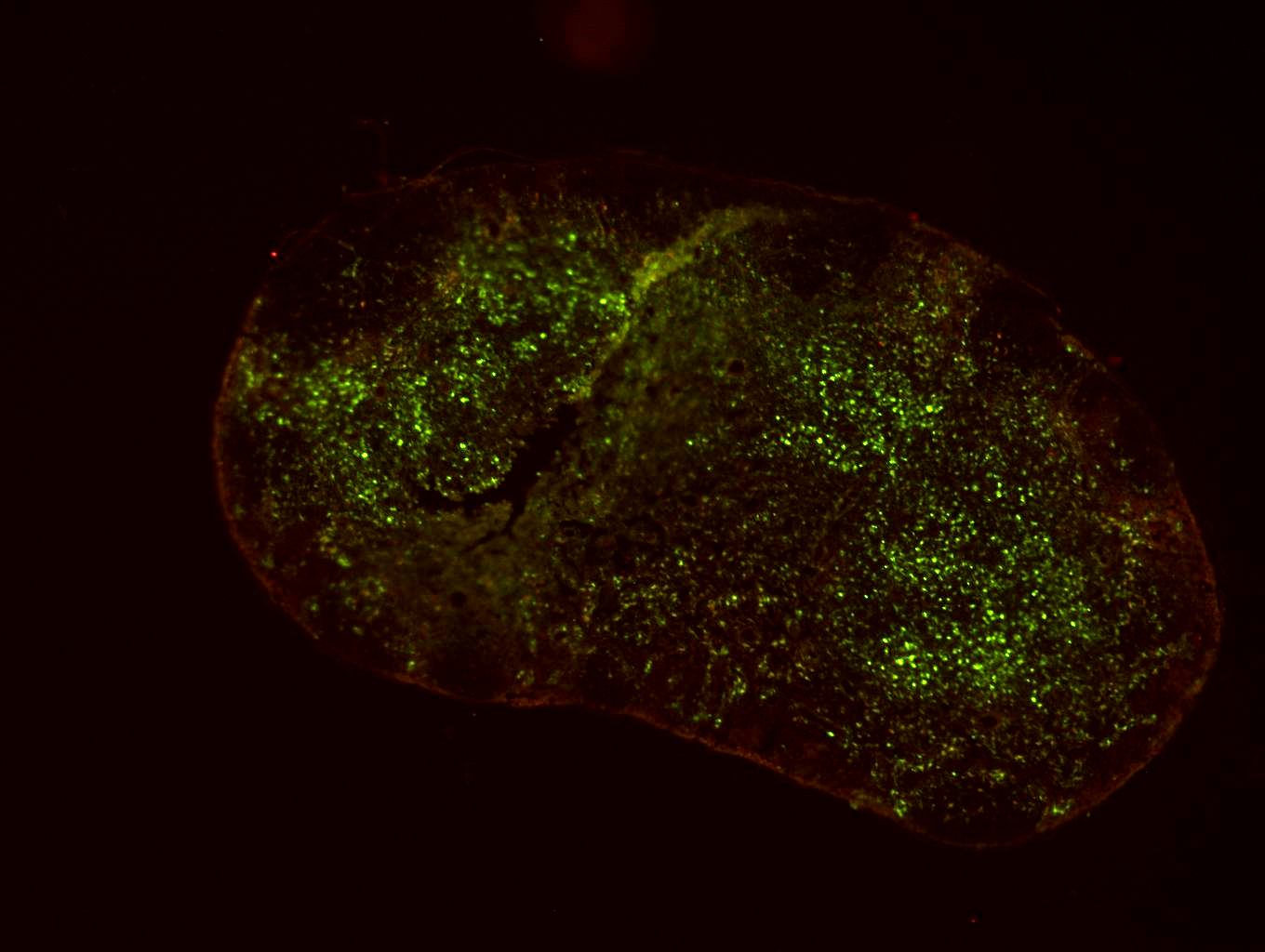

Supplement: Supplementary file 5 — Source data Fig. 2 [file 44321_2024_76_MOESM5_ESM.zip › Figure 2F/Day 0/His-HA/Merge.tif]

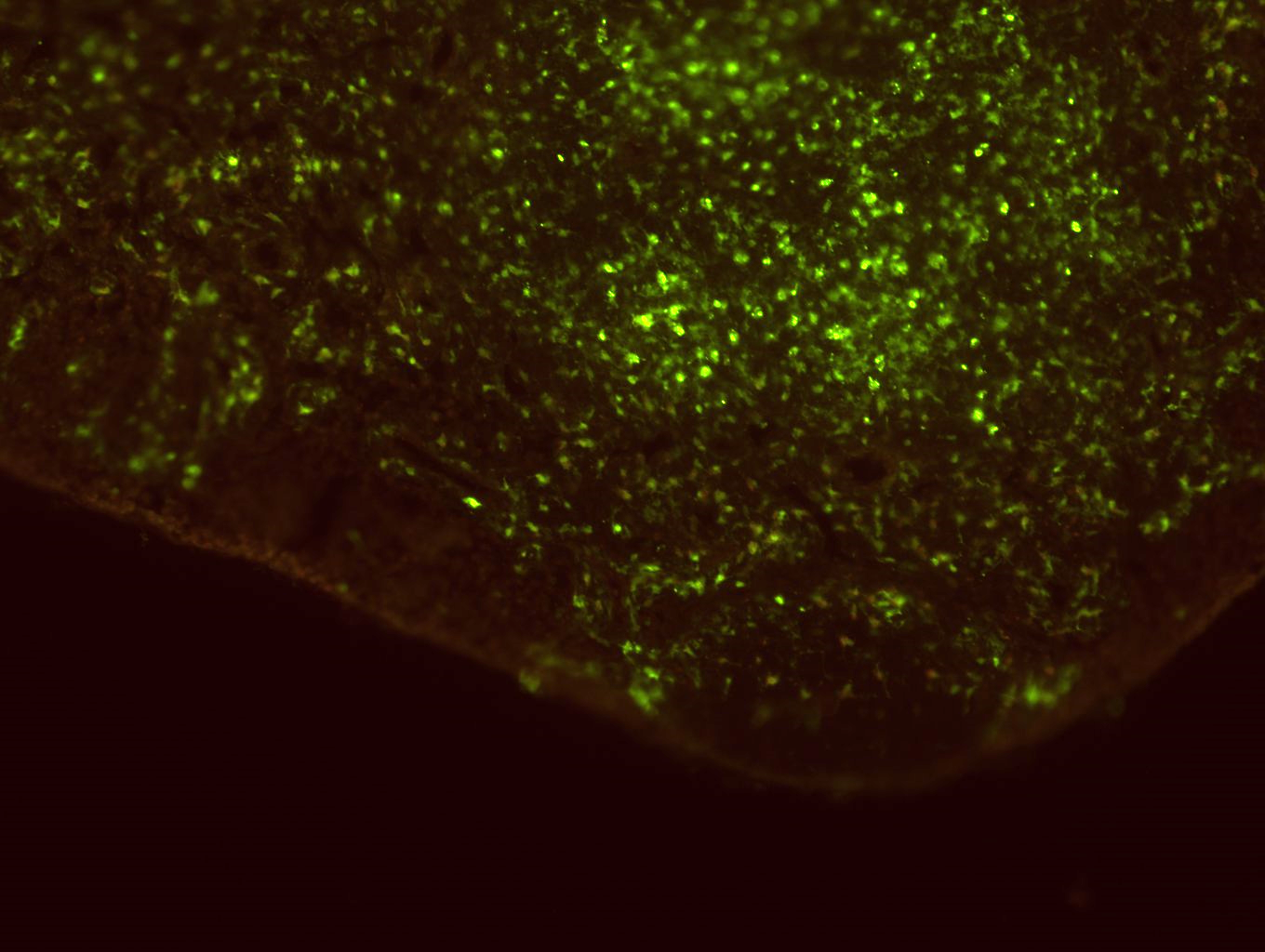

Supplement: Supplementary file 5 — Source data Fig. 2 [file 44321_2024_76_MOESM5_ESM.zip › Figure 2F/Day 0/His-HA/Zoom.tif]

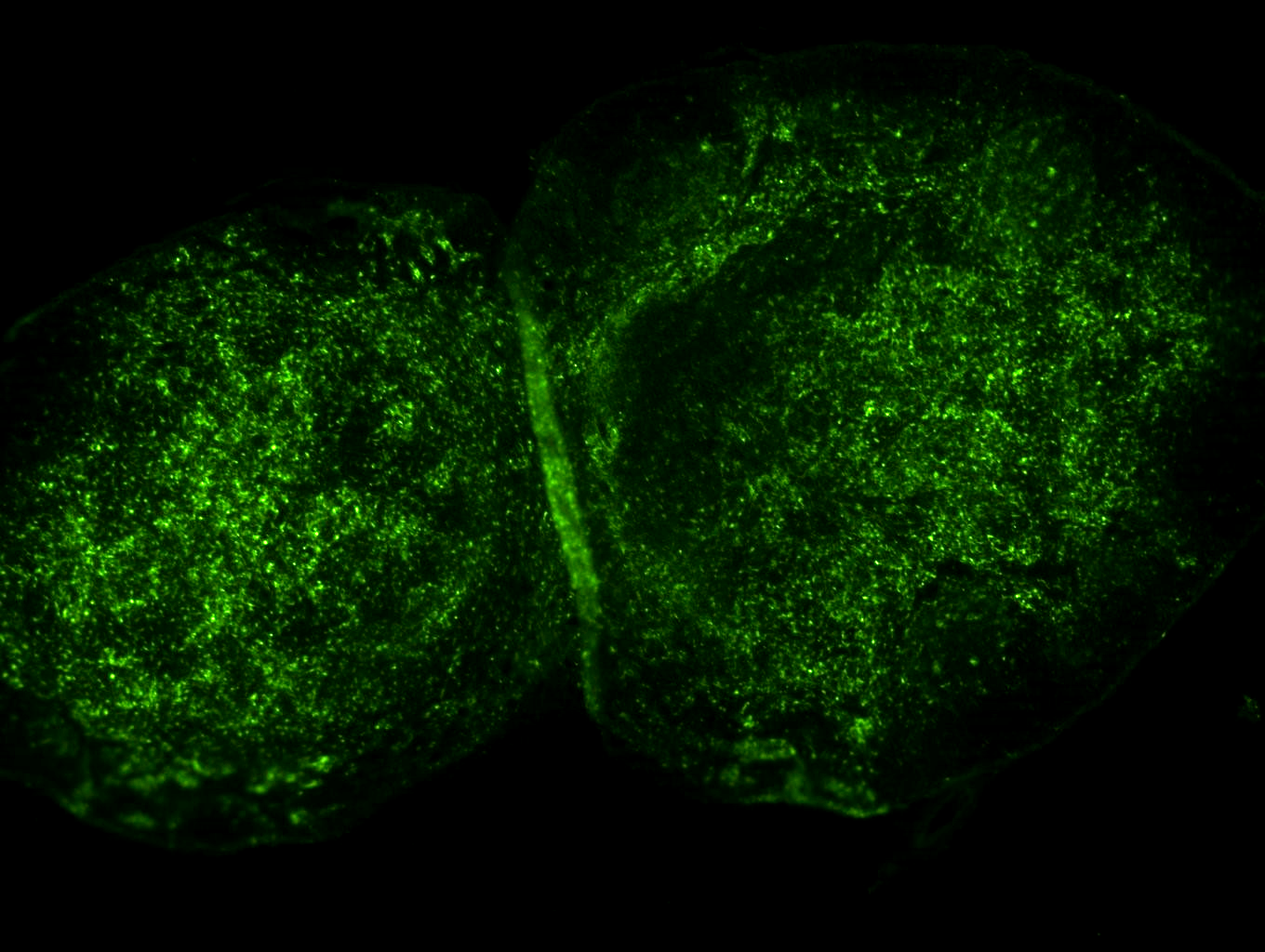

Supplement: Supplementary file 5 — Source data Fig. 2 [file 44321_2024_76_MOESM5_ESM.zip › Figure 2F/Day 7/His-HA-NPs/DC.jpg]

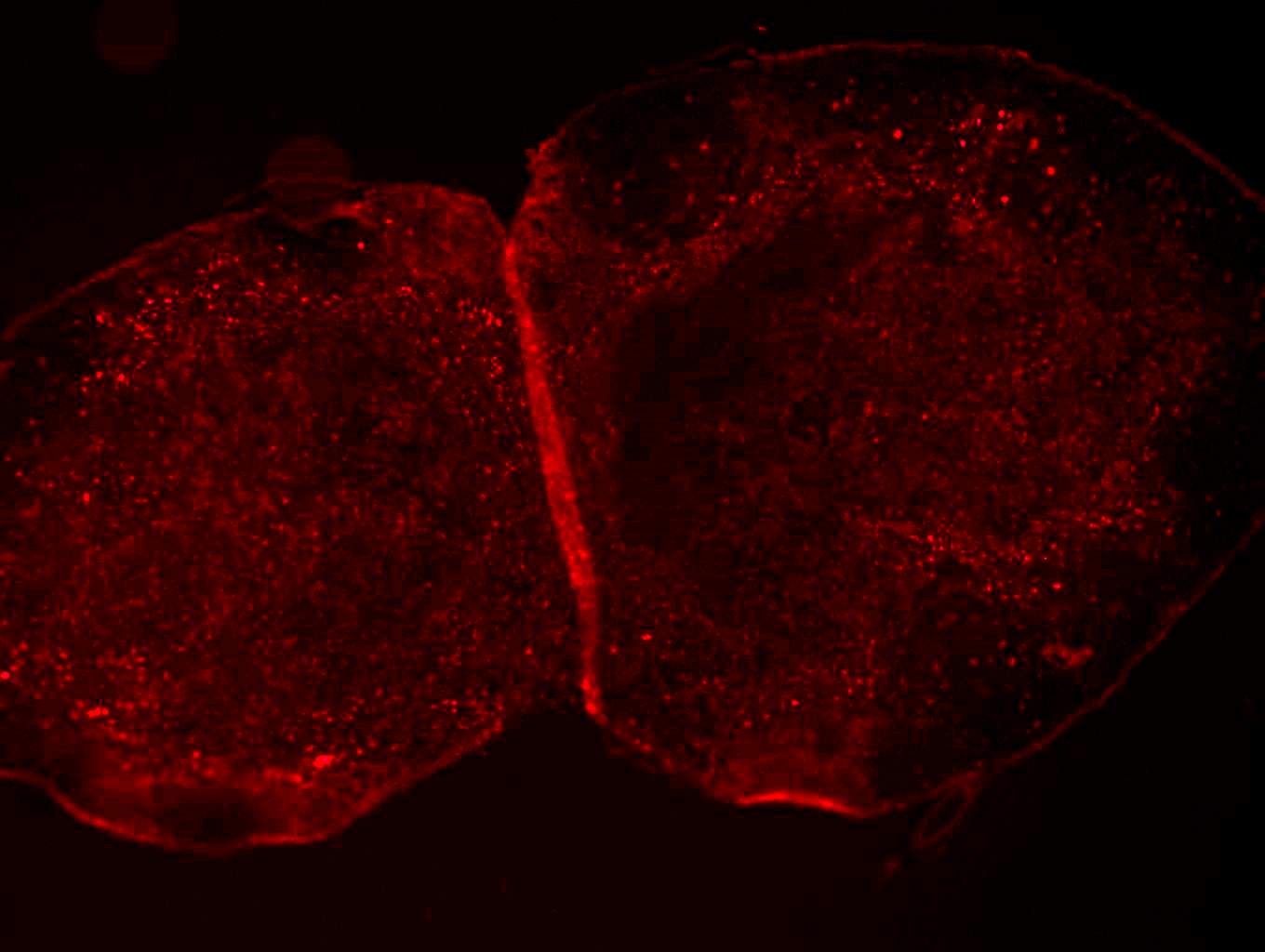

Supplement: Supplementary file 5 — Source data Fig. 2 [file 44321_2024_76_MOESM5_ESM.zip › Figure 2F/Day 7/His-HA-NPs/HA.jpg]

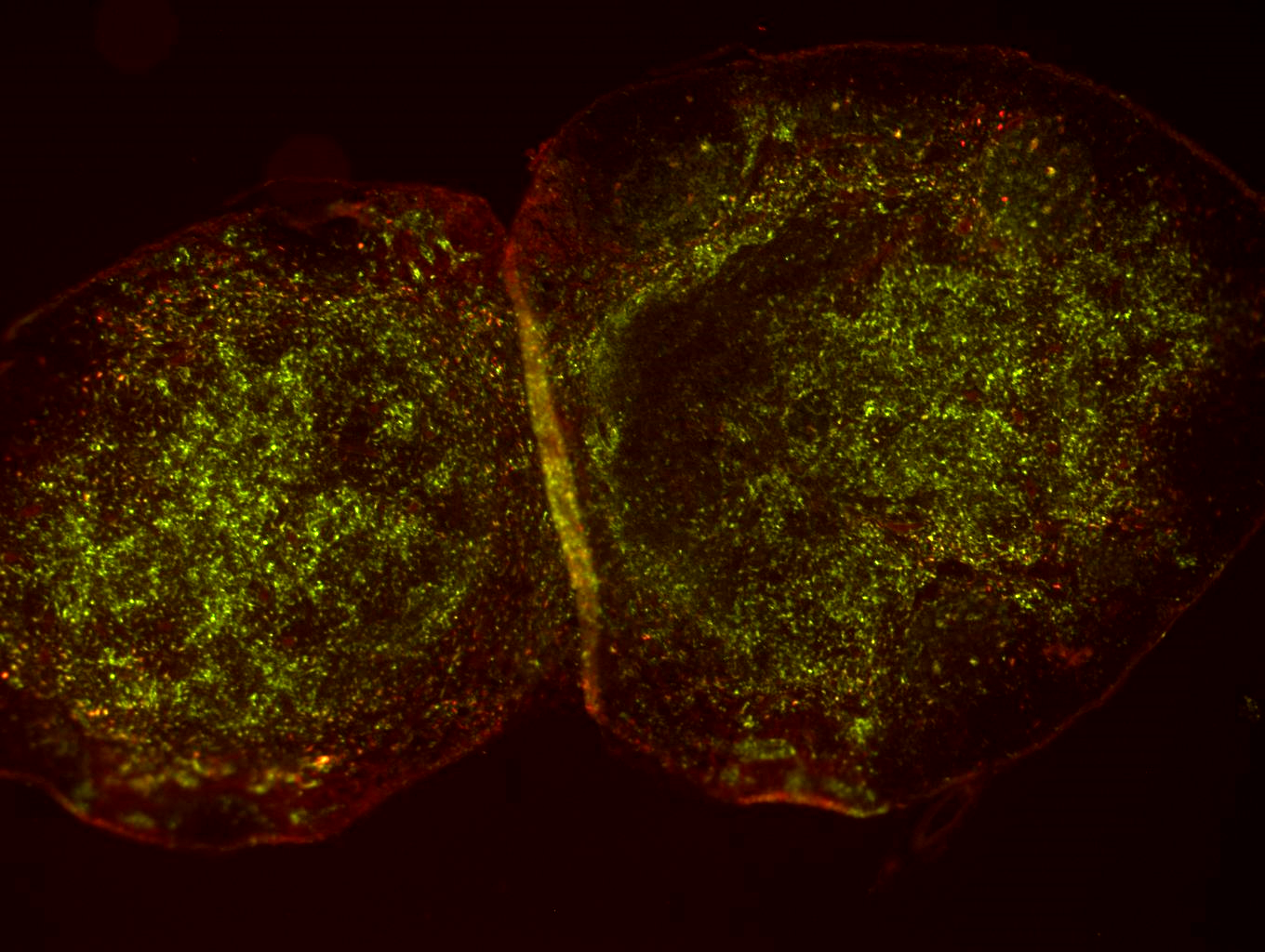

Supplement: Supplementary file 5 — Source data Fig. 2 [file 44321_2024_76_MOESM5_ESM.zip › Figure 2F/Day 7/His-HA-NPs/Merge.tif]

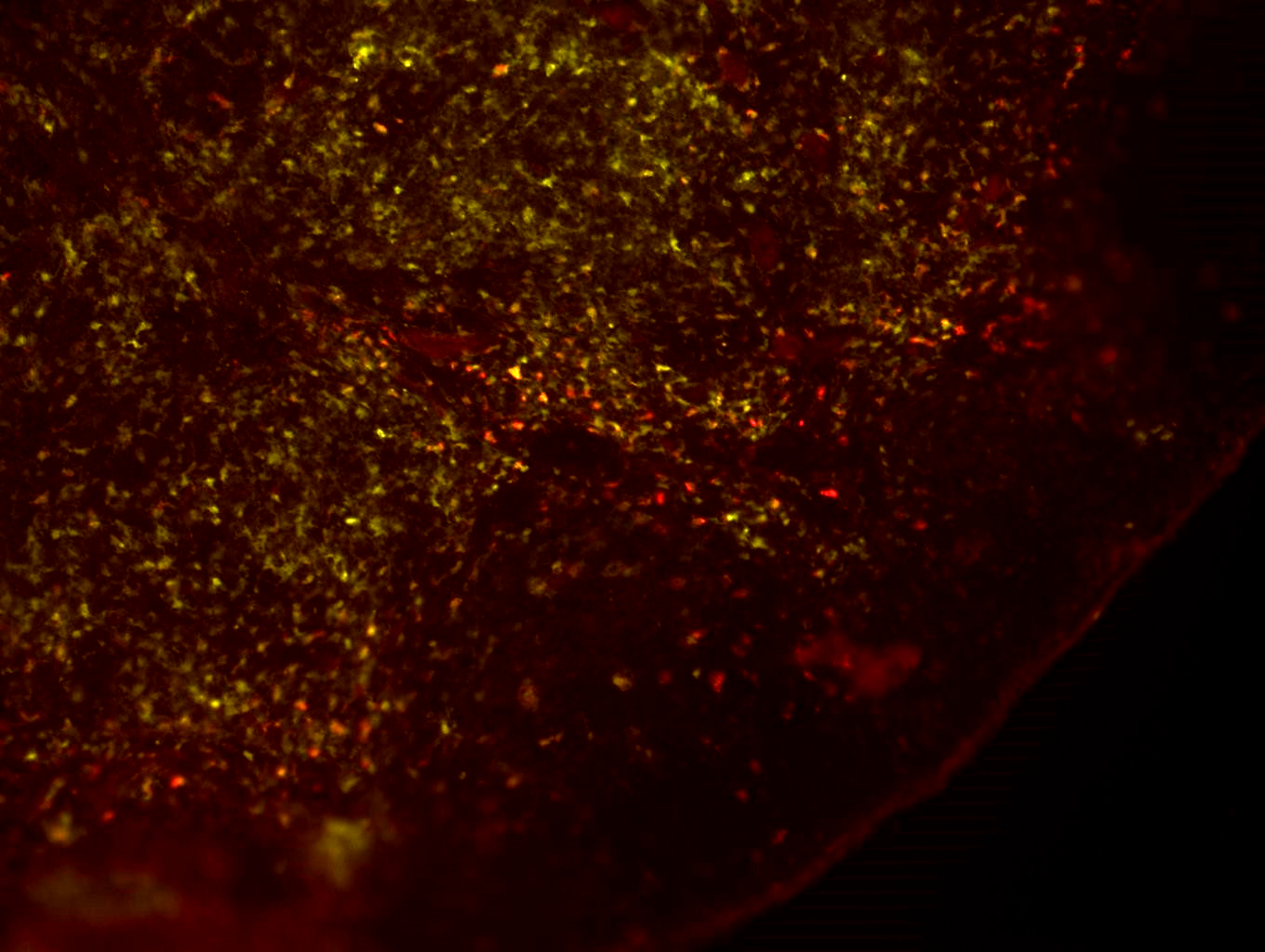

Supplement: Supplementary file 5 — Source data Fig. 2 [file 44321_2024_76_MOESM5_ESM.zip › Figure 2F/Day 7/His-HA-NPs/Zoom.jpg]

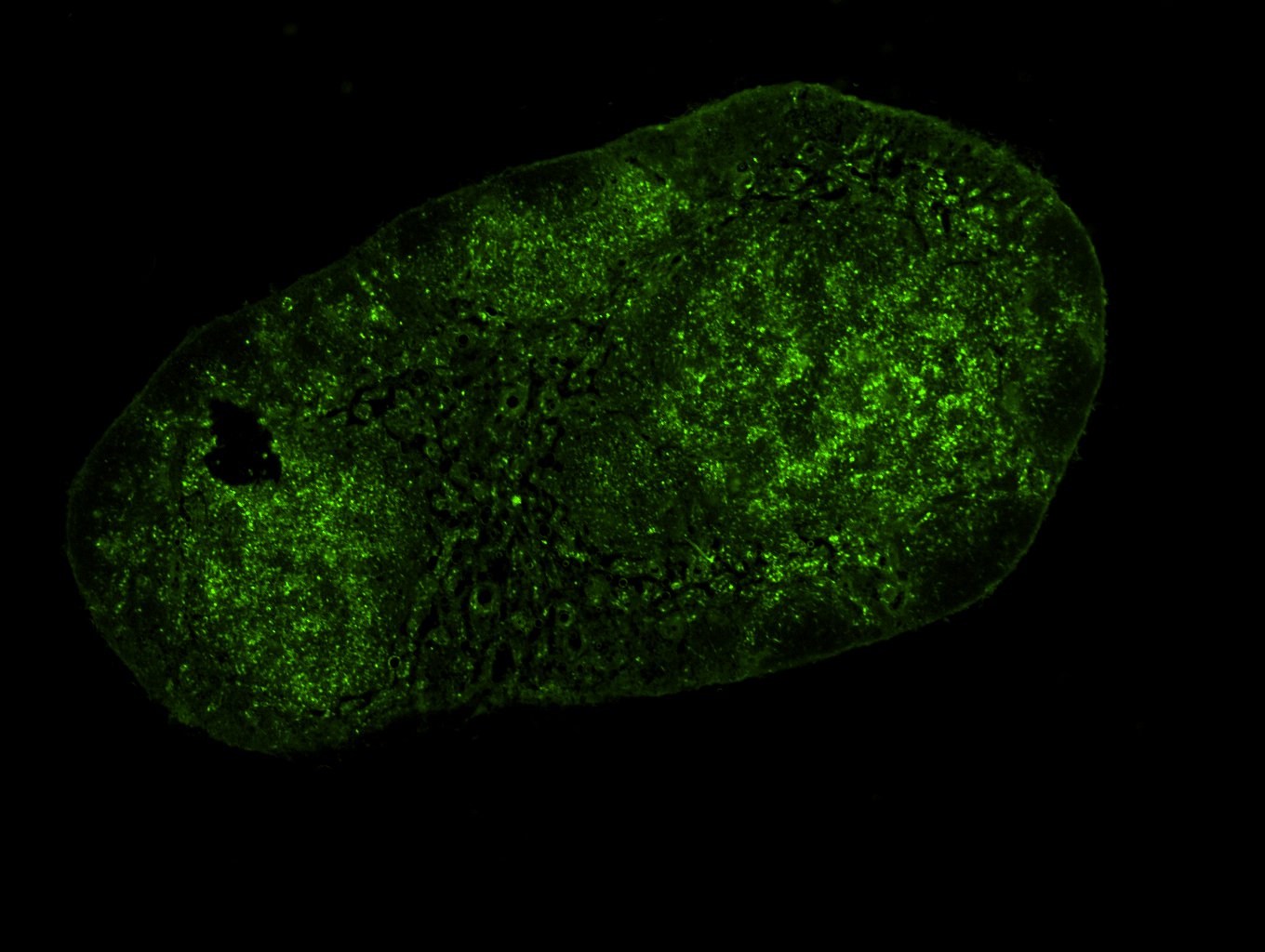

Supplement: Supplementary file 5 — Source data Fig. 2 [file 44321_2024_76_MOESM5_ESM.zip › Figure 2F/Day 7/His-HA/DC.jpg]

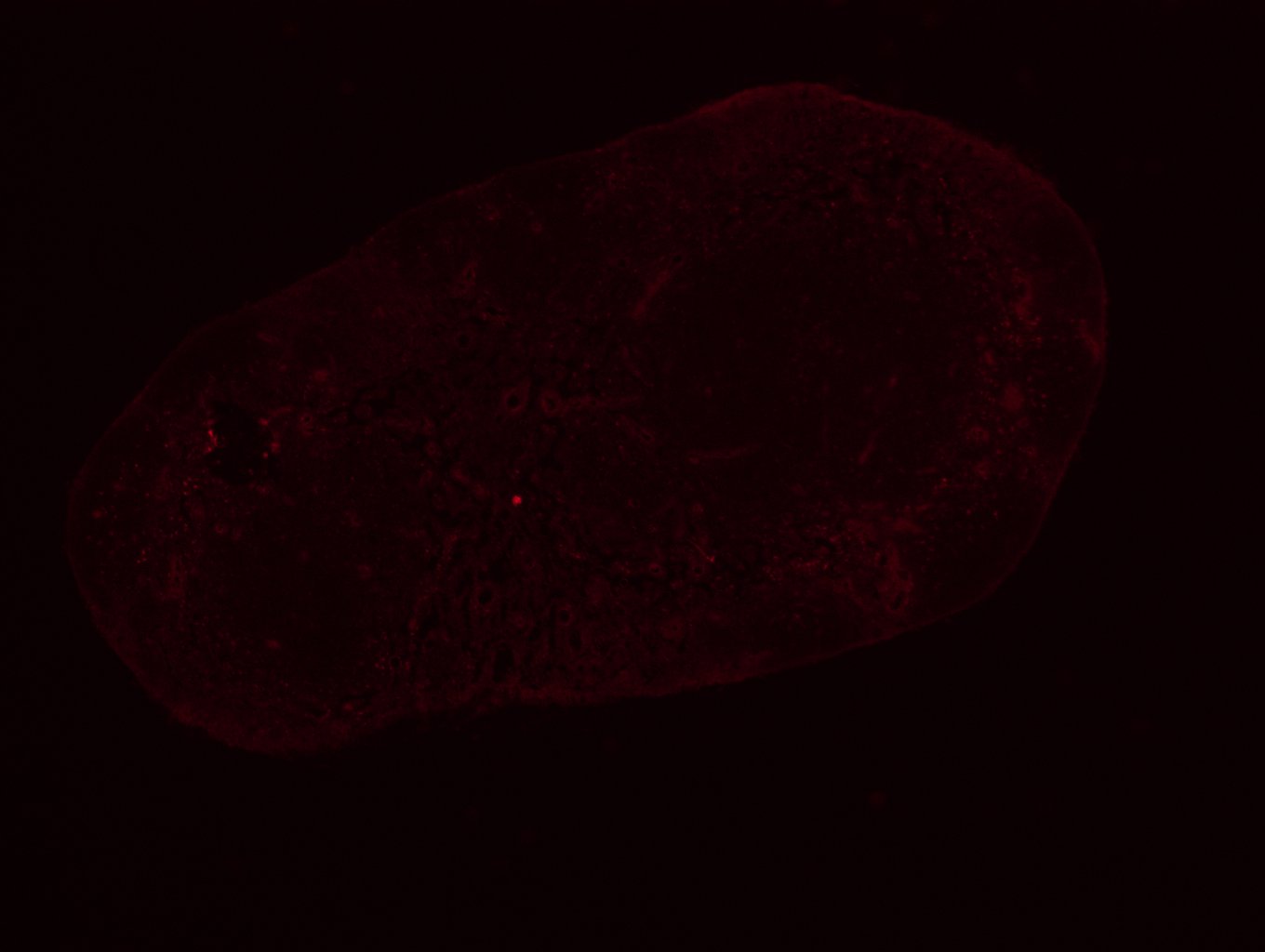

Supplement: Supplementary file 5 — Source data Fig. 2 [file 44321_2024_76_MOESM5_ESM.zip › Figure 2F/Day 7/His-HA/HA.jpg]

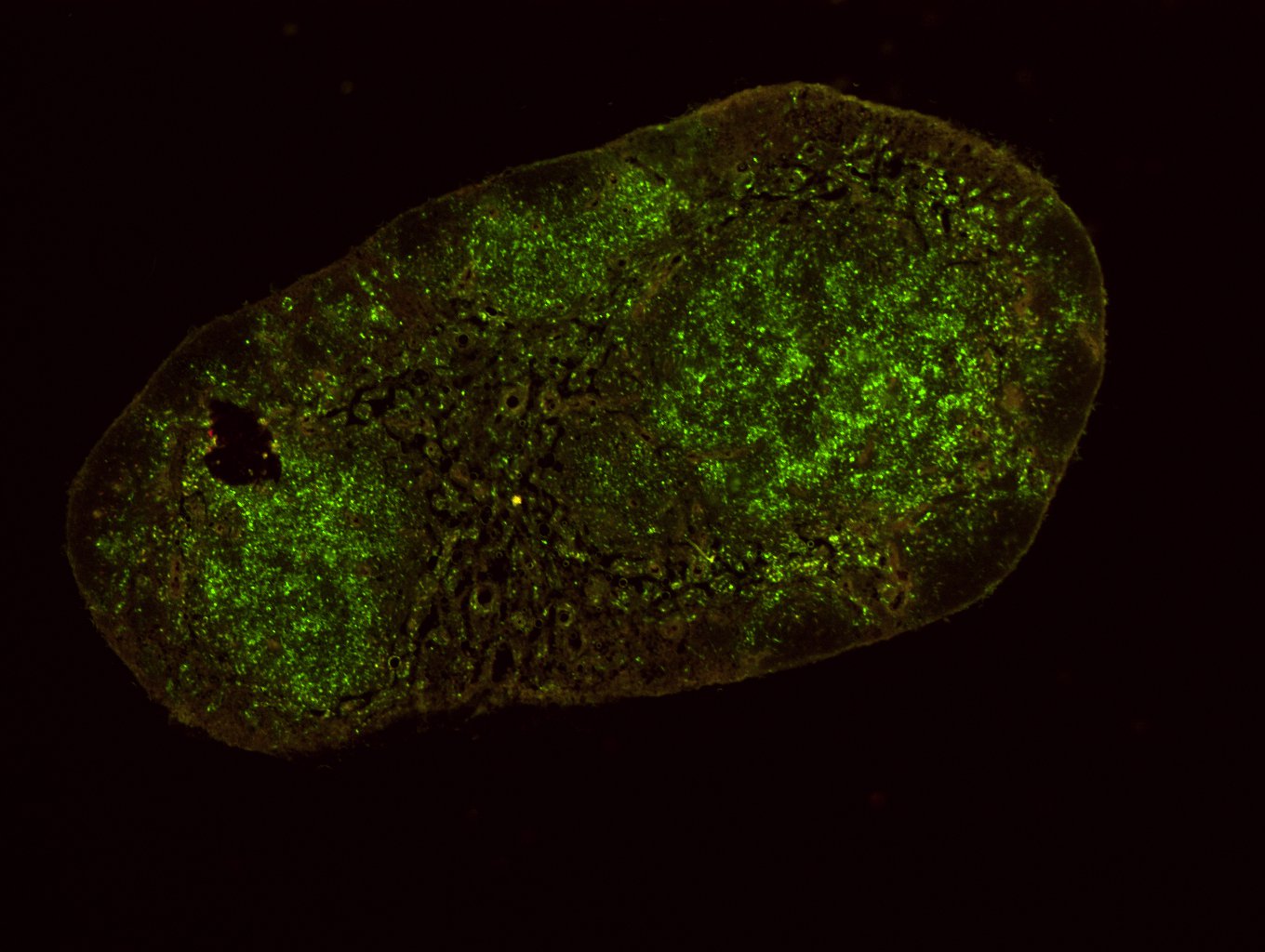

Supplement: Supplementary file 5 — Source data Fig. 2 [file 44321_2024_76_MOESM5_ESM.zip › Figure 2F/Day 7/His-HA/Merge.jpg]

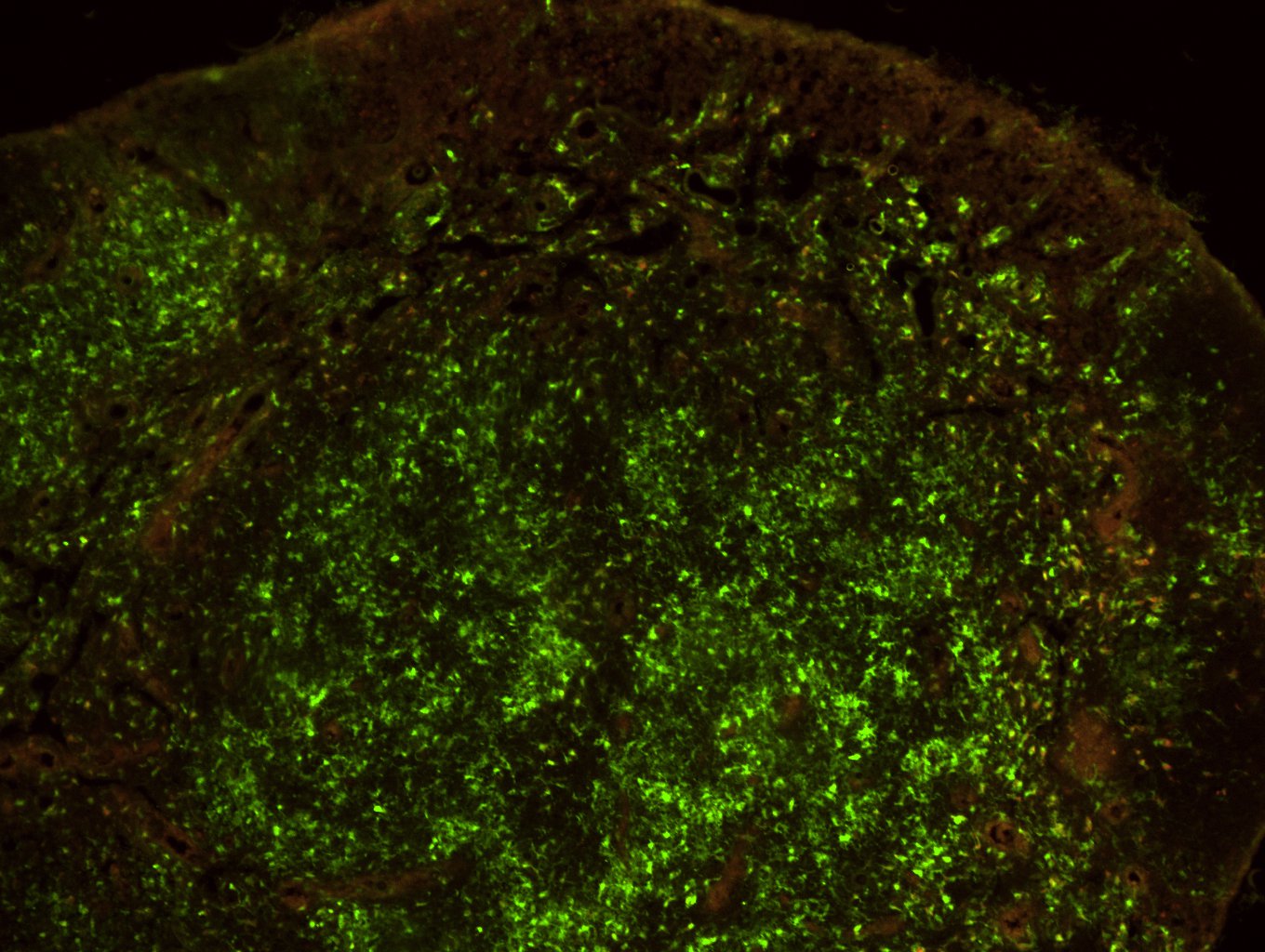

Supplement: Supplementary file 5 — Source data Fig. 2 [file 44321_2024_76_MOESM5_ESM.zip › Figure 2F/Day 7/His-HA/Zoom.jpg]

## Slide 1
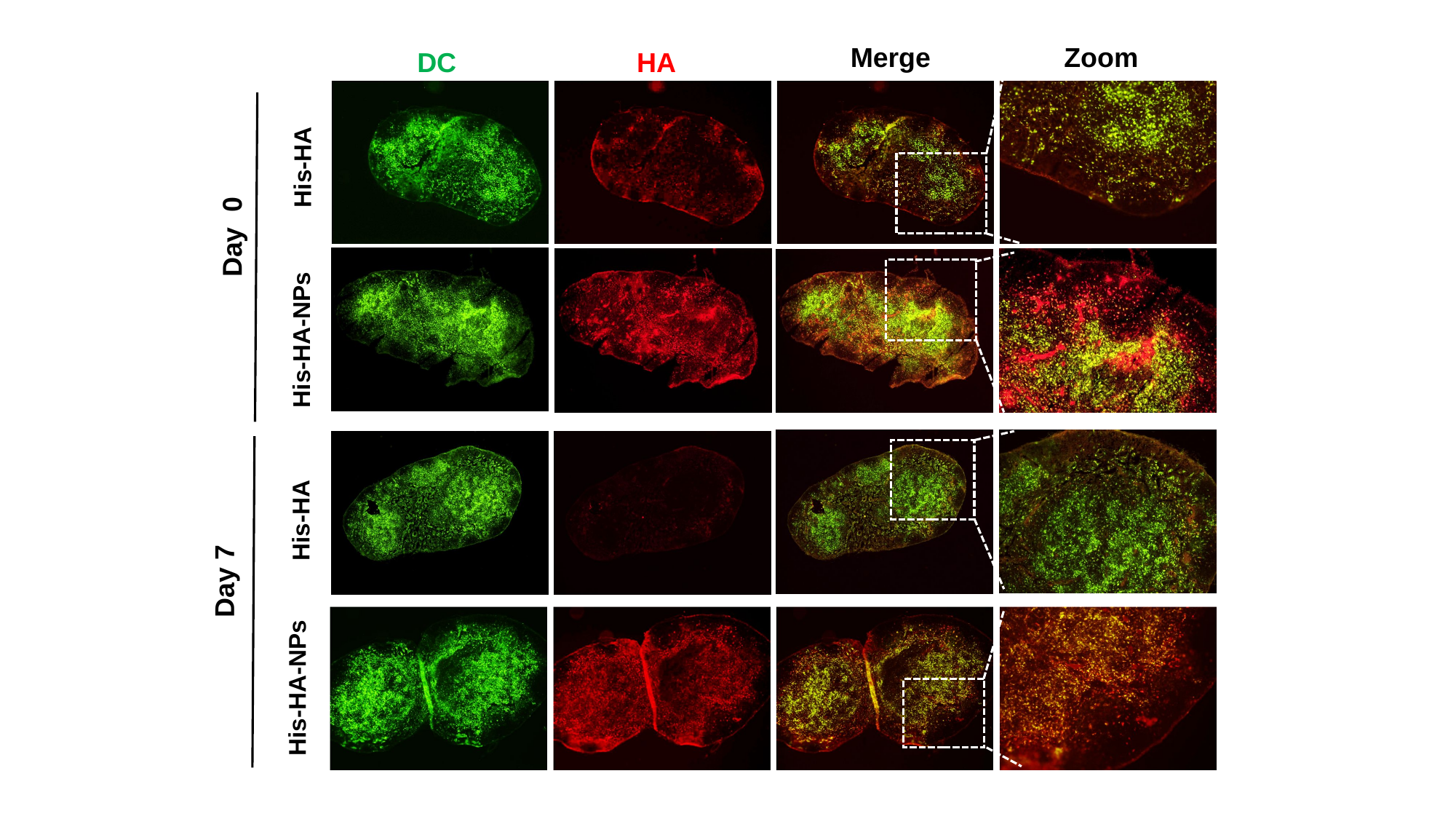

Merge
Zoom
DC
HA
His-HA
Day 0
His-HA-NPs
His-HA
Day 7
His-HA-NPs

Supplement: Supplementary file 5 — Source data Fig. 2 [file 44321_2024_76_MOESM5_ESM.zip › Figure 2F/Figure 2F.pptx]

## Slide 1
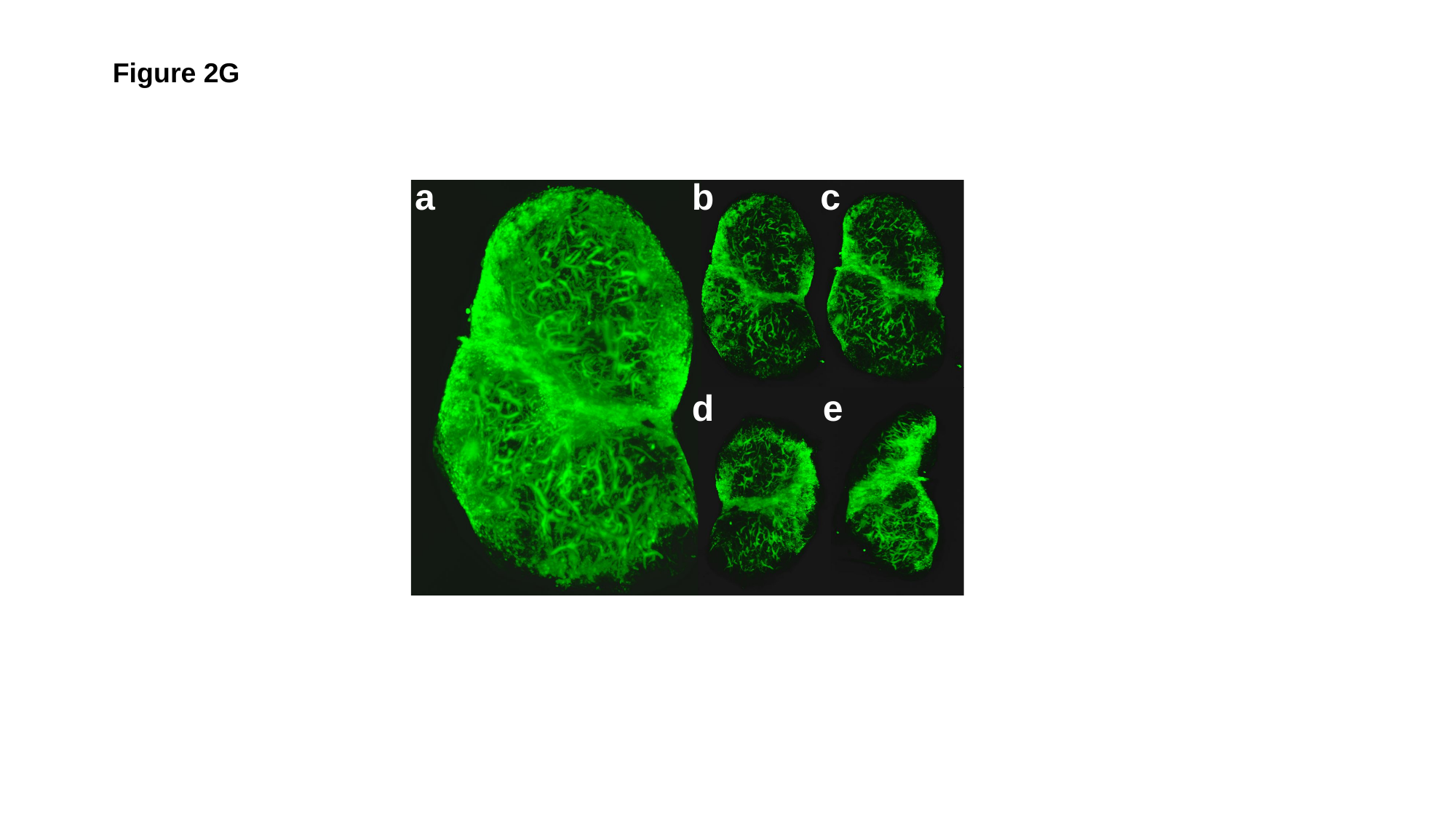

Figure 2G
a
b
c
d
e

Supplement: Supplementary file 5 — Source data Fig. 2 [file 44321_2024_76_MOESM5_ESM.zip › Figure 2G/Figure 2G.pptx]

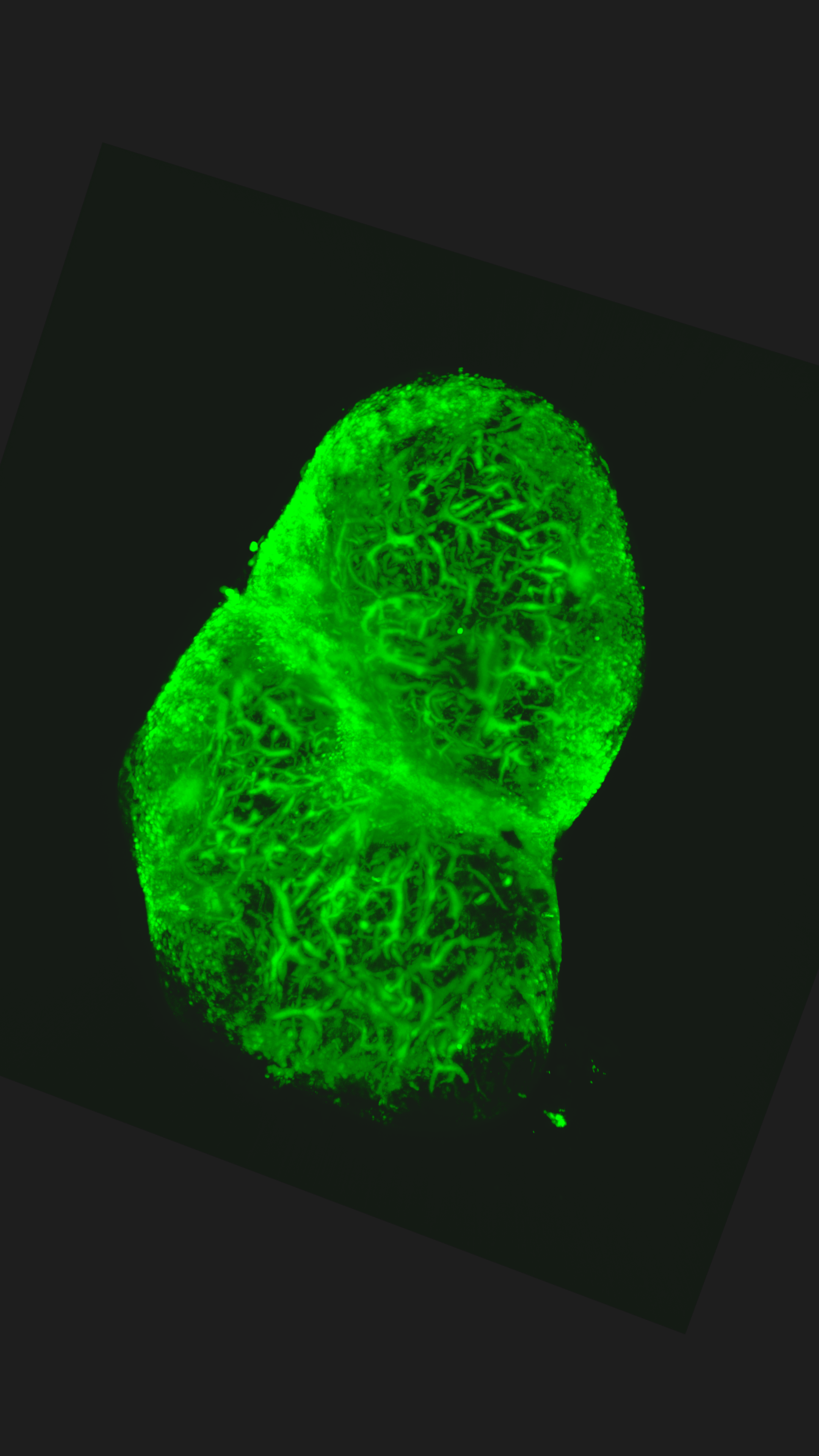

Supplement: Supplementary file 5 — Source data Fig. 2 [file 44321_2024_76_MOESM5_ESM.zip › Figure 2G/Inguinal lymph node/a.png]

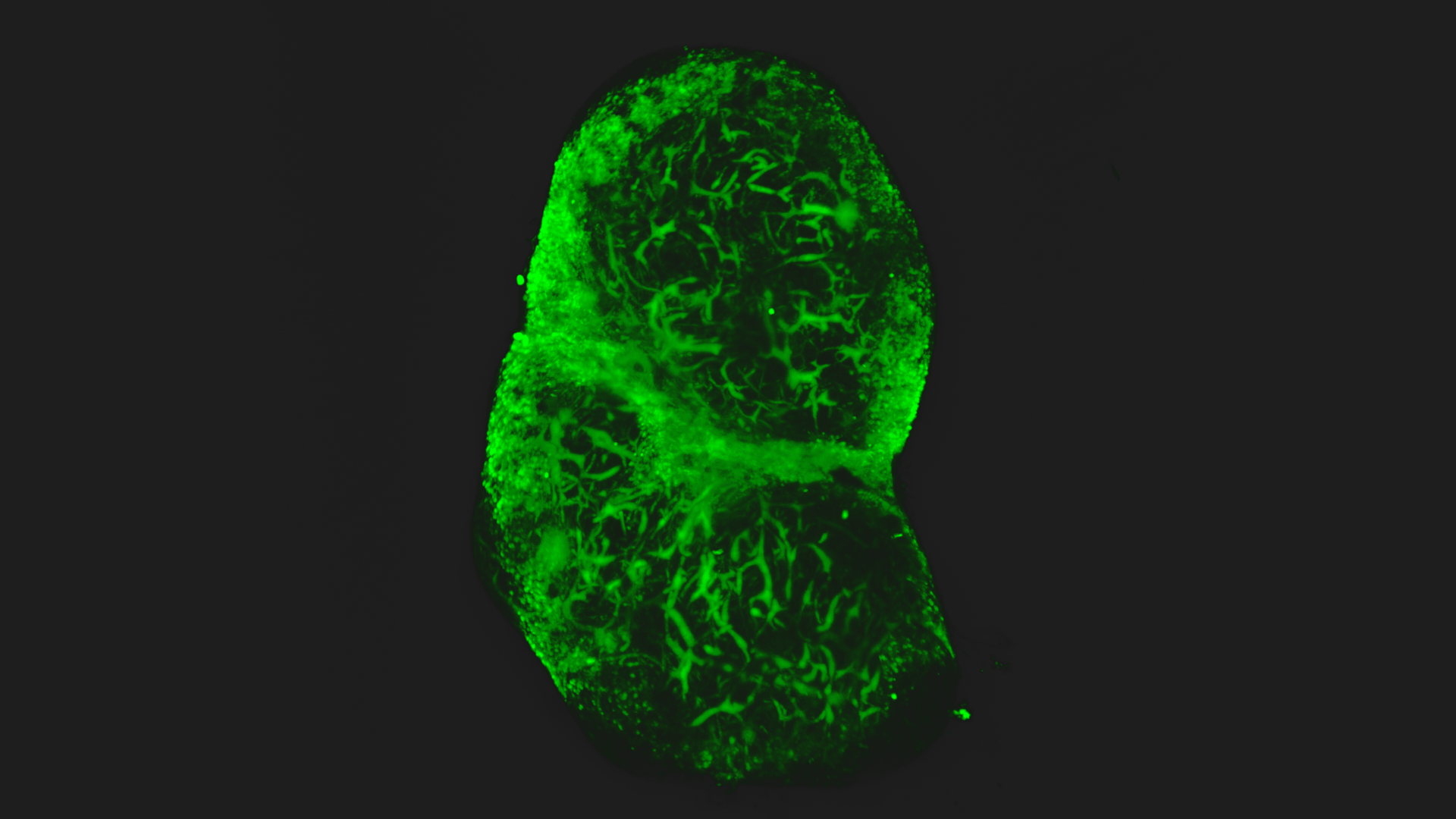

Supplement: Supplementary file 5 — Source data Fig. 2 [file 44321_2024_76_MOESM5_ESM.zip › Figure 2G/Inguinal lymph node/b.png]

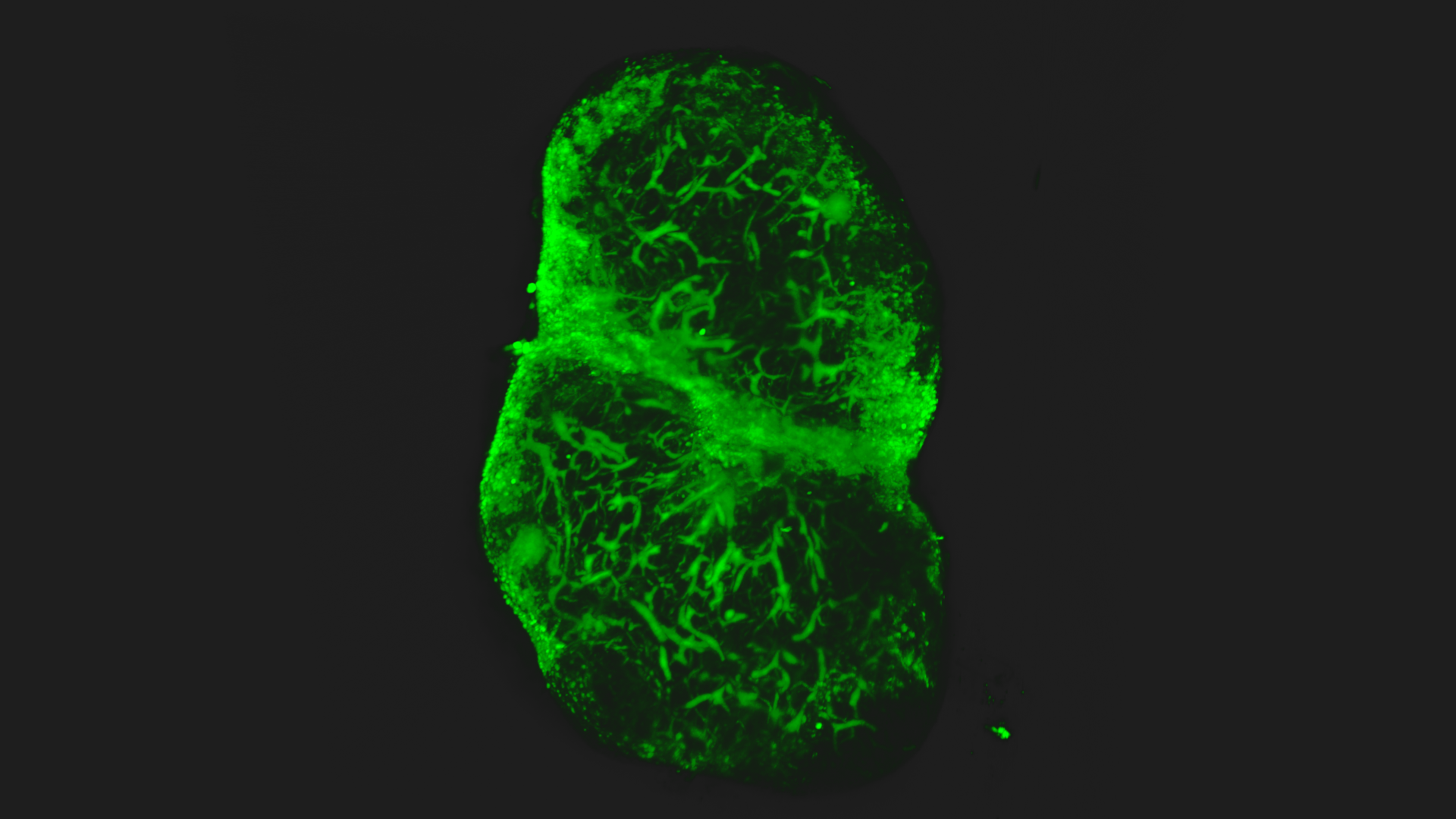

Supplement: Supplementary file 5 — Source data Fig. 2 [file 44321_2024_76_MOESM5_ESM.zip › Figure 2G/Inguinal lymph node/c.png]

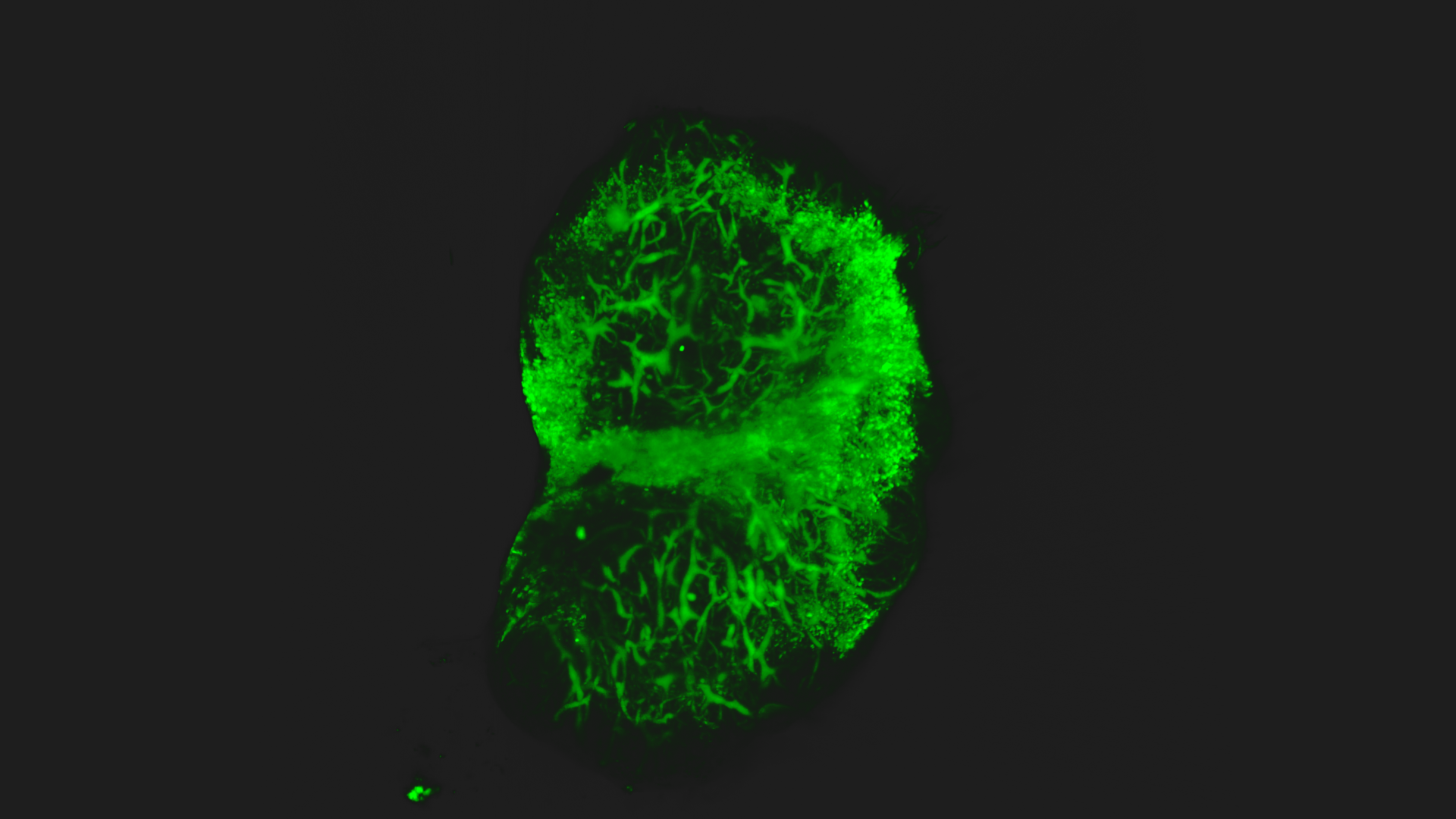

Supplement: Supplementary file 5 — Source data Fig. 2 [file 44321_2024_76_MOESM5_ESM.zip › Figure 2G/Inguinal lymph node/d.png]

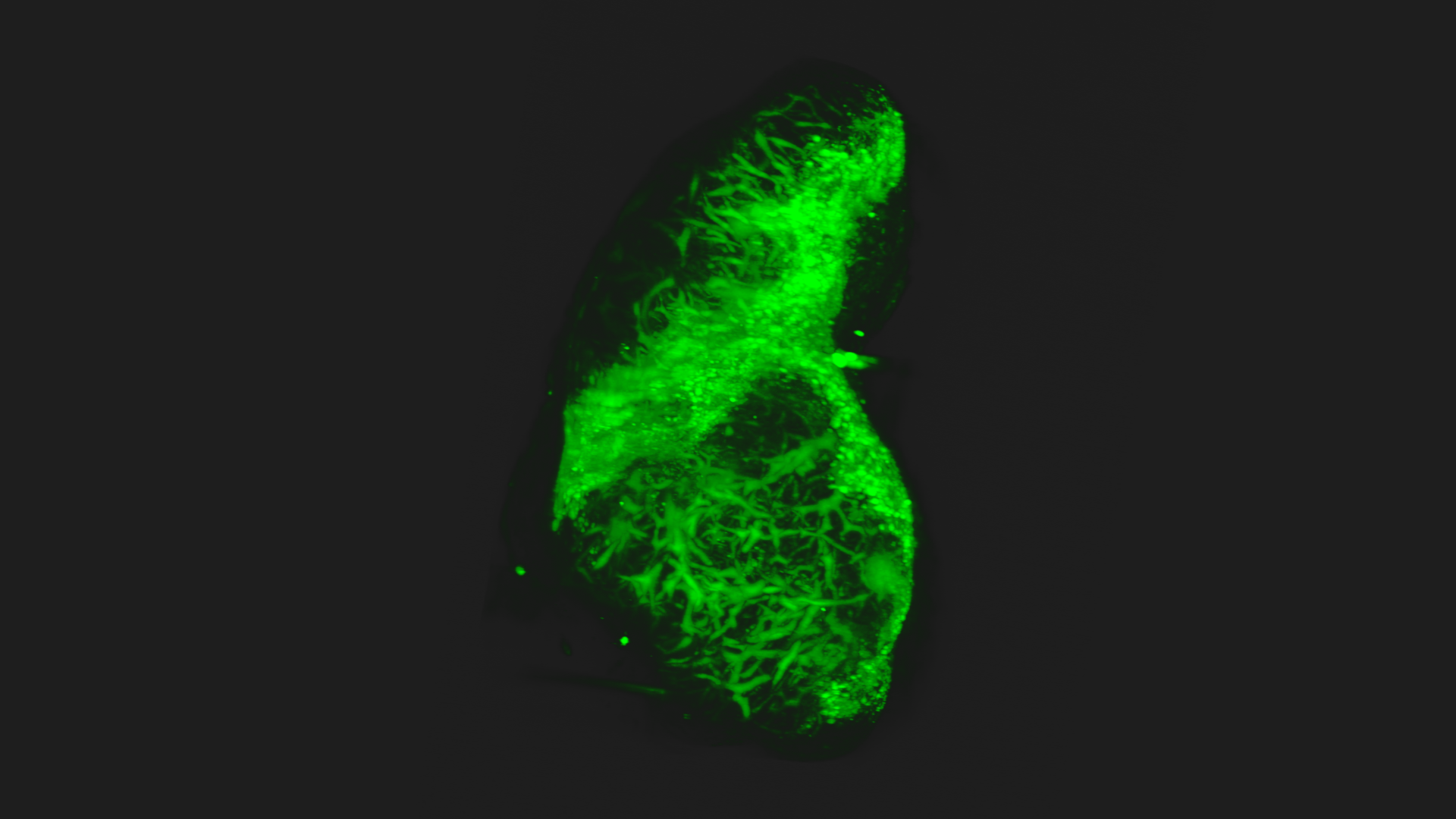

Supplement: Supplementary file 5 — Source data Fig. 2 [file 44321_2024_76_MOESM5_ESM.zip › Figure 2G/Inguinal lymph node/e.png]

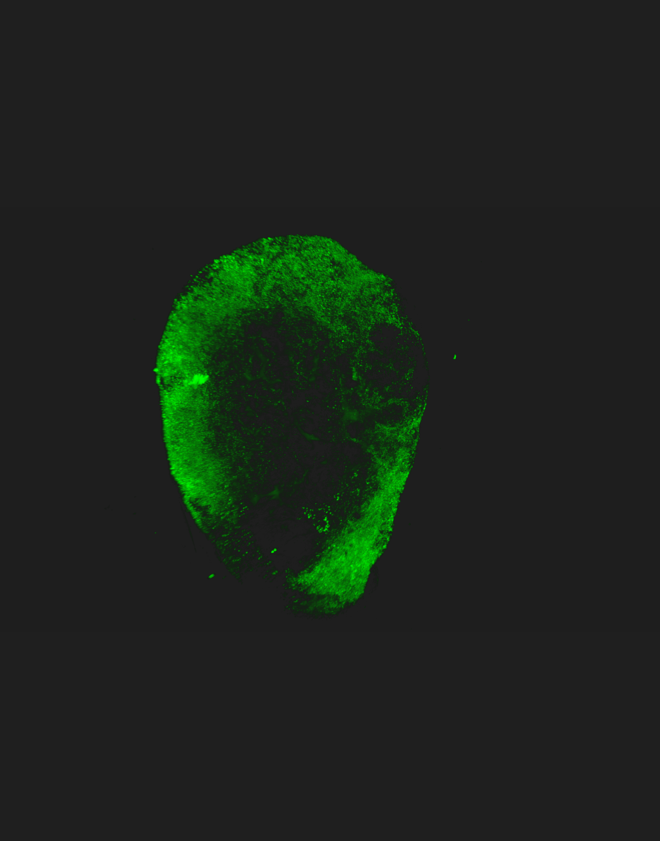

Supplement: Supplementary file 5 — Source data Fig. 2 [file 44321_2024_76_MOESM5_ESM.zip › Figure 2H/Axillary lymph node/a.png]

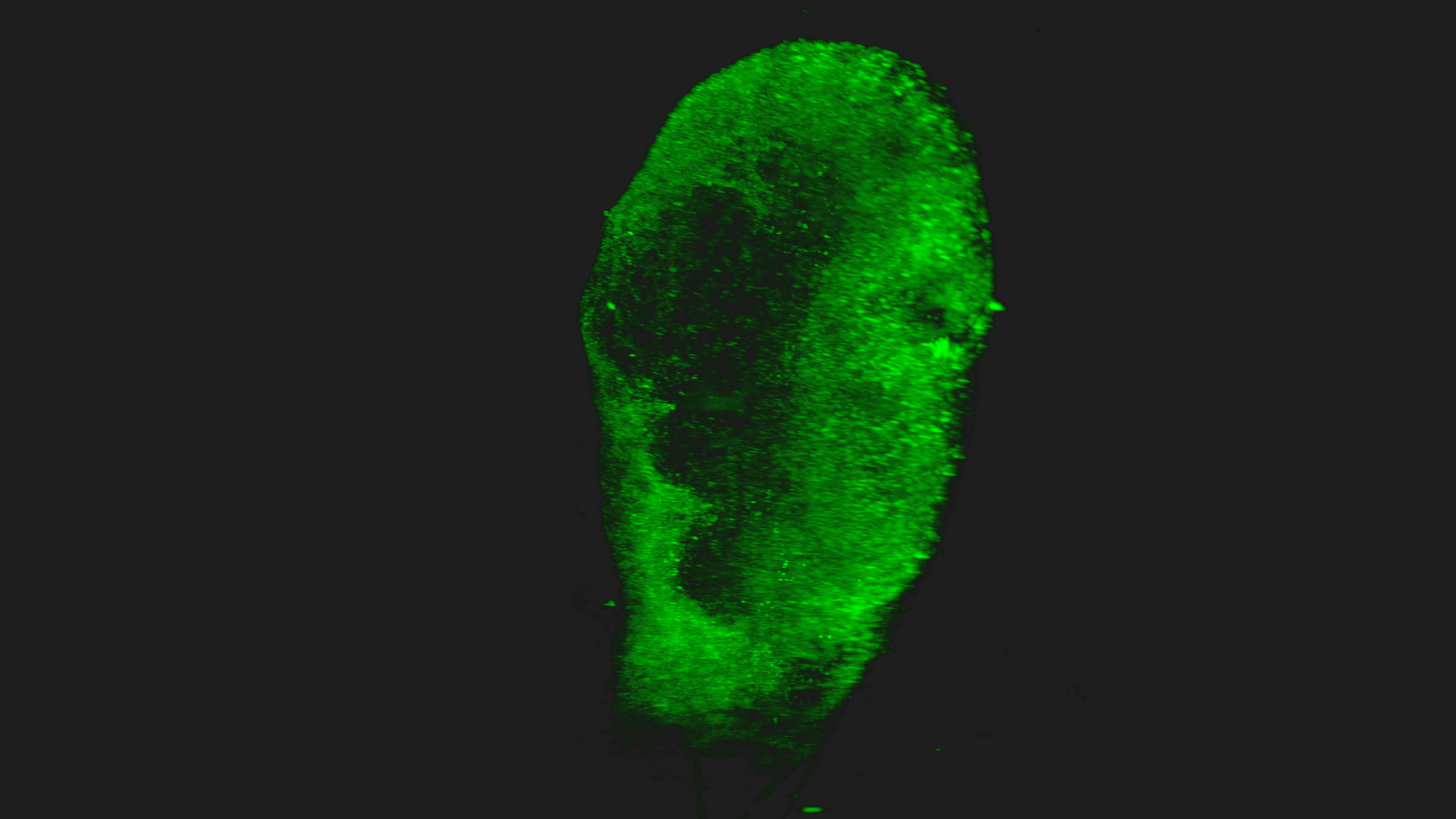

Supplement: Supplementary file 5 — Source data Fig. 2 [file 44321_2024_76_MOESM5_ESM.zip › Figure 2H/Axillary lymph node/b.png]

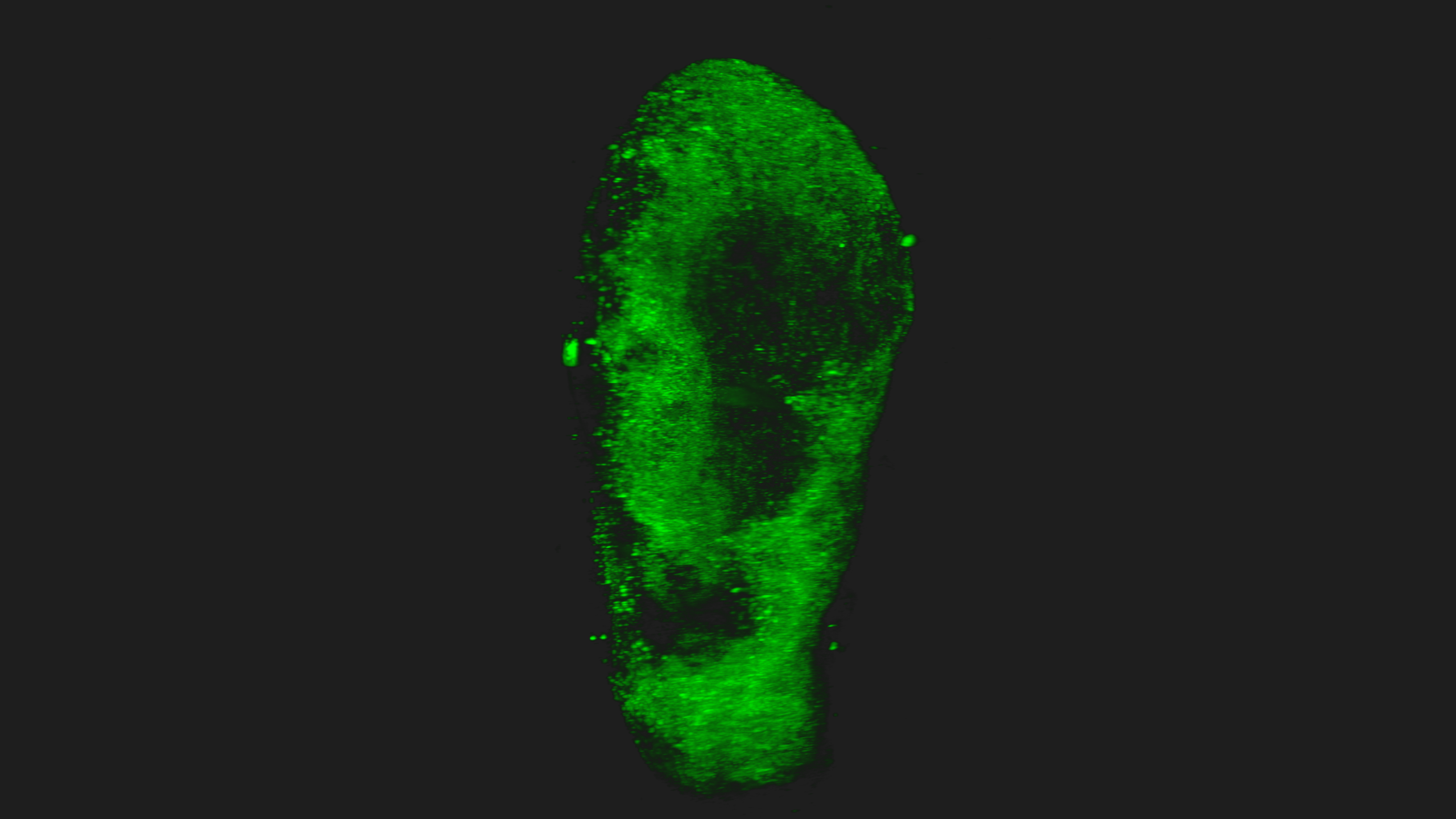

Supplement: Supplementary file 5 — Source data Fig. 2 [file 44321_2024_76_MOESM5_ESM.zip › Figure 2H/Axillary lymph node/c.png]

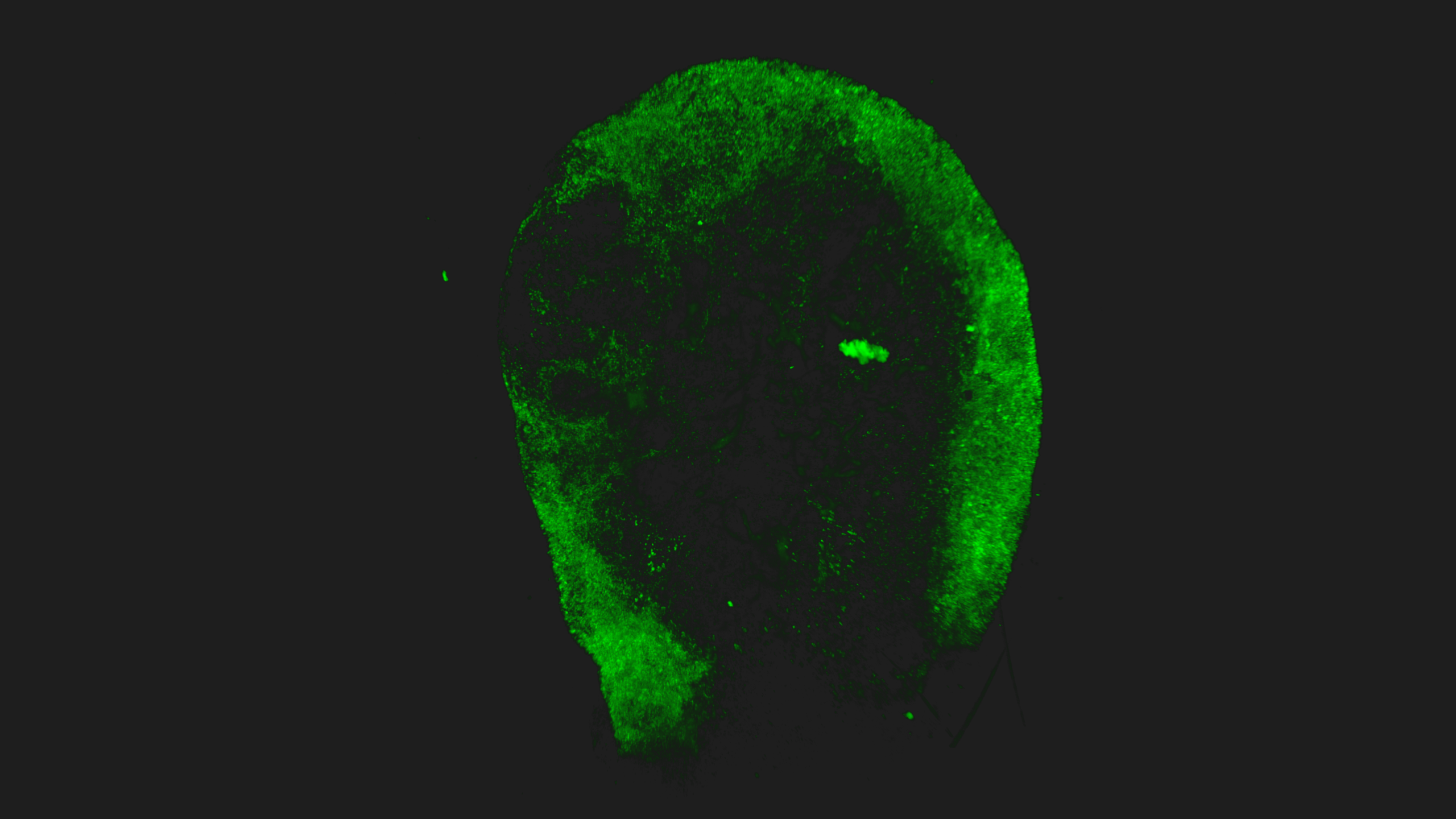

Supplement: Supplementary file 5 — Source data Fig. 2 [file 44321_2024_76_MOESM5_ESM.zip › Figure 2H/Axillary lymph node/d.png]

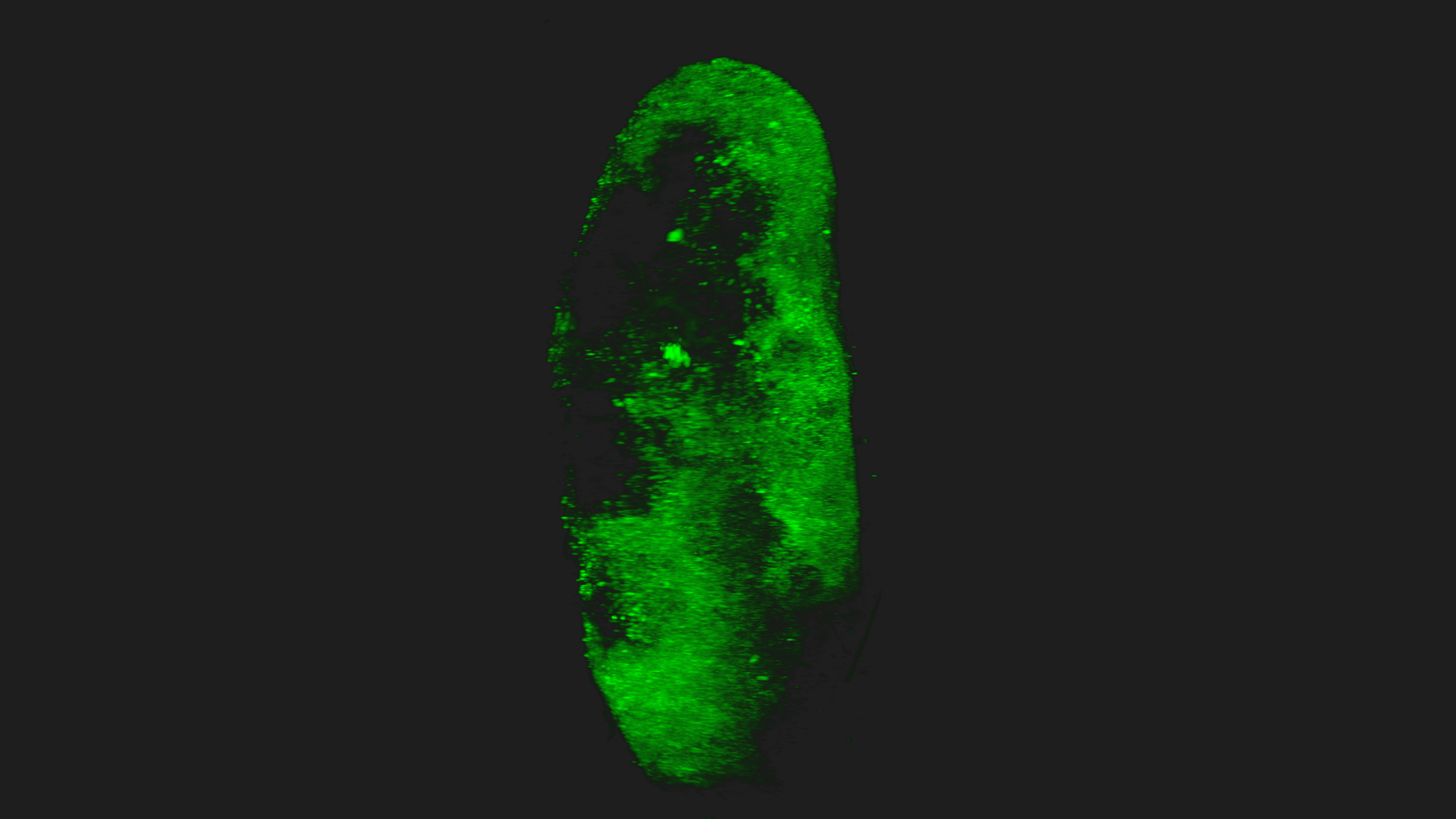

Supplement: Supplementary file 5 — Source data Fig. 2 [file 44321_2024_76_MOESM5_ESM.zip › Figure 2H/Axillary lymph node/e.png]

## Slide 1
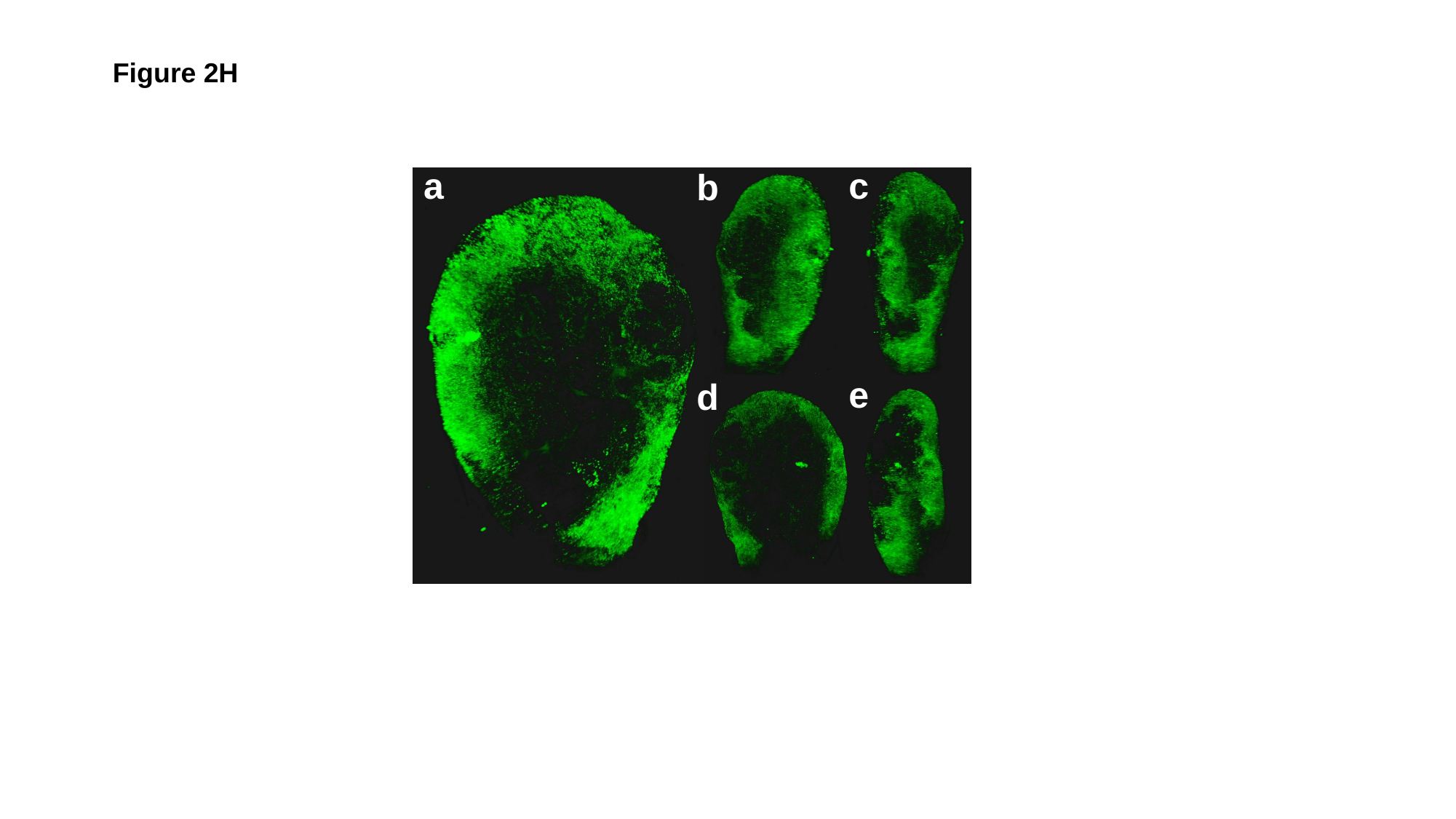

Figure 2H
a
c
b
e
d

Supplement: Supplementary file 5 — Source data Fig. 2 [file 44321_2024_76_MOESM5_ESM.zip › Figure 2H/Figure 2H.pptx]

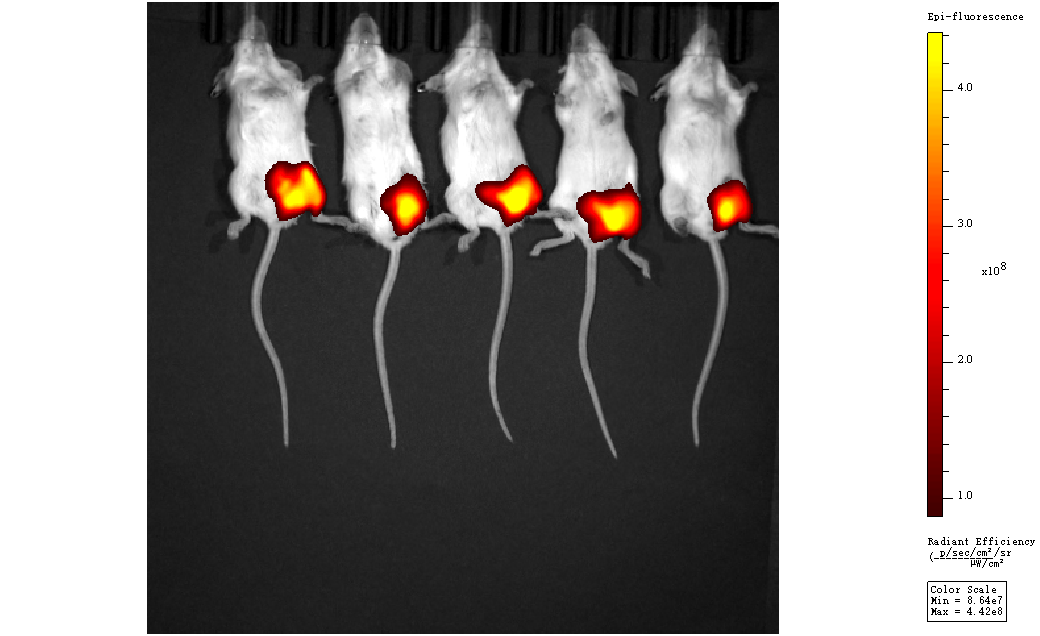

Supplement: Supplementary file 5 — Source data Fig. 2 [file 44321_2024_76_MOESM5_ESM.zip › Figure 2I/His-HA-NPs/0 d.png]

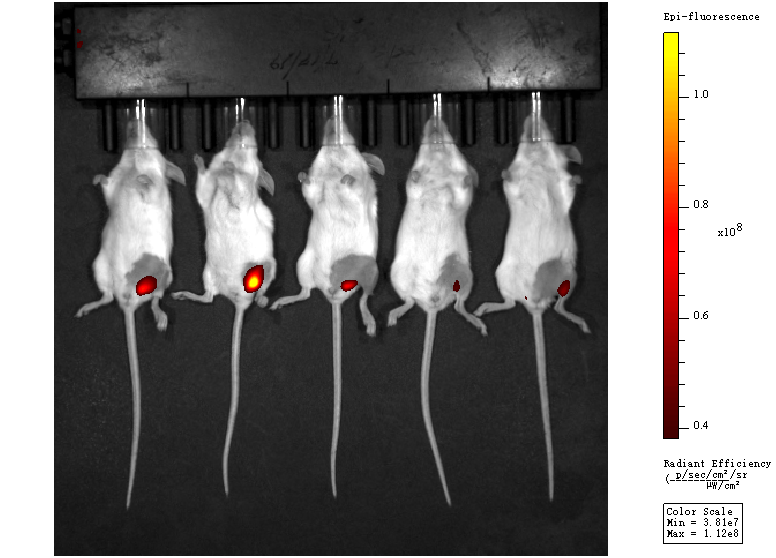

Supplement: Supplementary file 5 — Source data Fig. 2 [file 44321_2024_76_MOESM5_ESM.zip › Figure 2I/His-HA-NPs/12 d.png]

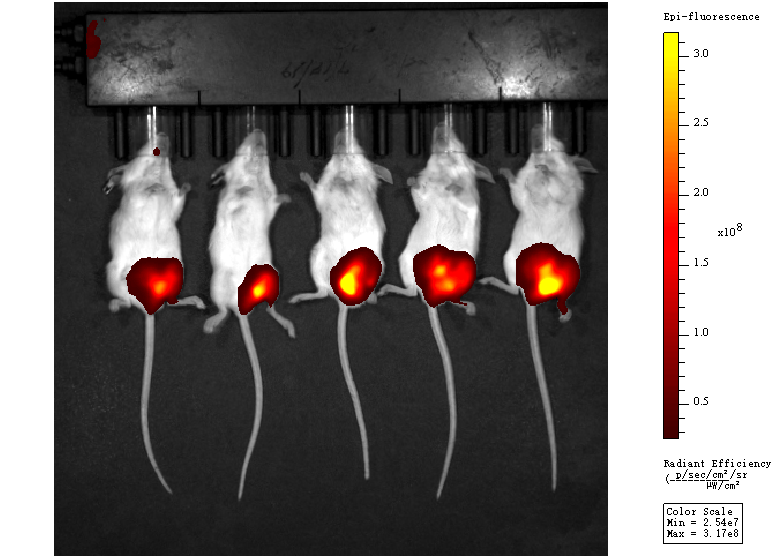

Supplement: Supplementary file 5 — Source data Fig. 2 [file 44321_2024_76_MOESM5_ESM.zip › Figure 2I/His-HA-NPs/3 d.png]

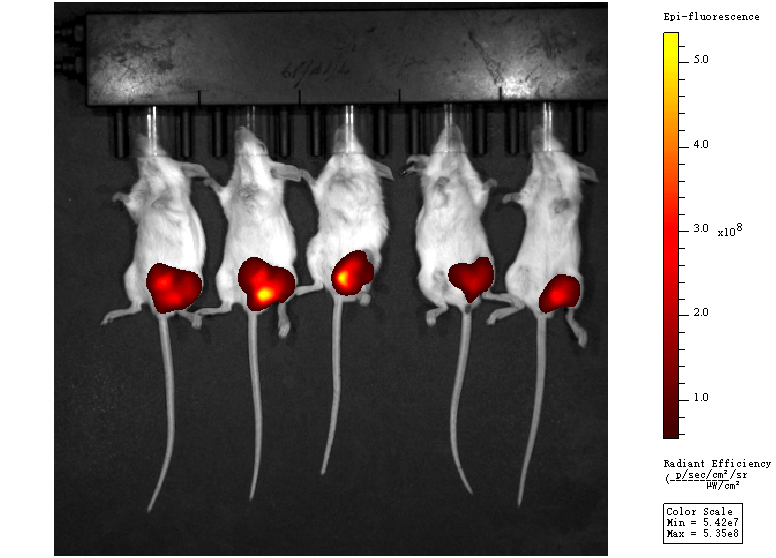

Supplement: Supplementary file 5 — Source data Fig. 2 [file 44321_2024_76_MOESM5_ESM.zip › Figure 2I/His-HA-NPs/6 d.png]

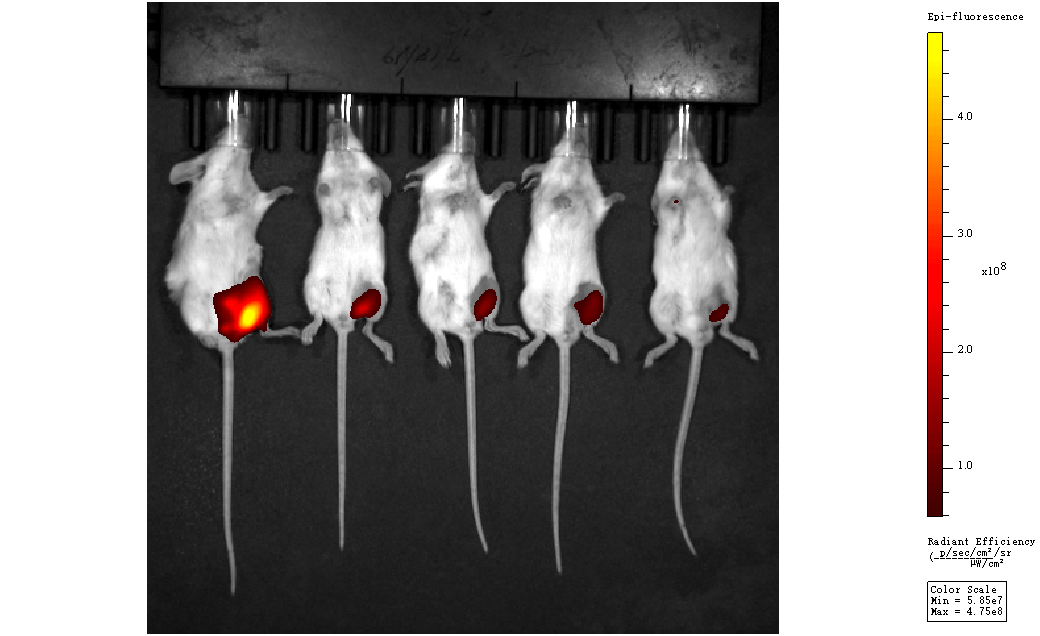

Supplement: Supplementary file 5 — Source data Fig. 2 [file 44321_2024_76_MOESM5_ESM.zip › Figure 2I/His-HA-NPs/9 d.png]

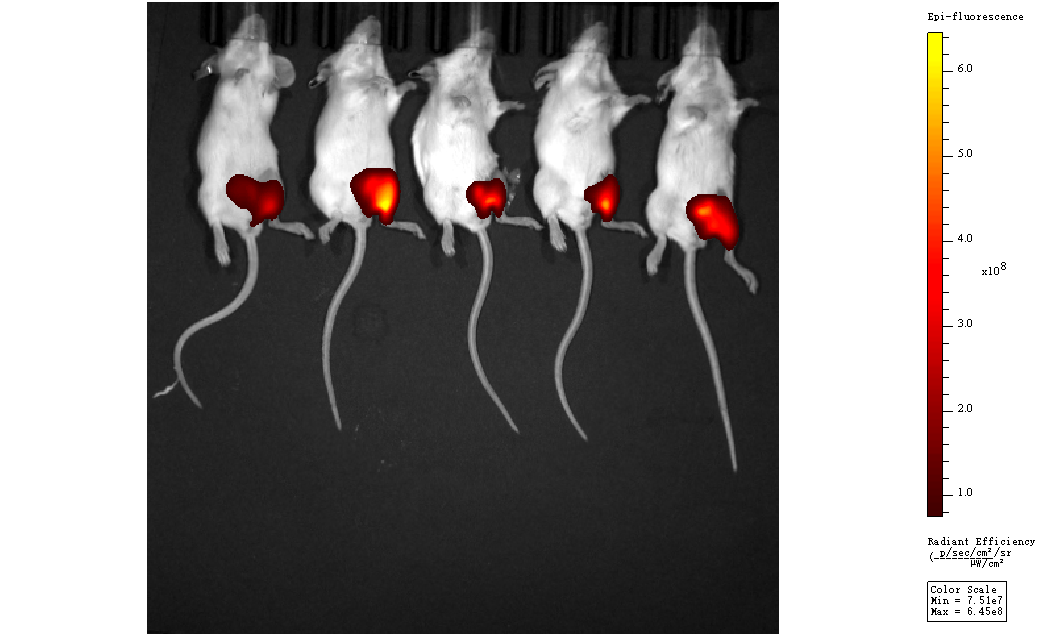

Supplement: Supplementary file 5 — Source data Fig. 2 [file 44321_2024_76_MOESM5_ESM.zip › Figure 2I/His-HA/0 d.png]

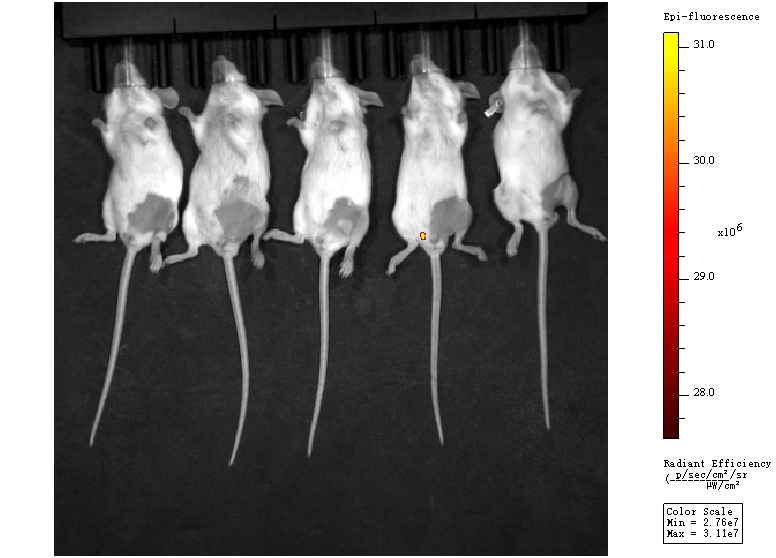

Supplement: Supplementary file 5 — Source data Fig. 2 [file 44321_2024_76_MOESM5_ESM.zip › Figure 2I/His-HA/12 d.png]

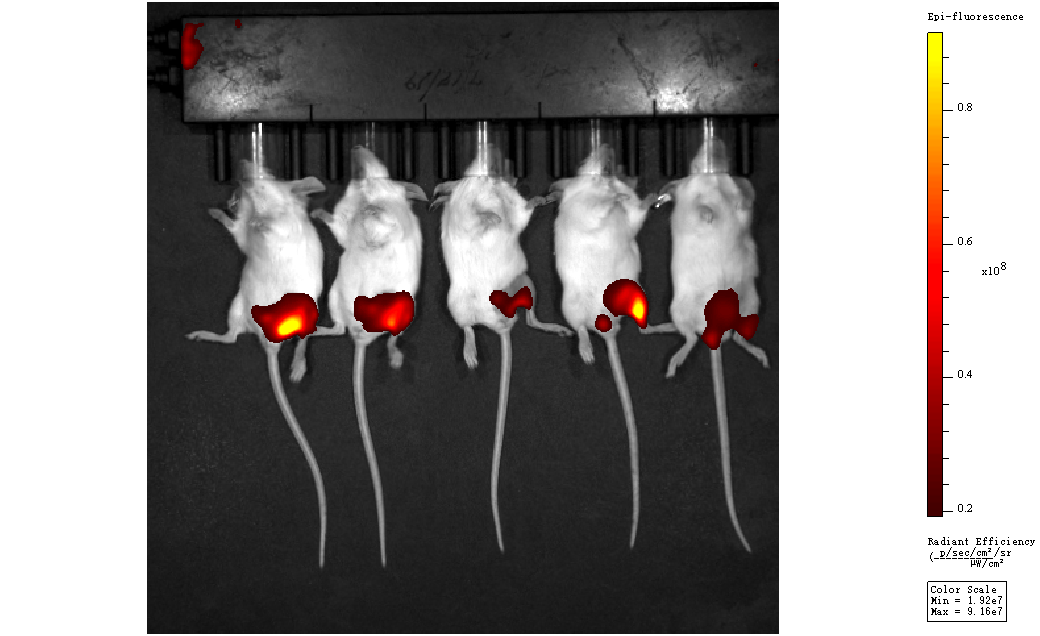

Supplement: Supplementary file 5 — Source data Fig. 2 [file 44321_2024_76_MOESM5_ESM.zip › Figure 2I/His-HA/3 d.png]

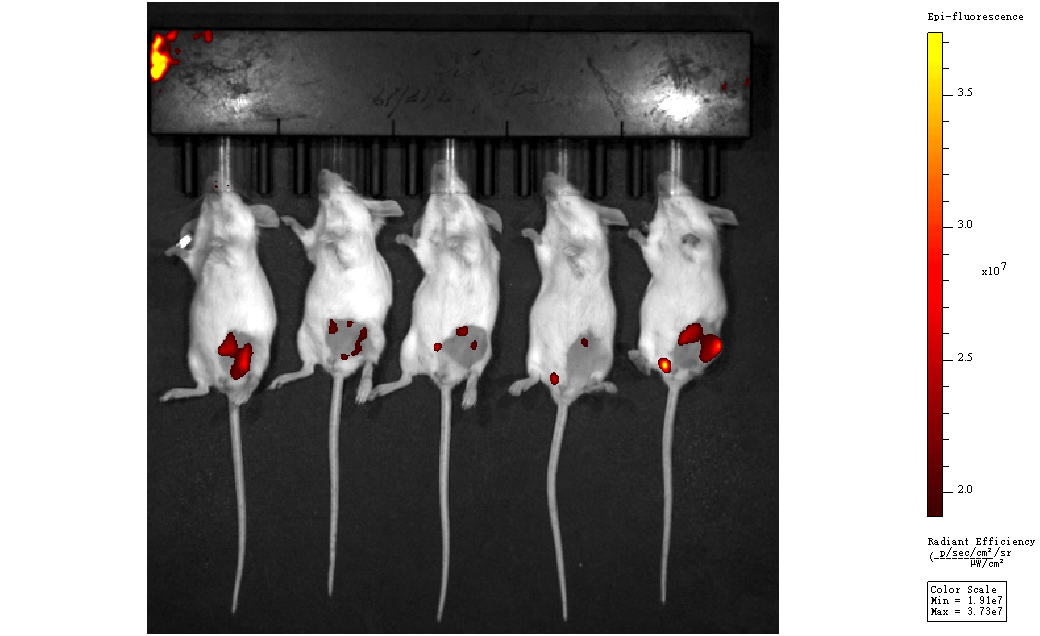

Supplement: Supplementary file 5 — Source data Fig. 2 [file 44321_2024_76_MOESM5_ESM.zip › Figure 2I/His-HA/6 d.png]

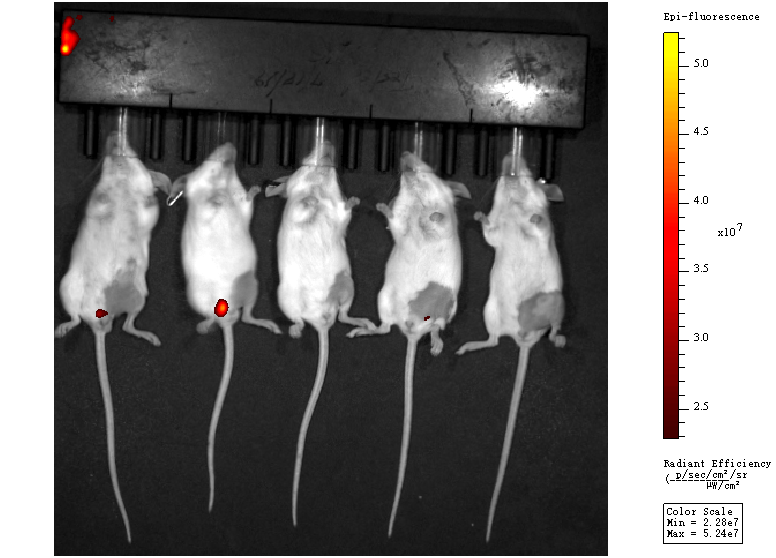

Supplement: Supplementary file 5 — Source data Fig. 2 [file 44321_2024_76_MOESM5_ESM.zip › Figure 2I/His-HA/9 d.png]

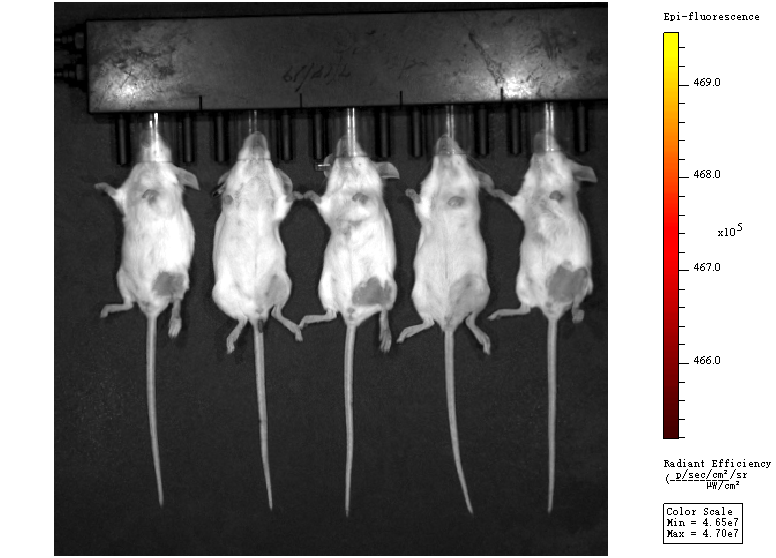

Supplement: Supplementary file 5 — Source data Fig. 2 [file 44321_2024_76_MOESM5_ESM.zip › Figure 2I/Mock/0 d.png]

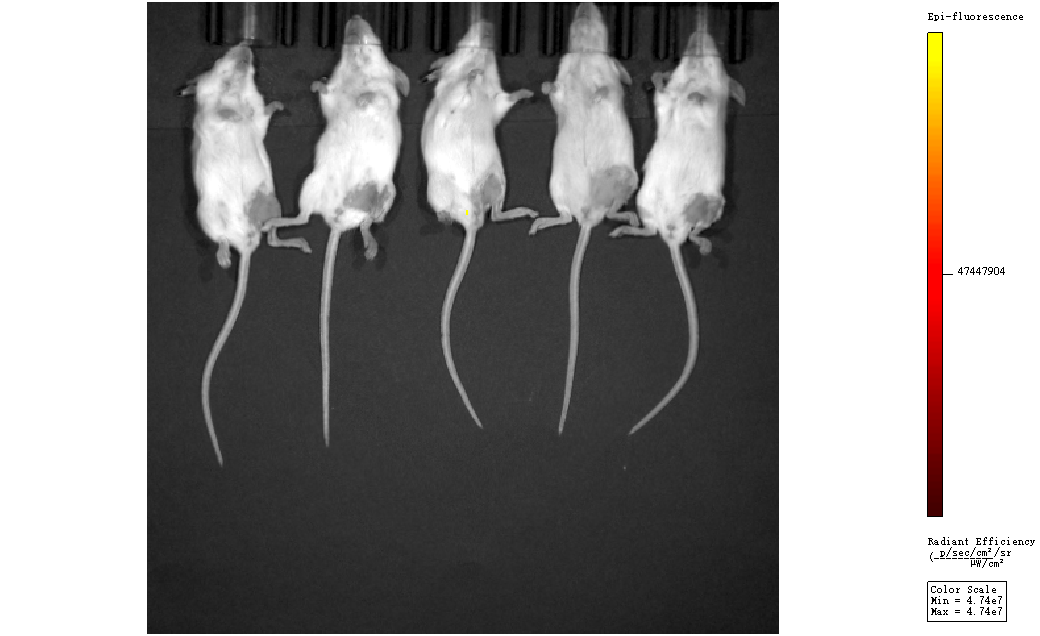

Supplement: Supplementary file 5 — Source data Fig. 2 [file 44321_2024_76_MOESM5_ESM.zip › Figure 2I/Mock/12 d.png]

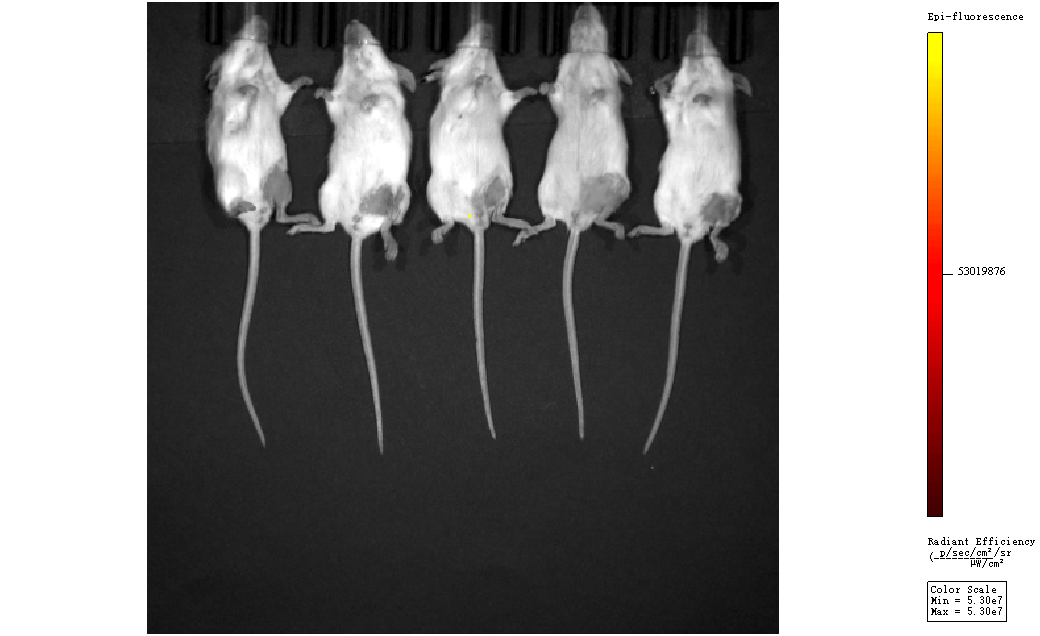

Supplement: Supplementary file 5 — Source data Fig. 2 [file 44321_2024_76_MOESM5_ESM.zip › Figure 2I/Mock/3 d.png]

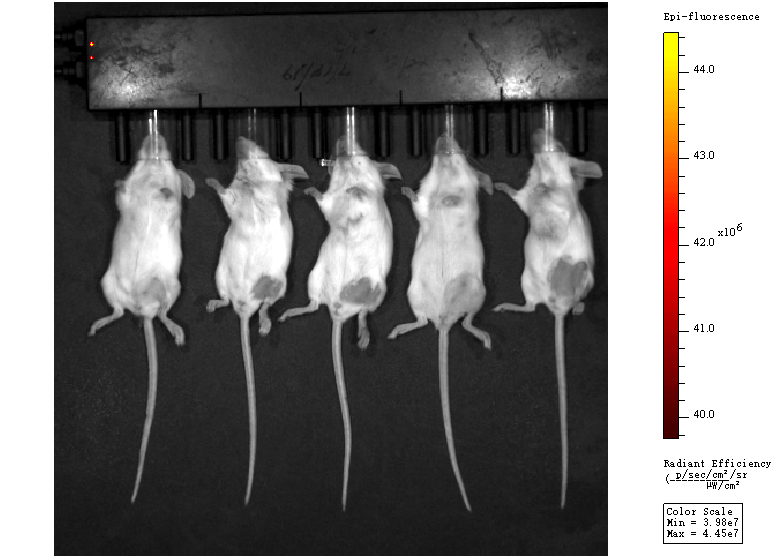

Supplement: Supplementary file 5 — Source data Fig. 2 [file 44321_2024_76_MOESM5_ESM.zip › Figure 2I/Mock/6 d.png]

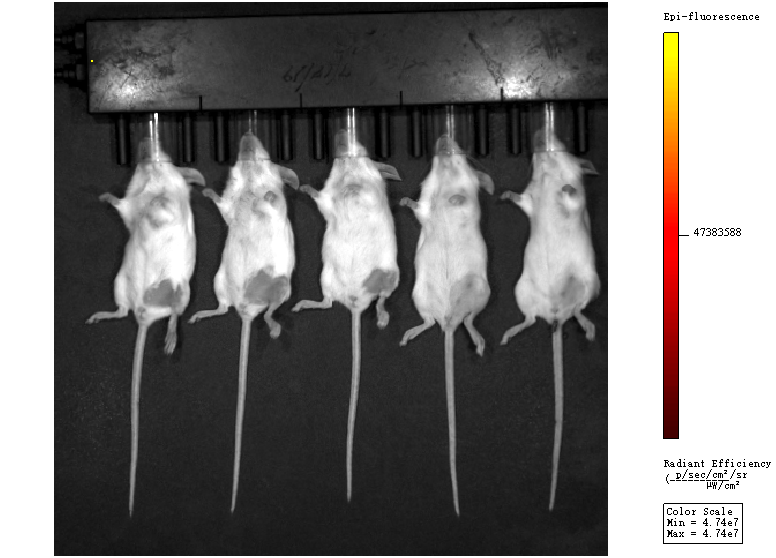

Supplement: Supplementary file 5 — Source data Fig. 2 [file 44321_2024_76_MOESM5_ESM.zip › Figure 2I/Mock/9 d.png]

## Slide 1
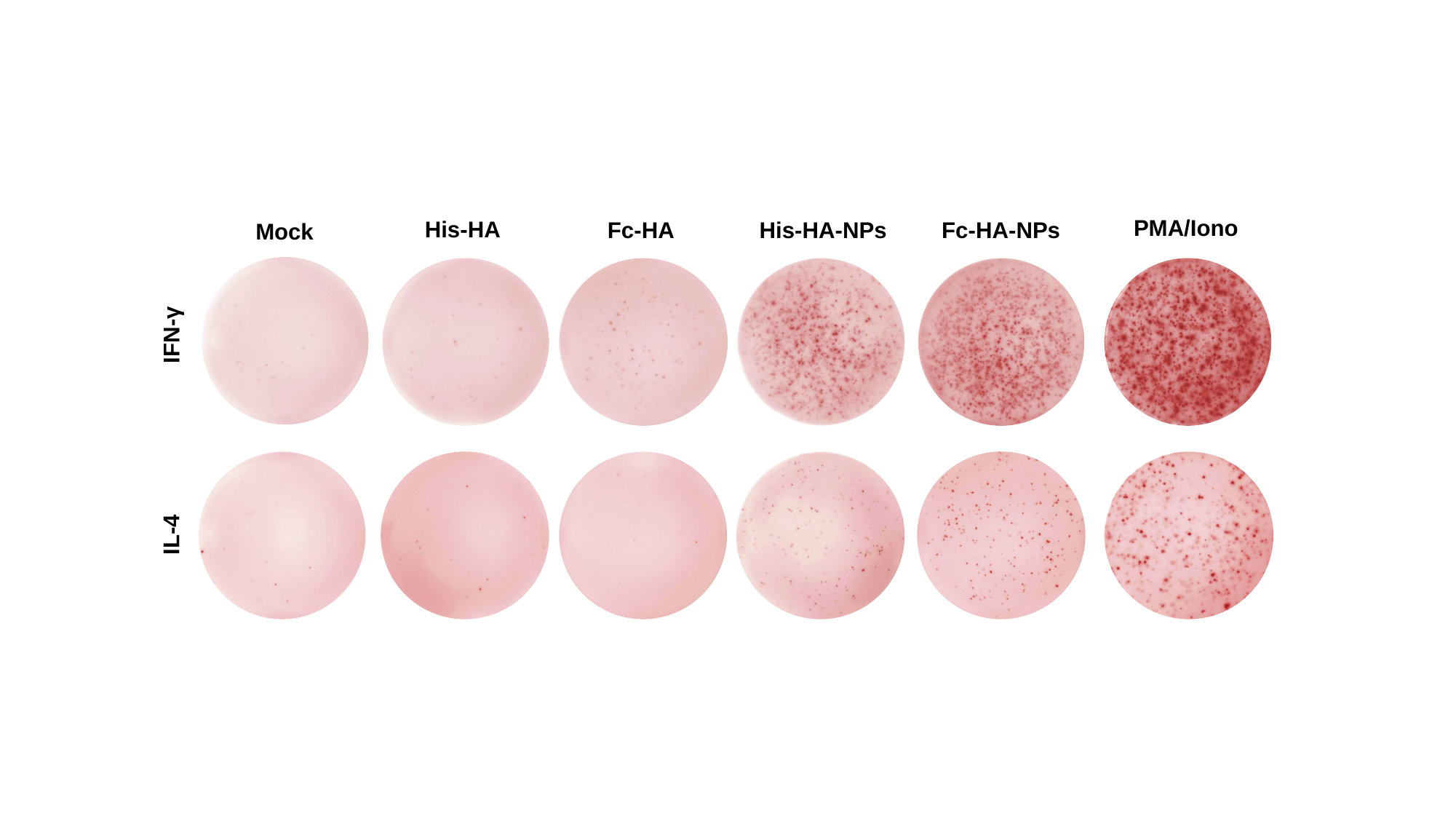

PMA/Iono
His-HA
His-HA-NPs
Fc-HA
Fc-HA-NPs
Mock
IFN-γ
IL-4

Supplement: Supplementary file 7 — Source data Fig. 4 [file 44321_2024_76_MOESM7_ESM.zip › Figure 4F/Figure 4F.pptx]

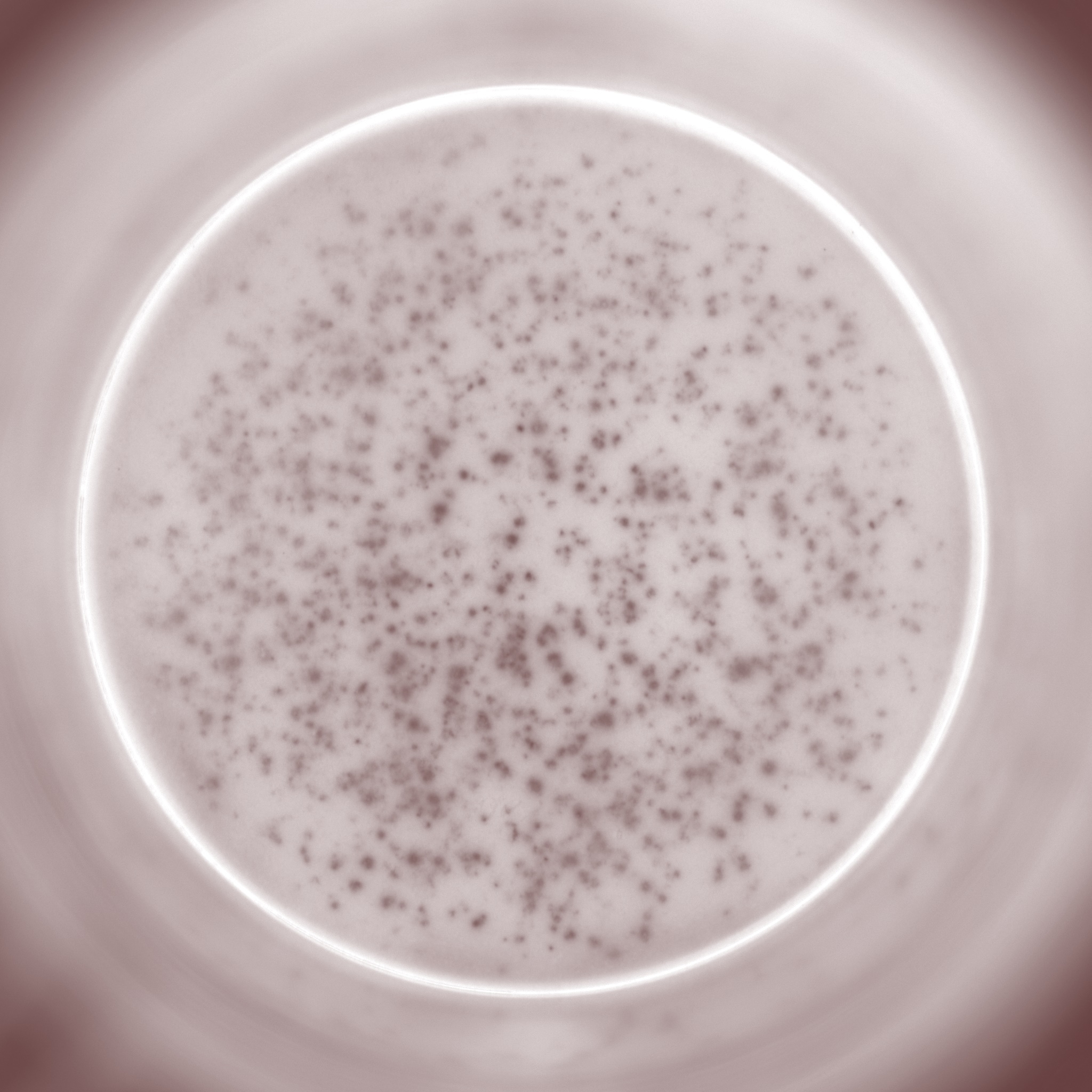

Supplement: Supplementary file 7 — Source data Fig. 4 [file 44321_2024_76_MOESM7_ESM.zip › Figure 4F/IFN-γ/Fc-HA-NPs.jpg]

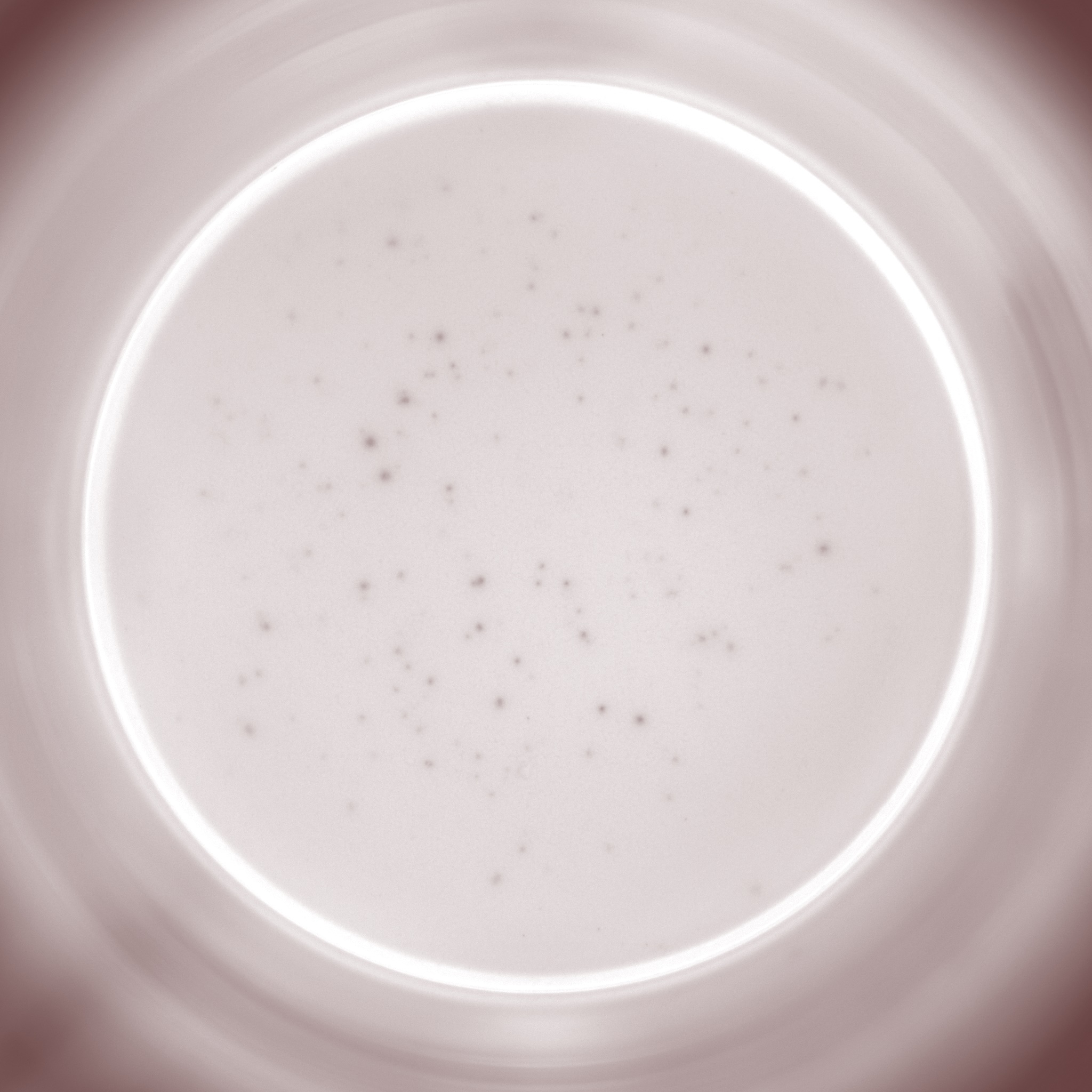

Supplement: Supplementary file 7 — Source data Fig. 4 [file 44321_2024_76_MOESM7_ESM.zip › Figure 4F/IFN-γ/Fc-HA.jpg]

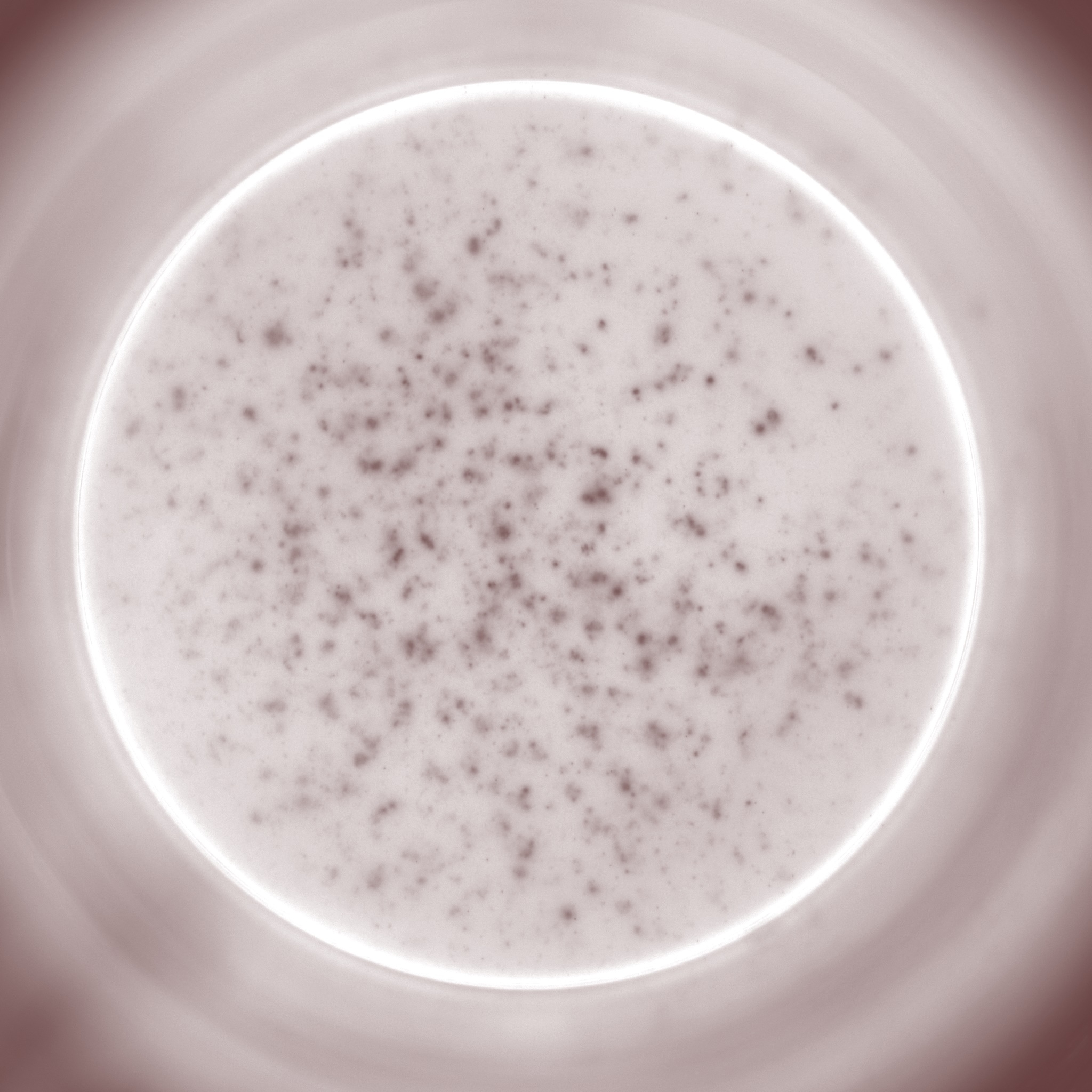

Supplement: Supplementary file 7 — Source data Fig. 4 [file 44321_2024_76_MOESM7_ESM.zip › Figure 4F/IFN-γ/His-HA-NPs.jpg]

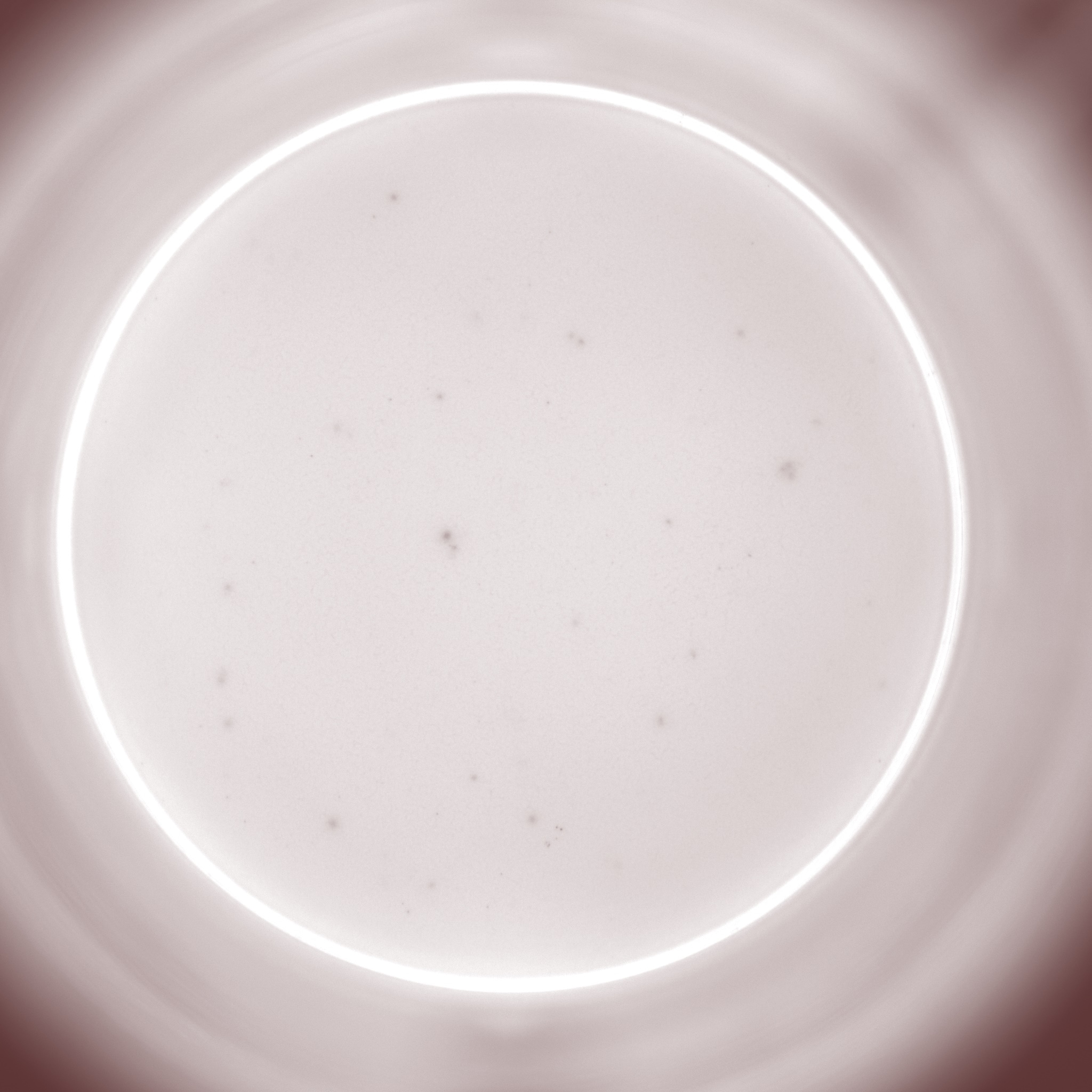

Supplement: Supplementary file 7 — Source data Fig. 4 [file 44321_2024_76_MOESM7_ESM.zip › Figure 4F/IFN-γ/His-HA.jpg]

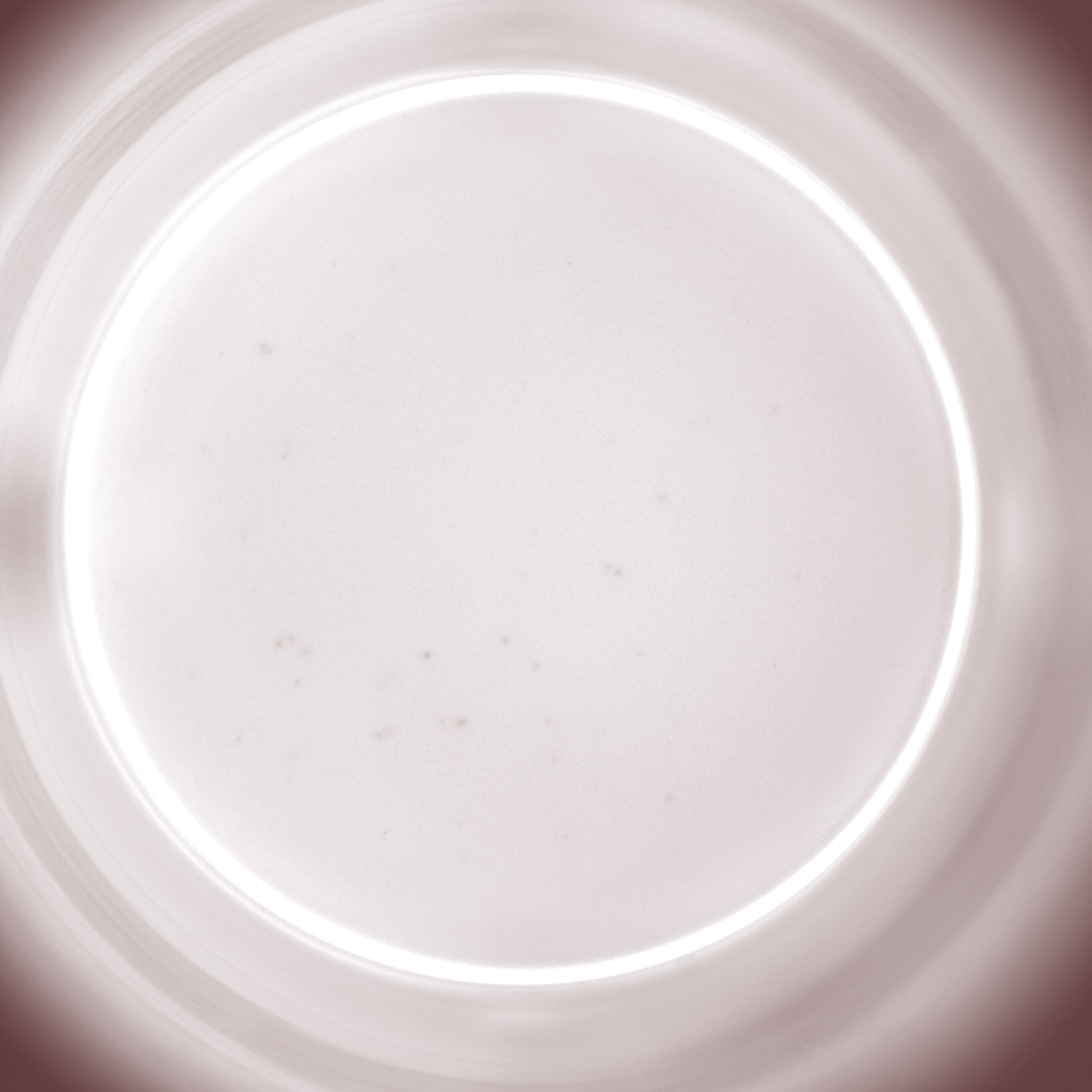

Supplement: Supplementary file 7 — Source data Fig. 4 [file 44321_2024_76_MOESM7_ESM.zip › Figure 4F/IFN-γ/Mock.jpg]

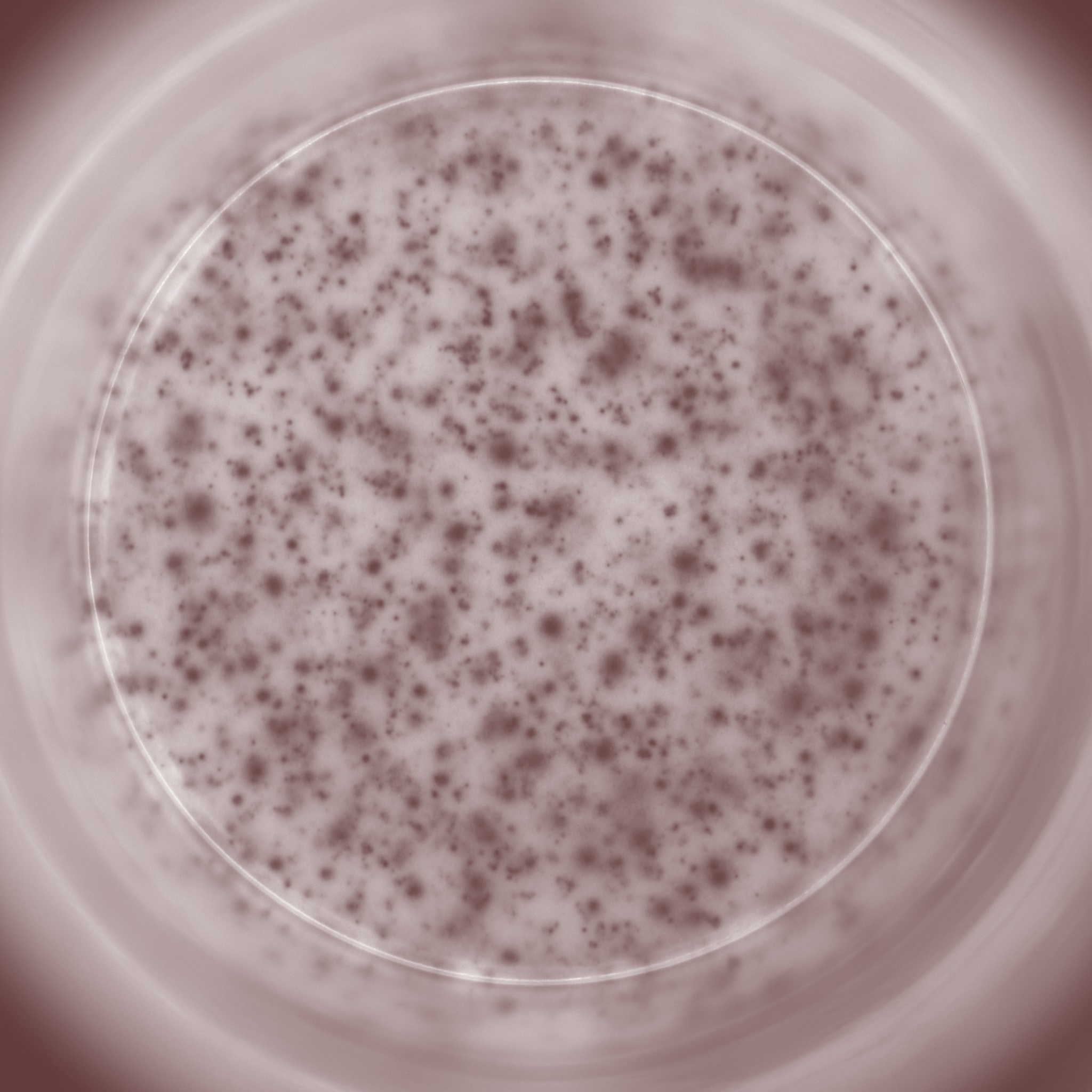

Supplement: Supplementary file 7 — Source data Fig. 4 [file 44321_2024_76_MOESM7_ESM.zip › Figure 4F/IFN-γ/PMAIono.jpg]

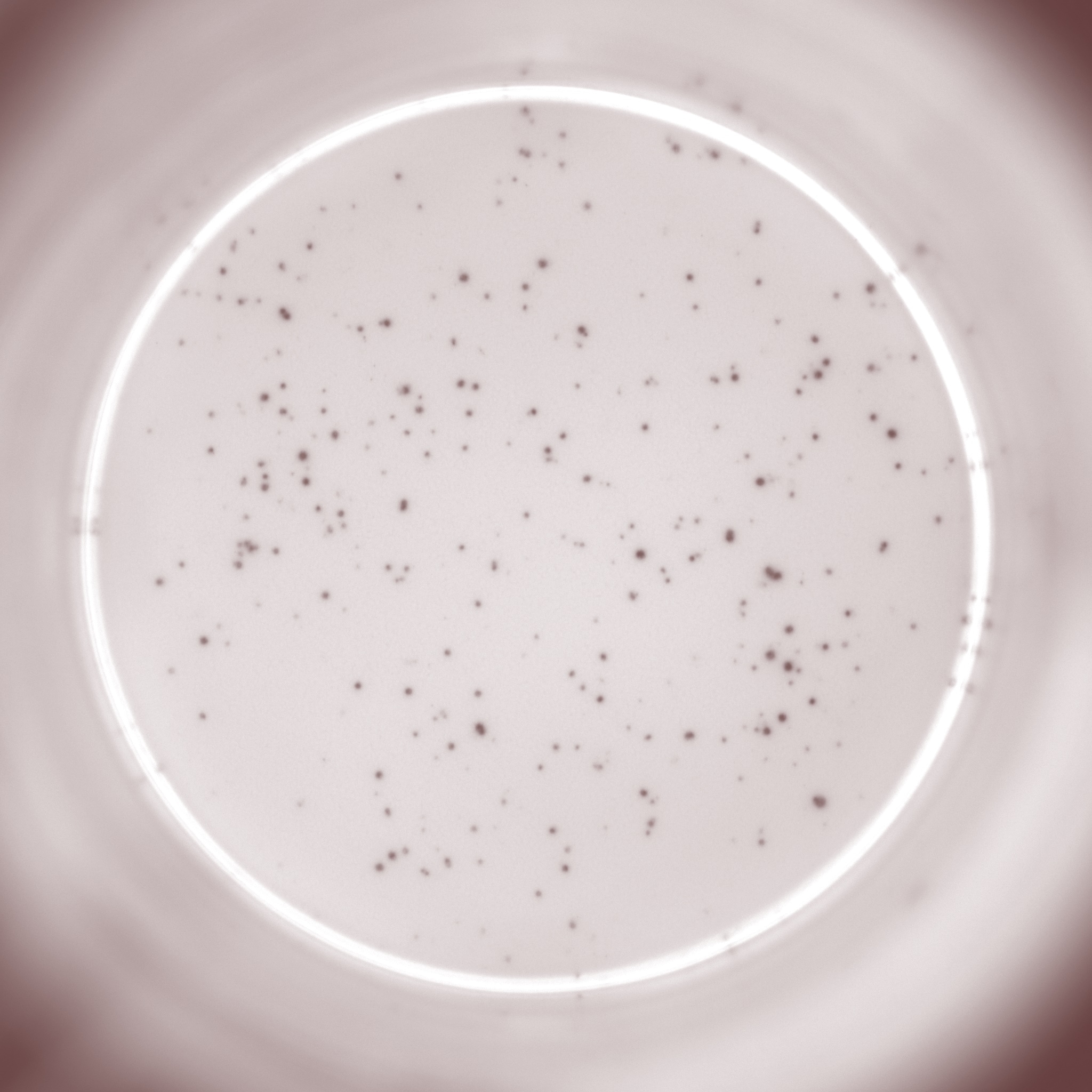

Supplement: Supplementary file 7 — Source data Fig. 4 [file 44321_2024_76_MOESM7_ESM.zip › Figure 4F/IL-4/Fc-HA-NPs.jpg]

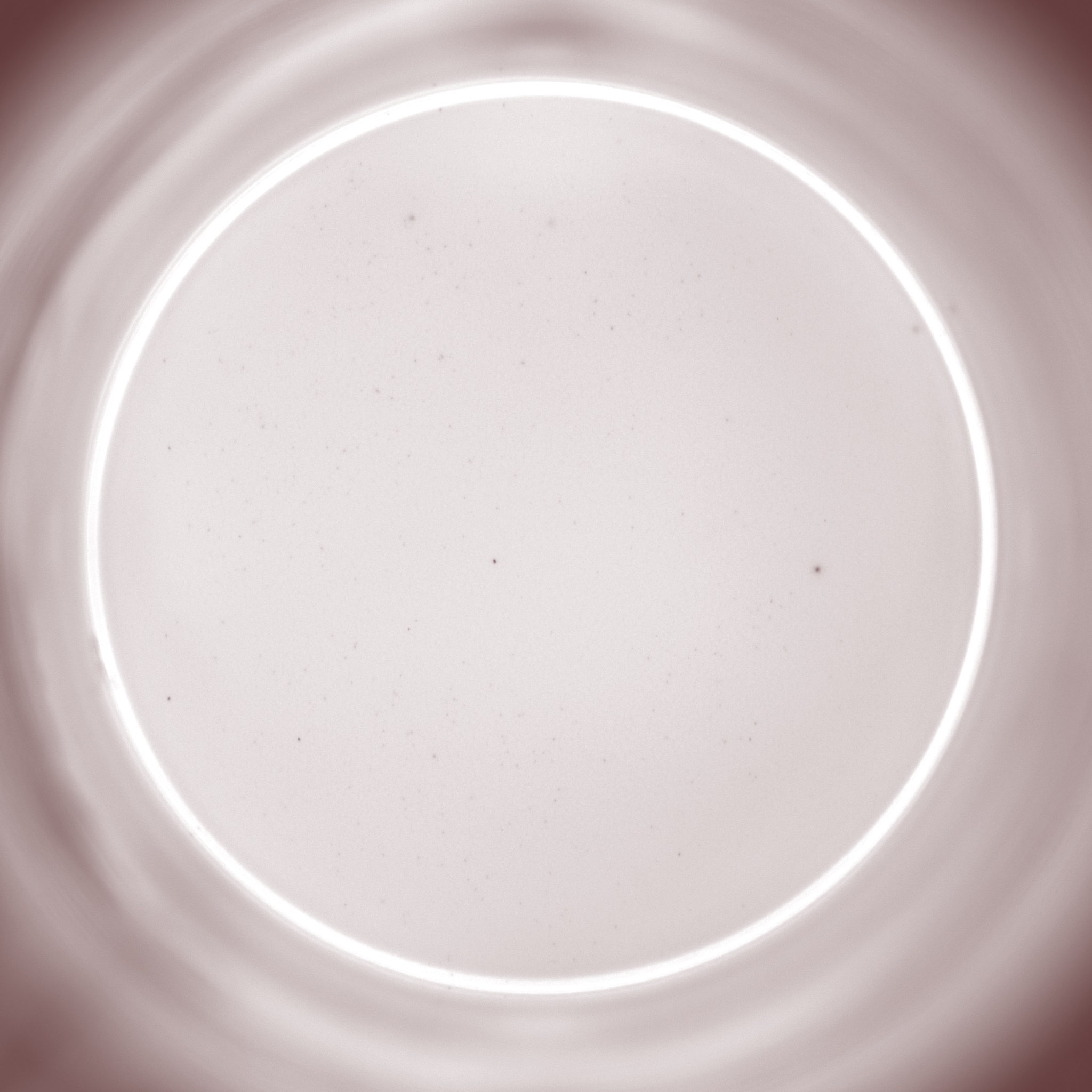

Supplement: Supplementary file 7 — Source data Fig. 4 [file 44321_2024_76_MOESM7_ESM.zip › Figure 4F/IL-4/Fc-HA.jpg]

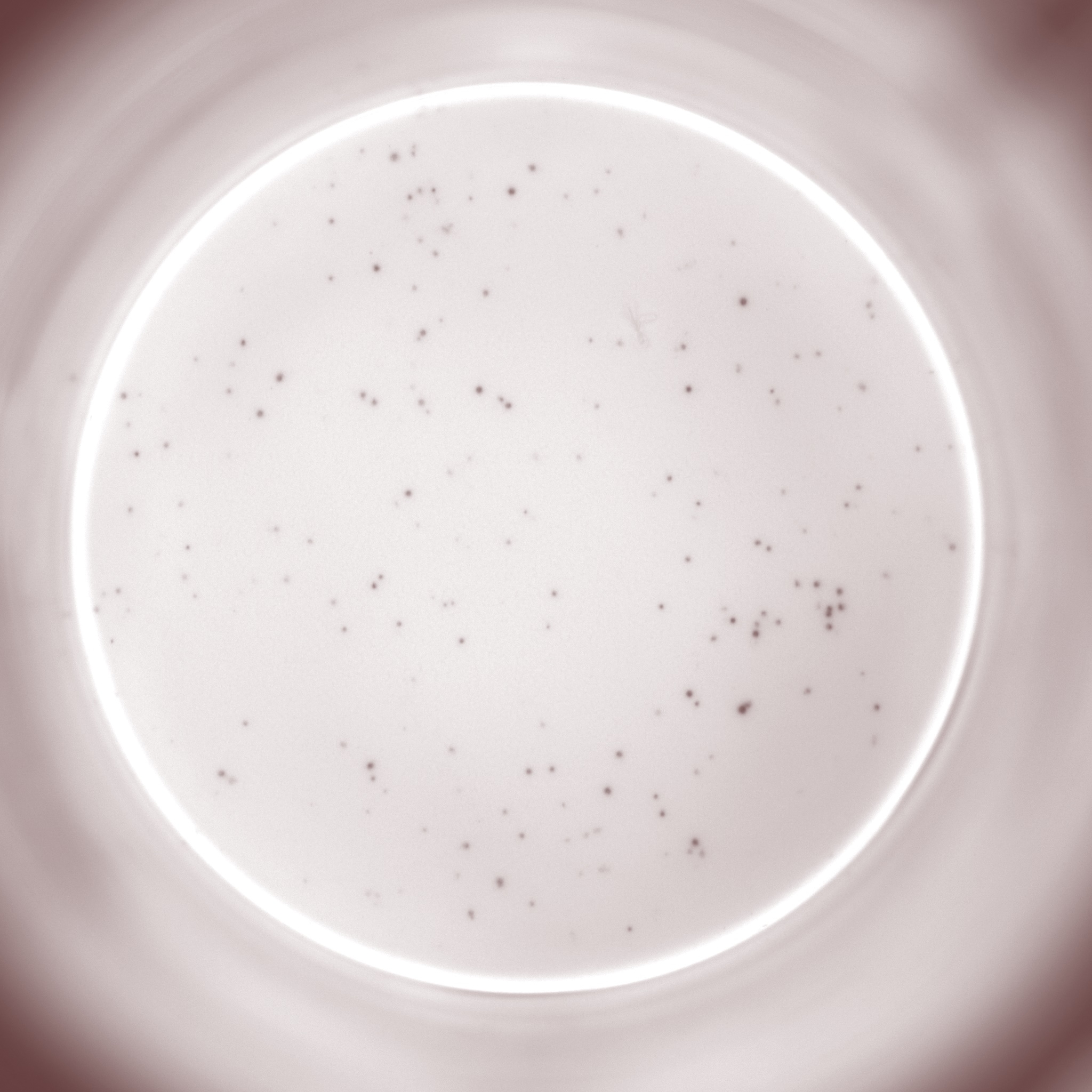

Supplement: Supplementary file 7 — Source data Fig. 4 [file 44321_2024_76_MOESM7_ESM.zip › Figure 4F/IL-4/His-HA-NPs.jpg]

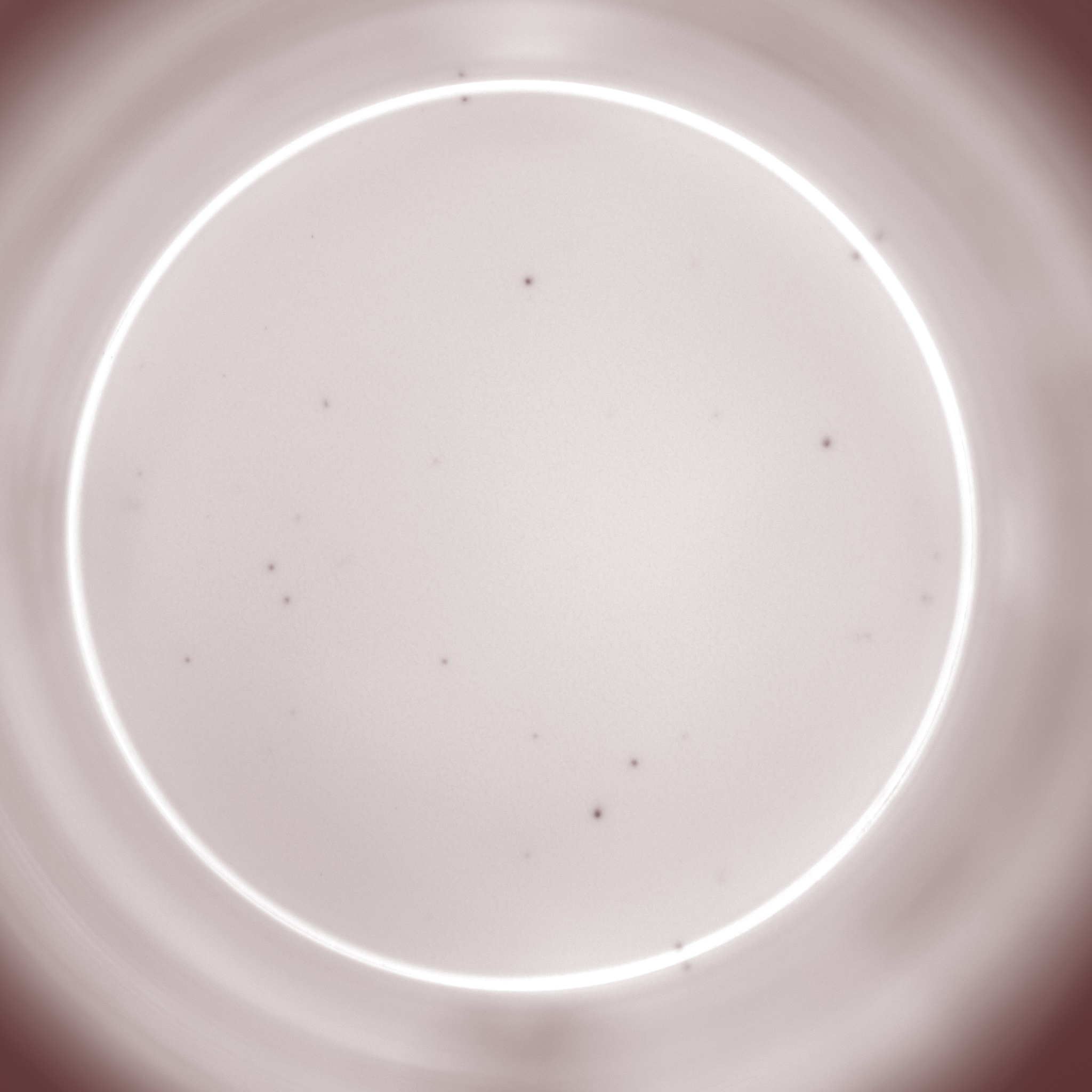

Supplement: Supplementary file 7 — Source data Fig. 4 [file 44321_2024_76_MOESM7_ESM.zip › Figure 4F/IL-4/His-HA.jpg]

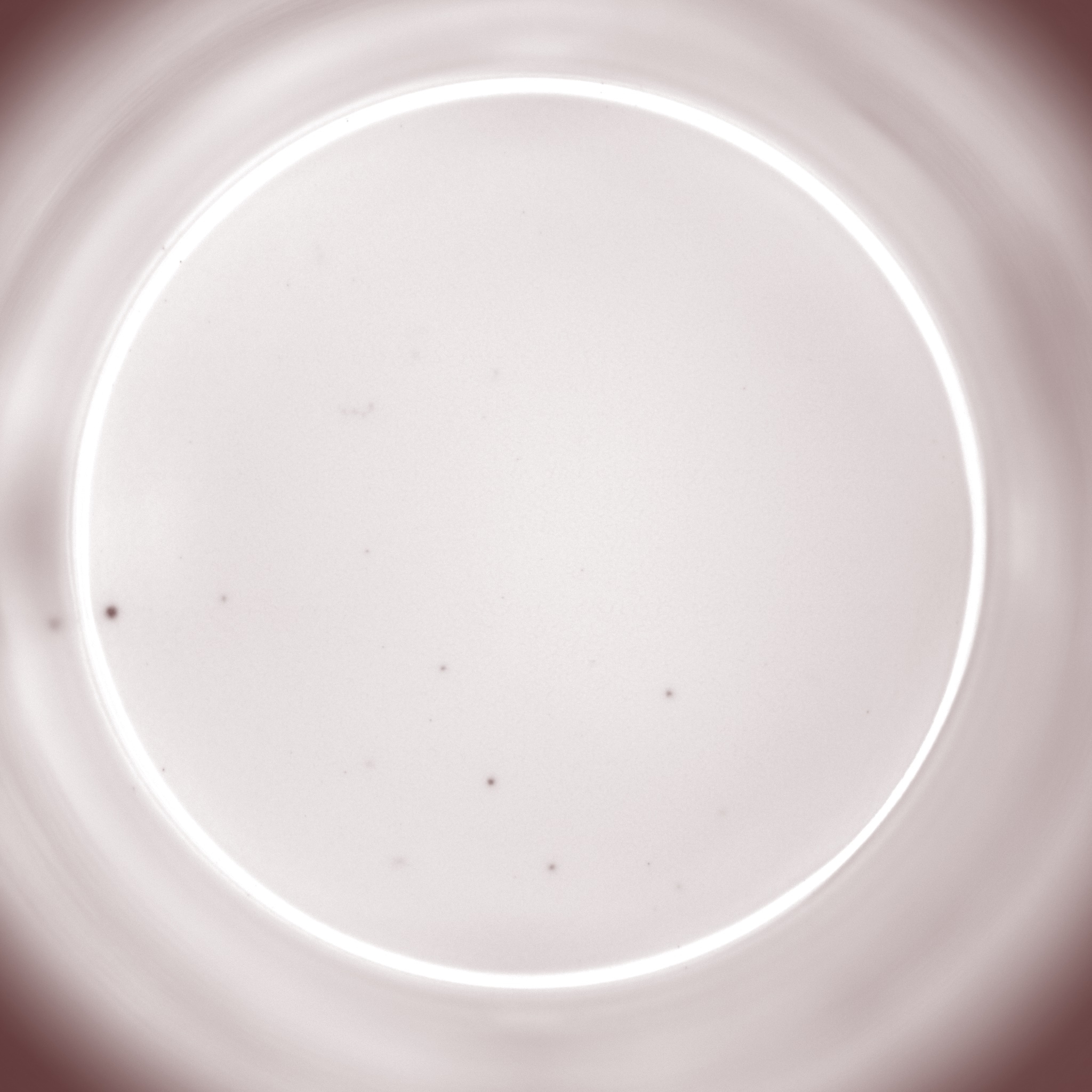

Supplement: Supplementary file 7 — Source data Fig. 4 [file 44321_2024_76_MOESM7_ESM.zip › Figure 4F/IL-4/Mock.jpg]

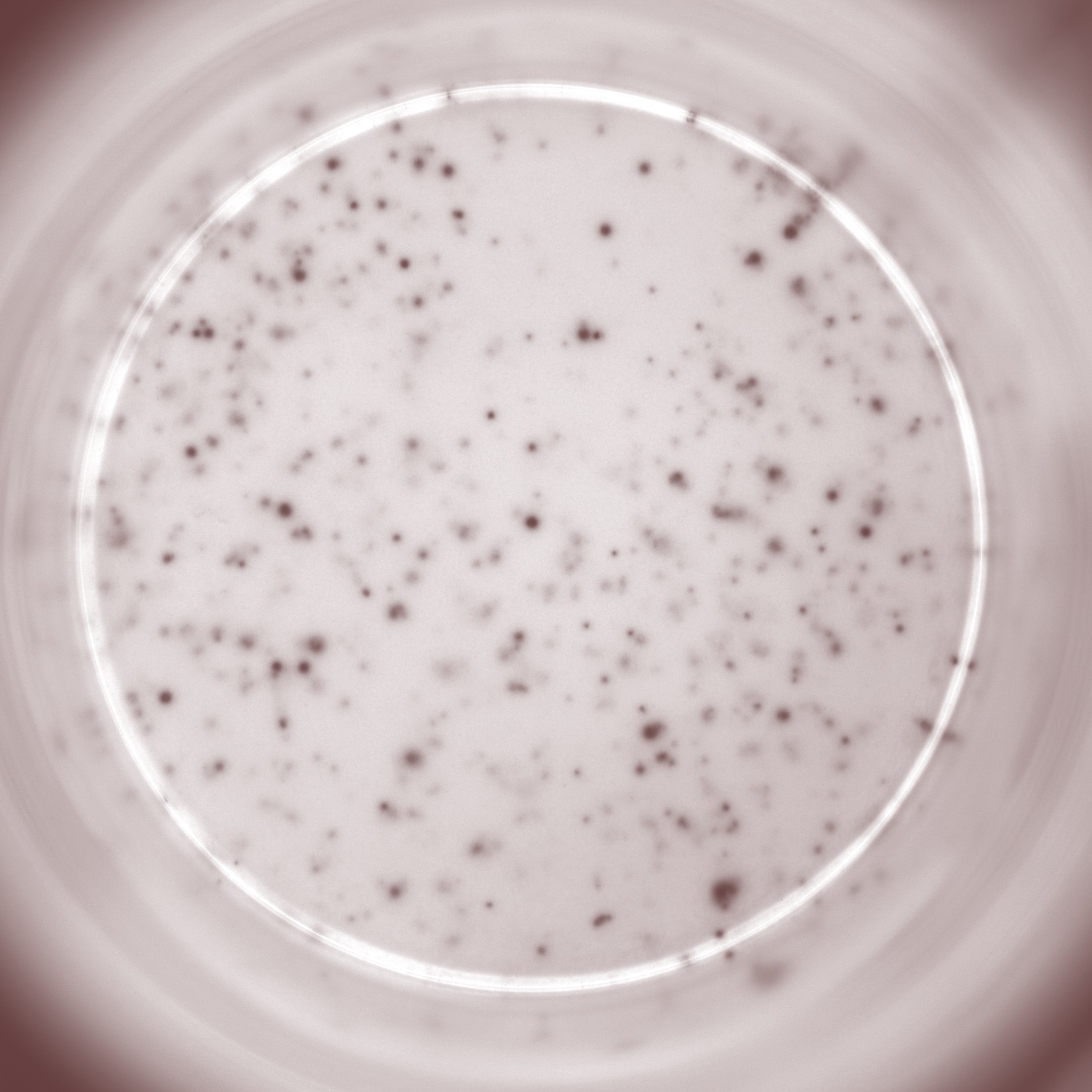

Supplement: Supplementary file 7 — Source data Fig. 4 [file 44321_2024_76_MOESM7_ESM.zip › Figure 4F/IL-4/PMAIono.jpg]

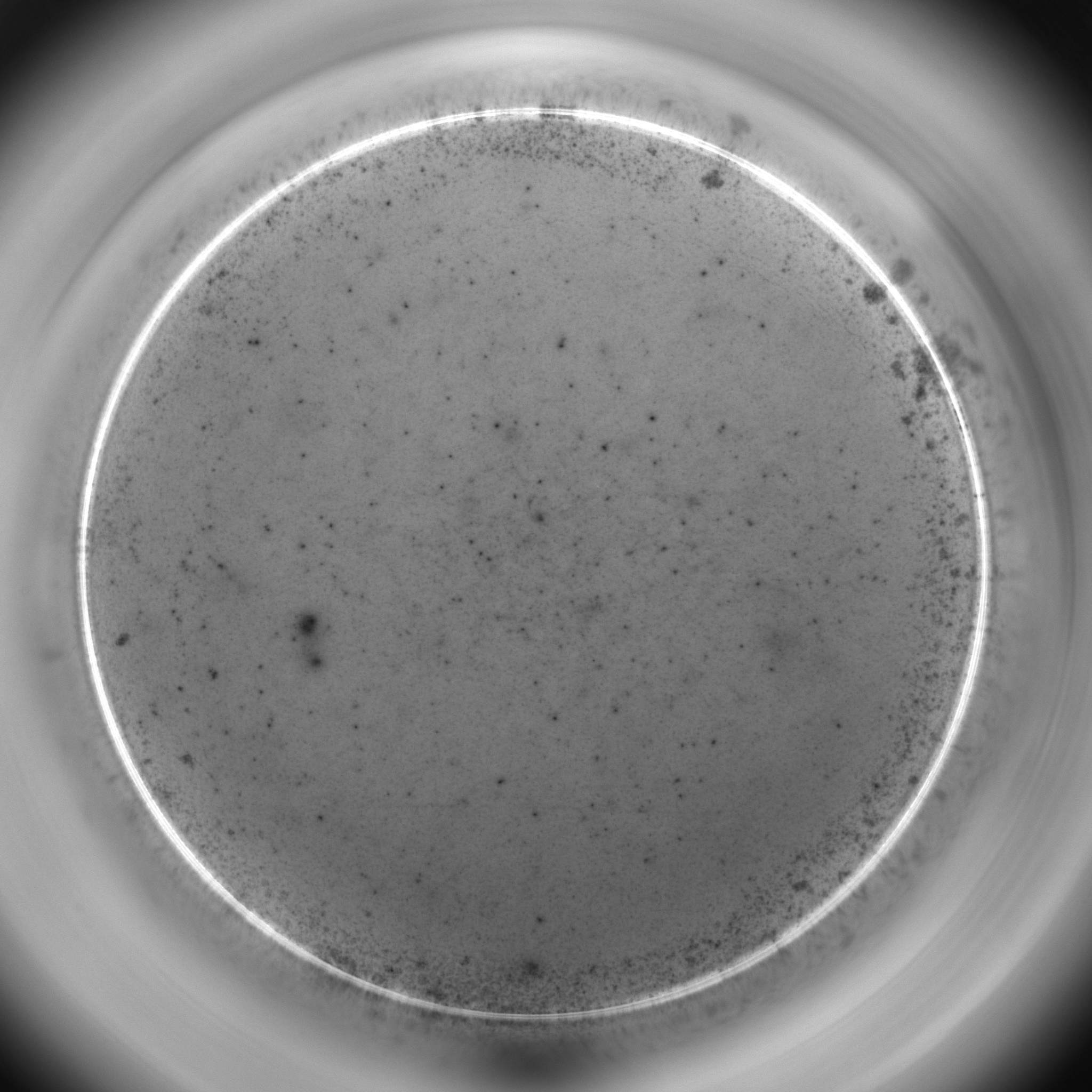

Supplement: Supplementary file 8 — Source data Fig. 5 [file 44321_2024_76_MOESM8_ESM.zip › Figure 5K/Bone marrow/Fc-HA-NPs.jpg]

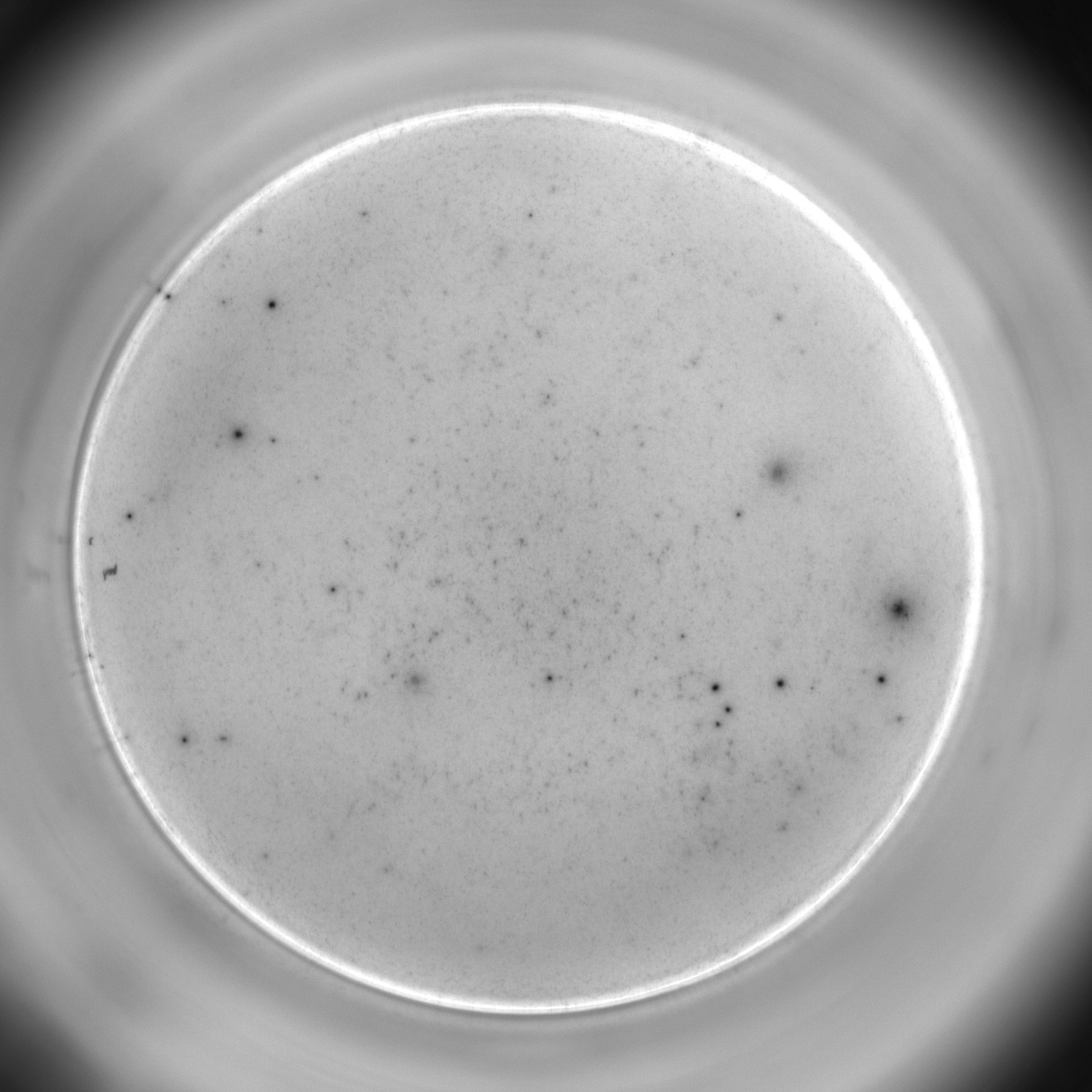

Supplement: Supplementary file 8 — Source data Fig. 5 [file 44321_2024_76_MOESM8_ESM.zip › Figure 5K/Bone marrow/Fc-HA.jpg]

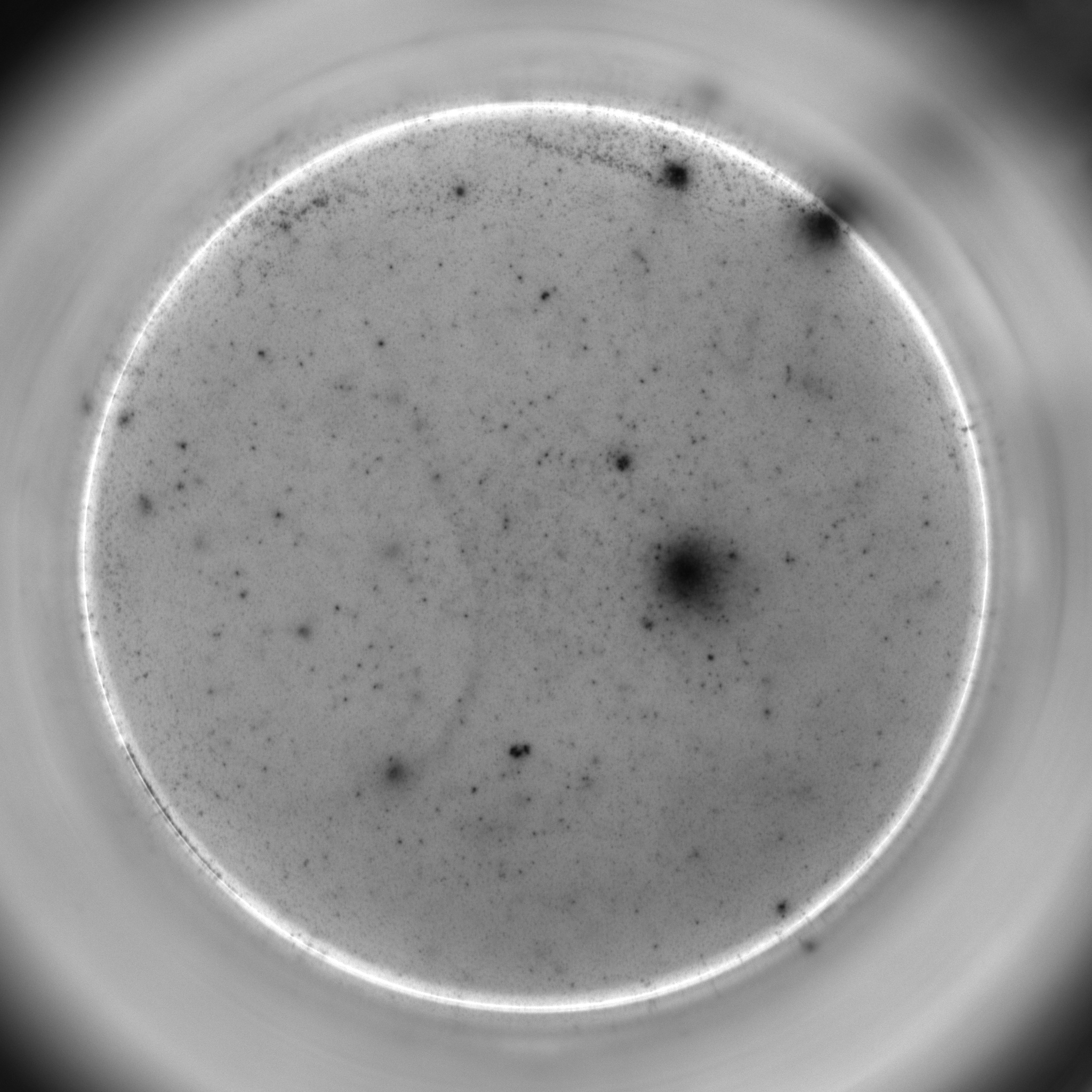

Supplement: Supplementary file 8 — Source data Fig. 5 [file 44321_2024_76_MOESM8_ESM.zip › Figure 5K/Bone marrow/His-HA-NPs.jpg]

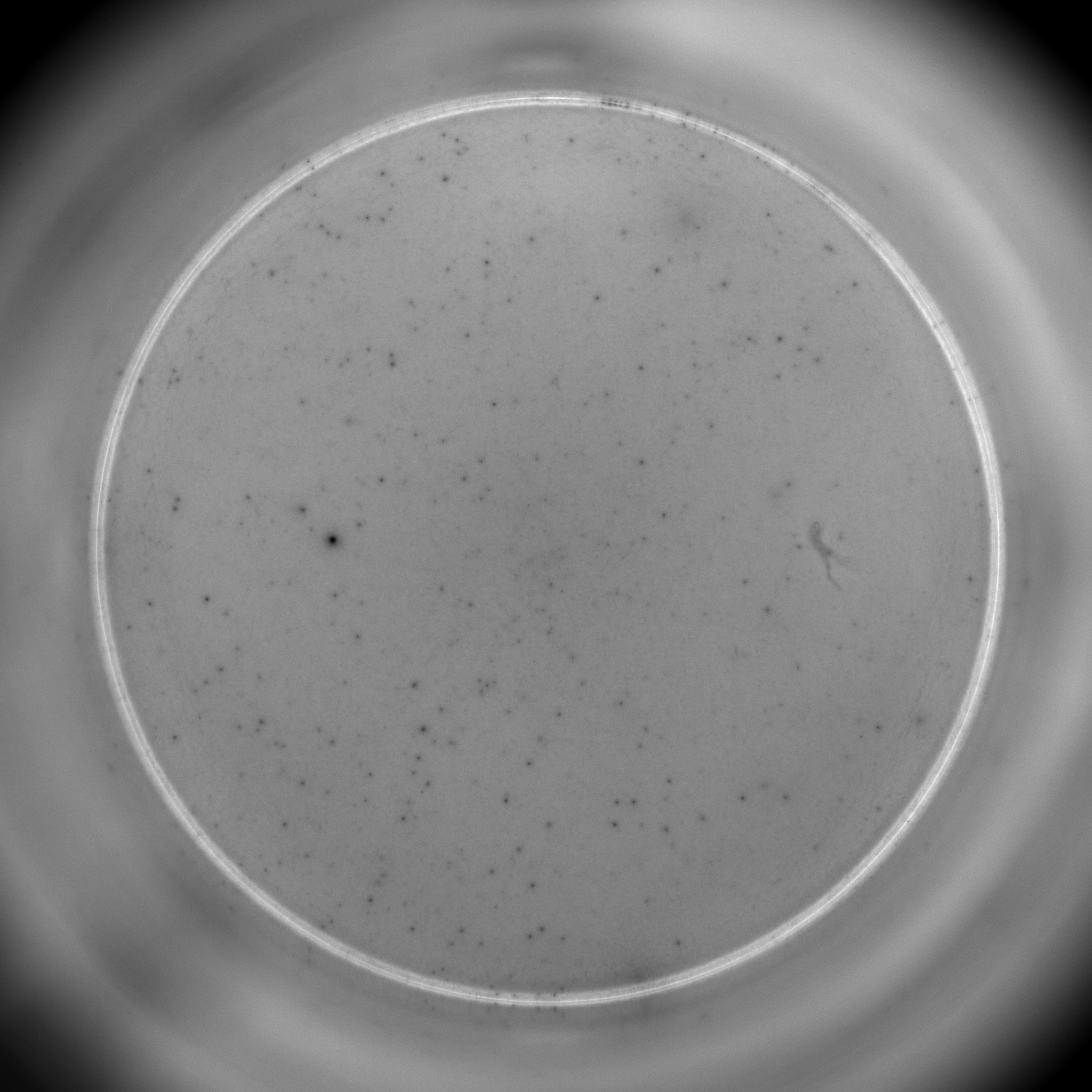

Supplement: Supplementary file 8 — Source data Fig. 5 [file 44321_2024_76_MOESM8_ESM.zip › Figure 5K/Bone marrow/His-HA.jpg]

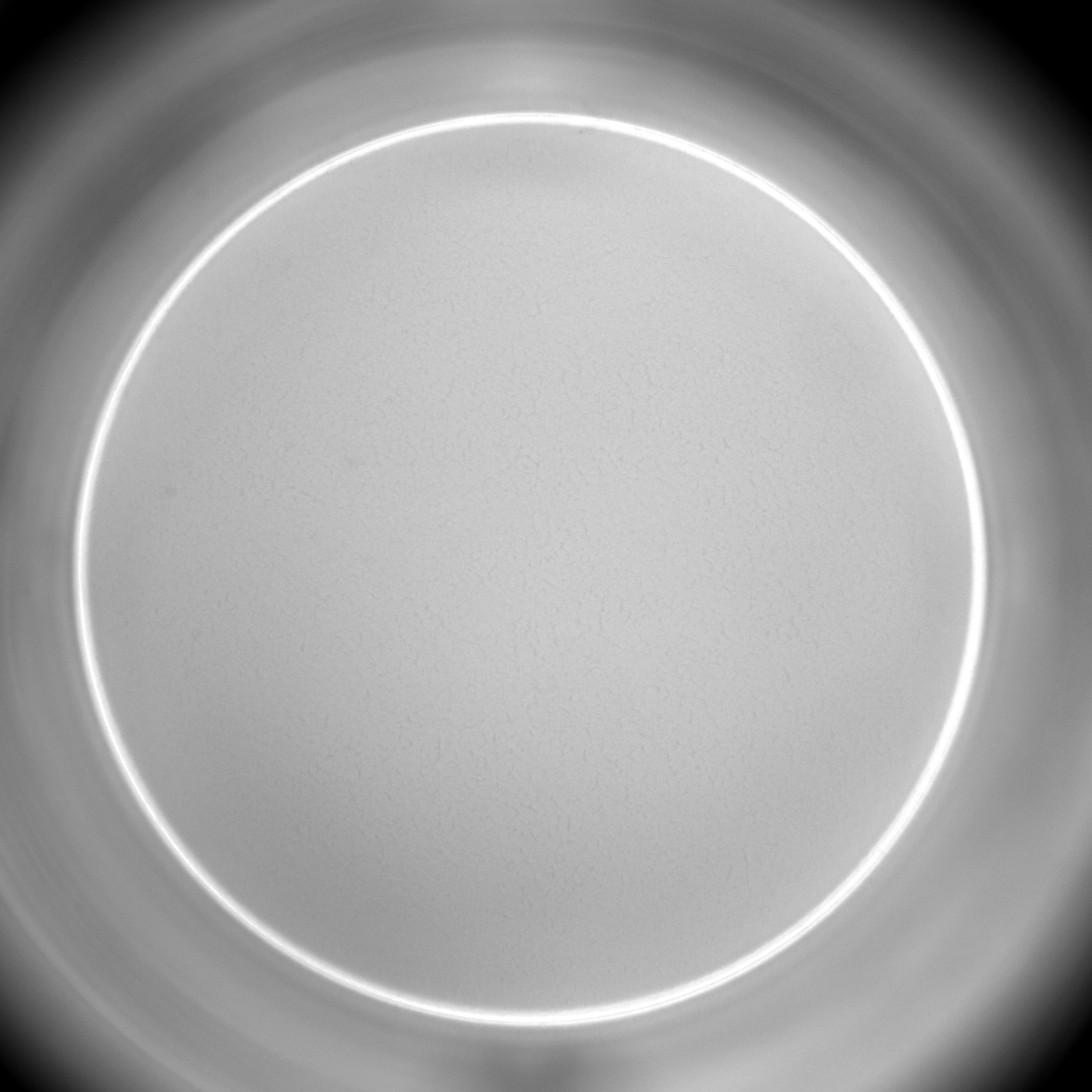

Supplement: Supplementary file 8 — Source data Fig. 5 [file 44321_2024_76_MOESM8_ESM.zip › Figure 5K/Bone marrow/Mock.jpg]

## Slide 1
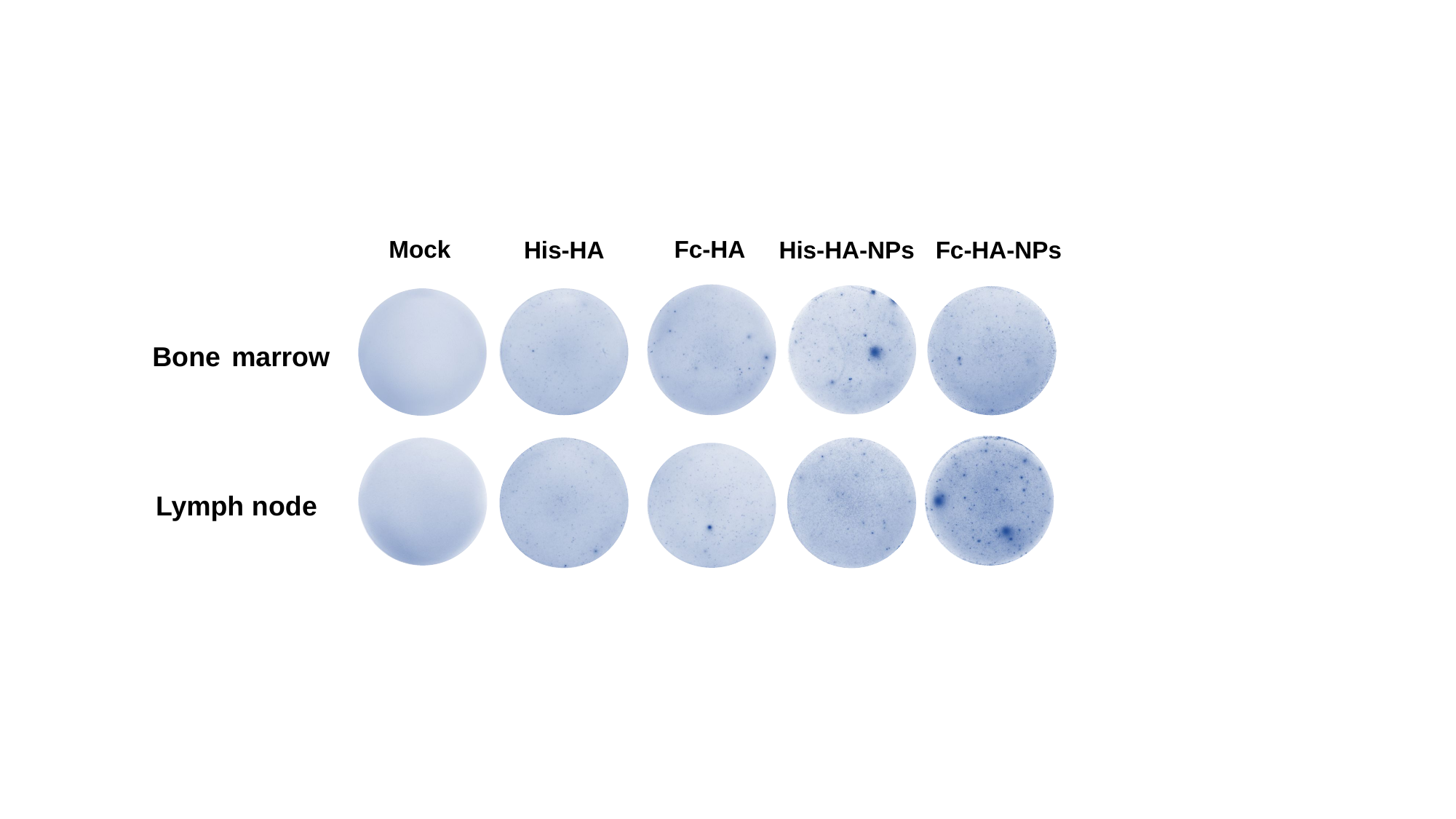

Mock
Fc-HA
His-HA
His-HA-NPs
Fc-HA-NPs
Bone marrow
Lymph node

Supplement: Supplementary file 8 — Source data Fig. 5 [file 44321_2024_76_MOESM8_ESM.zip › Figure 5K/Fiugure 5K.pptx]

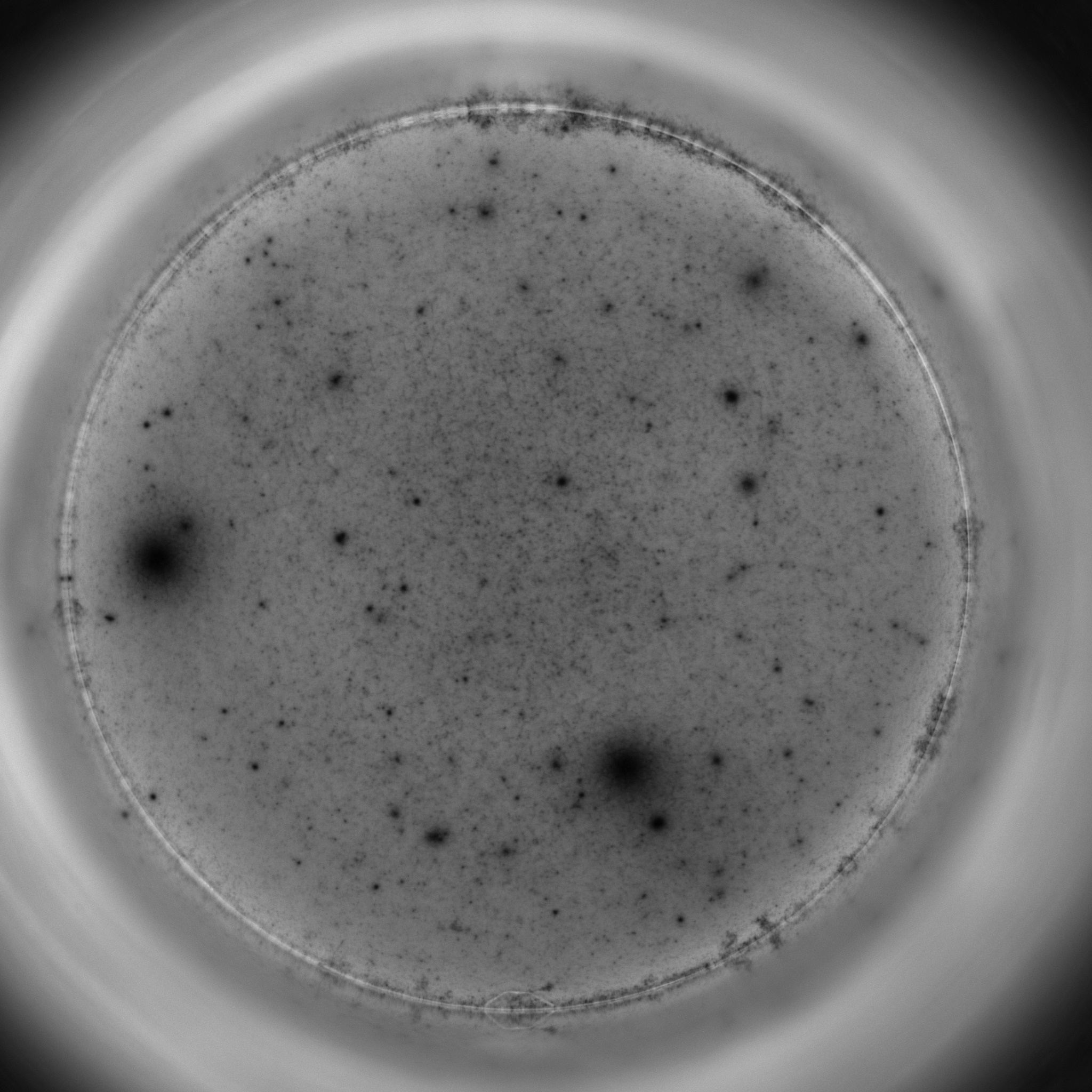

Supplement: Supplementary file 8 — Source data Fig. 5 [file 44321_2024_76_MOESM8_ESM.zip › Figure 5K/Lymph node/Fc-HA-NPs.jpg]

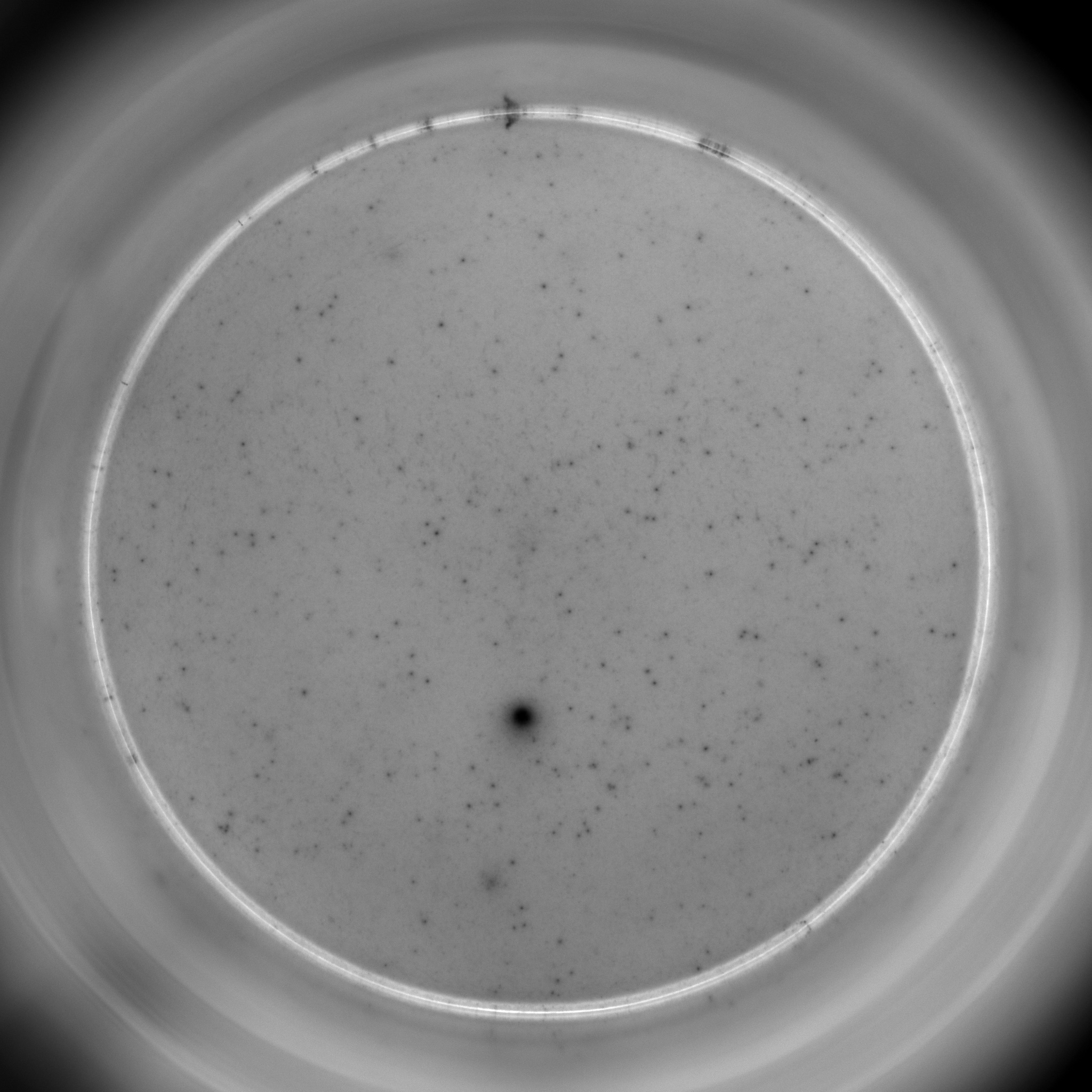

Supplement: Supplementary file 8 — Source data Fig. 5 [file 44321_2024_76_MOESM8_ESM.zip › Figure 5K/Lymph node/Fc-HA.jpg]

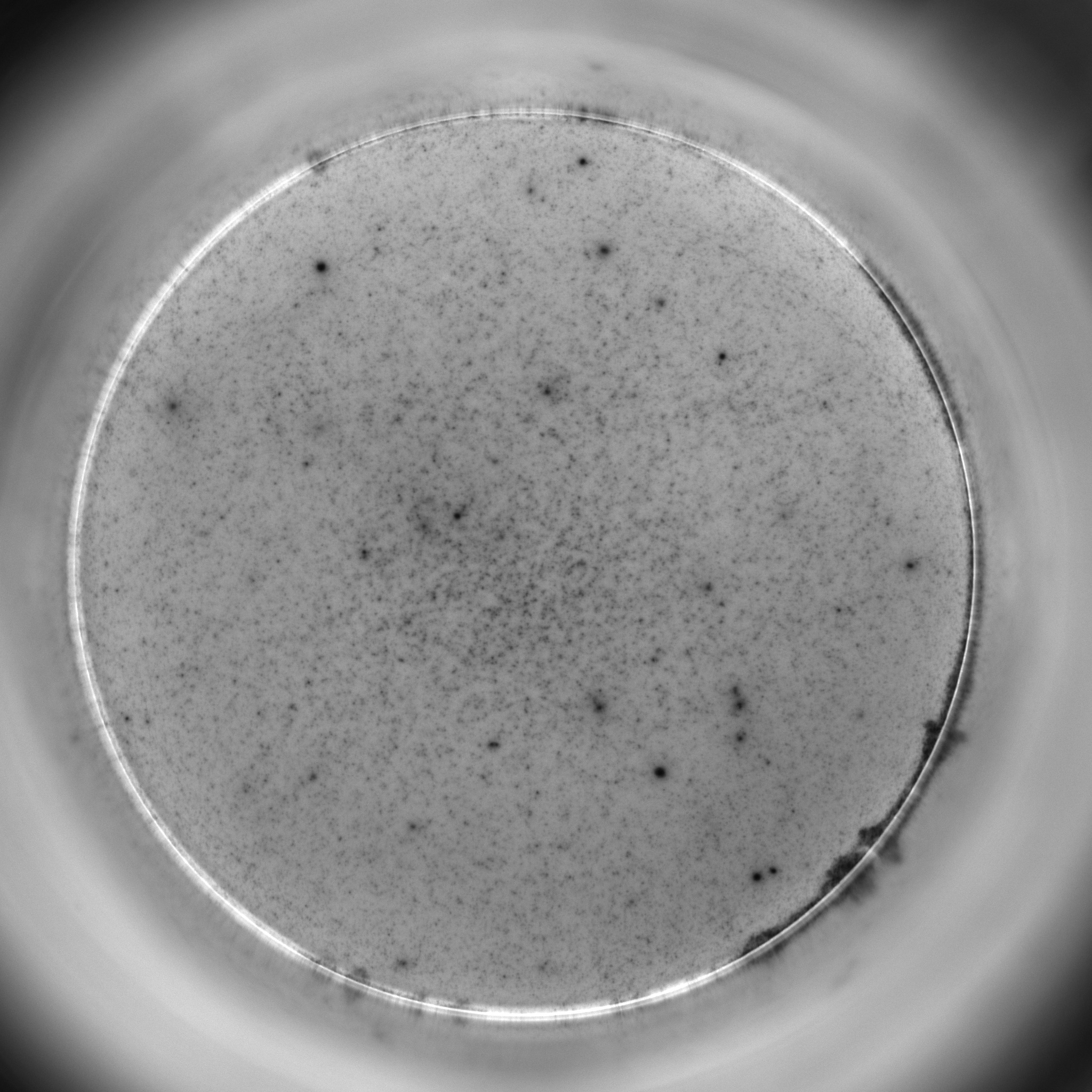

Supplement: Supplementary file 8 — Source data Fig. 5 [file 44321_2024_76_MOESM8_ESM.zip › Figure 5K/Lymph node/His-HA-NPs.jpg]

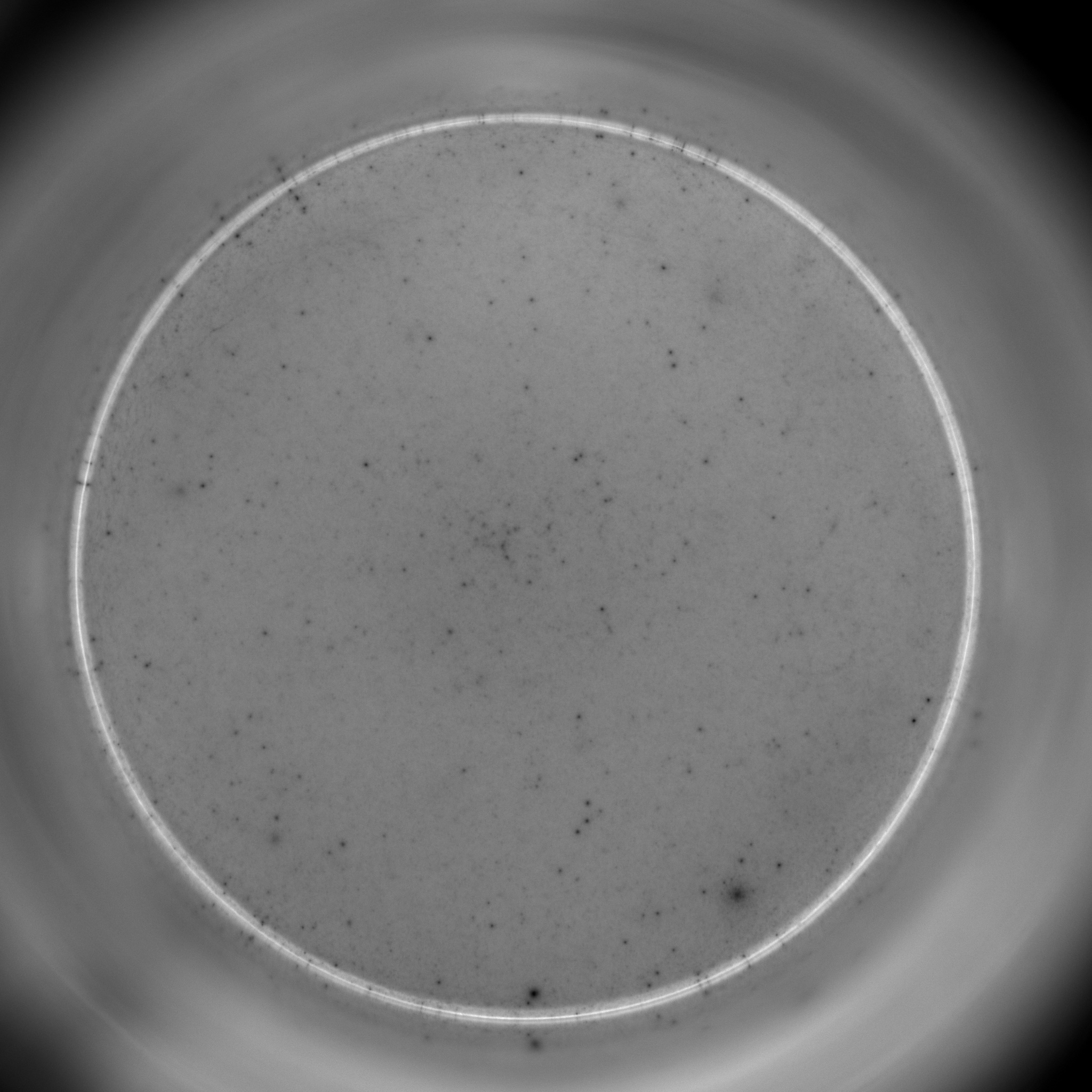

Supplement: Supplementary file 8 — Source data Fig. 5 [file 44321_2024_76_MOESM8_ESM.zip › Figure 5K/Lymph node/His-HA.jpg]

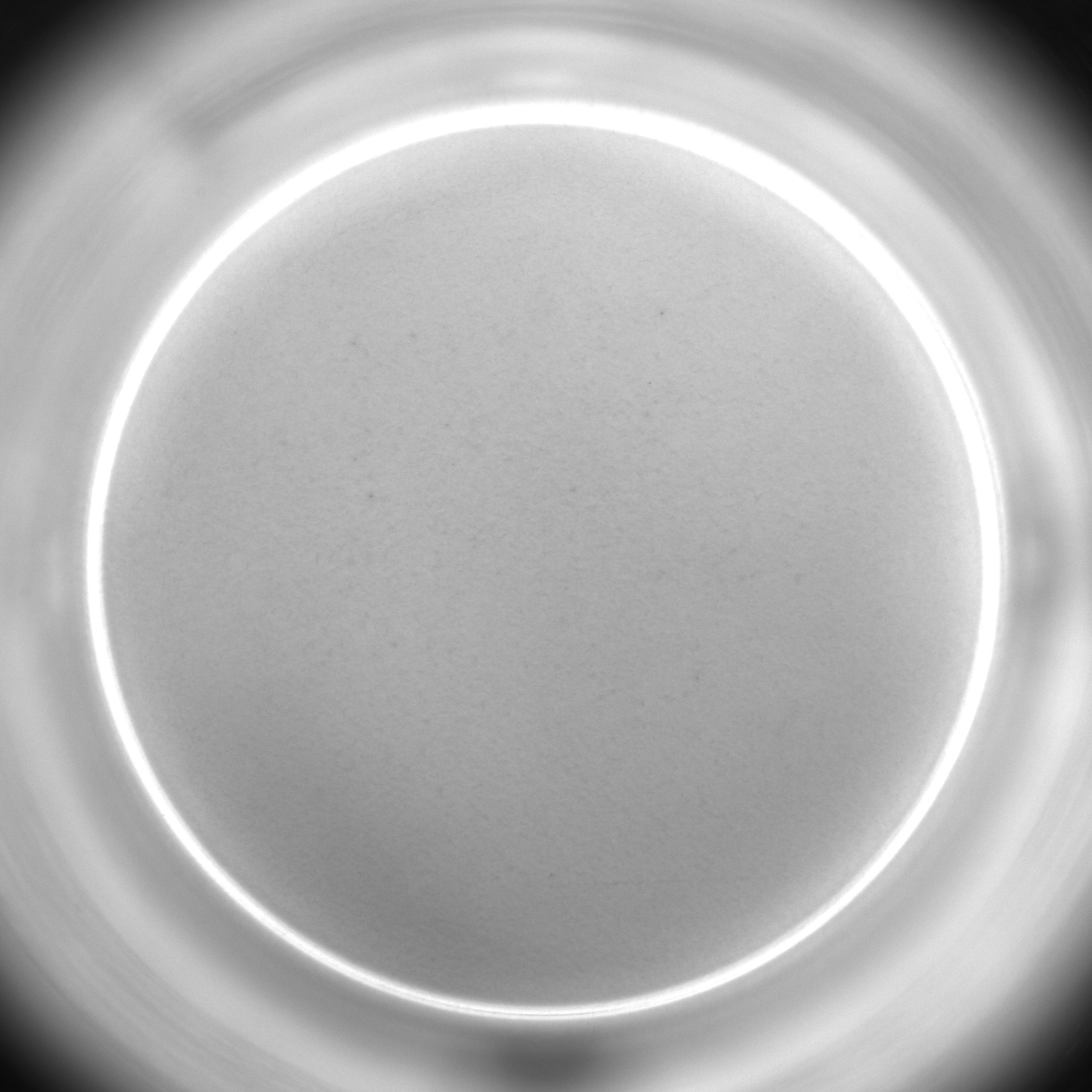

Supplement: Supplementary file 8 — Source data Fig. 5 [file 44321_2024_76_MOESM8_ESM.zip › Figure 5K/Lymph node/Mock.jpg]

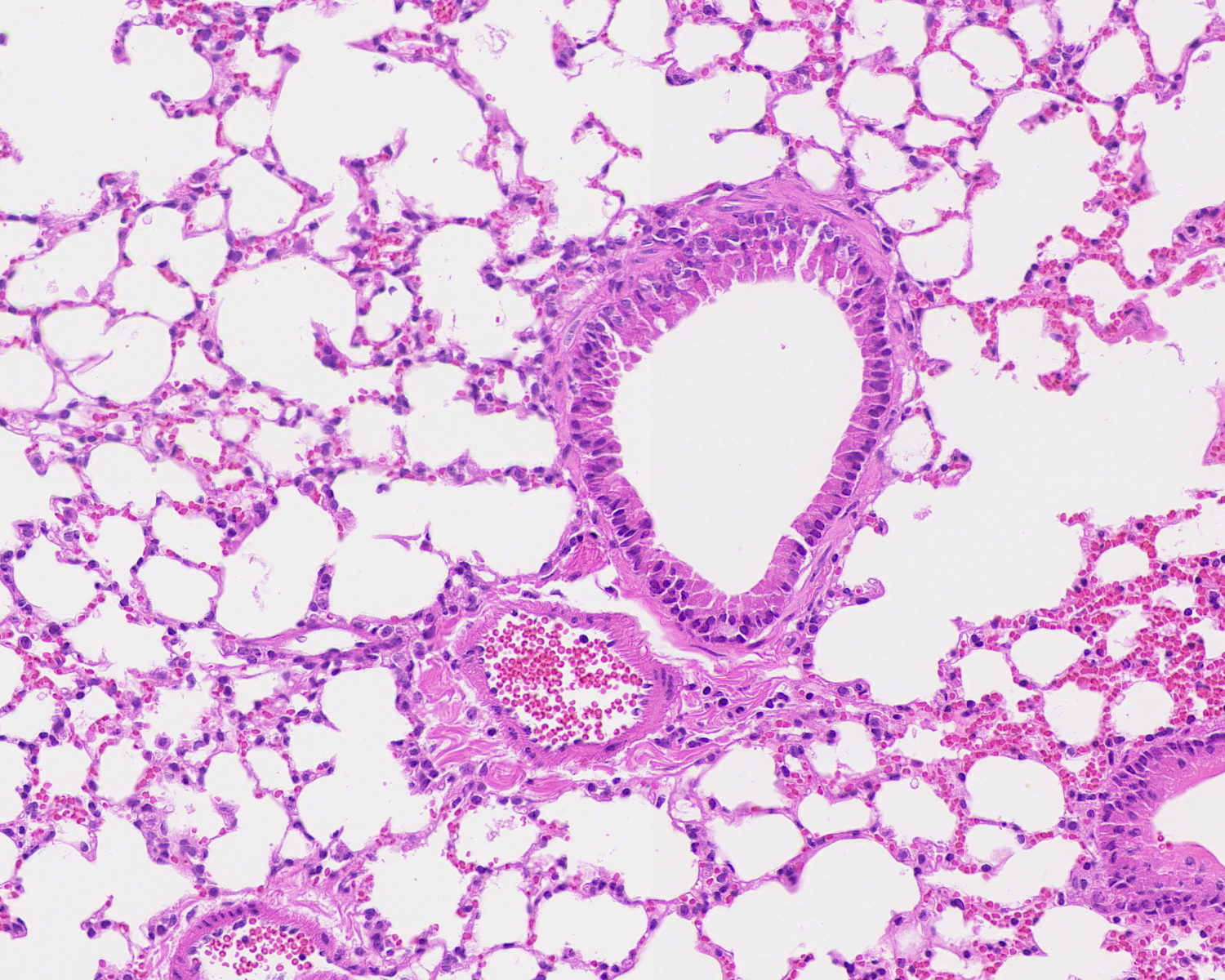

Supplement: Supplementary file 9 — Source data Fig. 6 [file 44321_2024_76_MOESM9_ESM.zip › Figure 6D/Figure 6D-H&E/Fc-HA-NPs/Fc-HA-NPs-20x.jpg]

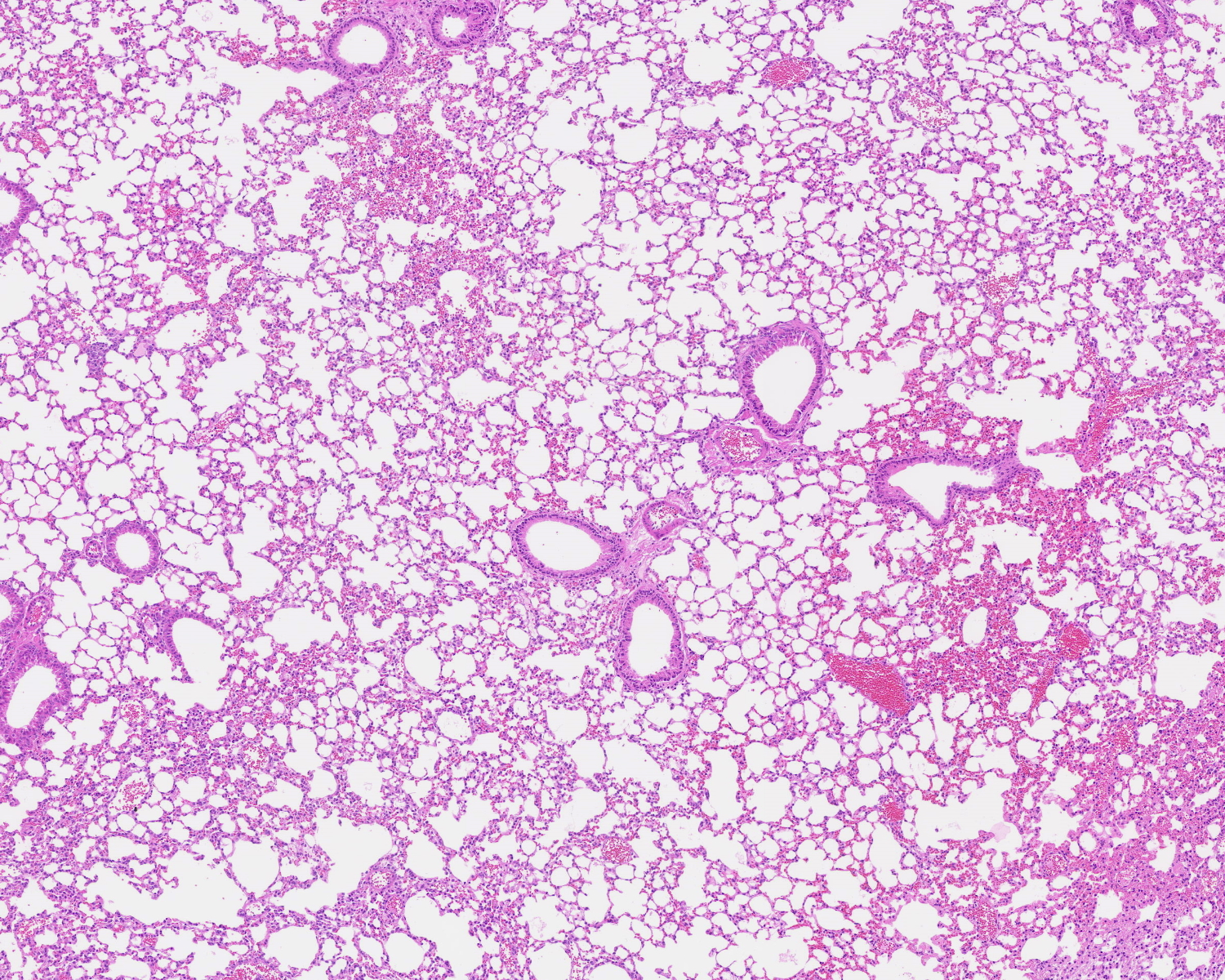

Supplement: Supplementary file 9 — Source data Fig. 6 [file 44321_2024_76_MOESM9_ESM.zip › Figure 6D/Figure 6D-H&E/Fc-HA-NPs/Fc-HA-NPs-4x.jpg]

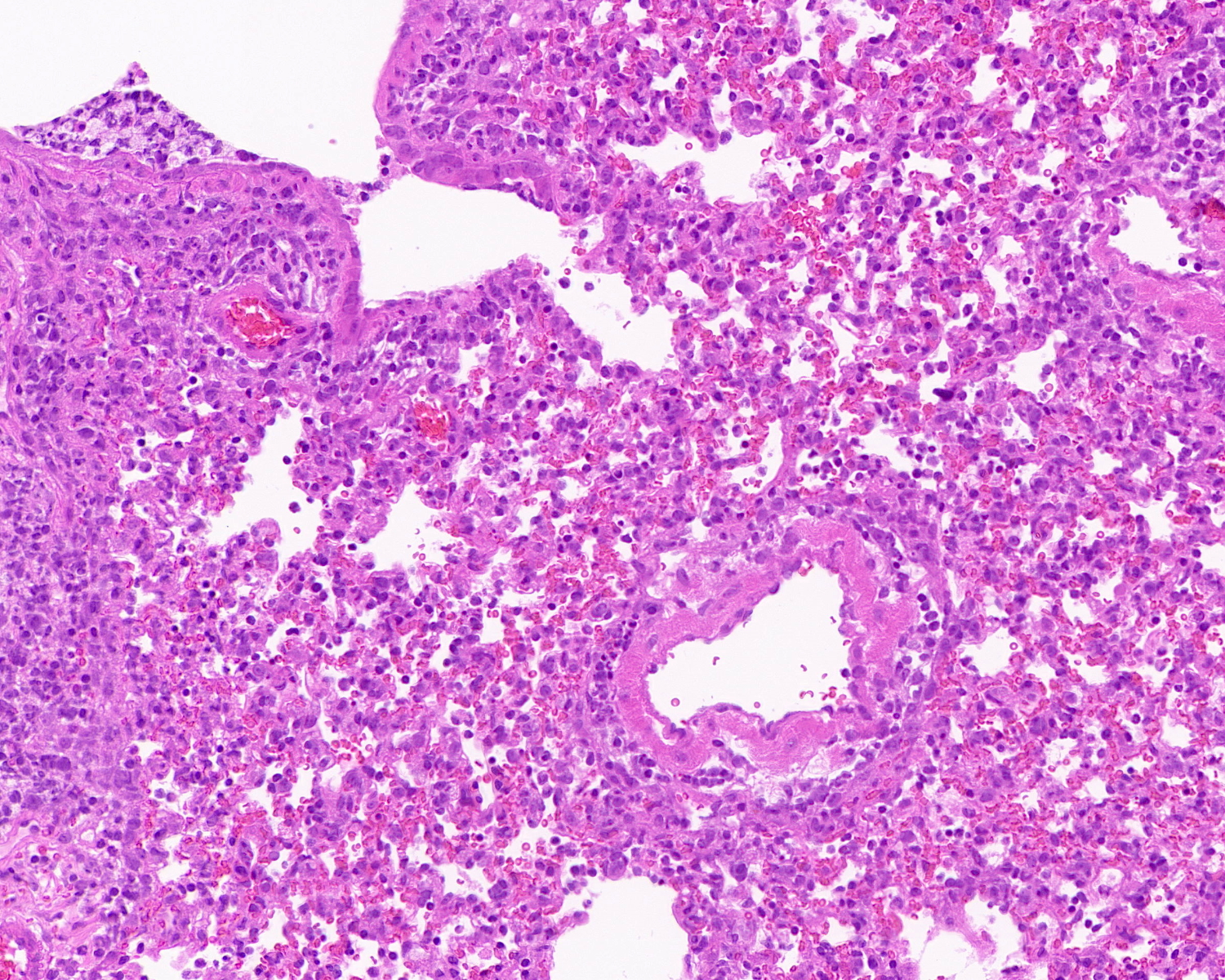

Supplement: Supplementary file 9 — Source data Fig. 6 [file 44321_2024_76_MOESM9_ESM.zip › Figure 6D/Figure 6D-H&E/Fc-HA/Fc-HA-20x.jpg]

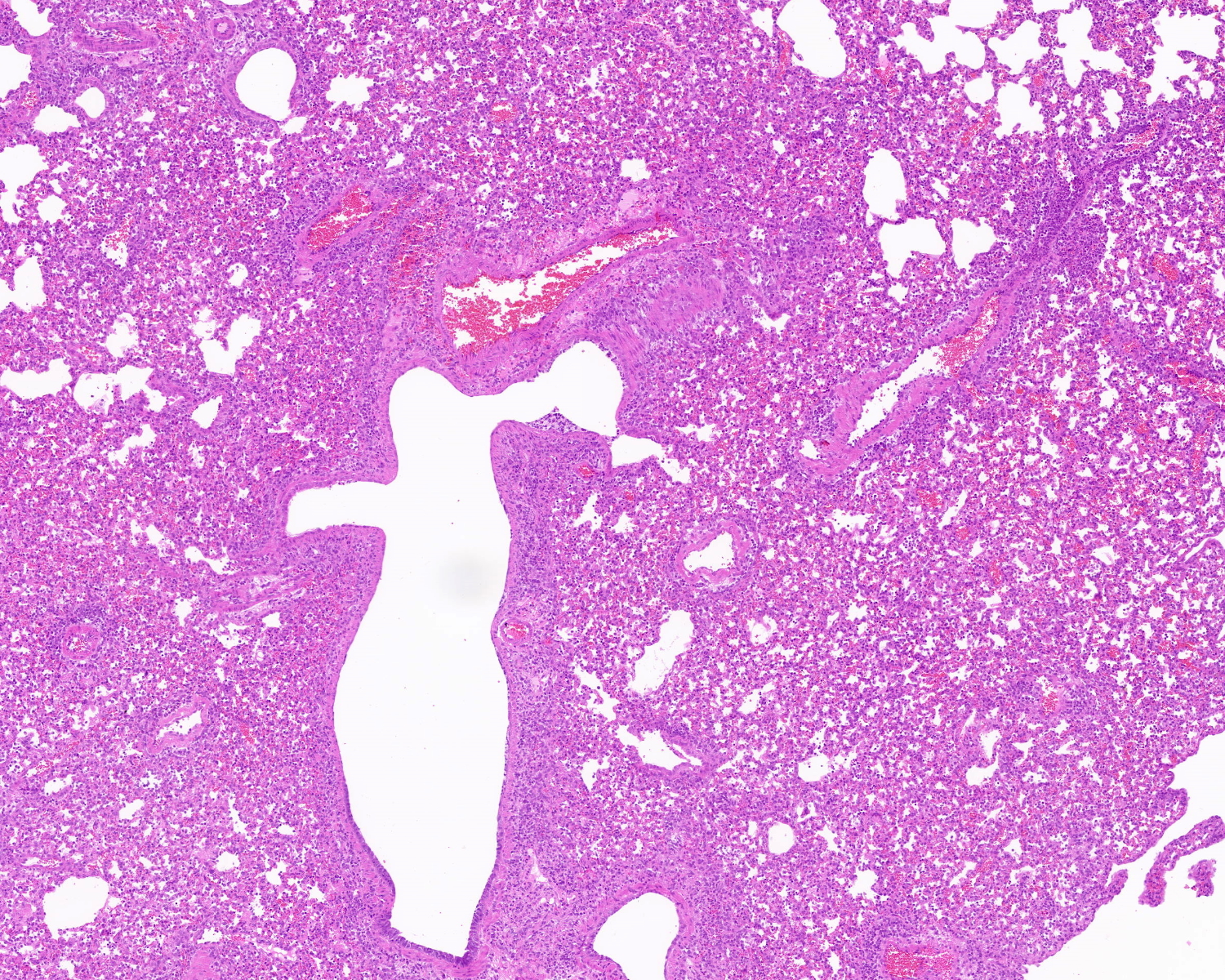

Supplement: Supplementary file 9 — Source data Fig. 6 [file 44321_2024_76_MOESM9_ESM.zip › Figure 6D/Figure 6D-H&E/Fc-HA/FC-HA-4x.jpg]

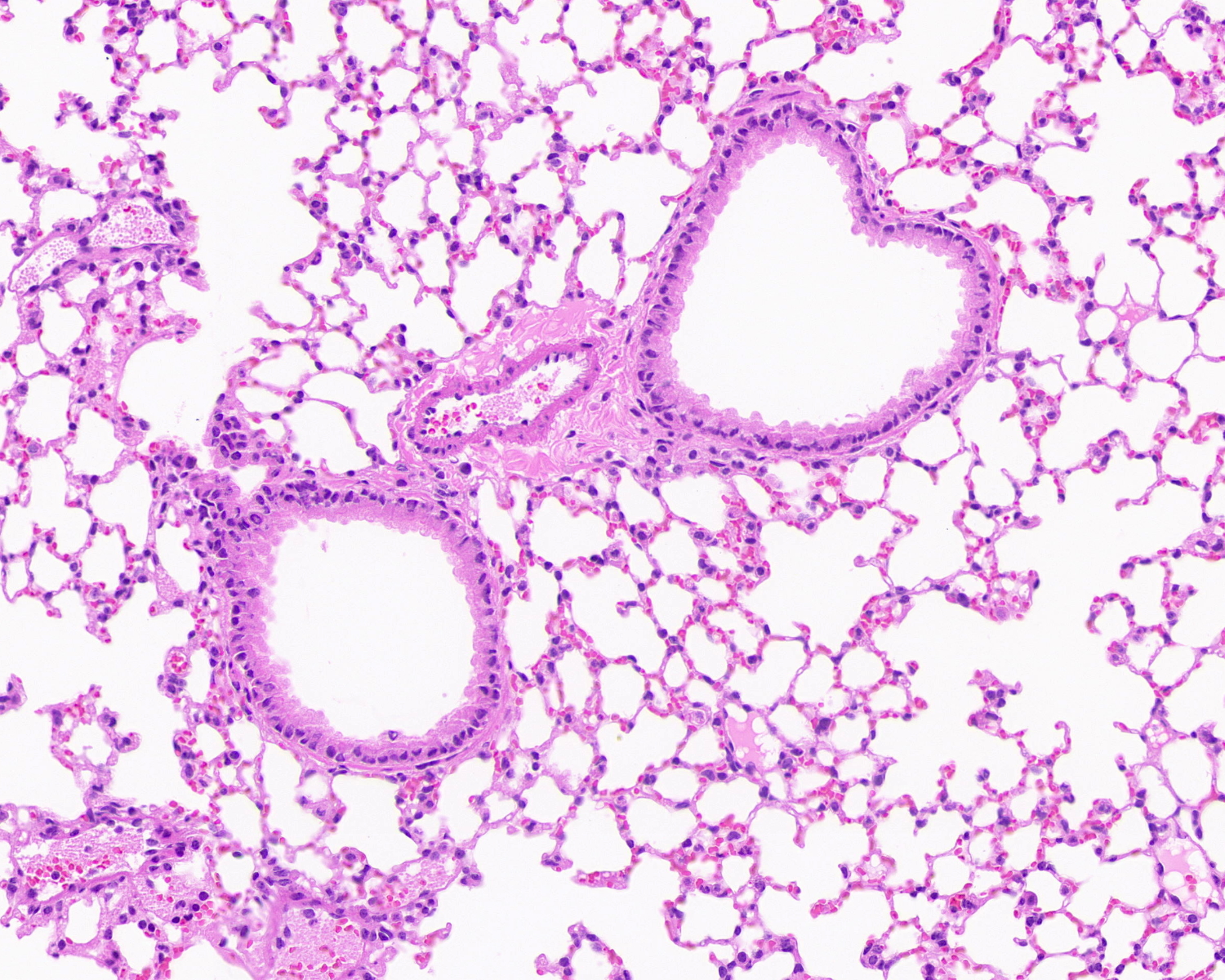

Supplement: Supplementary file 9 — Source data Fig. 6 [file 44321_2024_76_MOESM9_ESM.zip › Figure 6D/Figure 6D-H&E/His-HA-NPs/His-HA-NPs-20x.jpg]

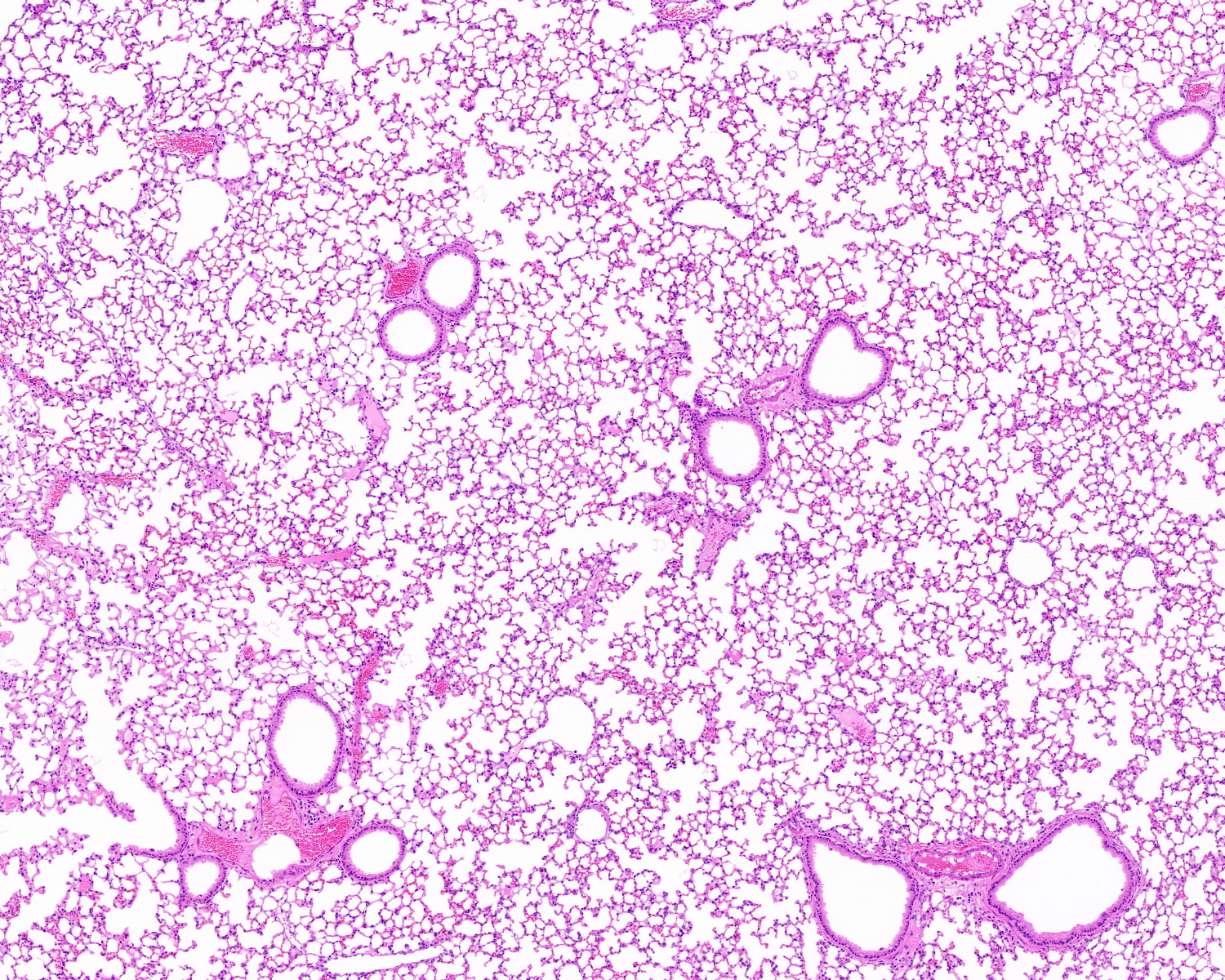

Supplement: Supplementary file 9 — Source data Fig. 6 [file 44321_2024_76_MOESM9_ESM.zip › Figure 6D/Figure 6D-H&E/His-HA-NPs/His-HA-NPs-4x.jpg]
