# Supplementary figures and images for: A broadly applicable protein-polymer adjuvant system for antiviral vaccines (part 2 of 2)
Source: EMBO Mol Med. 2024 May 15;16(6):1451–83. doi: 10.1038/s44321-024-00076-4 (PMC11178928; doi:10.1038/s44321-024-00076-4)

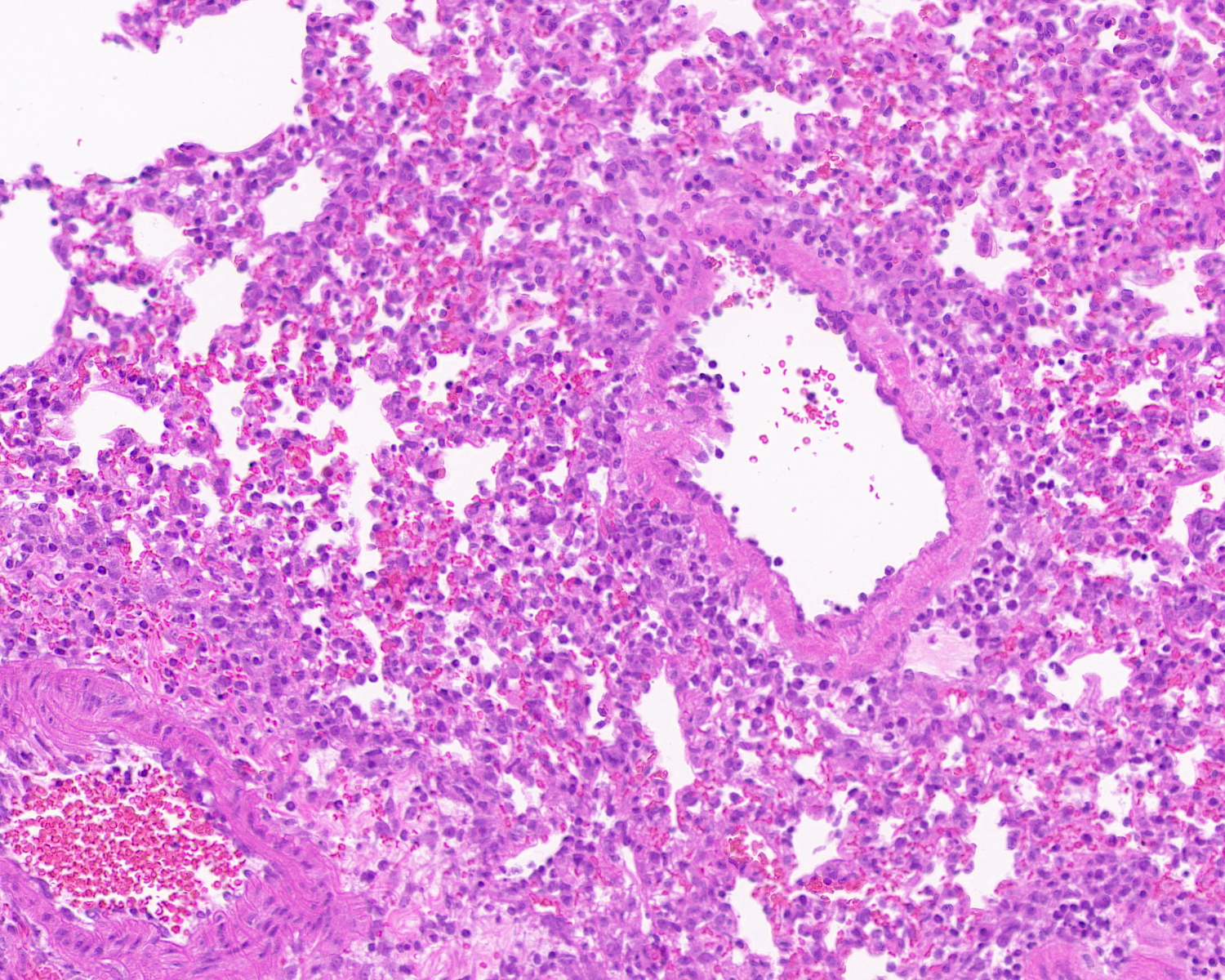

Supplement: Supplementary file 9 — Source data Fig. 6 [file 44321_2024_76_MOESM9_ESM.zip › Figure 6D/Figure 6D-H&E/His-HA/His-HA-20x.jpg]

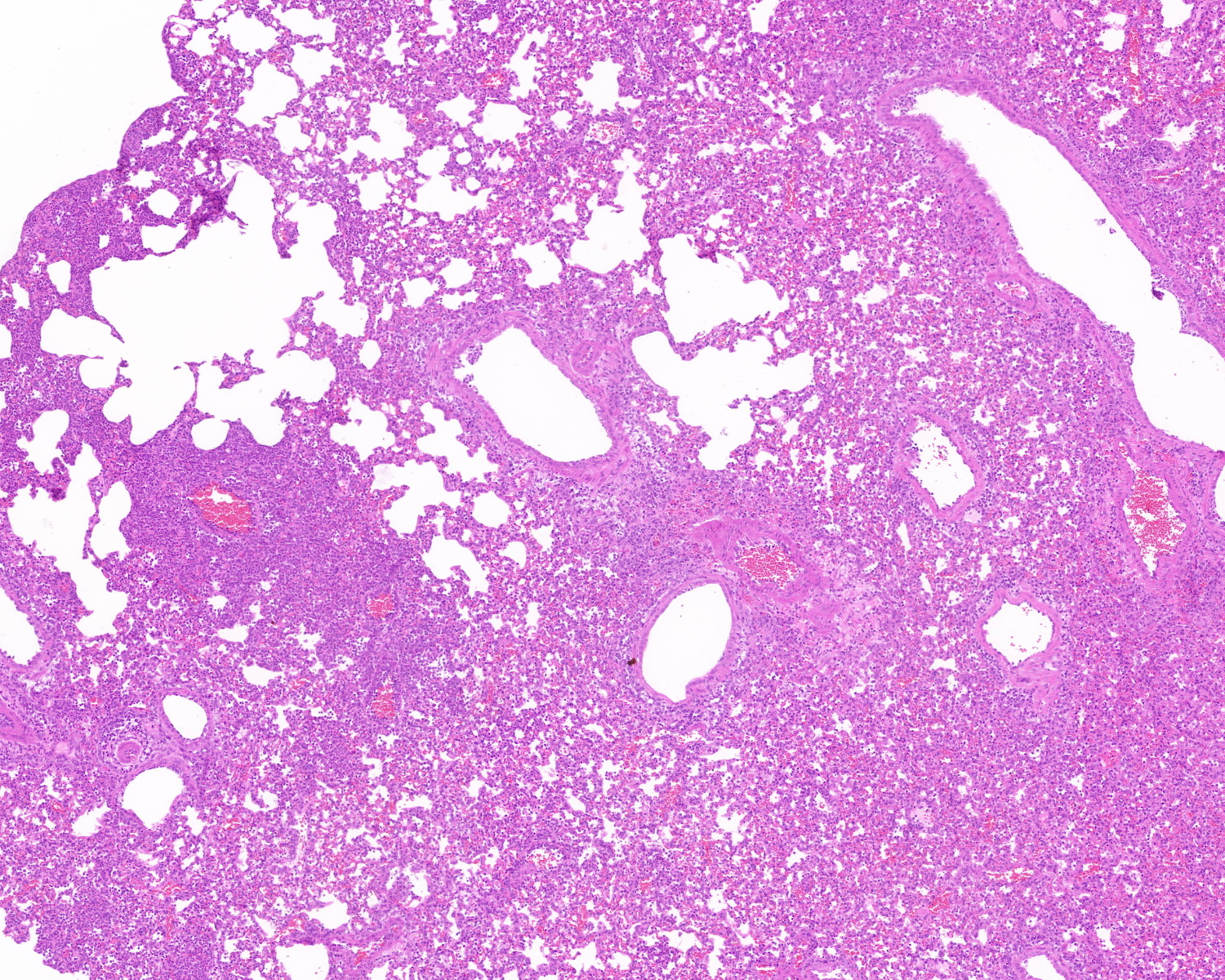

Supplement: Supplementary file 9 — Source data Fig. 6 [file 44321_2024_76_MOESM9_ESM.zip › Figure 6D/Figure 6D-H&E/His-HA/His-HA-4x.jpg]

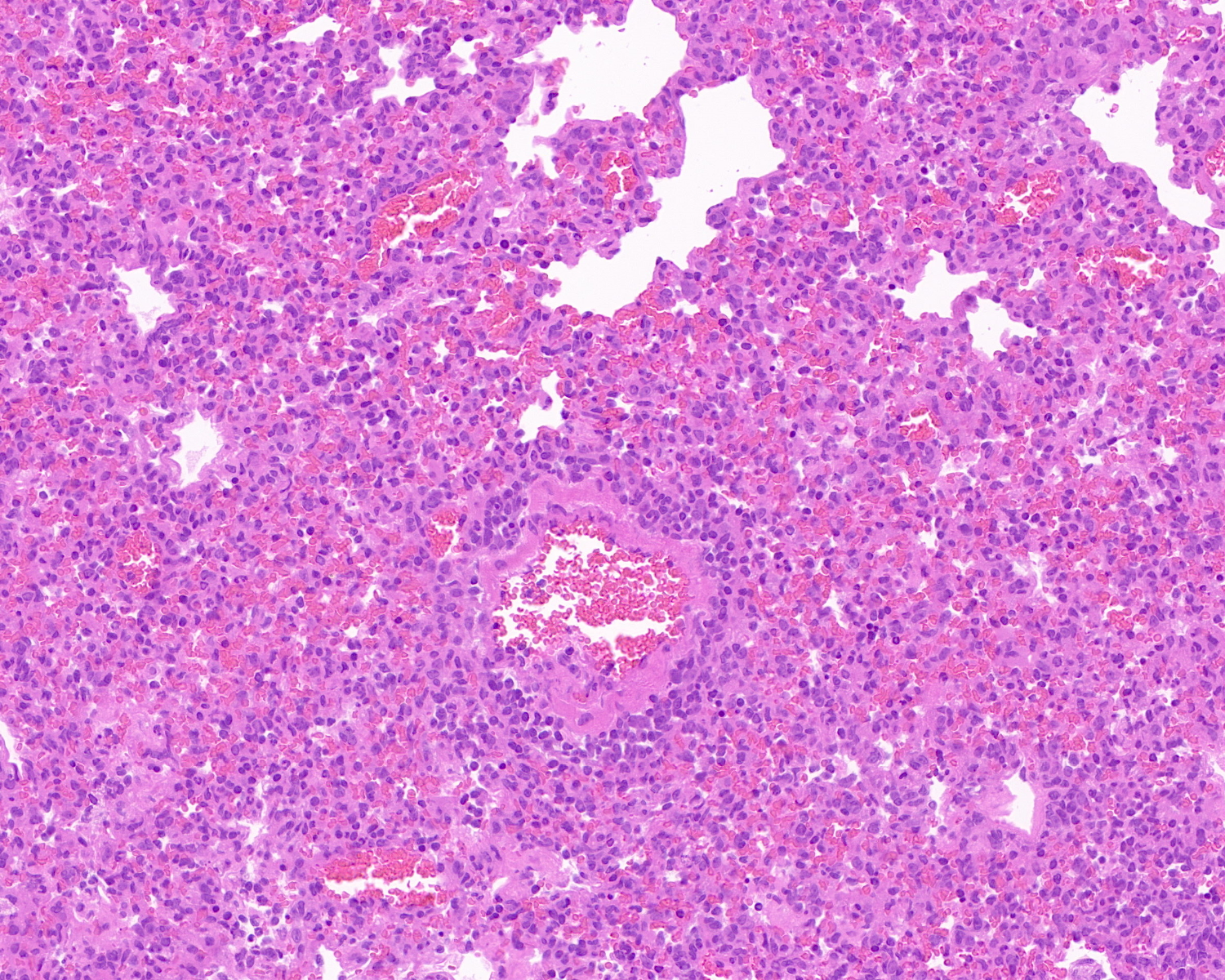

Supplement: Supplementary file 9 — Source data Fig. 6 [file 44321_2024_76_MOESM9_ESM.zip › Figure 6D/Figure 6D-H&E/Mock/Mock-20x.jpg]

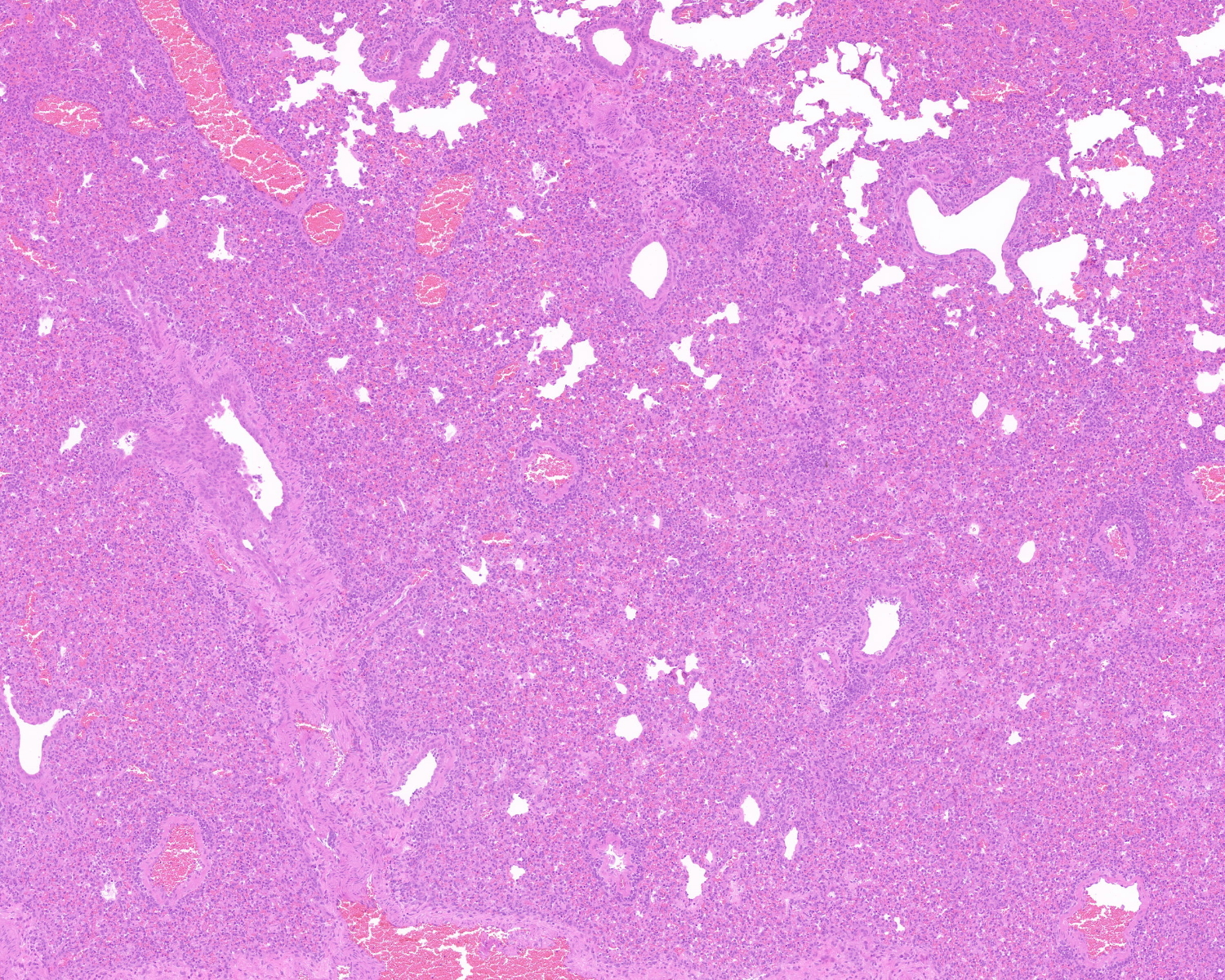

Supplement: Supplementary file 9 — Source data Fig. 6 [file 44321_2024_76_MOESM9_ESM.zip › Figure 6D/Figure 6D-H&E/Mock/Mock-4x.jpg]

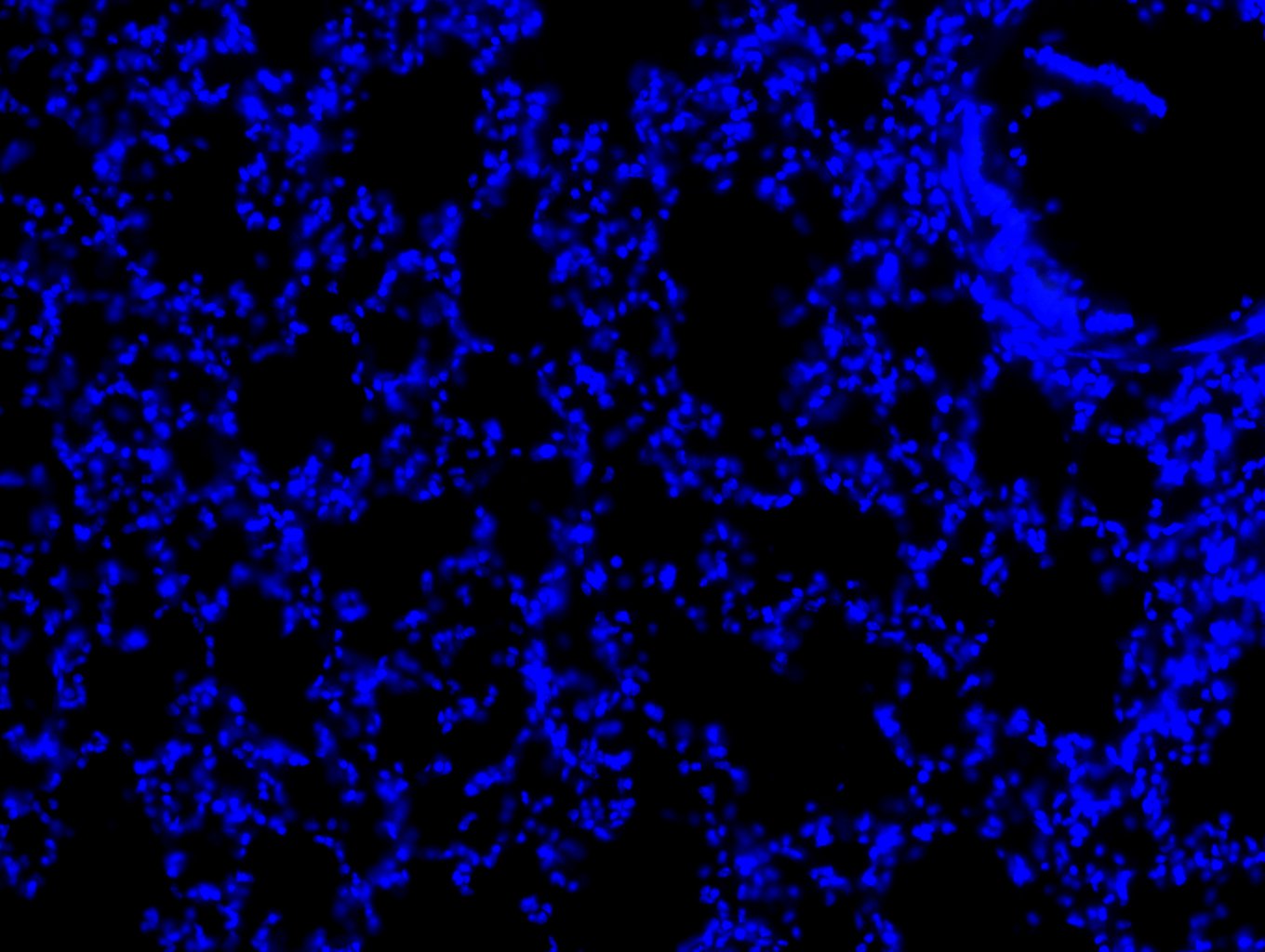

Supplement: Supplementary file 9 — Source data Fig. 6 [file 44321_2024_76_MOESM9_ESM.zip › Figure 6D/Figure 6D-IF/Fc-HA-NPs/Fc-HA-NP-zoom-DAPI.jpg]

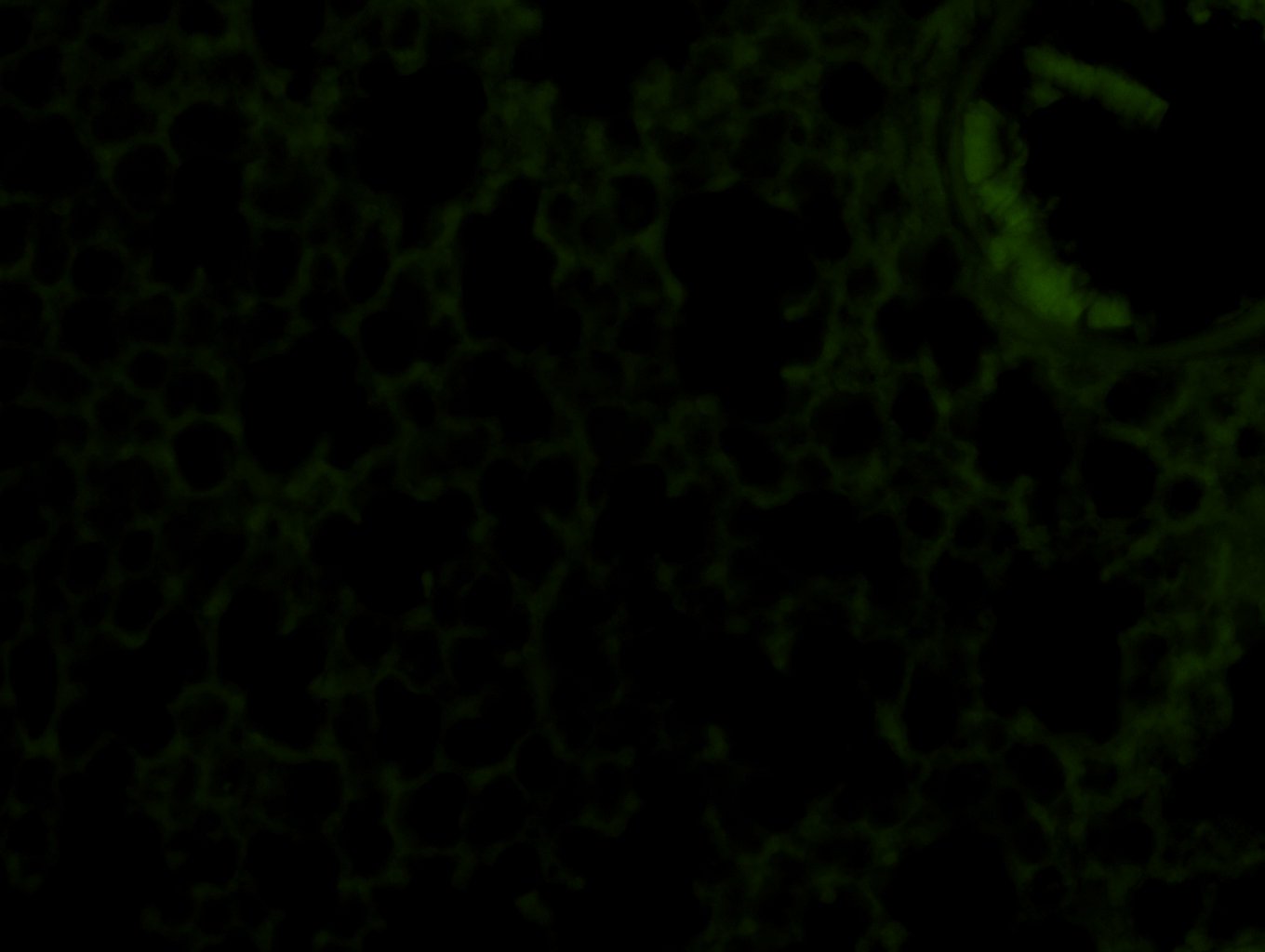

Supplement: Supplementary file 9 — Source data Fig. 6 [file 44321_2024_76_MOESM9_ESM.zip › Figure 6D/Figure 6D-IF/Fc-HA-NPs/Fc-HA-NP-zoom-GFP.jpg]

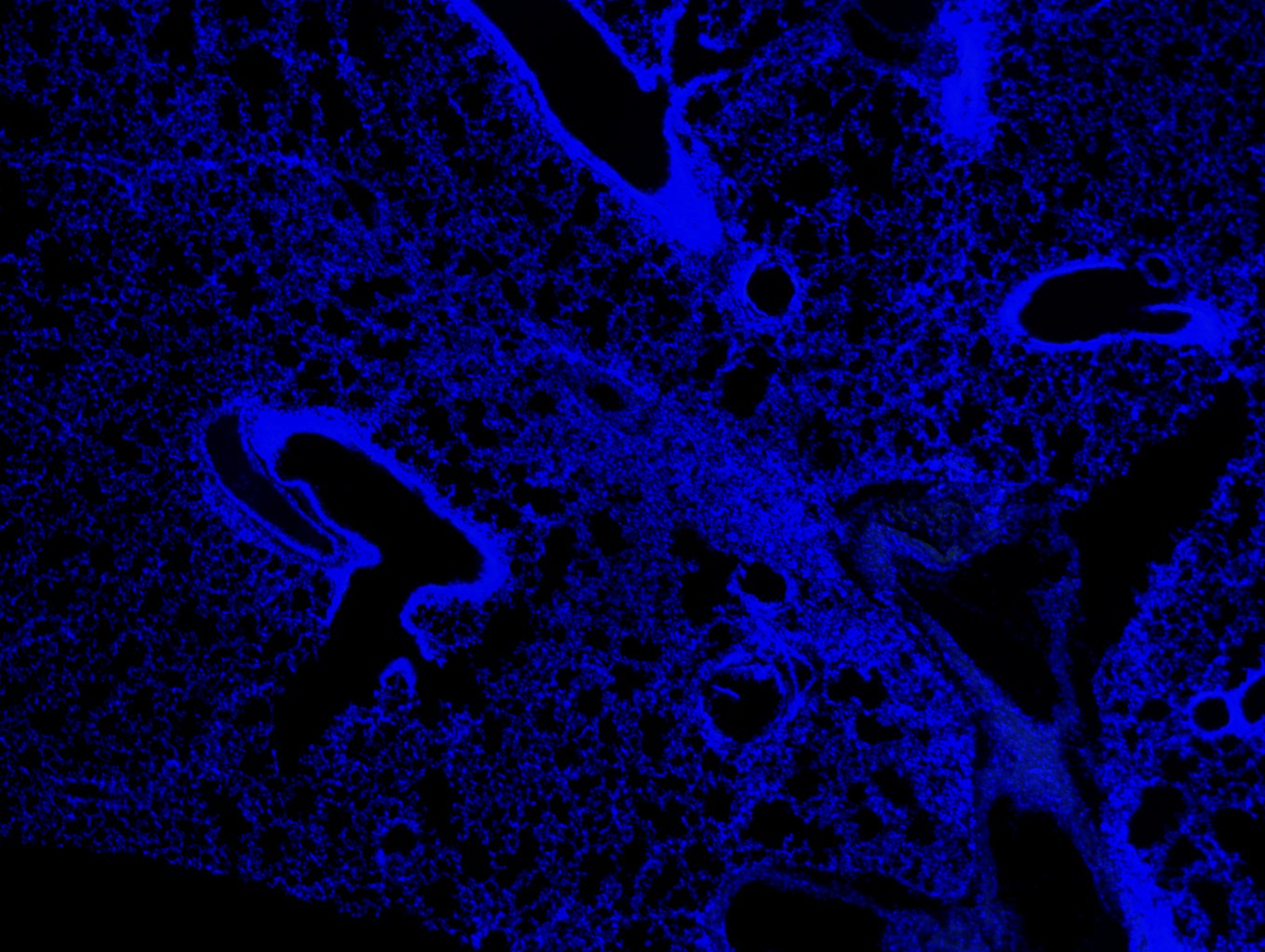

Supplement: Supplementary file 9 — Source data Fig. 6 [file 44321_2024_76_MOESM9_ESM.zip › Figure 6D/Figure 6D-IF/Fc-HA-NPs/Fc-HA-NP.jpg]

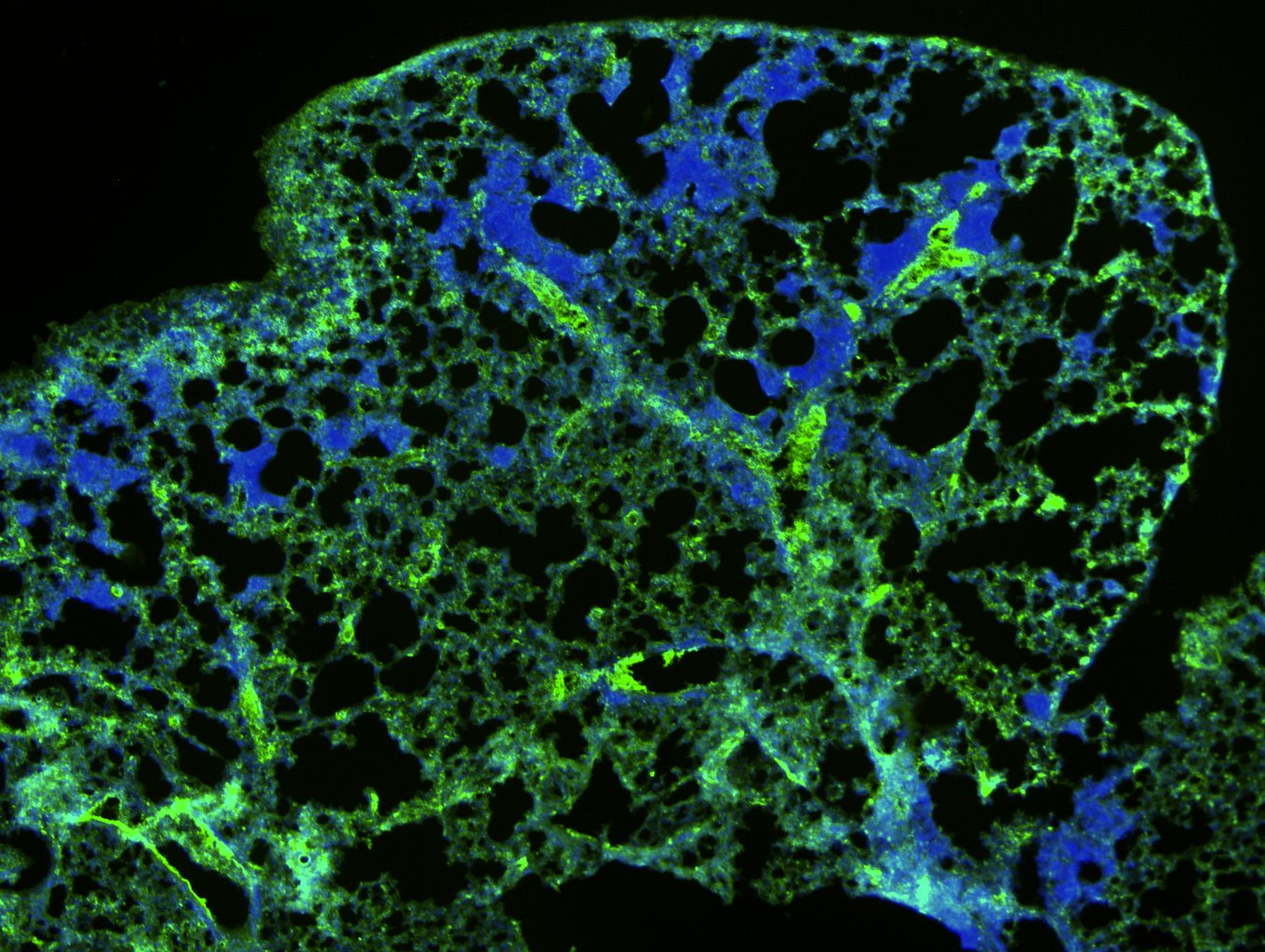

Supplement: Supplementary file 9 — Source data Fig. 6 [file 44321_2024_76_MOESM9_ESM.zip › Figure 6D/Figure 6D-IF/Fc-HA/Fc-HA-Merge.jpg]

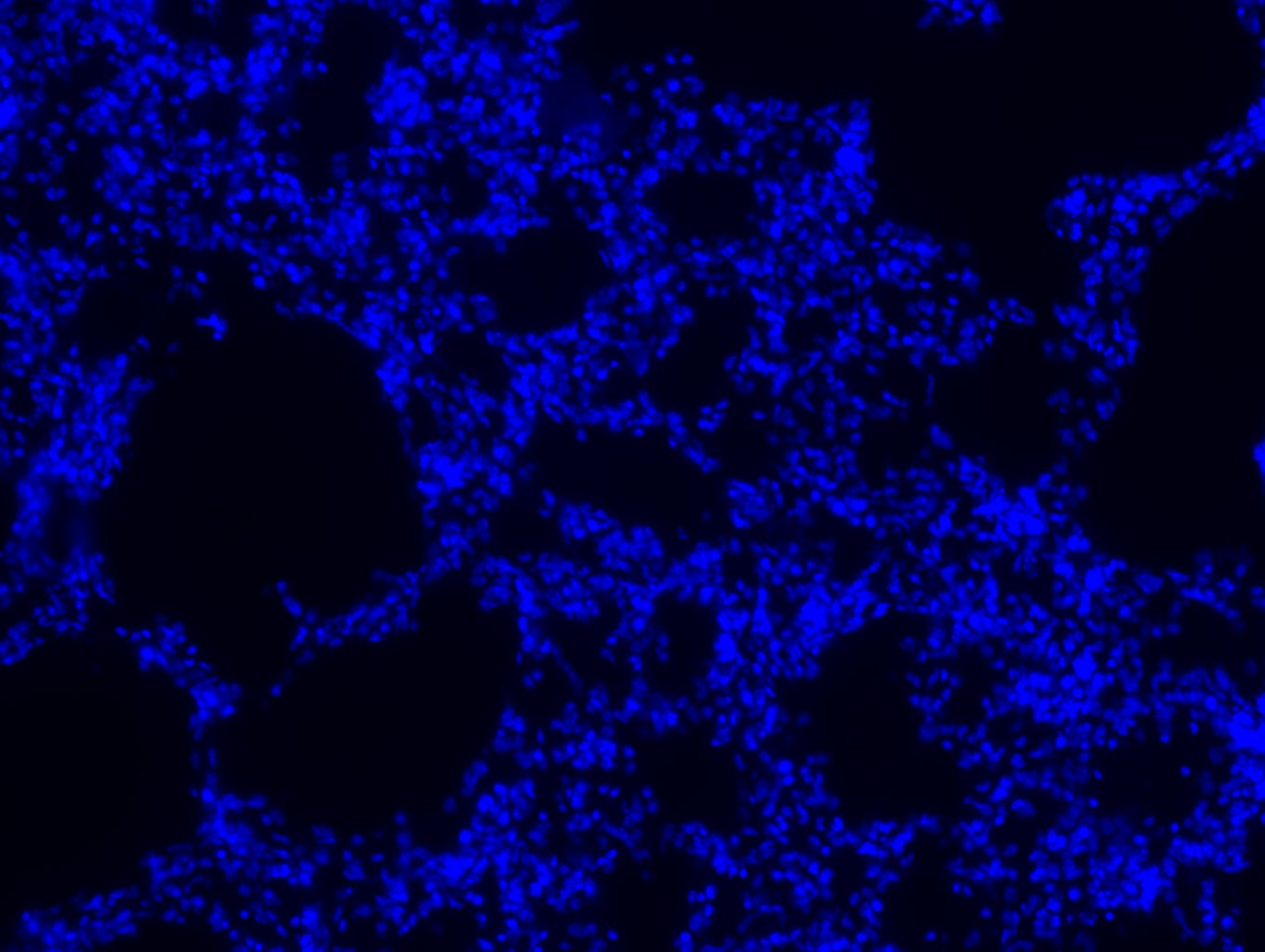

Supplement: Supplementary file 9 — Source data Fig. 6 [file 44321_2024_76_MOESM9_ESM.zip › Figure 6D/Figure 6D-IF/Fc-HA/Fc-HA-Zoom-DAPI.jpg]

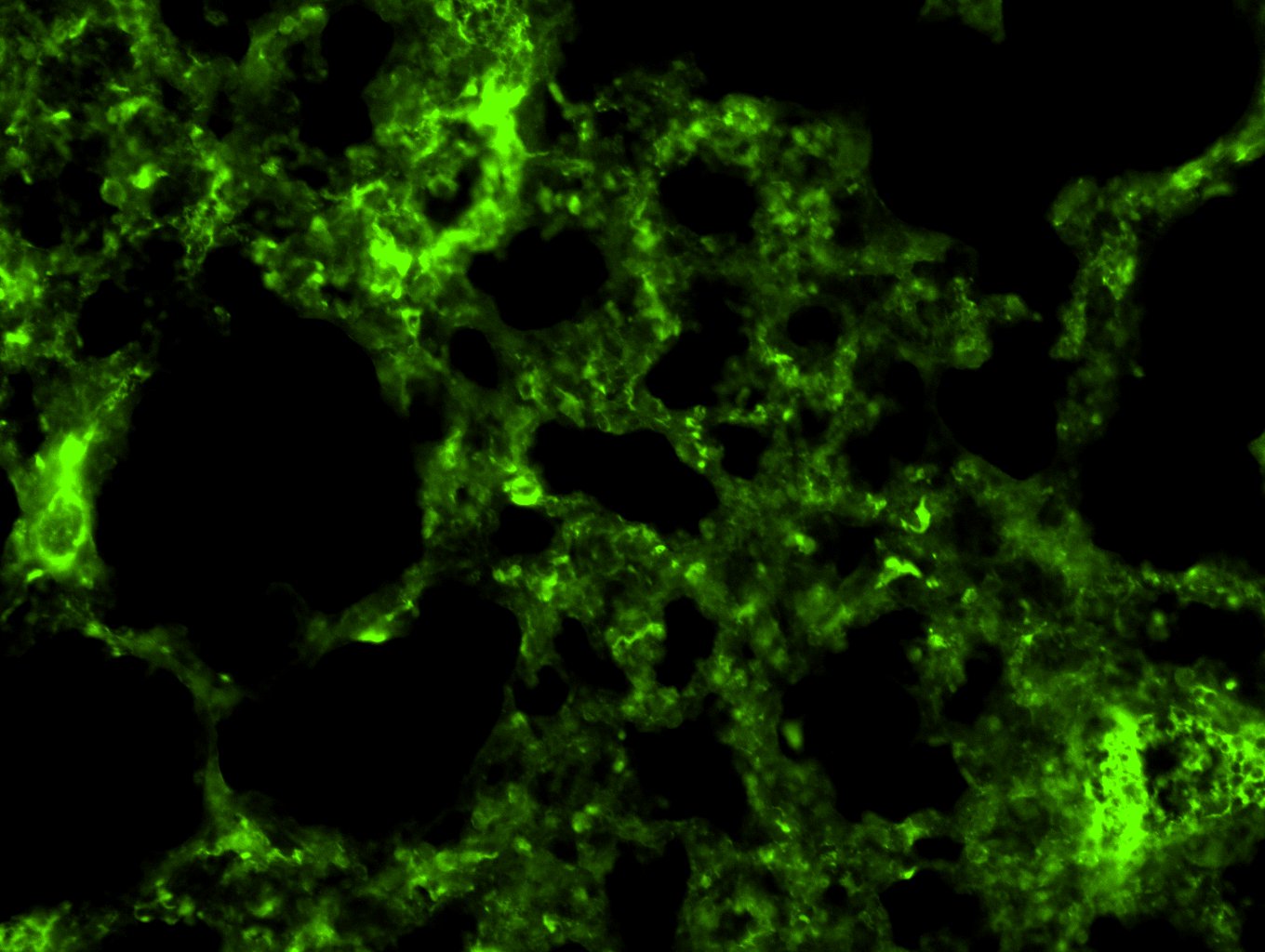

Supplement: Supplementary file 9 — Source data Fig. 6 [file 44321_2024_76_MOESM9_ESM.zip › Figure 6D/Figure 6D-IF/Fc-HA/Fc-HA-Zoom-GFP.jpg]

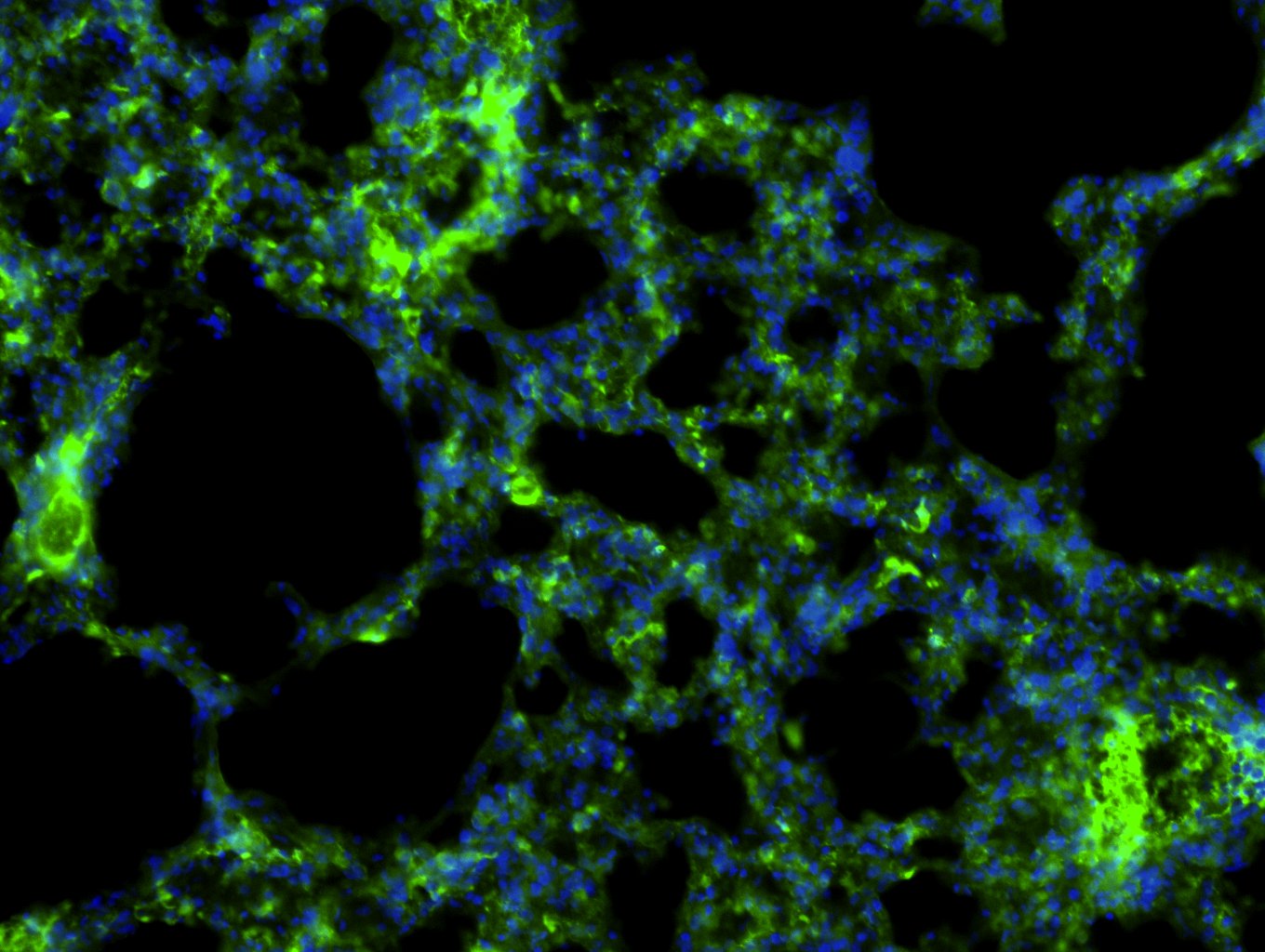

Supplement: Supplementary file 9 — Source data Fig. 6 [file 44321_2024_76_MOESM9_ESM.zip › Figure 6D/Figure 6D-IF/Fc-HA/Fc-HA-Zoom-merge.jpg]

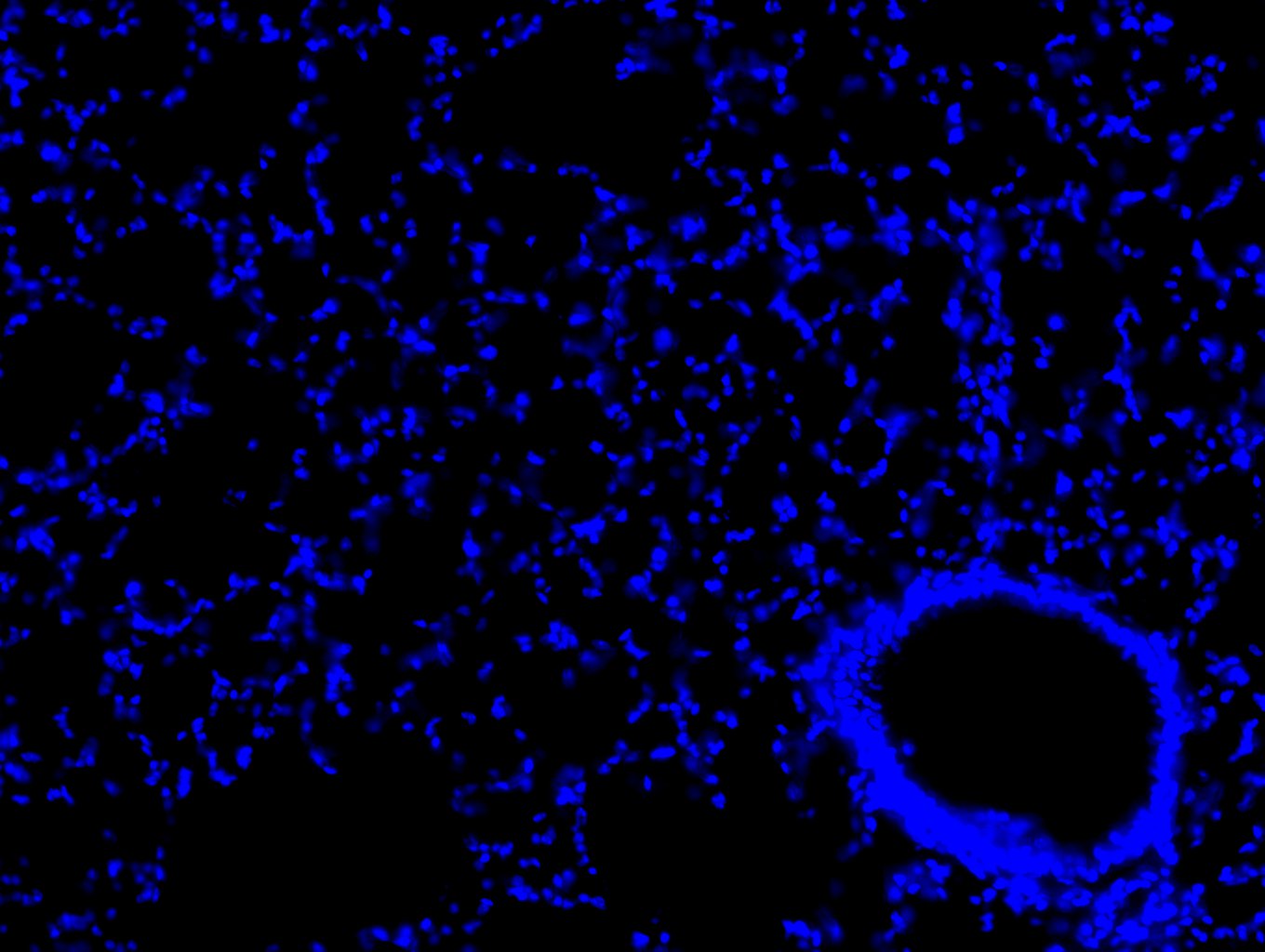

Supplement: Supplementary file 9 — Source data Fig. 6 [file 44321_2024_76_MOESM9_ESM.zip › Figure 6D/Figure 6D-IF/His-HA-NPs/His-HA-NP-zoom-DAPI.jpg]

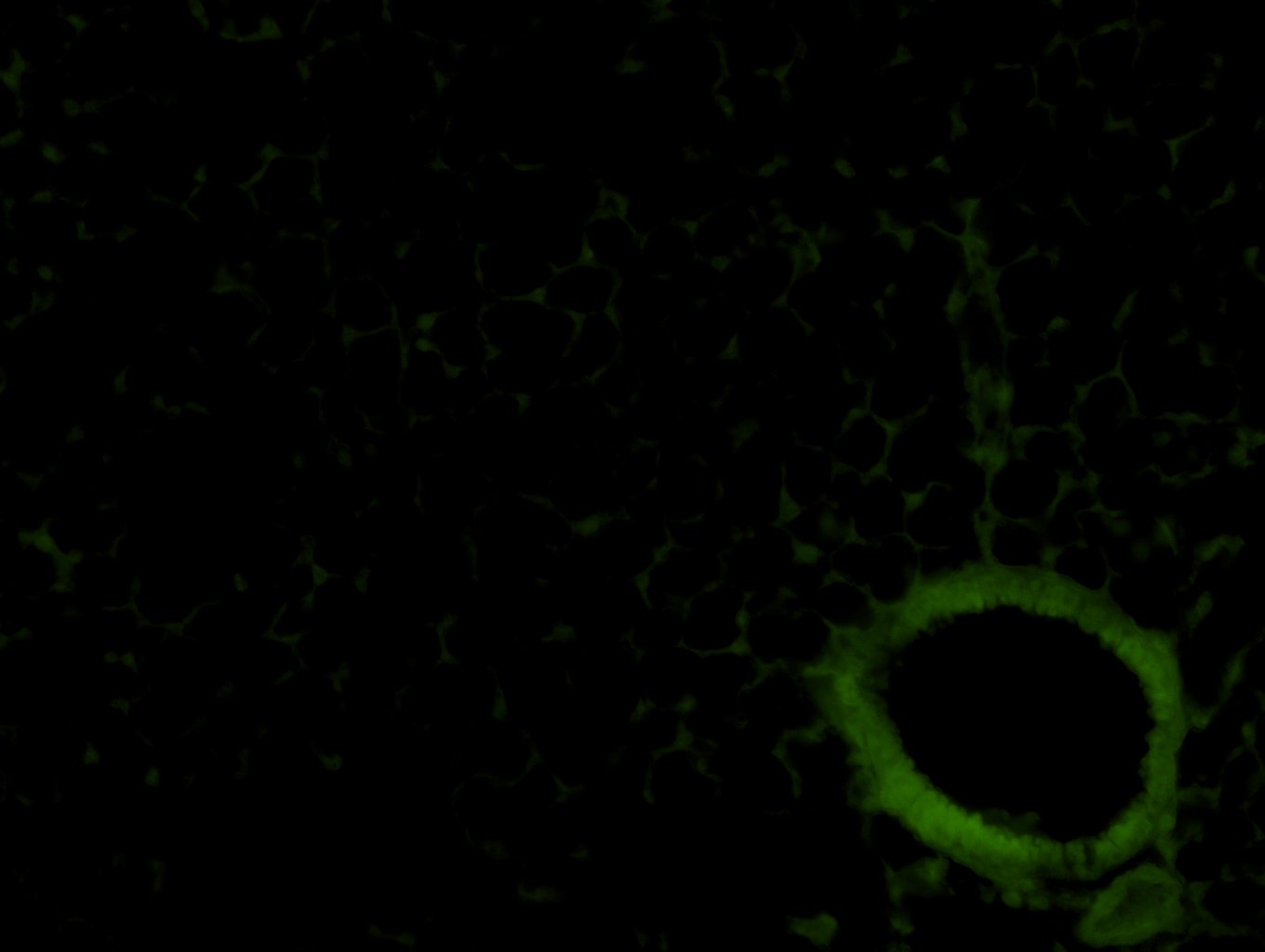

Supplement: Supplementary file 9 — Source data Fig. 6 [file 44321_2024_76_MOESM9_ESM.zip › Figure 6D/Figure 6D-IF/His-HA-NPs/His-HA-NP-zoom-GFP.jpg]

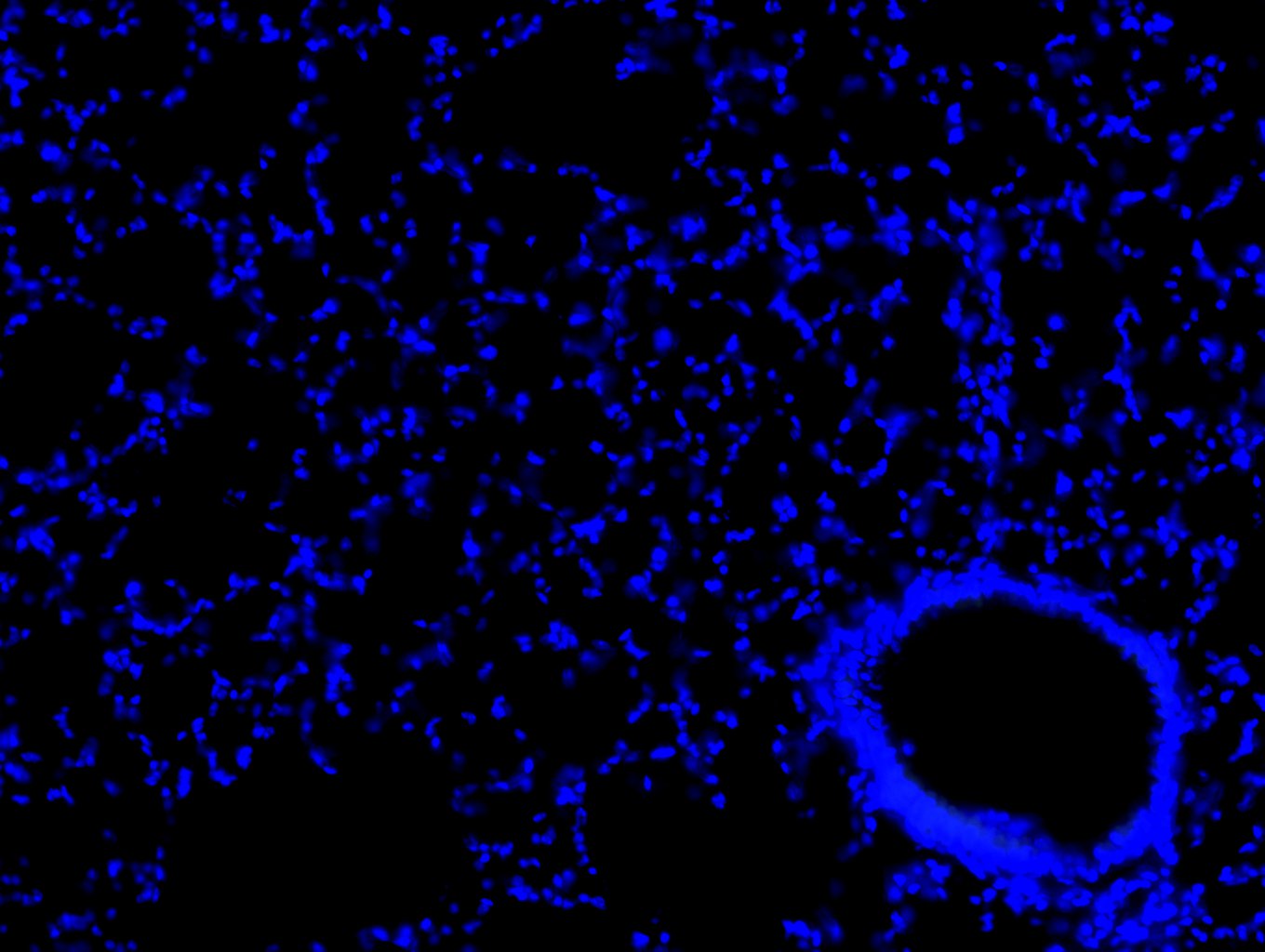

Supplement: Supplementary file 9 — Source data Fig. 6 [file 44321_2024_76_MOESM9_ESM.zip › Figure 6D/Figure 6D-IF/His-HA-NPs/His-HA-NP-zoom-Merge.jpg]

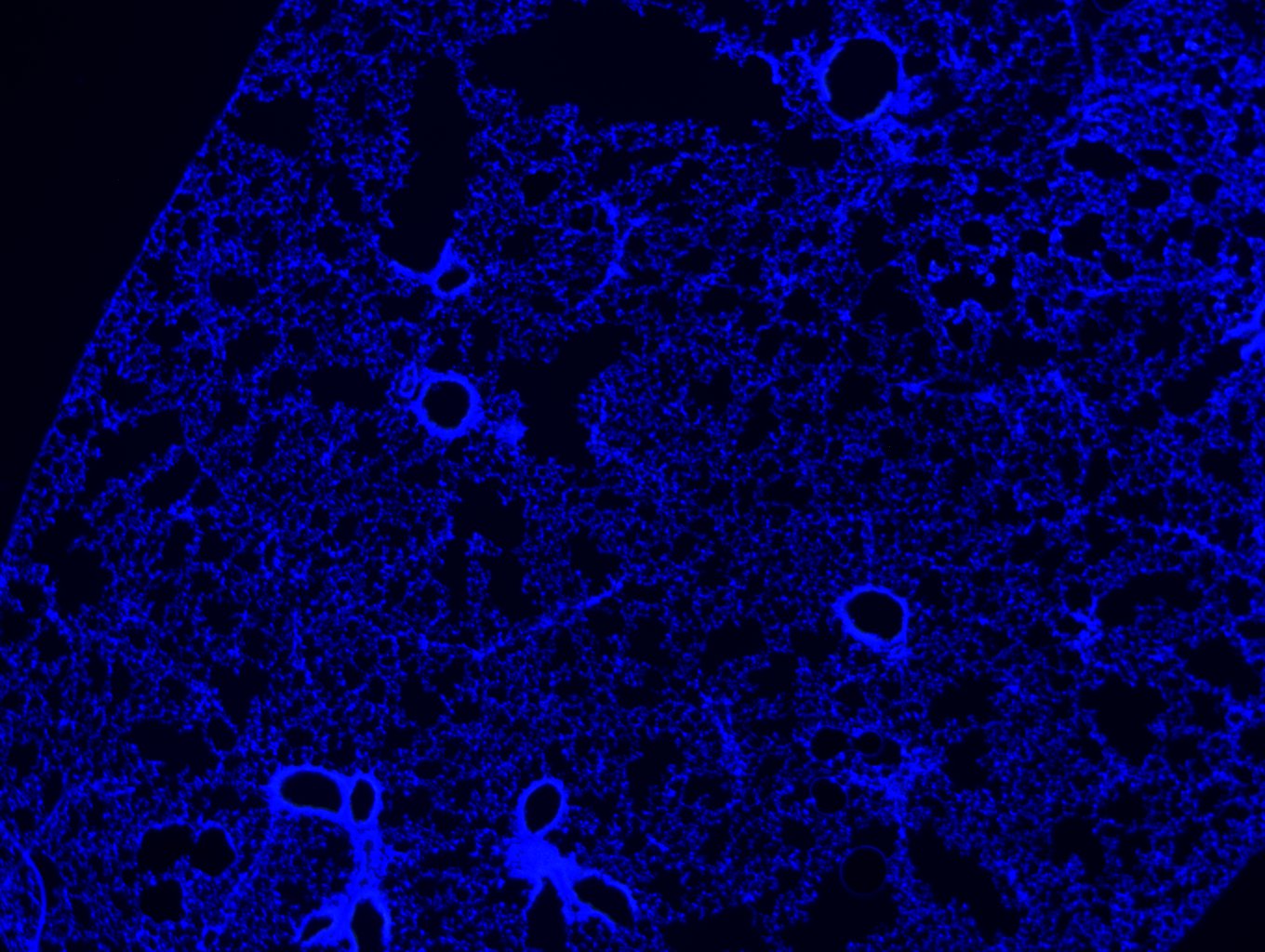

Supplement: Supplementary file 9 — Source data Fig. 6 [file 44321_2024_76_MOESM9_ESM.zip › Figure 6D/Figure 6D-IF/His-HA-NPs/His-HA-NP.jpg]

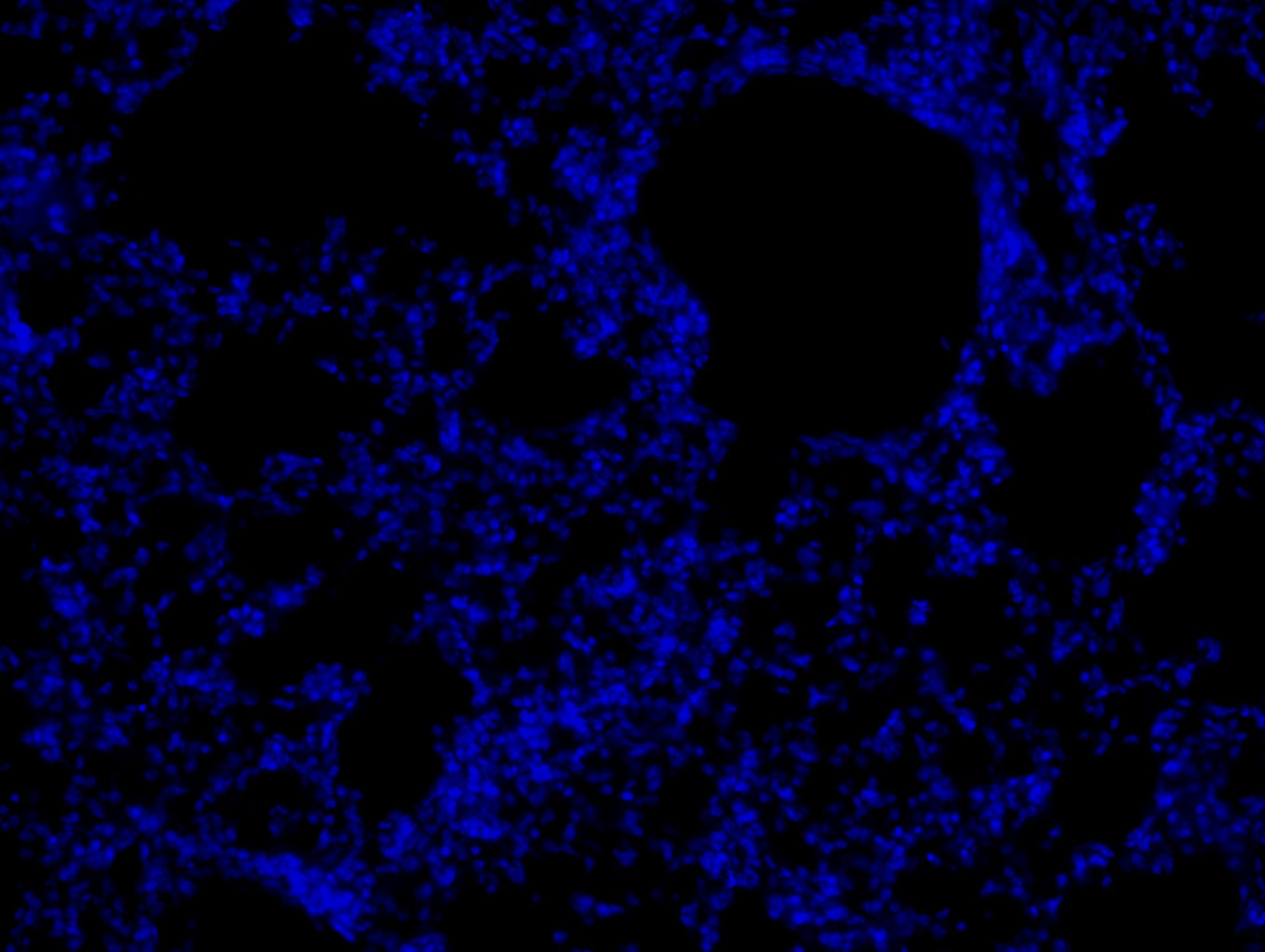

Supplement: Supplementary file 9 — Source data Fig. 6 [file 44321_2024_76_MOESM9_ESM.zip › Figure 6D/Figure 6D-IF/His-HA/His-HA-zoom-DAPI.jpg]

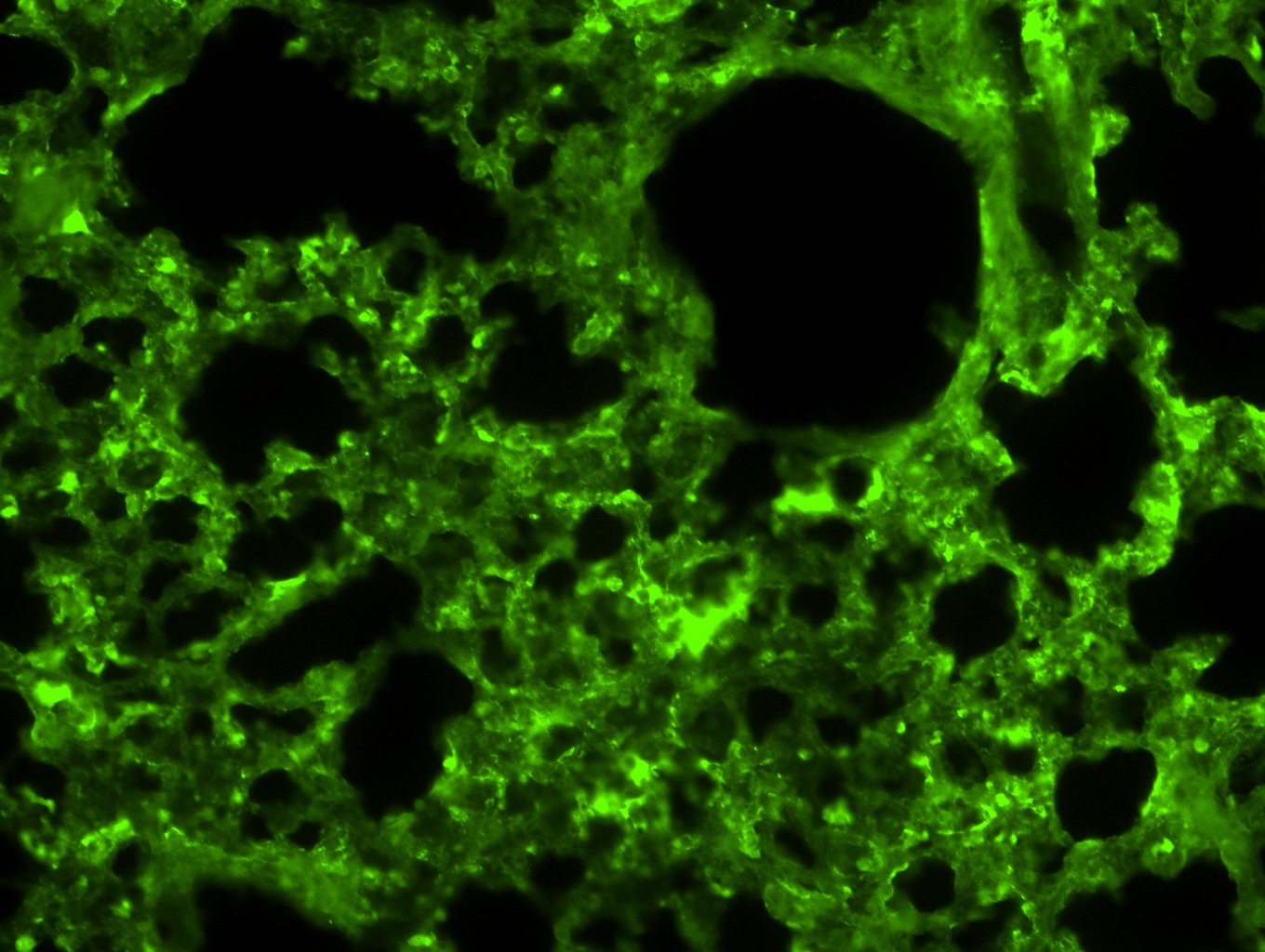

Supplement: Supplementary file 9 — Source data Fig. 6 [file 44321_2024_76_MOESM9_ESM.zip › Figure 6D/Figure 6D-IF/His-HA/His-HA-zoom-GFP.jpg]

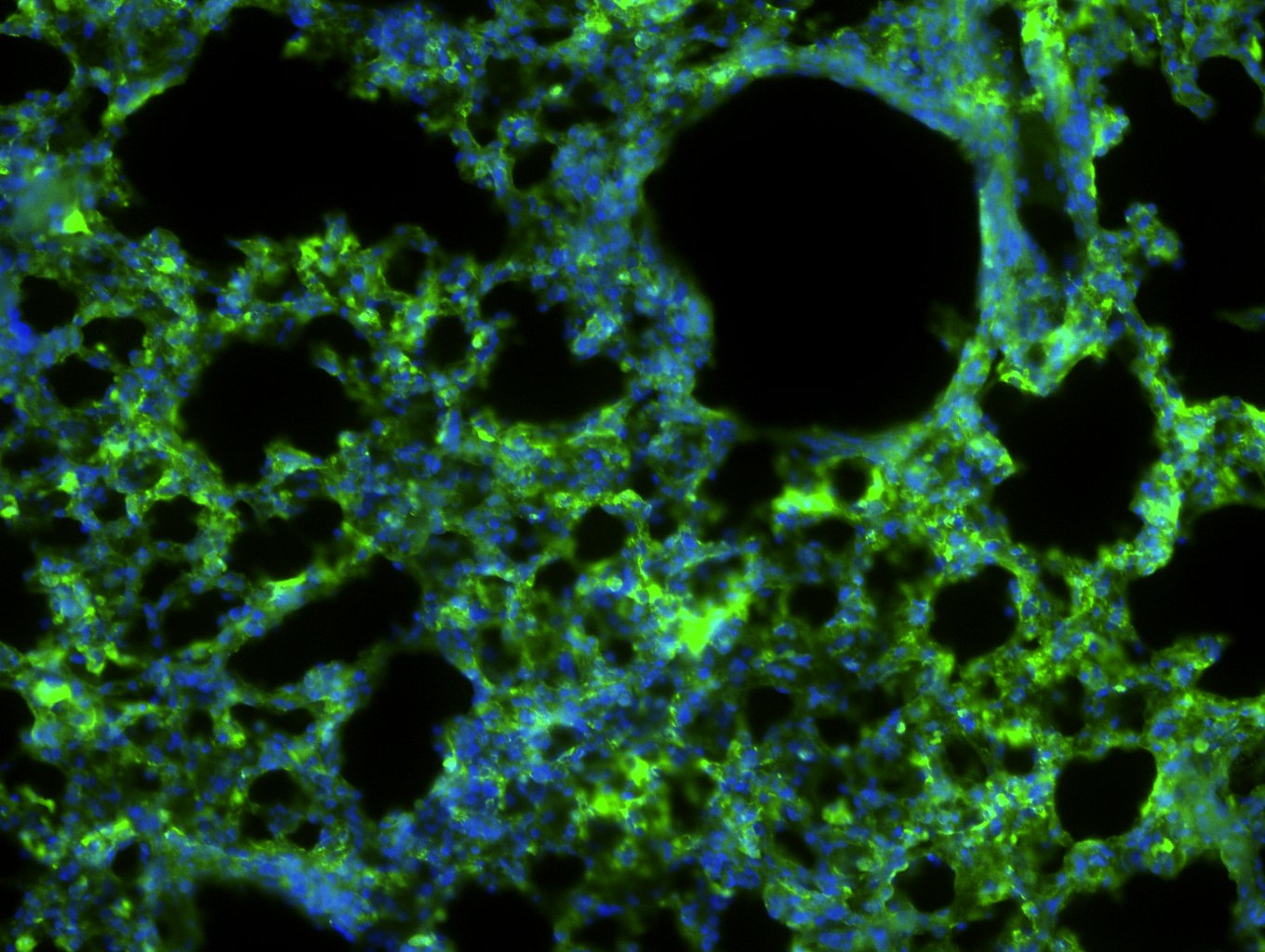

Supplement: Supplementary file 9 — Source data Fig. 6 [file 44321_2024_76_MOESM9_ESM.zip › Figure 6D/Figure 6D-IF/His-HA/His-HA-zoom-Merge.jpg]

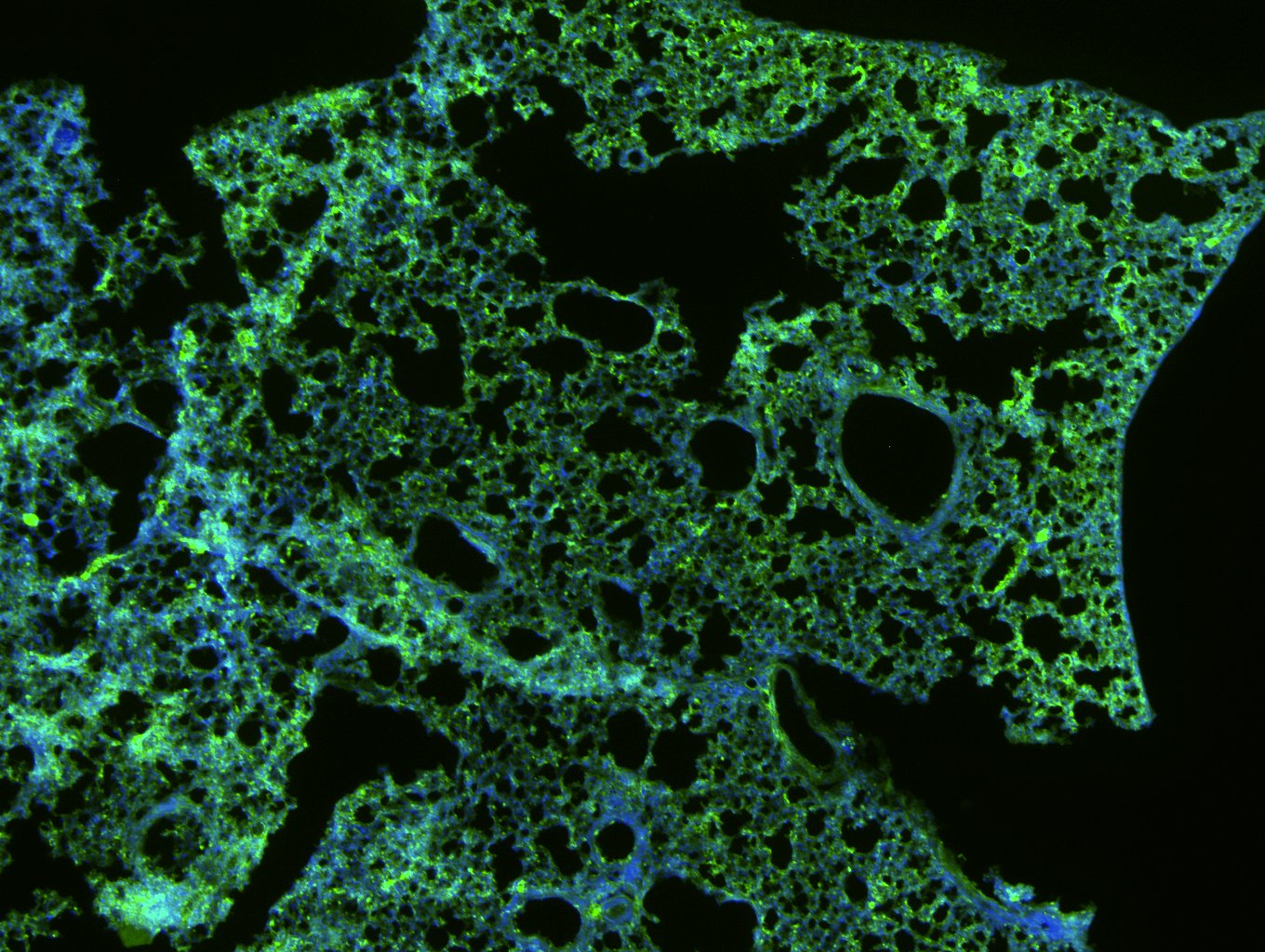

Supplement: Supplementary file 9 — Source data Fig. 6 [file 44321_2024_76_MOESM9_ESM.zip › Figure 6D/Figure 6D-IF/His-HA/His-HA.jpg]

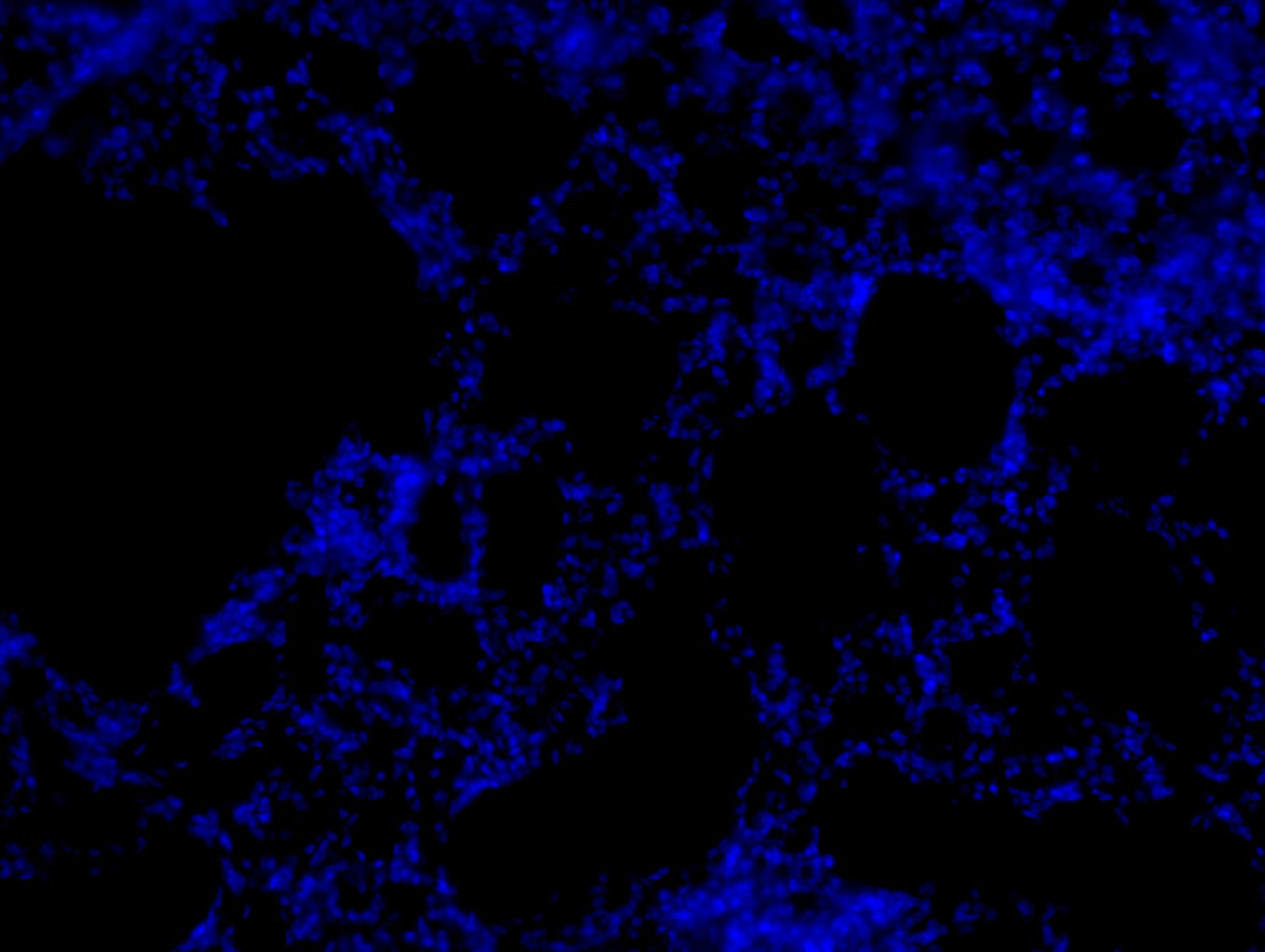

Supplement: Supplementary file 9 — Source data Fig. 6 [file 44321_2024_76_MOESM9_ESM.zip › Figure 6D/Figure 6D-IF/Mock/Mock-zoom-DAPI.jpg]

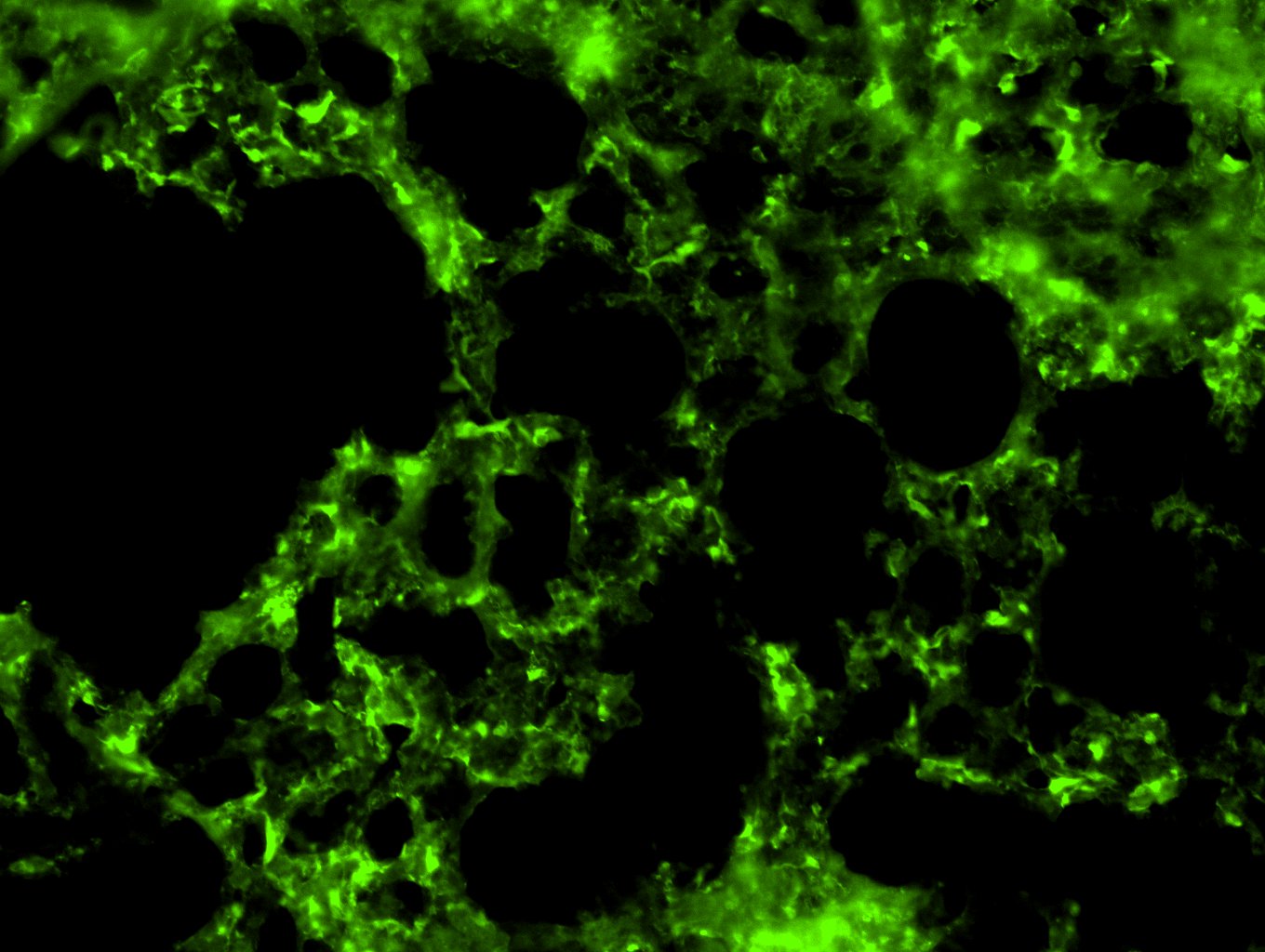

Supplement: Supplementary file 9 — Source data Fig. 6 [file 44321_2024_76_MOESM9_ESM.zip › Figure 6D/Figure 6D-IF/Mock/Mock-zoom-GFP.jpg]

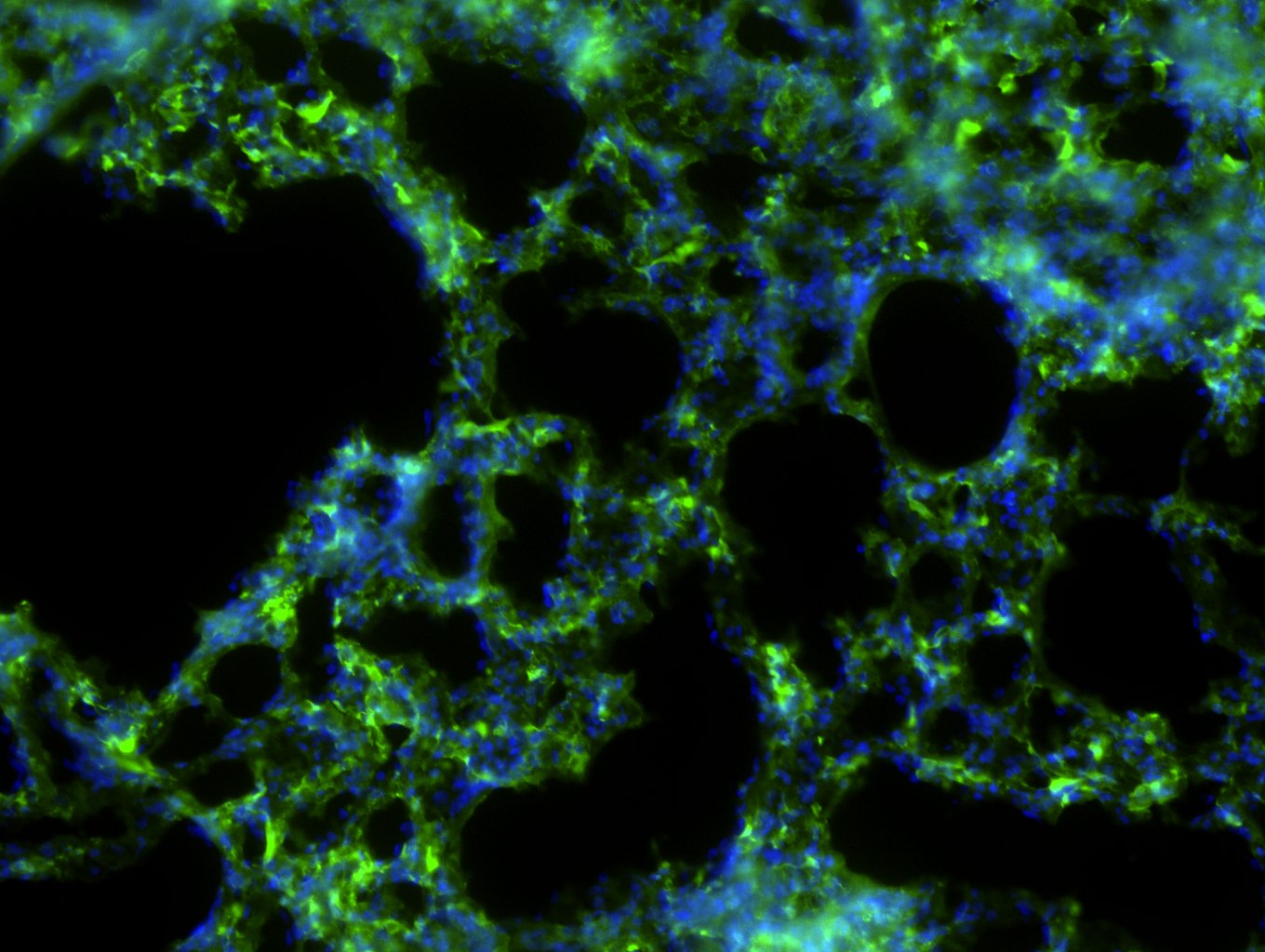

Supplement: Supplementary file 9 — Source data Fig. 6 [file 44321_2024_76_MOESM9_ESM.zip › Figure 6D/Figure 6D-IF/Mock/Mock-zoom-Merge.jpg]

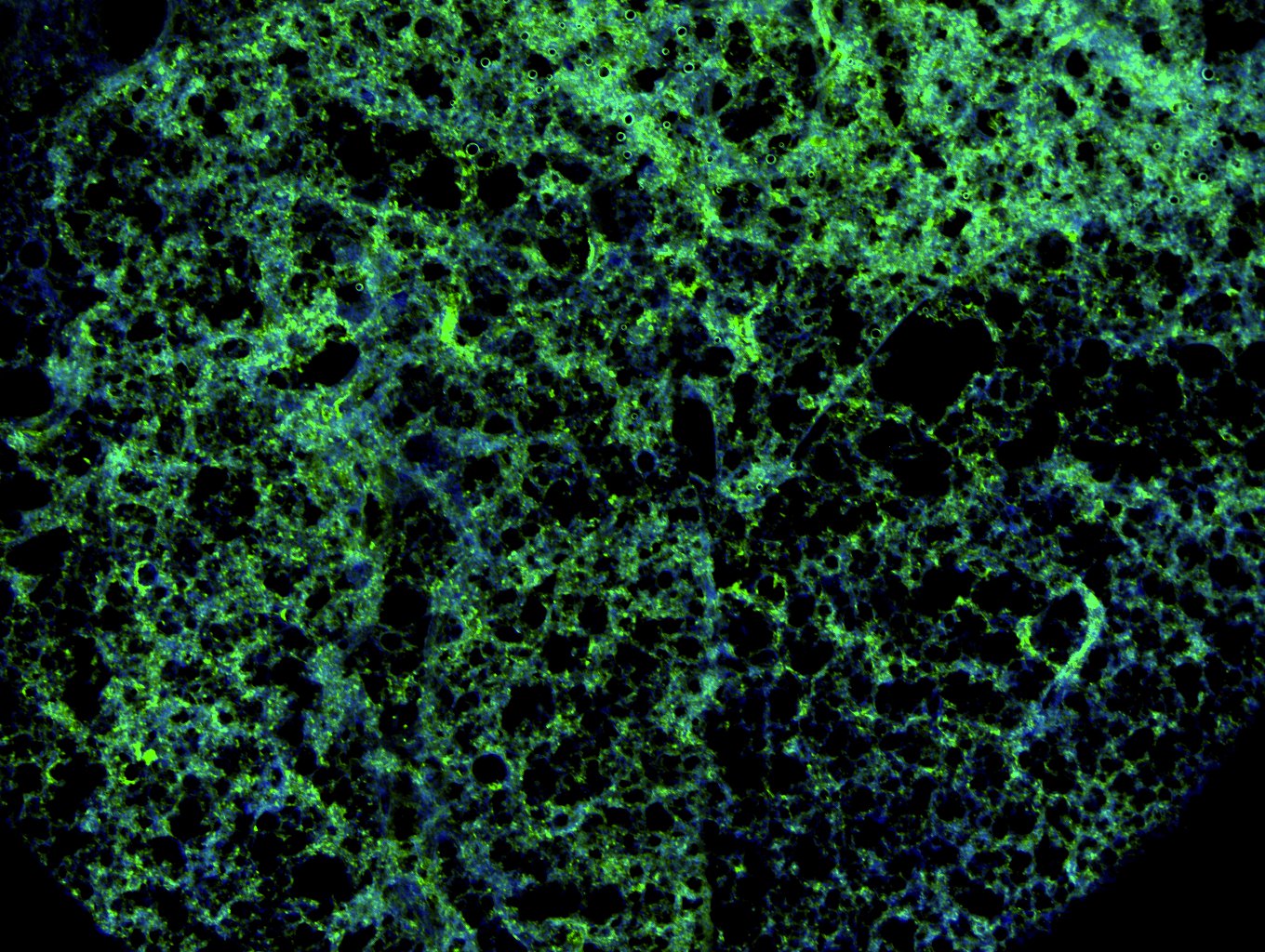

Supplement: Supplementary file 9 — Source data Fig. 6 [file 44321_2024_76_MOESM9_ESM.zip › Figure 6D/Figure 6D-IF/Mock/Mock.jpg]

## Slide 1
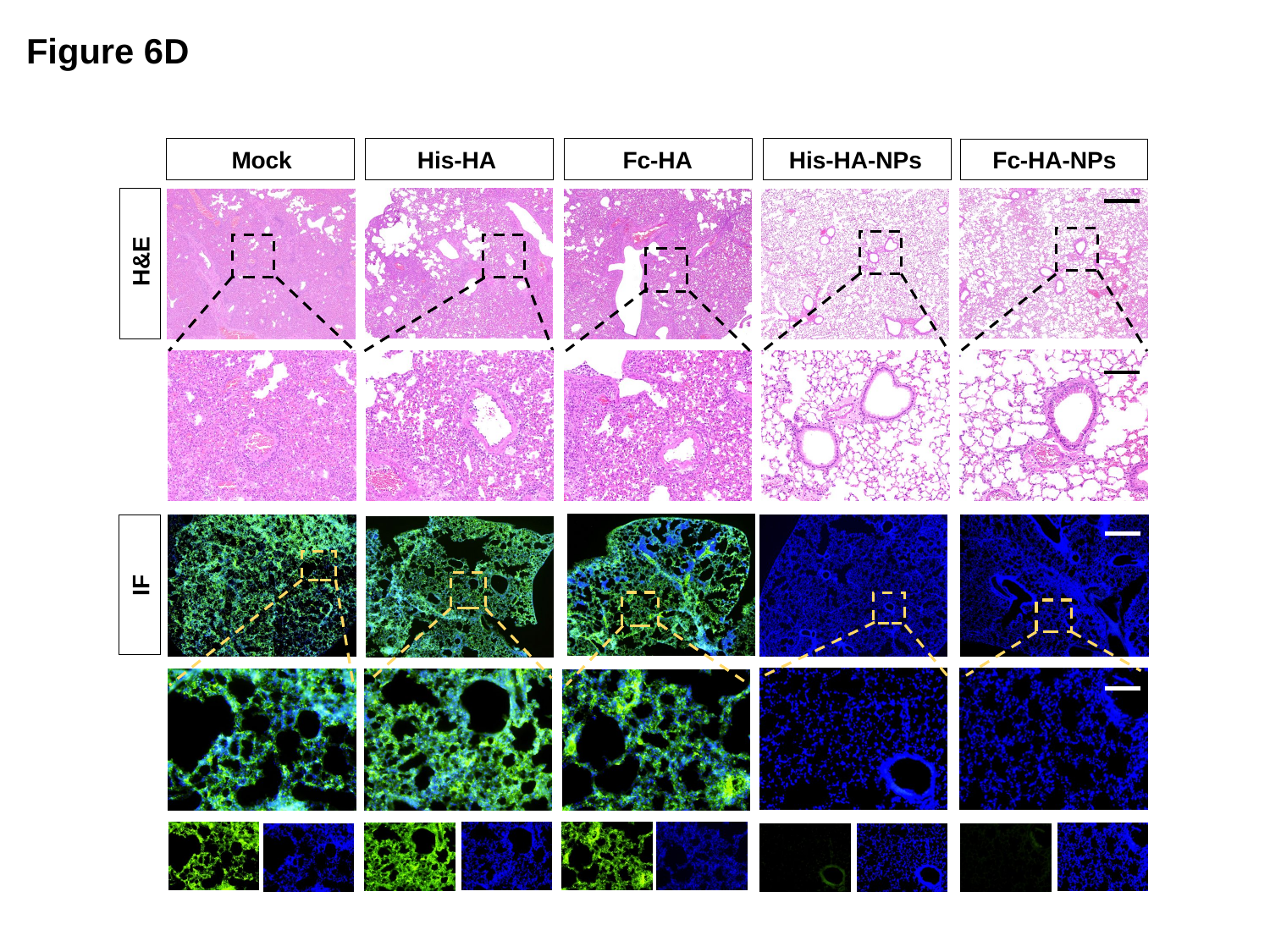

Figure 6D
 Mock
 His-HA
 Fc-HA
 His-HA-NPs
 Fc-HA-NPs
 H&E
 IF

Supplement: Supplementary file 9 — Source data Fig. 6 [file 44321_2024_76_MOESM9_ESM.zip › Figure 6D/Figure 6D.pptx]
