# Supplementary material for: A broadly applicable protein-polymer adjuvant system for antiviral vaccines
Source: EMBO Mol Med. 2024 May 15;16(6):1451–83. doi: 10.1038/s44321-024-00076-4 (PMC11178928; doi:10.1038/s44321-024-00076-4)
Supplement: Supplementary file 12 — Expanded View Figures [file 44321_2024_76_MOESM12_ESM.pdf]

Expanded View Figures

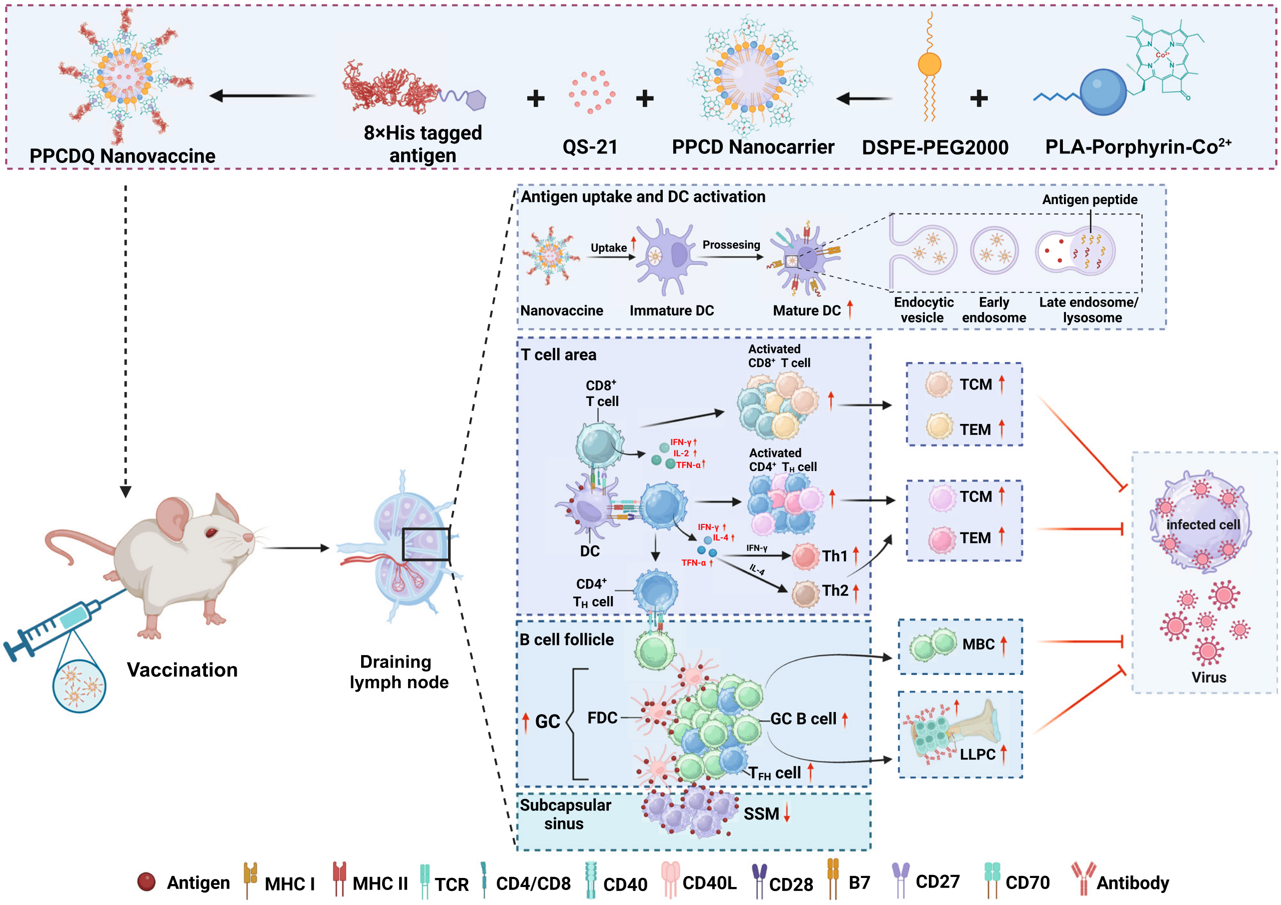

Figure EV1. Schematic illustration of the PPCDQ nanovaccine induce a humoral and cellular immune response against virus infection.

PPCDQ nanovaccine was synthesized by PLA-Porphyrin-Co^{2+}, DSPE-PEG2000, QS-21, and histidine-tagged antigen. After intramuscular injection, the PPCDQ nanovaccine drained into the lymph nodes, efficiently activated DCs and then activated T cells. Furthermore, the activated $CD4^+$ helper T cells (T_H cells) activated B cells to differentiate into plasma cells (PCs) to produce antibodies. Neutralizing antibodies and cytotoxic T lymphocytes (CTLs) against virus infection together. This figure was created with BioRender (https://biorender.com/).

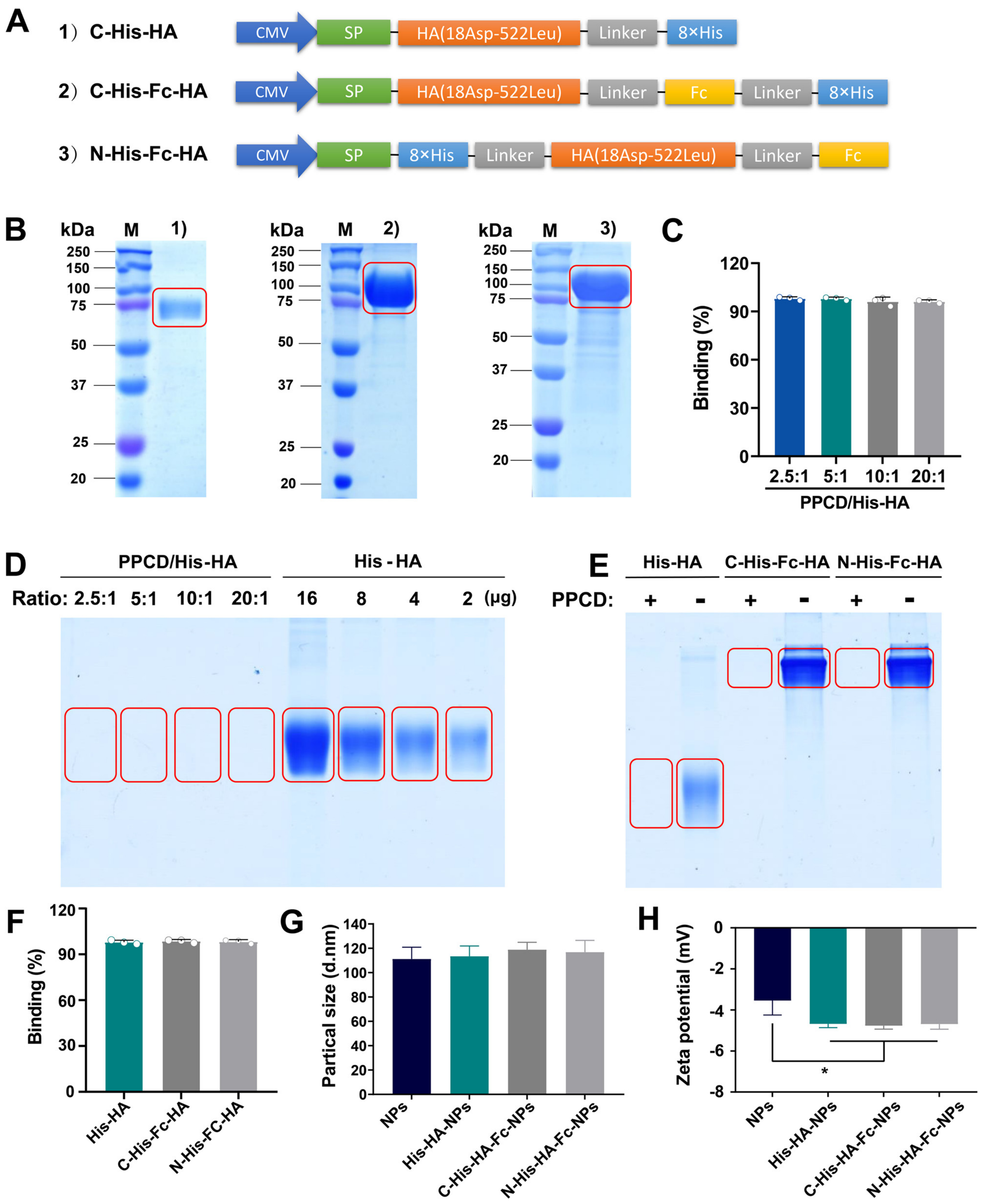
A
1) C-His-HA
CMV SP HA(18Asp-522Leu) Linker 8×His
2) C-His-Fc-HA
CMV SP HA(18Asp-522Leu) Linker Fc Linker 8×His
3) N-His-Fc-HA
CMV SP 8×His Linker HA(18Asp-522Leu) Linker Fc
B
kDa M 1)
kDa M 2)
kDa M 3)
250 150 100 75 50 37 25 20
C
Binding (%)
2.5:1 5:1 10:1 20:1
PPCD/His-HA
D
PPCD/His-HA
His - HA
Ratio: 2.5:1 5:1 10:1 20:1 16 8 4 2 (μg)
E
His-HA C-His-Fc-HA N-His-Fc-HA
PPCD: + - + - + -
F
Binding (%)
His-HA C-His-Fc-HA N-His-FC-HA
G
Partical size (d.nm)
NPs His-HA-NPs C-His-HA-Fc-NPs N-His-HA-Fc-NPs
H
Zeta potential (mV)
*
NPs His-HA-NPs C-His-HA-Fc-NPs N-His-HA-Fc-NPs

 © The Author(s)

Figure EV2. Efficiency of PPCD anchoring His-tagged proteins.

(**A**) Construction strategy of three expression plasmids. CMV was used as a promoter, an IgG1 signal peptide (SP) was fused to the N-terminal and an 8 × His tag was fused to the N-terminal or C-terminal. Fc tag was fused to the C-terminal of HA. (**B**) SDS-PAGE gel of C-His-HA (1), C-His-Fc-HA (2), and N-His-Fc-HA (3). (**C**, **D**) Native PAGE gel (**D**) and anchoring efficiency (**C**) of PPCD micelles with different proportions of His-HA proteins ($n = 3$ biological replicates per group). (**E**, **F**) Native PAGE gel (**E**) and statistical graph of binding efficiency (**F**) of PPCD micelles to three kinds of HA proteins ($n = 3$ biological replicates per group). (**G**, **H**) Practical size (**G**) and zeta potential (**H**) of PPCDQ combined with or without protein ($n = 3$ biological replicates per group). Data information: Data in (**C**, **F**, **G**, **H**) are mean ± SD, statistical analysis in (**H**) was performed by one-way ANOVA with Tukey's multiple comparisons test. *$P < 0.05$.

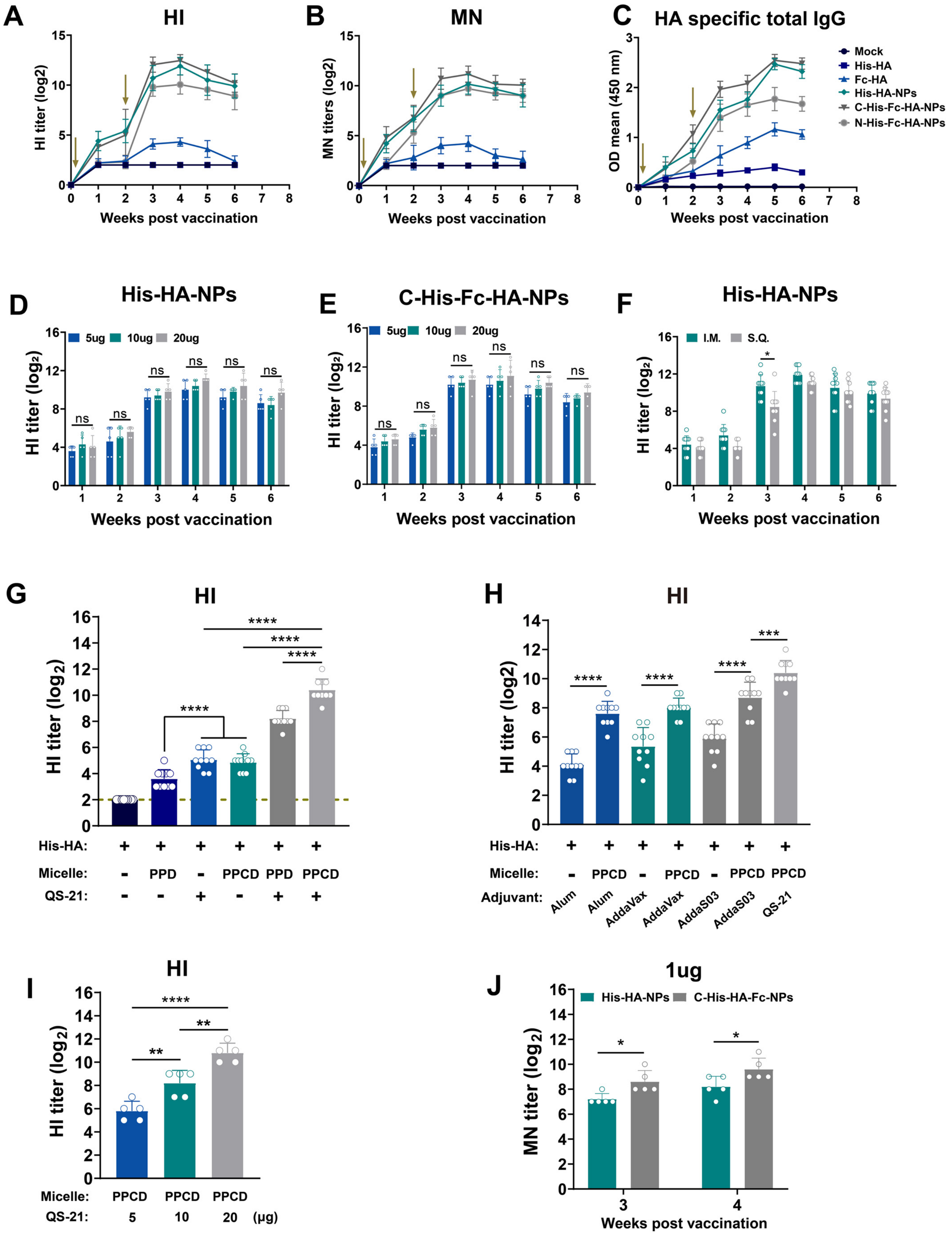
A
HI
HI titer (log2)
Weeks post vaccination
B
MN
MN titers (log2)
Weeks post vaccination
C
HA specific total IgG
OD mean (450 nm)
Weeks post vaccination
Mock
His-HA
Fc-HA
His-HA-NPs
C-His-Fc-HA-NPs
N-His-Fc-HA-NPs
D
His-HA-NPs
5ug 10ug 20ug
HI titer (log₂)
Weeks post vaccination
E
C-His-Fc-HA-NPs
5ug 10ug 20ug
HI titer (log₂)
Weeks post vaccination
F
His-HA-NPs
I.M. S.Q.
HI titer (log₂)
Weeks post vaccination
G
HI
HI titer (log₂)
His-HA: + + + + + +
Micelle: - PPD - PPCD PPD PPCD
QS-21: - - + - + +
H
HI
HI titer (log2)
His-HA: + + + + + + +
Micelle: - PPCD - PPCD - PPCD PPCD
Adjuvant: Alum Alum AddaVax AddaVax AddaS03 AddaS03 QS-21
I
HI
HI titer (log₂)
Micelle: PPCD PPCD PPCD
QS-21: 5 10 20 (μg)
J
1ug
His-HA-NPs C-His-HA-Fc-NPs
MN titer (log₂)
Weeks post vaccination

 © The Author(s)

Figure EV3. The synergistic effect of PPCD and QS-21 is critical for antibody production.

(**A–C**) HI titers (**A**), MN titers (**B**), and HA-specific total IgG (**C**) of immunized C57/BL6 mice at each time point ($n = 10$ animals per group). Arrows represent the time points of vaccination. (**D**, **E**) C57/BL6 mice were immunized with different dose (5, 10, and 20 µg) of His-HA-NPs (**D**) or C-His-Fc-HA-NPs (**E**). HI titers were detected at indicated time point ($n = 5$ animals per group). (**F**) C57/BL6 mice were intramuscularly or subcutaneously immunized with 5 µg His-HA-NPs and HI titers of each time point were tested ($n = 10$ animals per group). (**G**) C57/BL6 mice were immunized with different formulations and HI titers were detected at 3 weeks post vaccination ($n = 10$ animals per group). (**H**) C57/BL6 mice were immunized intramuscularly with the mixture of different adjuvants (Alum, AddaVax, AddaS03, and QS-21) and His-HA or His-HA@PPCD, respectively. HI titers were detected at 3 weeks post vaccination ($n = 10$ animals per group). (**I**) C57/BL6 mice were immunized with His-HA@PPCDQ containing different doses of QS-21(5, 10, and 20 µg). HI titers were detected at 3 weeks post vaccination ($n = 5$ animals per group). (**J**) C57/BL6 mice were immunized intramuscularly with 1 µg dose of His-HA-NPs or C-His-Fc-HA-NPs and MN titers were detected at 3 and 4 weeks post vaccination ($n = 5$ animals per group).
Data information: Data are presented as mean ± SD, statistical analysis in (**D–F**, **J**) was determined by two-way ANOVA with Tukey's multiple comparisons test. statistical significance in (**G–I**) was determined by one-way ANOVA with Tukey's multiple comparisons test. *$P < 0.05$, **$P < 0.01$, ***$P < 0.001$, ****$P < 0.0001$.

© The Author(s)

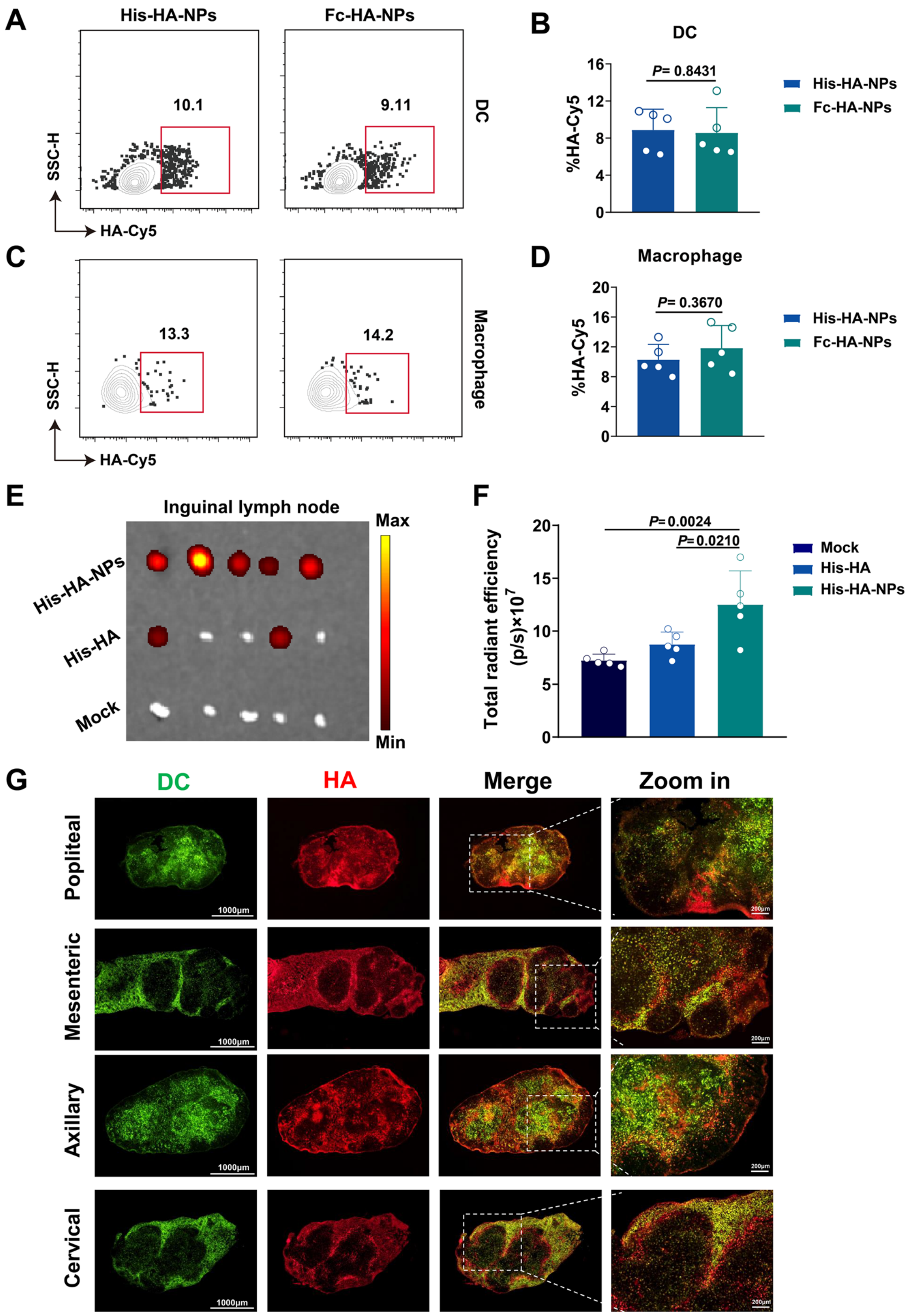

Figure EV4. His-HA-NPs rapidly accumulate in inguinal lymph nodes and flow to remote lymph nodes.

(**A**) Representative flow cytometric plots of Cy5 positive DCs (B220$^-$CD11c$^+$MHC-II$^+$, top panel) in iLNs at 4 h post injection. (**B**) Statistical graphs of Cy5 positive DCs ($n = 5$ animals per group). (**C**) Representative flow cytometric plots of Cy5 positive macrophages (B220$^-$CD11b$^+$F4/80$^+$). (**D**) Statistical graphs of Cy5 positive macrophages ($n = 5$ animals per group). (**E**) ILNs were harvested at 4 h post injection for the living image by IVIS Spectrum system. (**F**) Statistical graphs of the total radiant efficiency analyzed by Living Image Vision 4.4 ($n = 5$ animals per group). (**G**) Transgenic mice CD11c-EYFP were immunized with equal mass of iFlour^{TM} 594-tagged His-HA or His-HA-NPs. Inguinal, popliteal, mesenteric, axillary, and cervical lymph nodes were obtained at 4 h post injection. Antigen distribution of these lymph nodes was shown by the cryosections. Scalar bar: 1000 μm (left), 200 μm (right). Data information: Data in (**B**, **D**, **F**) are mean ± SD, statistical analysis was determined by one-way ANOVA with Tukey's multiple comparisons test.

 © The Author(s)

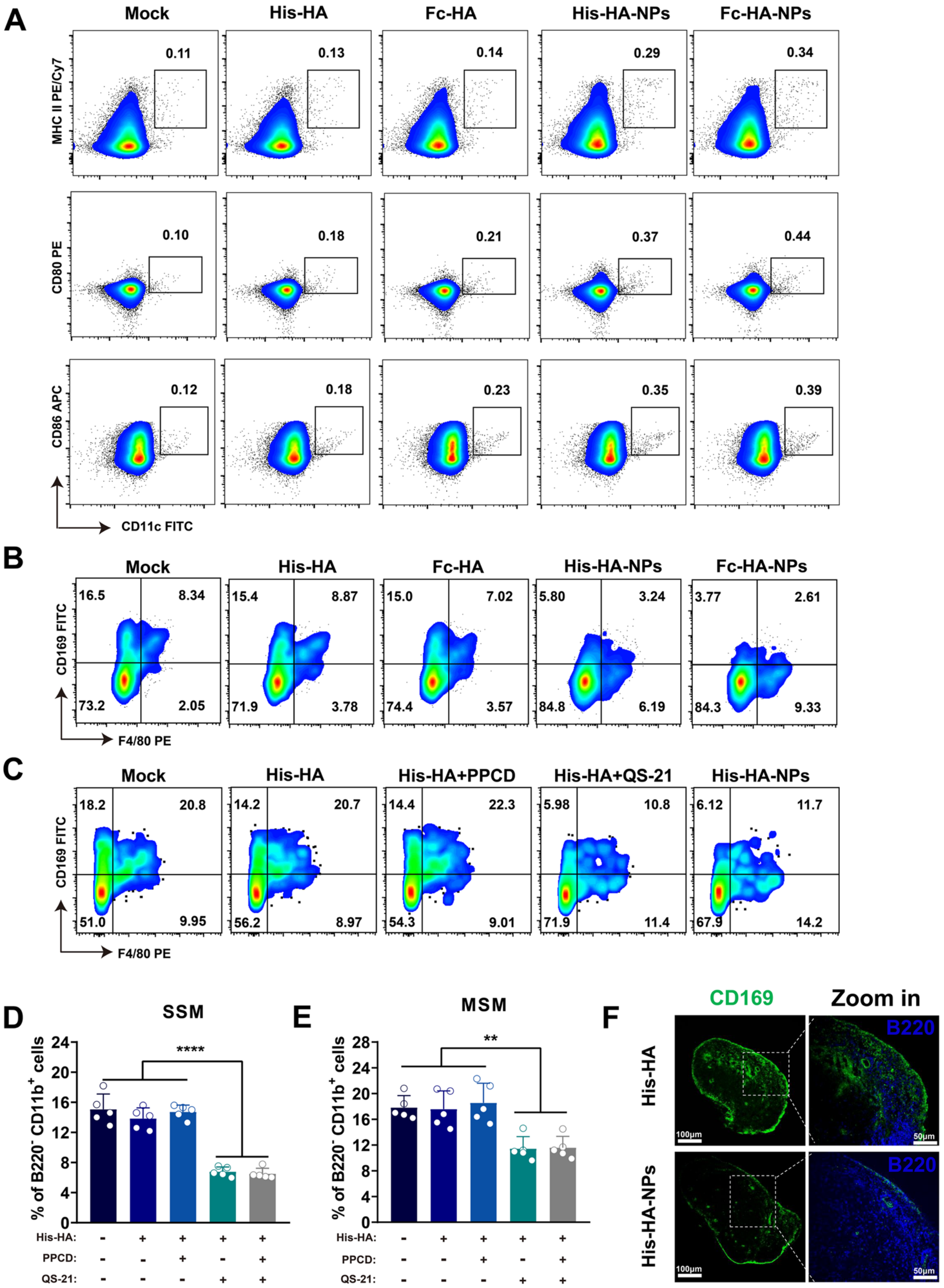

Figure EV5. HA@PPCDQ promotes DC activation and depletes CD169$^+$ subcapsular sinus macrophages.

(**A**) Representative flow cytometric plots of CD11c$^+$MHC-II$^+$DCs, CD11c$^+$CD80$^+$ DCs, and CD11c$^+$CD86$^+$DCs in iLNs at 2 days post boost immunization. (**B**) Representative flow cytometric plots of subcapsular sinus macrophage (SSM, B220$^-$CD11b$^+$F4/80$^-$CD169$^+$) and medullary sinus macrophage (MSM, B220$^-$CD11b$^+$F4/80$^+$CD169$^+$) in iLN. (**C–E**) C57/BL6 mice were immunized with different formulations, and iLNs were collected for FCM. (**C**) Representative flow cytometric plots of SSM and MSM. Statistical graphs of SSM (**D**) and MSM (**E**) ($n = 5$ animals per group). (**F**) Cryosections of inguinal lymph nodes were incubated with FITC anti-mouse CD169 (Siglec-1) and Brilliant Violet 421™ anti-mouse/human CD45R/B220. Images were taken on a Nikon super-resolution spinning-disk confocal microscopy. Scalar bar: 100 μm (left) and 50 μm (right). Data information: Data in (**D**), and (**E**) are mean ± SD, statistical analysis was determined by one-way ANOVA with Tukey's multiple comparisons test. $^{**}P < 0.01$, $^{****}P < 0.0001$.

© The Author(s)
